# New anionic cobalt(III) complexes enable enantioselective synthesis of spiro-fused oxazoline and iodoacetal derivatives

**DOI:** 10.3389/fchem.2022.1034291

**Published:** 2022-10-13

**Authors:** Mohamed S. H. Salem, Shinobu Takizawa

**Affiliations:** ^1^ SANKEN, Osaka University, Ibaraki, Japan; ^2^ Pharmaceutical Organic Chemistry Department, Faculty of Pharmacy, Suez Canal University, Ismailia, Egypt

**Keywords:** cobalt(III) complexes, phase-transfer catalyst, iodoacetalization, iodocyclization, enantioselective synthesis, chiral-at-metal

## Abstract

Anionic salicylimine-based cobalt (III) complexes featuring chiral ligands derived from isoleucine amino acids were used as efficient bifunctional phase-transfer catalysts for electrophilic iodination of enol ethers. The Brønsted acids of these complexes enabled the enantioselective asymmetric iodocyclization of enol ethers, furnishing spiro-fused oxazoline derivatives in high yields with up to 90:10 er. In addition, chiral cobalt (III) complexes catalyze the asymmetric intermolecular iodoacetalization of enol ethers with various alcohols to afford 3-iodoacetal derivatives in high yields with up to 92:8 er.

## 1 Introduction

**SCHEME 1:**
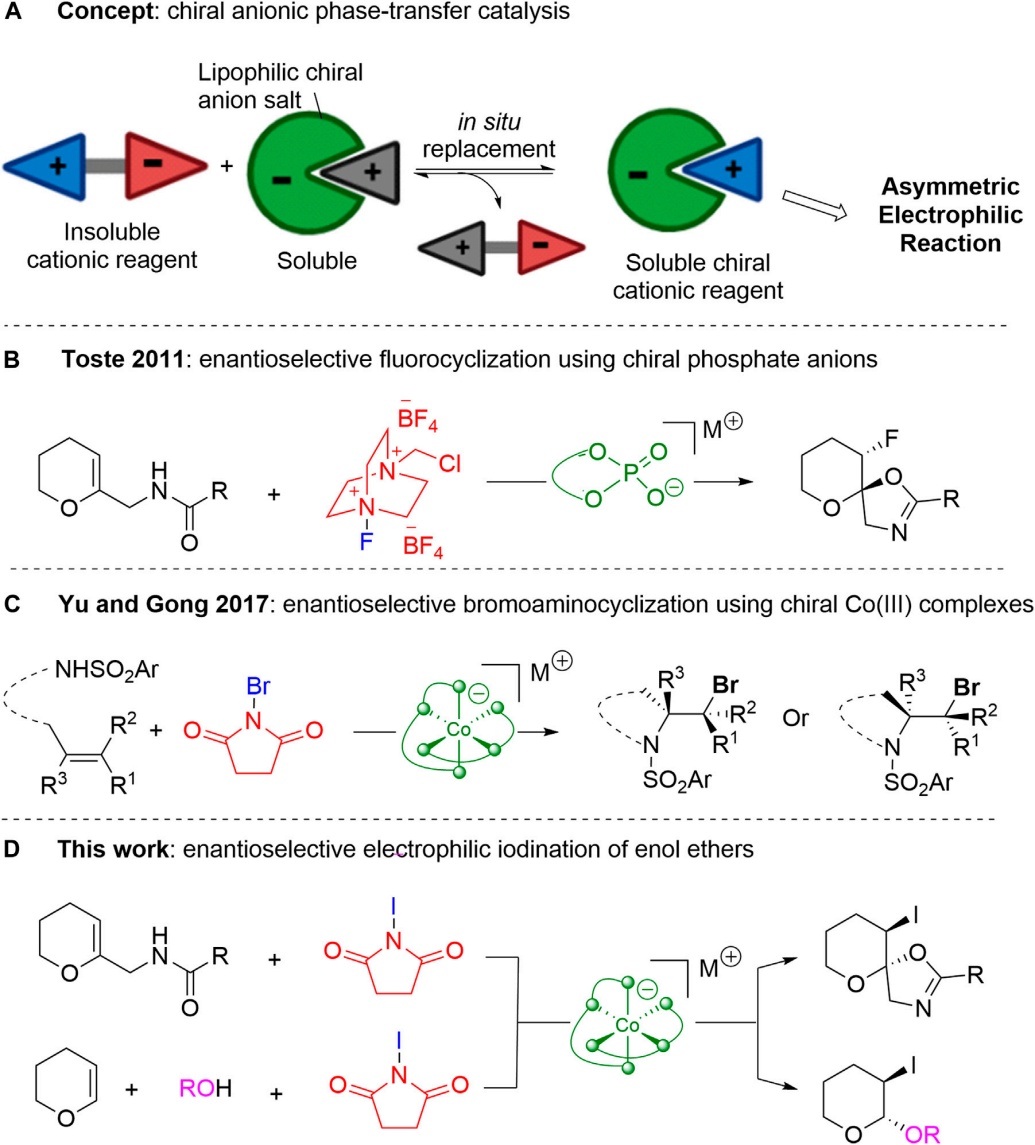
Chiral Anion-mediated Enantioselective Halocyclization. **(A)** Concept of anionic phase transfer catalysis. **(B)** Chiral phosphate anion-mediated enantioselective fluorocyclization. **(C)** Chiral cobalt anion-mediated enantioselective bromoaminocyclization. **(D)** Chiral cobalt anion-mediated enantioselective iodocyclization and iodoacetalization of enol ethers.

Chiral anionic phase-transfer catalysis has emerged as a distinct strategy to achieve various stereoselective transformations (Phipps et al., 2012; Kaneko et al., 2016; Larionov et al., 2021). Nelson et al. introduced this concept for the first time (Carter et al., 2003), but the mode of catalysis remained elusive until Toste et al. realized that the *in situ* replacement of ions solubilizes the cationic reagent in the organic layer and provides a chiral environment for the desired reaction with the substrate (Scheme 1A) (Rauniyar et al., 2011). Toste et al. chose Selectfluor™ as a model reagent to be solubilized and rendered chiral through ion-pairing with chiral phosphate anion catalysts for enantioselective fluorocyclization of enol ethers (Scheme 1B) (Rauniyar et al., 2011). This milestone was followed by various anionic phase-transfer catalysts and their application to different enantioselective transformations (Wang et al., 2012; Phipps and Toste, 2013; Yang et al., 2014; Yamamoto et al., 2016; Avila et al., 2017; Biswas et al., 2018; Liang et al., 2018; Long et al., 2021). Although most of these anionic catalysts show high efficiency in many reactions, their synthetic protocols are quite lengthy and expensive. Hence, novel chiral anionic platforms are highly desirable for opening new horizons for this promising anionic phase-transfer strategy.

Belokon et al. introduced a new series of octahedral anionic salicylimine-based cobaltate (III) complexes that could be readily prepared *via* a facile one-pot protocol (Belokon et al., 1977). The three-dimensional arrangement of two enantiopure tridentate salicylimine-based ligands around the octahedral cobalt center can render the center stereogenic, generating two types of meridional isomers: Λ and Δ *mer* isomers (Ghosh et al., 2017; Cruchter and Larionov, 2018). The metal center itself does not play any role in the catalytic cycle, although it offers a rigid framework to create a chiral environment.

In the last decade, the use of anionic chiral cobalt (III) complexes as phase-transfer catalysts for different asymmetric transformations has received much attention (Gong et al., 2014; Yan et al., 2017; Cruchter and Larionov, 2018; Liu K et al., 2018; Larionov et al., 2021). This can be attributed to the ease of creating a wide range of new structurally diverse chiral metal complexes with distinct features. The positive charge on the cobalt center is compensated for by ligand coordination to generate a lipophilic chiral anion that can be either in the form of a Brønsted acid or salt. Although the separation of these two *mer* isomers *via* column chromatography (Belokon et al., 2004) is essential for their application, the highly stereocontrolled preparation of octahedral cobalt complexes remains challenging (Belokon et al., 2012; Larionov et al., 2015; Larionov et al., 2016; Salem et al., 2021).

Generally, two octahedral anionic salicylimine-based cobaltate (III) complexes can be distinguished: stereogenic*-(only)*-at-metal complexes and stereogenic-at-metal complexes featuring chiral ligands (Cruchter and Larionov, 2018). In the first type, cobalt acts as the exclusive element of chirality; for example, the first complex reported by Belokon et al., in which the salicylimine ligand was derived from a glycine amino acid (Belokon et al., 1977). Alternatively, stereogenic-at-metal complexes featuring chiral ligands represent the main proportion of salicylimine-based cobalt (III) complexes and offer two significant advantages over the first type. First, it is easier to separate and purify the two *mer* isomers compared to other stereogenic*-(only)*-at-metal complexes that require either expensive chiral counter cations or resolution *via* chiral HPLC. Second, this additional element of chirality creates an optimum chiral environment that affords enantioselective transformations. Therefore, only the second type of complex was used as a chiral anionic phase-transfer catalyst.

In 2017, Yu et al. reported the enantioselective bromoaminocyclization of olefins using anionic cobalt (III) complexes as bifunctional phase-transfer catalysts to shuttle the less-soluble brominating reagent to the reaction solution and to control stereoselectivity (Scheme 1C) (Jiang et al., 2017). Similarly, Yu et al. utilized anionic cobalt (III) complexes for the intramolecular and intermolecular halogenation of olefinic bonds (Li et al., 2018; Liu W et al., 2018; Wang et al., 2019; Sun et al., 2021; Wu et al., 2021). However, the enantioselective iodocyclization of enol ethers has been studied less because of the limitations posed by the background reaction.

Thus, in this work, we conduct an anionic octahedral cobalt (III) complex-mediated enantioselective iodocyclization reaction of enol ethers and a diastereoselective synthetic study of anionic octahedral cobalt (III) complexes as bifunctional phase-transfer catalysts (Scheme 1D).

## 2 Results and discussion

### 2.1 Iodocyclization of enol ethers

#### 2.1.1 Catalyst screening

Enol ether derivative **1a** was selected as the model starting material to investigate its iodocyclization reaction, which can afford spiro-fused oxazoline scaffold **2a**, the core structure of many chiral ligands, and biologically active compounds as one of the *N*-heterocycles (Banks et al., 2001; Heckmann and Bach, 2005; Holmes et al., 2005; Yadav et al., 2011; Bhandari Neupane et al., 2019; Bylo et al., 2020; Salem et al., 2020; Connon et al., 2021; Gardouh et al., 2021). The reaction of enol ether derivative **1a** with *N*-iodosuccinimide (NIS) was initially examined in the presence of 10 mol% of the sodium salts of anionic chiral cobalt (III) complexes: Λ-(*S*,*S*)-**3a** and Λ-(*S*,*S*)-**3b** (Table 1). As anticipated, the desired reaction proceeded smoothly to give spiro-fused oxazoline derivative **2a** in high yields but with no enantioselectivity (entries 1–2). Some previous reports have highlighted that metal-templated Brønsted acids exhibit much higher catalytic activity and stereoselectivity than their corresponding salts (Li et al., 2018). Hence, we attempted to check this possibility with Λ-(*S*,*S*)-**3c**; however, there was no improvement in the enantioselectivity (entry 3). Although the opposite *mer* isomers Δ-(*S*,*S*)-**3b** and Δ-(*S*,*S*)-**3c** were also obtained, no improvement in the enantioselectivity was observed (entries 4–5). Similarly, increasing the bulkiness of side chain *R* ^2^ in the Λ-(*S*,*S*)-**3d** complex did not significantly enhance the enantioselectivity (entry 6). Therefore, we reasoned that a significant adjustment to the cobalt (III) complex structure would be crucial for breaking the enantioselectivity ceiling that we bumped into.

**TABLE 1:** Screening of Various Anionic Octahedral Salicylimine-based Cobalt (III) Complexes^a^.

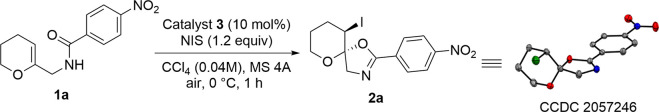				
Entry	Catalyst	Yield (%)^b^	dr^d^	er^d^
1	Λ-(*S*,*S*)-**3a**	91	>20:1	50:50
2	Λ-(*S*,*S*)-**3b**	96	>20:1	51:49
3	Λ-(*S*,*S*)-**3c**	97	>20:1	52:48
4	Δ-(*S*,*S*)-**3b**	93	>20:1	51:49
5	Δ-(*S*,*S*)-**3c**	90	>20:1	50:50
6	Λ-(*S*,*S*)-**3d**	98	>20:1	53:47
7	Λ-(*S*,*R*,*S R*)-**3e**	92	>20:1	65:35
8	Λ-(*S*,*S*,*S,S*)-**3f**	97	>20:1	76:24
9	Δ-(*S*,*S*,*S,S*)-**3f**	87	>20:1	55:45
10	Λ-(*S*,*S*,*S,S*)-**3g**	96 (93)^c^	>20:1	79:21

a The reaction of 1a (0.04 mmol), NIS (0.048 mmol, 1.2 equiv), 3 (0.004 mmol) was conducted in the solvent (1.0 ml).

b Yields were determined *via* ^1^H NMR, spectroscopy using 1,3,5-trimethoxybenzene as an internal standard.

c Isolated yield.

d Determined by HPLC, using (Daicel Chiralpak IBN-5).

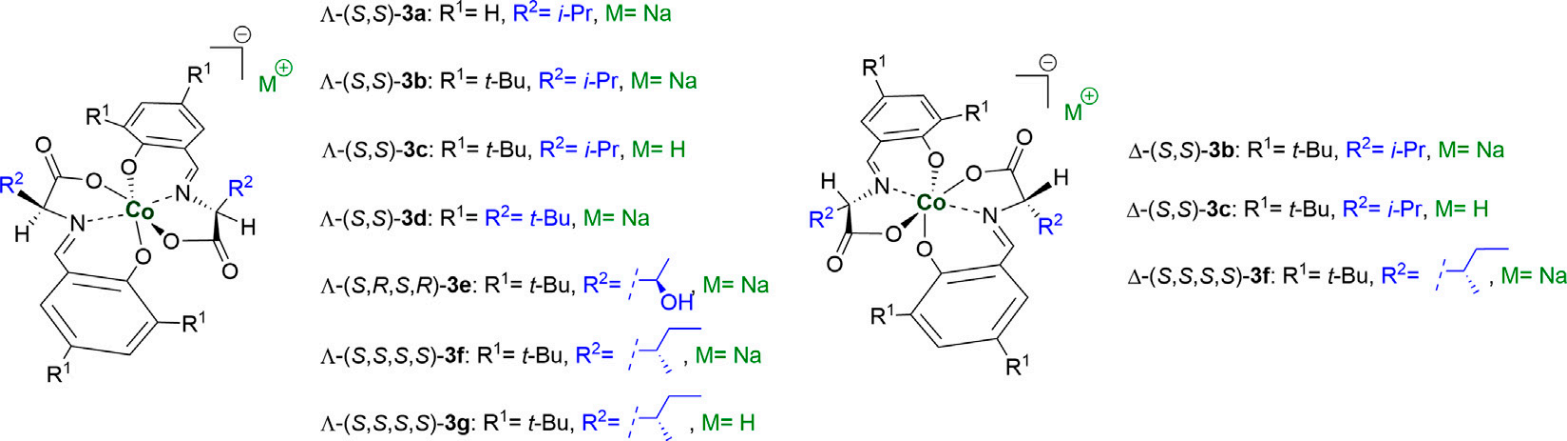

#### 2.1.2 Designing new catalysts

Although asymmetric induction of anionic octahedral cobalt (III) complexes has not been identified, the catalytic cavity lies near the amino acid moiety of salicylimine ligands (Cruchter and Larionov, 2018). The above analysis led us to propose that introducing an additional element of chirality in the coordinating salicylimine ligands can modify the chiral environment and improve the enantioselectivity of this iodocyclization reaction. Accordingly, we used two amino acids with chiral side chains (threonine and isoleucine) to prepare the corresponding anionic cobalt (III) complexes *via* a one-pot protocol. These complexes were then tested in the model reaction under the same conditions, and spiro-fused oxazoline derivative **2a** was isolated in high yields and with substantially improved enantioselectivity (entries 7–8). In particular, Λ-(*S*,*S*,*S*,*S*)-**3f** provided 97% yield and 76:24 er. We also evaluated the opposite *mer* isomer Δ-(*S*,*S*,*S*,*S*)-**3f**, in which the enantiomeric ratio decreased to 55:45 er (entry 9). Generally, the two *mer* isomers (Λ and Δ) do not offer an equivalent degree of enantio-induction owing to the distortion of the chelate rings, which leads to an increase in the distance between the coordinating ligands and alters the chiral environment (Belokon et al., 2004; Belokon et al., 2009; Maleev et al., 2013). Finally, we checked the potential of the corresponding metal-templated Brønsted acid Λ-(*S*,*S*,*S*,*S*)-**3g**, which slightly increased the enantioselectivity to 79:21 er (entry 10). The absolute configuration of **2a** was determined using X-ray crystallography (Table 1). To the best of our knowledge, this is the first report of anionic cobalt (III) complexes showing three elements of chirality (one from the stereogenic center of cobalt and two in each tridentate ligand coming from the chiral centers of amino acids). This additional element of chirality plays a critical role because of its proximity to the catalytic sites of complexes.

#### 2.1.3 Diastereoselective complexation of octahedral cobalt complexes

**FIGURE 1:**
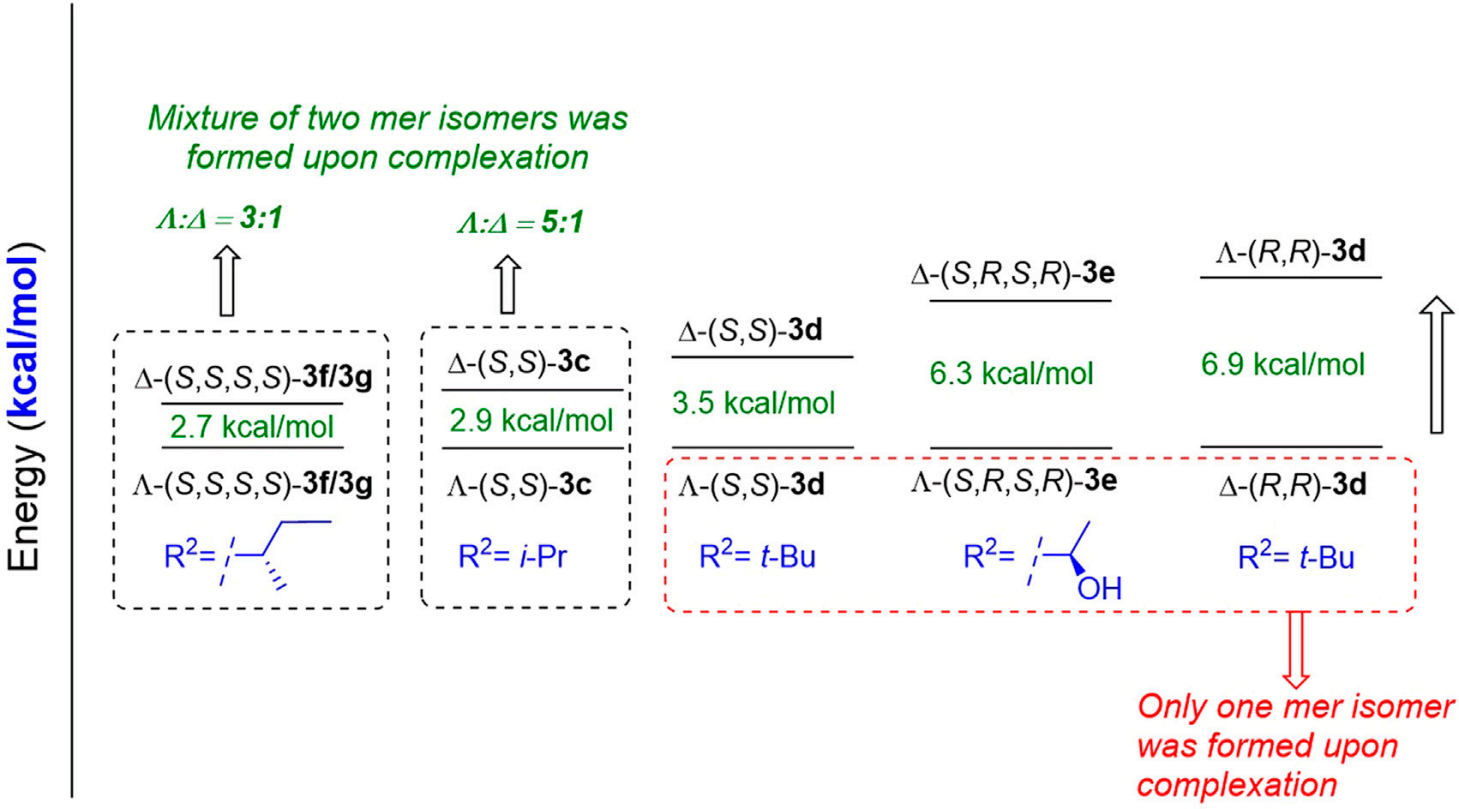
Diastereoselective complexation of octahedral cobalt complexes.

As highlighted previously, the diastereoselective complexation of octahedral salicylimine-based cobalt complexes remains elusive. Although a few tridentate ligands afforded a single *mer* isomer exclusively upon complexation with cobalt, most of the similar ligands afforded a mixture of Λ and Δ *mer* isomers (Belokon et al., 2012; Larionov et al., 2015; Larionov et al., 2016; Salem et al., 2021). Yu et al. conducted a preliminary study on the effects of reaction conditions on the ratio of the two *mer* isomers (Jiang et al., 2017). To understand the reason for these discrepancies, we correlated our experimental observations with a preliminary computational study to further understand this phenomenon. Based on DFT calculations at the B3LYP function using the Wachters–Hay basis set for Co and D95** for other atoms in the Gaussian 16 package, we observed a variation in energy between the two *mer* isomers. Λ-(*S*,*S*)-**3** complexes were more stable than Δ-(*S*,*S*)-**3**, which explains why the former were obtained as the major product in most cases. Some Λ-(*S*,*S*)-**3d** and Λ-(*S*,*R*,*S*,*R*)-**3e** complexes were obtained exclusively as pure Λ isomers because of the high energy gaps between Λ and Δ. Our computational results are also in good agreement with the experimental results obtained (Yu et al., 2015) that demonstrated that Δ-(*R*,*R*)-**3** was obtained exclusively using d-amino acid-based ligands (Figure 1). Finally, our DFT calculations suggested that the bulkiness and configuration of the side chain of the amino acid (*R* ^2^) are key parameters that control the yield and configuration of the major isomer (see ESI for more information).

#### 2.1.4 Optimization of reaction conditions

After determining the optimal catalyst for this reaction, we started to optimize the conditions by screening different parameters, including catalyst loading (Table 2, entries 1–3), solvents (entries 4–11), concentrations (entries 12–15), temperature (entry 16), iodinating agents (entries 17–18), and additives (MS 3A, MS 4A) (entry 19), which showed that the reaction in CCl_4_ afforded a higher enantioselectivity and MS 4A was the best additive to adsorb any moisture and confirm the ultimate dryness. The higher enantioselective induction in the case of CCl_4_ compared to that in other solvents can be attributed to its lower dielectric constant (∼2.2); hence, CCl_4_ molecules do not interpose between the two ions, which is crucial for chiral anion-mediated reactions (Lacour and Moraleda, 2009). Notably, the reaction temperature and concentration exerted little effect on enantioselectivity.

**TABLE 2:** Optimization of reaction conditions^a^.

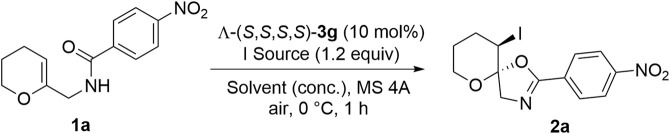				
Entry	[I] source	Solvent	Yield (%)^b^	er^c^
1	NIS	CCl_4_ (0.04 M)	96	79:21
2^d^	NIS	CCl_4_ (0.04 M)	95	73:27
3^e^	NIS	CCl_4_ (0.04 M)	99	75:25
4	NIS	CHCl_3_ (0.04 M)	45	50:50
5	NIS	TFE (0.04 M)	89	51:49
6	NIS	toluene (0.04 M)	91	53:47
7	NIS	CH_2_Cl_2_ (0.04 M)	98	53:47
8	NIS	EtOH (0.04 M)	84	51:49
9	NIS	1,4-dioxane (0.04 M)	93	52:48
10	NIS	C_6_H_5_F (0.04 M)	97	51:49
11	NIS	C_6_H_5_Cl (0.04 M)	99	50:50
12	NIS	CCl_4_ (0.02 M)	92	65:35
13	NIS	CCl_4_ (0.06 M)	95	80:20
14	NIS	CCl_4_ (0.08 M)	97	84:16
15	NIS	CCl_4_ (0.1 M)	98	78:22
16^f^	NIS	CCl_4_ (0.08 M)	95	90:10
17^f^	NIP	CCl_4_ (0.08 M)	89	59:41
18^f^	DIH	CCl_4_ (0.08 M)	99	78:22
19^f^ ^,^ ^g^	NIS	CCl_4_ (0.08 M)	98	87:13

a The reaction of 1a (0.04 mmol), [I] source (0.048 mmol, 1.2 equiv), Λ(*S*,*S*,*S*,*S*)-3 g (0.004 mmol) was conducted in the solvent (1.0 ml).

b Yields were determined *via* ^1^H NMR, spectroscopy using 1,3,5-trimethoxybenzene as an internal standard.

c Determined by HPLC, using (Daicel Chiralpak IBN-5) and dr > 20:1 in all entries.

d Catalyst loading (5 mol%).

e Catalyst loading (20 mol%).

f Temperature = −20°C.

g Using MS, 3A.

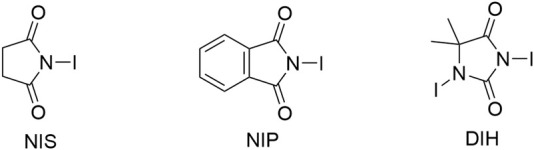

#### 2.1.5 Substrate scope and limitations

**FIGURE 2:**
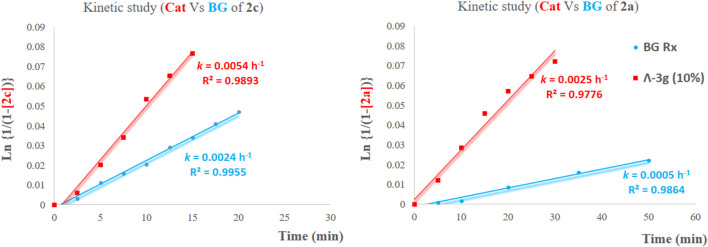
Kinetic studies on the iodocyclization reaction of enol ethers.

With the optimized conditions in hand, we next explored the substrate scope of enantioselective iodocyclization (Table 3). Various enol ether derivatives (**1**) were well tolerated, affording spiro-fused oxazoline derivatives (**2**) in up to 99% yield and 90:10 er. Lower enantioselectivities were observed for halo-substituted spiro-fused oxazoline derivatives **2b**, **2c**, **2g**, **2i**, and **2j**, because of the higher solubility of their corresponding starting materials. Highly soluble substrates are more likely to undergo fast background reactions, which, in turn, affect the enantioselectivity. This phenomenon can be further realized by comparing both the catalytic and background reactions of the two substrates that show a large variance in enantioselectivity, for example, **1a** and **1c**. The calculated rate constants *k* for the formation of **2c** showed that the catalytic pathway proceeded 2.25 times faster than the background pathway (Figure 2A), while *k* for the formation of **2a** was approximately five times faster than that of the background pathway. Hence, we can rationalize the variance in the enantioselectivities of different substrates (Figure 2B).

**TABLE 3:** Substrate scope and limitations^a^.


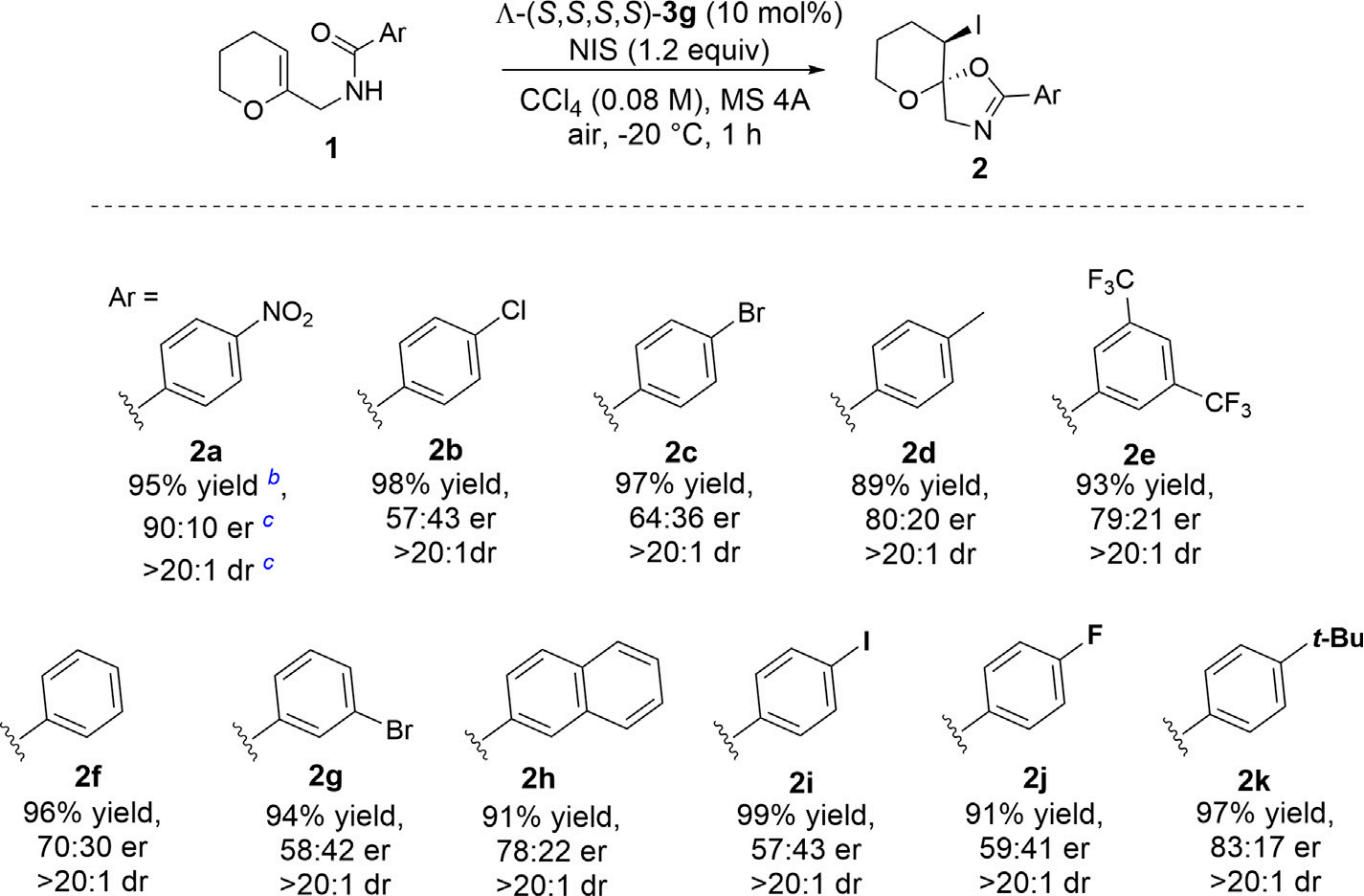

a The reaction of **1** (0.04 mmol), NIS (0.048 mmol, 1.2 equiv), Λ(*S*,*S*,*S*,*S*)-**3g** (0.004 mmol) was conducted in CCl_4_ (1.0 ml).

b Yields were determined *via* ^1^H NMR, spectroscopy using 1,3,5-trimethoxybenzene as an internal standard.

c Determined by HPLC.

#### 2.1.6 Plausible reaction mechanism

**SCHEME 2:**
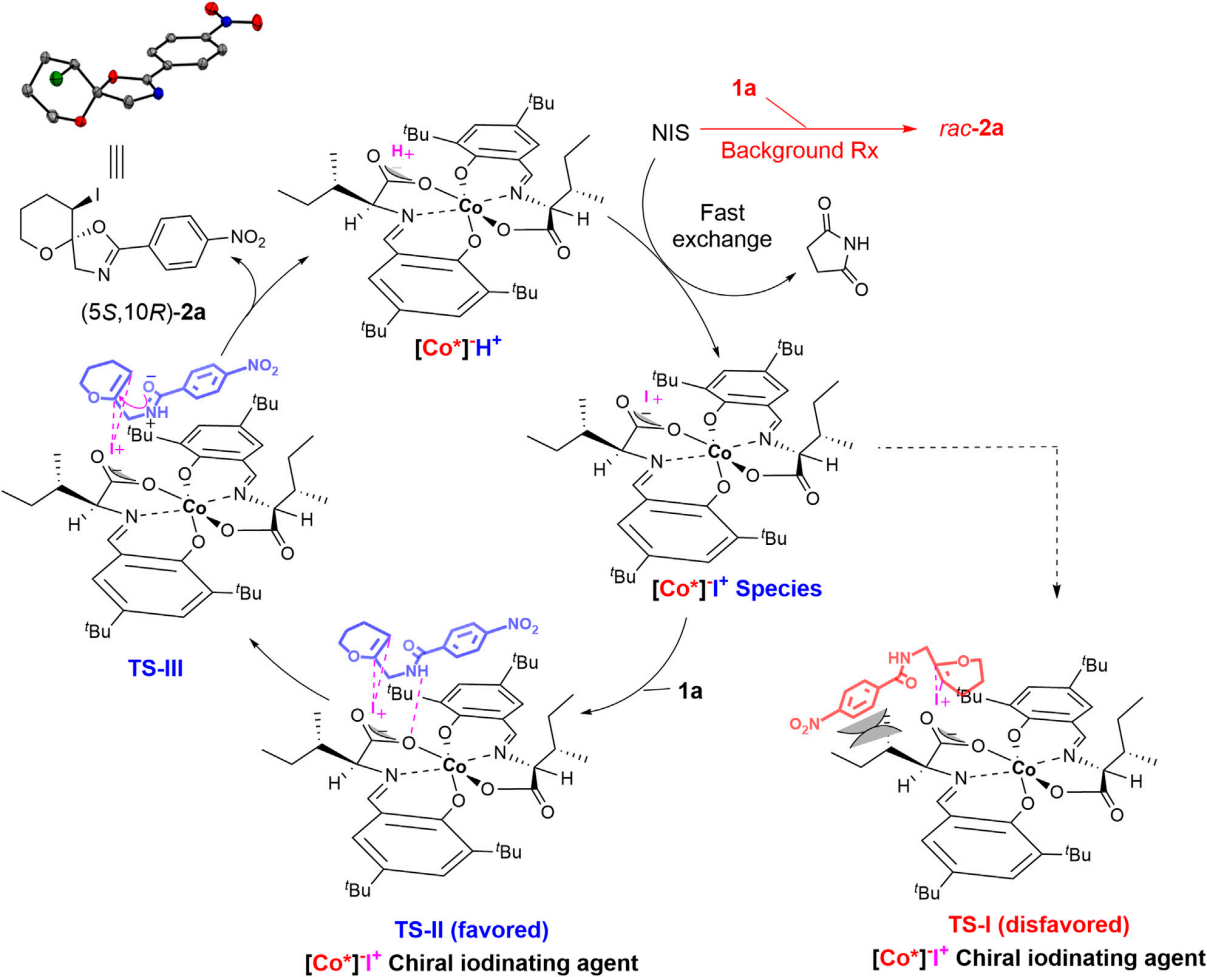
Possible Catalytic Cycle and Transition States.

Based on the kinetic study (Figure 2) and previous reports on halocyclization reactions (Marsden et al., 1994; Jiang et al., 2017; Liu W et al., 2018), a plausible mechanism was proposed (Scheme 2). The Brønsted acid [Co*]^−^H^+^ undergoes a fast exchange with the NIS to generate a lipophilic chiral iodinating agent [Co*]^−^I^+^. The generated chiral [Co*]^−^I^+^ species are highly soluble in CCl_4_ and thereby undergo an asymmetric iodocyclization reaction with enol ether derivative **1** to generate product **2** and regenerate the Brønsted acid [Co*]^−^H^+^ *via* transition states **TS-II** and **TS-III**. As shown in (Scheme 2), iodocyclization could favorably occur on the Re face in **TS-II** *,* as the *Si* face in **TS-I** might be disfavored because of the steric hindrance between the phenyl ring of substrate **1** and the *sec*-butyl side chain of the cobalt complex itself.

### 2.2 Iodoacetalization of enol ethers

To demonstrate the capacity of our new Co(III)-complex Λ-(*S*,*S*,*S*,*S*)-**3g**, featuring chiral ligands with two elements of chirality in each tridentate ligand, we studied another electrophilic iodination reaction of olefins, the iodoacetalization of enol ethers **4** with alcohols **5**, affording 3-iodoacetal derivatives **6**, which have high biological and synthetic values (Ollivier and Renaud, 2001; Haidzinskaya et al., 2015; Martinez et al., 2016). In 2018, Yu et al. conducted a primary study on this reaction using chiral Co(III)-complex-templated Brønsted acids to afford enantioenriched 3-iodoacetals in high yields and up to 83:17 er (Li et al., 2018) using only primary alcohols as substrates. Under our optimal conditions (see ESI, *Funding*), the scope of asymmetric intermolecular iodoacetalization with various alcohols **5** (Table 4) was explored. When primary alcohols such as substituted benzyl or naphthylmethyl were examined, 3-iodoacetal derivatives **6a**–**6i** were isolated in up to 99% yield with 85:15 er. Electron-withdrawing substituents such as nitro groups (**6c** and **6e**) or bromo groups (**6 g–6i**) on the benzyl ring improved the enantioselectivity, and the highest enantiomeric ratio of 85:15 was obtained with 4-NO_2_-substituted benzyl alcohol **6c**. In contrast, electron-donating substituents such as methoxy groups did not have any positive impact on the enantioselectivity of product **6f**. The number of electron-withdrawing substituents (e.g., two nitro groups) or their position did not show any improvement in terms of yield and enantioselectivity for derivatives **6e** and **6 g**–**6i**. Changing enol ether **4** from DHP to DHF afforded 3-iodoacetal derivative **6d** in a good yield (84%); however, the enantiomeric ratio dropped to 66:34. Secondary alcohols also proved to be the optimum nucleophiles to give 3-iodoacetal derivatives **6j**–**6n** in up to 97% yield with 90:10 er. Similarly, electron-withdrawing substituents improved the enantiomeric ratio, as shown by a comparison of derivative **6l** (90:10) with **6j** (83:17). Meanwhile, using *rac.* methyl mandelate provided two easily separable diastereomers **6n** with a high enantiomeric ratio. When we attempted the reaction using both optically pure (*R*) and (*S*)-1-phenylethanol, we obtained moderate enantiomeric ratios for both, with a slight improvement in the case of (*R*)-**6m** (80:20) over (*S*)-**6m** (71:29), because its configuration is more suitable for the catalytic pocket of Λ-(*S*,*S*,*S*,*S*)-**3g**. Screening tertiary alcohols also afforded high yields of 3-iodoacetals **6o**–**6s** with a high enantiomeric ratio of 92:8 er. Many functional groups were found to be compatible with our reaction system, including nitro groups (**6c**–**6e**), bromides (**6g**–**6i**), chlorides (**6l**), esters (**6n**), and dienones (**6q**–**6s**).

**TABLE 4:** Iodoacetalization of enol ethers.


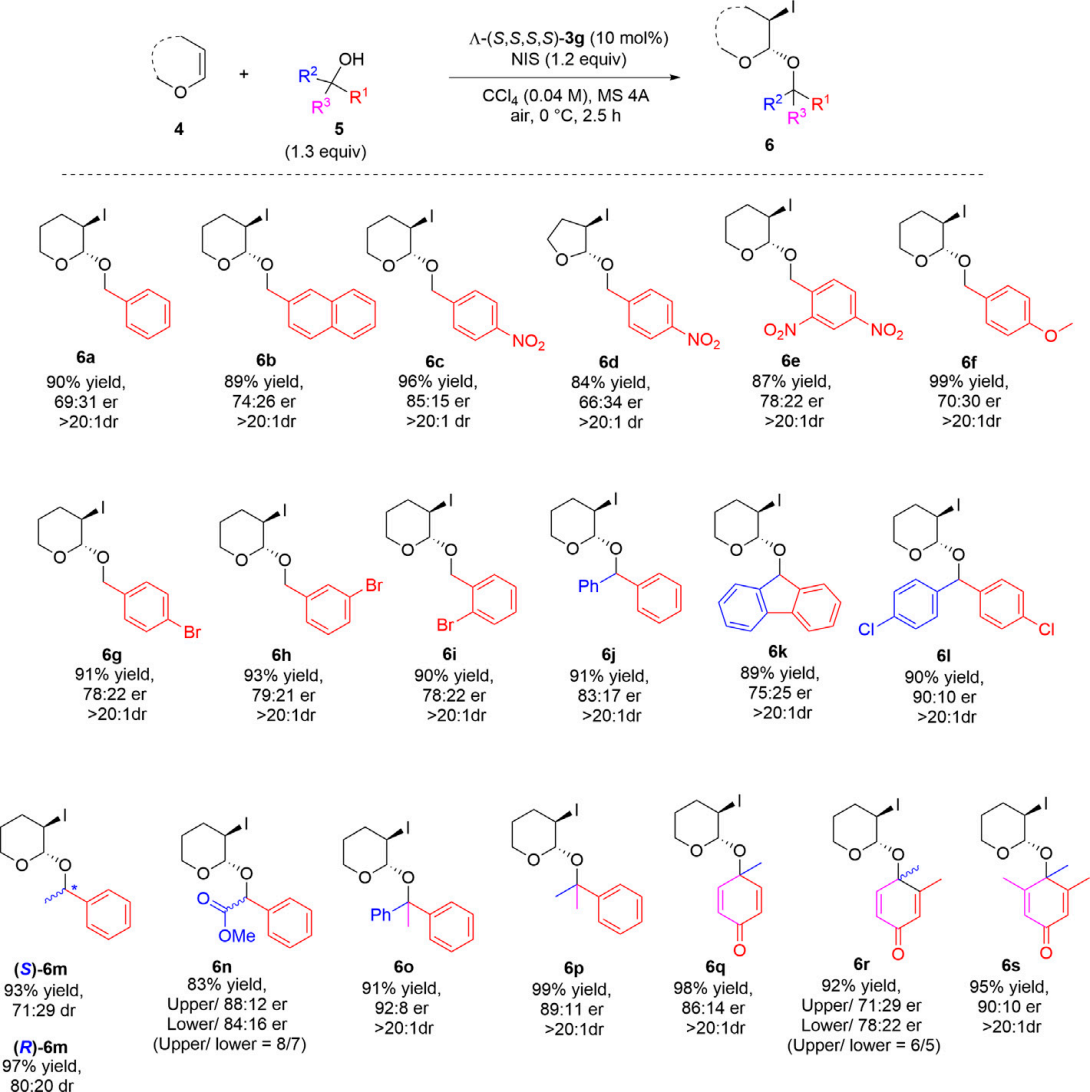

## 3 Conclusion

We introduced new anionic salicylimine-based cobalt (III) complexes with an additional element of chirality, which improved their catalytic activities toward the electrophilic iodination reactions of enol ethers. We attempted to rationalize the reasons for the discrepancies between the ratios of the *mer* isomers during the preparation of analogous octahedral cobalt (III) complexes. In addition, two iodination reactions of enol ethers have been studied. Iodocyclization of enol ethers **1** furnished valuable spiro-fused oxazoline derivatives **2** in high yields and good enantioselectivities up to 90:10 er. A plausible mechanism of this reaction was proposed. Asymmetric iodoacetalization of enol ethers **4** was also promoted to afford various 3-iodoacetals **6**, derived from primary, secondary, and tertiary alcohols, in high yields and enantiomeric ratios of up to 92:8 er. More applications of these new anionic salicylimine-based cobalt (III) complexes **3** are currently under investigation.

## Data Availability

Additional data supporting the findings described in this manuscript are available in the Supplementary Material. The X-ray crystallographic coordinate for structures reported in this study has been deposited at the Cambridge Crystallographic Data Centre (CCDC) under deposition number CCDC-2057246 (**2a**). These data can be obtained free of charge from The Cambridge Crystallographic Data Centre *via* www.ccdc.cam.ac.uk/data_request/cif. The authors declare that all other data supporting the findings of this study are available within the article and Supplementary Material and are available from the corresponding authors upon reasonable request.
