# Supplementary material for: New anionic cobalt(III) complexes enable enantioselective synthesis of spiro-fused oxazoline and iodoacetal derivatives
Source: Front Chem. 2022 Oct 13;10:1034291. doi: 10.3389/fchem.2022.1034291 (PMC9606352; doi:10.3389/fchem.2022.1034291)

Supporting Information

New Anionic Cobalt(III) Complexes Enable Enantioselective Synthesis of Spiro-fused Oxazoline and Iodoacetal Derivatives

Mohamed S. H. Salem,*[1,2] Shinobu Takizawa*[1]

[1] SANKEN, Osaka University, Mihogaoka, Ibaraki-shi, Osaka 567-0047, Japan

[2] Pharmaceutical Organic Chemistry Department, Faculty of Pharmacy, Suez Canal University, Ismailia 41522, Egypt

E-mail: taki@sanken.osaka-u.ac.jp, mohamedsalem43@sanken.osaka-u.ac.jp

Tel: +81-6-6879-8467; Fax: +81-6-6879-8469

Table of Contents

1.	General information	S2
2.	Materials	S2
3.	Experimental Procedures	S2
	3.1. Preparation of cobalt complexes	S2
	3.2. Preparation of starting materials **1a-1k**	S4
	3.3. Preparation of spiro-fused oxazoline derivatives **2a-2k**	S5
	3.4. Preparation of 3-iodoacetal derivatives **6a-6s**	S6
4.	Characterization Data	S7
5.	Optimization of iodocyclization reaction conditions	S25
6.	Optimization of iodoacetalization reaction conditions	S27
7.	Extended substrate scope of iodoacetalization reaction	S29
8.	Unsuccessful cobalt(III)-catalyzed enantioselective transformations	S30
9.	Suboptimal cobalt(III)-catalyzed enantioselective transformations	S31
10.	DFT calculations	S32
11.	Kinetic study and data analysis	S49
12.	Stability of halocyclization products	S50
13.	CD spectra	S52
14.	References	S52
15.	NMR spectra	S53
16.	HPLC charts	S97
17.	X-ray Crystallographic Analysis	S129

1. General information

^{1}H-, and ^{13}C-NMR spectra were recorded with JEOL JMN ECS400 FT NMR, JNM ECA600 FT NMR or Bruker AVANCE II (^{1}H-NMR 400, or 600 MHz, ^{13}C-NMR 100, or 150 MHz) ^{1}H-NMR spectra are reported as follows: the chemical shift in ppm downfield of tetramethylsilane (TMS) and referenced to residual solvent peak ($CDCl_3$) at 7.26 ppm, (CD_3OD) at 3.31 ppm or ($(CD_3)_2CO$) at 2.05 ppm, integration, multiplicities (s = singlet, d = doublet, t = triplet, q = quartet, m = multiplet, dd = doublet of doublets, dt = doublet of triplets), and coupling constants (Hz). ^{13}C-NMR spectra were reported in ppm relative to the central line of triplet for $CDCl_3$ at 77.16 ppm, the central line of (CD_3OD) at 49.03 ppm, or the central line of septet for ($(CD_3)_2CO$) at 29.84 ppm. ESI-MS spectra were obtained with JMS-T100LC (JEOL). Optical rotations were measured with JASCO P-1030 polarimeter. HPLC analyses were performed on JASCO HPLC system (JASCO PU 980 pump and UV-975 UV/Vis detector) using a mixture of *n*-hexane and HPLC grade *i*-PrOH as eluents. Chiralpak IB, IBN-5, IC, IC-3, OJ-H, and IF-3 columns were purchased from Daicel Chemical Industries, Ltd. FT-IR spectra were recorded on JASCO FT-IR system (FT/IR4100). Thin-layer chromatography (TLC) analysis of reaction mixtures was performed using Merck silica-gel 60 F254 TLC plates and visualized under UV Column chromatography on SiO_2 was performed with Kanto silica-gel 60 (63-210 μm).

2. Materials

Analytic grade solvents for the column chromatography and commercially available reagents were used as received. CCl_4 was dried over molecular sieves MS 4A and distilled prior to use. All alcohols **5** and acid chlorides were purchased or prepared according to previous literature.

3. Experimental procedures

3.1. Preparation of cobalt complexes

3.1.1. General procedures for preparation of Λ and Δ octahedral cobalt(III) complexes

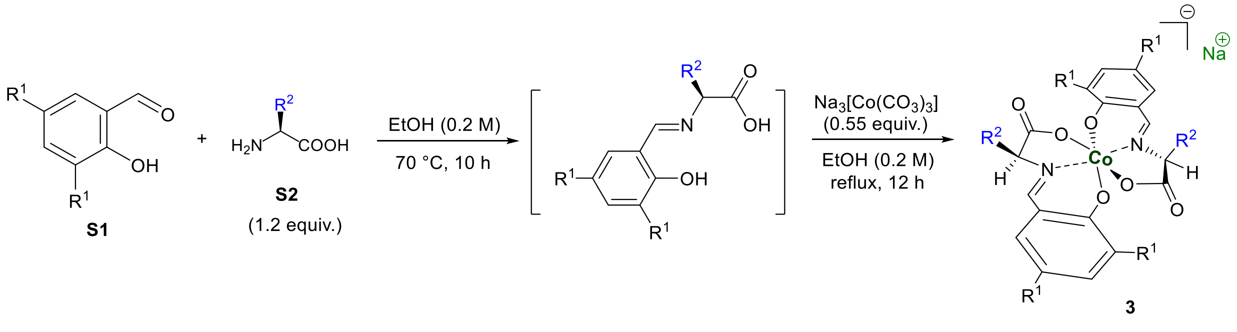

In a 50 mL round-bottom flask was placed the salicylaldehyde derivative (5 mmol), *L*-aminoacid (5 mmol) in EtOH (25 mL). The reaction mixture became homogeneous by heating at 70 ºC for 10 h and then freshly prepared $Na_3[Co(CO_3)_3]$ was added. The resultant mixture was refluxed 12 h, and then filtered after cooling to room temperature. The filtrate was concentrated *in vacuo*, and the residue was purified by column chromatography on neutral Al_2O_3 (EtOH as eluent, the Λ-(*S*,*S*)-complexes were always observed to have higher R_f values than the Δ-(*S*,*S*)-complexes), followed by silica gel column (eluent; MeOH/DCM = 1/10). Cobalt(III) complexes Λ-(*S*,*S*)-**3a** (Belokon et al., 2008), Λ-(*S*,*S*)-**3b**, Δ-(*S*,*S*)-**3b** (Jiang et al., 2017) and Λ-(*S*,*S*)-**3d** (Yu et al., 2015) have been reported previously in the literature.

3.1.2. General procedures for preparation of sodium tricarbonatocobaltate(III) Na3[Co(CO3)3]

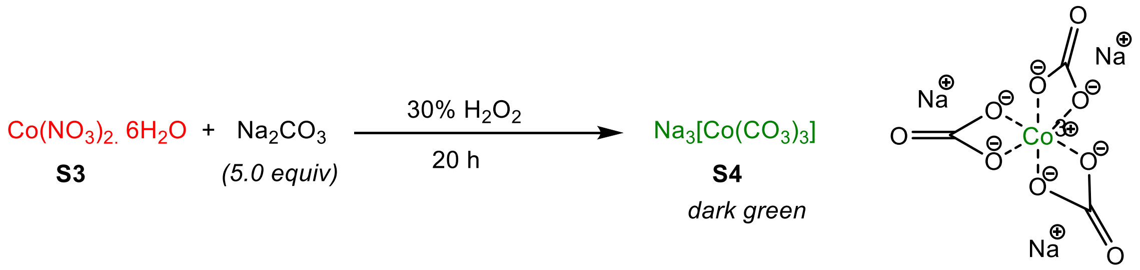

Sodium Tricarbonatocobaltate(III) $Na_3[Co(CO_3)_3]$ was prepared according to (Belokon et al., 2009). A solution of cobalt(II) nitrate hexahydrate $Co(NO_3)_2.6H_2O$ (25 mmol) in H_2O (12.5 mL) and 30% H_2O_2 (5 mL) was added dropwise to a susp. of sodium carbonate Na_2CO_3 (5.0 equiv.) in H_2O (12.5 mL) while cooling to 0 °C. The reaction mixture was stirred for 20 h, at room temperature. The dark-green precipitate that formed was filtered off and washed with H_2O and EtOH to give t sodium tricarbonatocobaltate as a dark green solid.

3.1.3. General procedures for preparation of Brønsted acids of cobalt(III) complexes

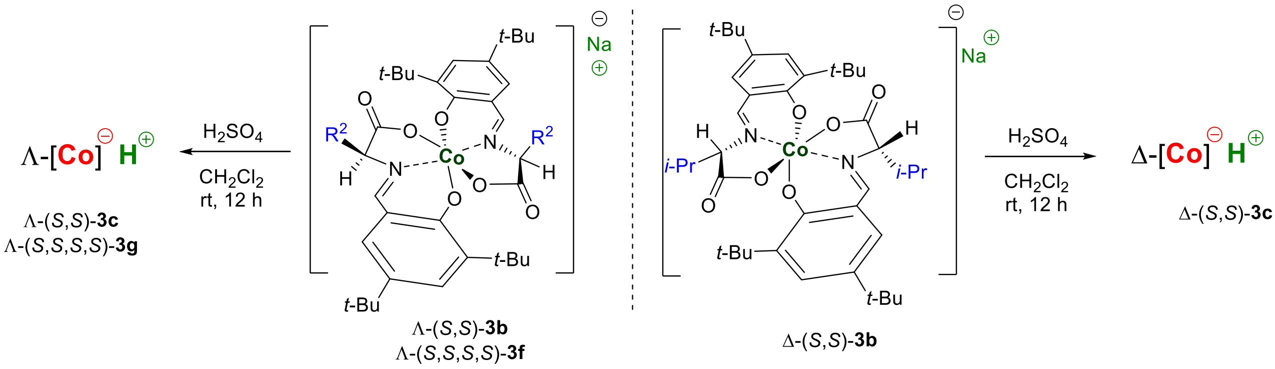

Brønsted acids of cobalt(III) complexes were prepared from the corresponding sodium salts according to (Jiang et al., 2017). In a 50 mL round-bottom flask, conc. H_2SO_4 (0.55 mmol) was added to the sodium salt of cobalt(III) complex (0.5 mmol) in CH_2Cl_2 (25 mL). The resulting solution was allowed to warm to room temperature and stirred vigorously overnight. Then, the mixture was filtered and the filtrate was washed (liquid extraction) with 3N HCl (20 mL × 2 times), and H_2O (20 mL × 2 times). The organic layers were collected, dried over Na_2SO_4, and concentrated *in vacuo* to afford the corresponding Brønsted acids of cobalt(III) complexes. Cobalt(III) complexes Λ-(*S*,*S*)-**3c**, and Δ-(*S*,*S*)-**3c** have been reported previously in the literature (Jiang et al., 2017).

3.2. Preparation of starting materials for iodocyclization reaction 1a-1k

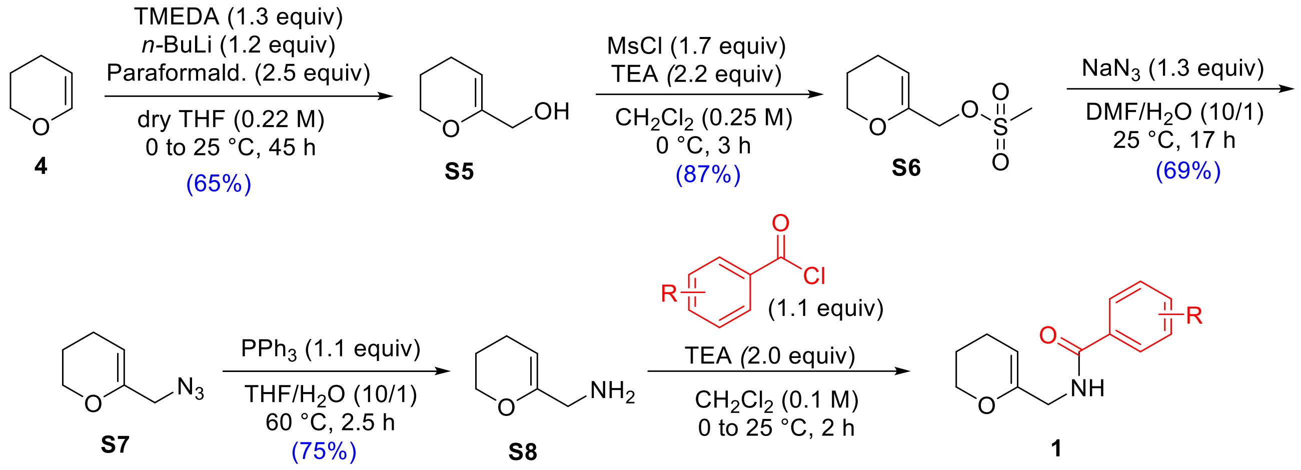

Starting materials **S5**-**S8** were synthesized according to the above scheme and following the previous report of (Rauniyar et al., 2011).

3.2.1. General procedures for preparation of compound ***1a-1k***

To a homogenous solution of methanmine **S8** (5.0 mmol) in CH_2Cl_2 (50 mL) at 0 °C, TEA (2.0 equiv) was added dropwise under nitrogen, and then stirred for 15 minutes. The appropriate acid chloride was added (1.1 equiv), and the mixture was allowed to warm to room temperature and stirred for 2 h. After completion of the reaction (monitored by TLC), the mixture was extracted with CH_2Cl_2, washed with brine (30 mL × 2 times), dried over Na_2SO_4, and concentrated *in vacuo*. The residue was purified by a TEA-treated silica gel column (eluent: EtOAC/*n*-hexane = 1/4, with 1% TEA).

3.3. Preparation of spiro-fused oxazoline derivatives 2a-2k

*3.3.1. General procedures for preparation of racemic spiro-fused oxazoline derivatives **2a-2k***

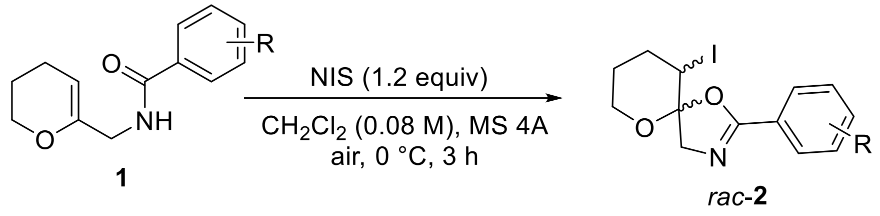

All authentic racemic samples were prepared according to the following procedure: A 15-mL oven-dried reaction vessel was charged with substrates **1a-1k** (0.20 mmol), activated MS 4A (100 mg), and CH_2Cl_2 (2.5 mL) at room temperature. NIS (0.24 mmol) was added and the resulting solution was stirred at 0 °C under air atmosphere in absence of light for 3 h. The reaction was then quenched with sat. aqueous soln. of $Na_2S_2O_3$ (0.1 mL). The mixture was purified by thin layer chromatography (silica gel, *n*-hexane/EtOAc = 10:1) to give the racemic spiro-fused oxazoline derivatives **2a-2k**.

*3.3.2. Preparation of enantioenriched spiro-fused oxazoline derivatives **2a-2k***

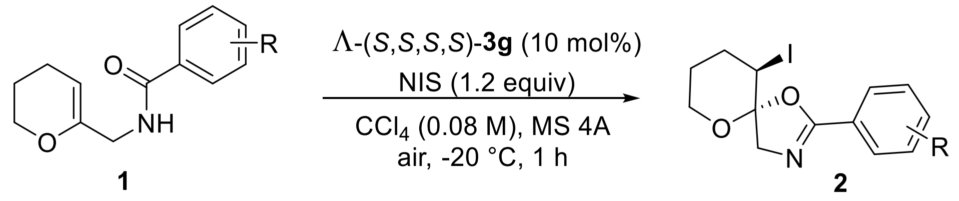

At room temperature, a 15-mL oven-dried reaction vessel was charged with catalyst Λ-(*S*,*S*,*S*,*S*)-**3g** (7.5 mg, 0.01 mmol), activated MS 4A (50 mg), substrate **1** (0.10 mmol), and CCl_4 (1.25 mL). The vessel was protected from light *via* aluminum foil, cooled to -20 ºC, and stirred for 15 min. NIS (27 mg, 0.12 mmol) was added to the resulting solution and stirred vigorously until completion of the reaction (monitored by TLC, around 1 h). The reaction was then quenched with sat. aqueous soln. of $Na_2S_2O_3$ (0.1 mL). The mixture was extracted with DCM, washed with water, dried over Na_2SO_4, and concentrated *in vacuo*. Using silica gel column chromatography (*n*-hexane/EtOAc = 10:1) the enantioenriched spiro-fused oxazoline derivatives **2a-2k** were afforded as pure white solids in high yields and kept at a low temperature (< -20 °C) to avoid their fast decomposition. The absolute configuration of **2a** was determined by X-ray crystallographic analysis.

3.4. Preparation of 3-iodoacetal derivatives 6a-6s

*3.4.1. General procedures for preparation of racemic 3-iodoacetal derivatives **6a-6s***

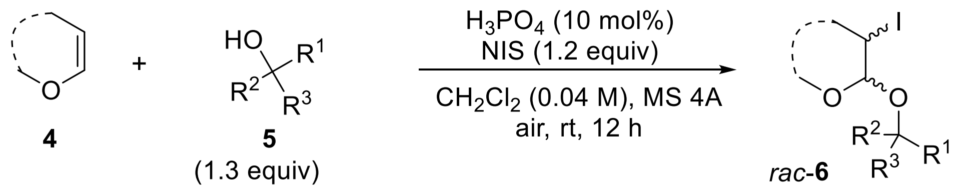

All authentic racemic samples were prepared according to the reported procedures (Li et al., 2018): A 15-mL oven-dried reaction vessel was charged with enol ethers **4** (0.20 mmol), alcohols **5** (0.26 mmol), orthophosphoric acid (0.02 mmol), activated MS 4A (100 mg) and CH_2Cl_2 (5.0 mL) at room temperature. NIS (0.24 mmol) was then added and the resulting solution was stirred vigorously under an air atmosphere within 12 hours in absence of light. The reaction was then quenched with sat. aqueous soln. of $Na_2S_2O_3$ (0.1 mL). The mixture was purified by thin layer chromatography (silica gel, *n*-hexane /EtOAc = 8:1) to give the racemic 3-iodoacetal derivatives **6a-6s**.

*3.4.2. Preparation of enantioenriched 3-iodoacetal derivatives **6a-6s***

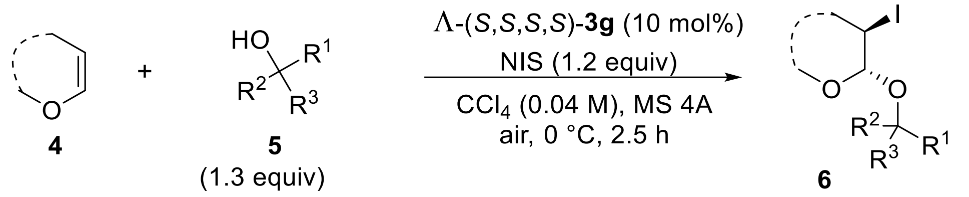

At room temperature in the absence of light, a 15-mL oven-dried reaction vessel was charged with catalyst Λ-(*S*,*S*,*S*,*S*)-**3g** (7.5 mg, 0.01 mmol), NIS (27 mg, 0.12 mmol), activated MS 4A (50 mg), alcohol **5** (0.13 mmol), and dry CCl_4 (2.5 mL, 0.04 M). The mixture was cooled to 0 °C and stirred for 15 min. Then, enol ether **4** (9.125 μL, 0.1 mmol) was added and the resulting solution was stirred vigorously under an air atmosphere for 2.5 h. The reaction was then quenched with TEA (70 μL, 0.5 mmol) and sat. aqueous soln. of $Na_2S_2O_3$ (0.1 mL). The mixture was purified by thin layer chromatography (silica gel, *n*-hexane /EtOAc = 8:1) to give the enantioenriched 3-iodoacetal derivatives **6a-6s**. The absolute configuration of **6c** was assigned based on the previous report of Yu and coworkers (Li et al., 2018).

4. Characterization data

Sodium Λ-bis[*N*-(3,5-di-*tert*-butyl-salicylidene)-(*S*,*R*)-threonato] cobaltate Λ-(*S*,*R*,*S*,*R*)-3e:

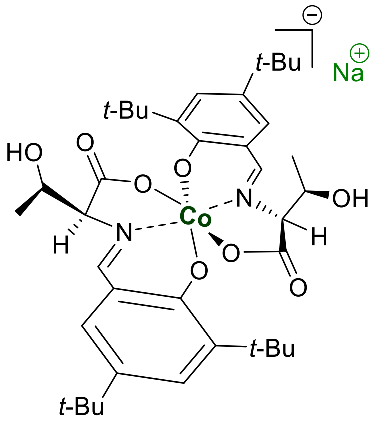

Λ-(*S*,*R*,*S*,*R*)-3e

obtained as a single *mer* isomer (Λ) only in 77% yield (dark red color); mp> 300 °C; **[α]D[20]** = -3513.7 (c 0.09 $CHCl_3$); ^{1}**H-NMR** (600 MHz, CD_3OD) *δ* 8.41 (s, 2H), 7.17 (d, *J* = 2.1 Hz, 2H), 7.04 (d, *J* = 2.1 Hz, 2H), 4.65 (d, *J* = 6.2 Hz, 2H), 4.30 (t, *J* = 5.5 Hz, 2H), 1.47 (d, *J* = 6.2 Hz, 6H), 1.23 (s, 18H), 0.97 (s, 18H); ^{13}**C-NMR** (150 MHz, CD_3OD) *δ* 183.71, 168.79, 163.13, 143.23, 136.60, 129.34, 129.28, 119.94, 79.21, 69.86, 36.07, 34.54, 31.87, 30.09, 20.42; **HRMS** (ESI) calculated for $C_{38}H_{54}CoN_2O_8$ $[M\text{-}Na]^-$: 725.3212, found 725.3201; **IR** (KBr): 3389, 2956, 2906, 2870, 1634, 1614, 1438, 1255, 1170, 780 cm^{-1}.

Sodium Λ-bis[*N*-(3,5-di-*tert*-butyl-salicylidene)-(*S*,*S*)-isoleucinato] cobaltate Λ-(*S*,*S*,*S*,*S*)-3f:

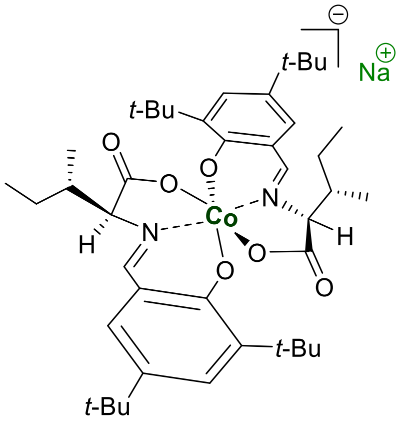

Λ-(*S*,*S*,*S*,*S*)-3f

obtained as a mixture of two *mer* isomer (Λ/Δ = 3/1) in 62% total yield (dark red color); the configuration of the major *mer* isomer (Λ) was assigned by the comparison with the known absolute configuration of previously reported Λ-(*S*,*S*)-**3d** (see section 12: CD spectra); mp> 300 °C; **[α]D[15]** = -2409.5 (c 0.053 $CHCl_3$); ^{1}**H-NMR** (600 MHz, CD_3OD) *δ* 8.43 (s, 2H), 7.18 (s, 2H), 7.04 (s, 2H), 4.68 (d, *J* = 3.4 Hz, 2H), 2.47-2.52 (m, 2H), 1.94-1.98 (m, 2H), 1.64-1.69 (m, 2H), 1.25 (s, 6H), 1.24 (s, 18H), 1.11 (t, *J* = 5.2 Hz, 6H), 1.00 (s, 18H); ^{13}**C-NMR** (150 MHz, CD_3OD) *δ* 185.21, 166.94, 163.14, 142.84, 136.23, 129.24, 129.05, 119.65, 76.84, 70.61, 42.21, 36.09, 34.60, 31.97, 30.04, 29.55, 27.98; **HRMS** (ESI) calculated for $C_{42}H_{62}CoN_2O_6$ $[M\text{-}Na]^-$: 749.3940, found 749.3918; **IR** (KBr): 3373, 2960, 2907, 2873, 1642, 1607, 1439, 1256, 1169, 780 cm^{-1}.

Sodium Δ-bis[*N*-(3,5-di-*tert*-butyl-salicylidene)-(*S*,*S*)-isoleucinato] cobaltate Δ-(*S*,*S*,*S*,*S*)-3f: obtained as a mixture of two *mer* isomer (Λ/Δ = 3/1) in 62% total yield (dark red color); mp> 300 °C; **[α]$_D^{18}$** = +1454.8 (c 0.06 $CHCl_3$); ^{1}**H-NMR** (600 MHz, CD_3OD) δ 8.34 (s, 2H), 7.20 (s, 2H), 7.04 (d, J = 2.1 Hz, 2H), 4.49 (s, 2H), 2.83-2.87 (m, 2H), 1.95-1.99 (m, 2H), 1.81-1.84 (m, 2H), 1.25 (s, 18H), 1.21-1.24 (m, 6H), 1.12 (t, J = 6.2 Hz, 6H), 0.90 (s, 18H); ^{13}**C-NMR** (150 MHz, CD_3OD) δ 183.99, 166.33, 163.95, 143.66, 136.88, 129.53, 121.92, 75.20, 70.61, 38.21, 36.18, 34.64, 31.94, 30.25, 29.55, 27.68 (One carbon overlapped); **HRMS** (ESI) calculated for $C_{42}H_{62}CoN_2O_6$ $[M-Na]^-$: 749.3940, found 749.3926; **IR** (KBr): 3371, 2960, 2906, 2873, 1644, 1608, 1436, 1255, 1168, 780 cm^{-1}.

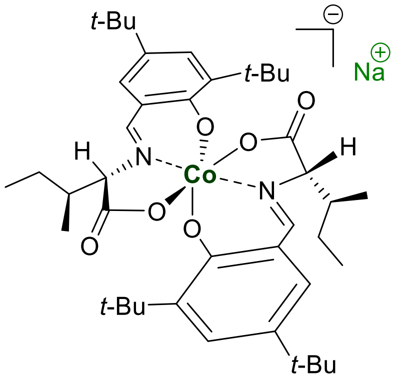

Δ-(*S*,*S*,*S*,*S*)-3f

Λ-Bis[*N*-(3,5-di-*tert*-butyl-salicylidene)-(*S*,*S*)-isoleucinato] cobaltic acid Λ-(*S*,*S*,*S*,*S*)-3g: obtained in 93% yield; mp> 300 °C; **[α]$_D^{15}$** = -1986.3 (c 0.051 $CHCl_3$); ^{1}**H-NMR** (600 MHz, $(CD_3)_2CO$) δ 8.54 (s, 2H), 7.24 (d, J = 2.7 Hz, 2H), 7.04 (d, J = 2.1 Hz, 2H), 4.61 (d, J = 2.7 Hz, 2H), 2.43-2.48 (m, 2H), 1.90-1.97 (m, 2H), 1.61-1.68 (m, 2H), 1.24 (s, 18H), 1.04 (t, J = 7.2 Hz, 6H), 0.97-0.99 (m, 24H); ^{13}**C-NMR** (150 MHz, CD_3OD) δ 185.12, 167.04, 163.20, 142.86, 136.13, 129.21, 128.87, 119.70, 76.74, 70.58, 42.28, 35.98, 34.57, 32.04, 30.12, 29.41, 28.07; **HRMS** (ESI) calculated for $C_{42}H_{62}CoN_2O_6$ $[M-H]^-$: 749.3940, found 749.3914; **IR** (KBr): 3375, 2961, 2907, 2874, 1643, 1605, 1440, 1257, 1169, 781 cm^{-1}.

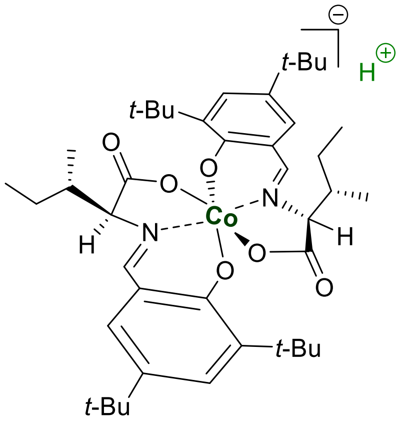

Λ-(*S*,*S*,*S*,*S*)-3g

***N*-((3,4-Dihydro-2*H*-pyran-6-yl)methyl)-4-nitrobenzamide 1a**: yield: 63%; (Silica gel column chromatography eluent, *n*-hexane/ethyl acetate = 4/1 with 1% TEA); white solid; ^{1}**H-NMR** (400 MHz, $CDCl_3$) δ 8.29 (d, J = 8.7 Hz, 2H), 7.95 (d, J = 8.7 Hz, 2H), 6.41 (s, 1H), 4.82 (t, J = 3.7 Hz, 1H), 4.01-4.06 (m, 4H), 2.03-2.07 (m, 2H), 1.81-1.86 (m, 2H); ^{13}**C-NMR** (100 MHz, $CDCl_3$) δ 165.29, 149.69, 149.49, 140.25, 128.34, 123.94, 98.89, 66.67, 43.35, 22.25, 20.08; **HRMS** (ESI) calculated for $C_{13}H_{14}N_2NaO_4$ $[M+Na]^+$: 285.0846, found 285.0847; **IR** (KBr): 3317, 3078, 2929, 2845, 1639, 1524, 1349, 1239, 1055, 871

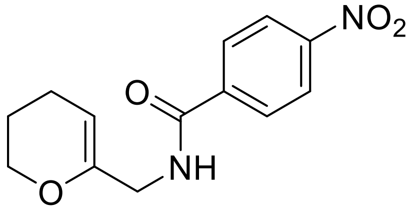

1a

cm^{-1}.

4-Chloro-*N*-((3,4-dihydro-2*H*-pyran-6-yl)methyl)benzamide 1b: yield: 56%; (Silica gel column chromatography eluent, *n*-hexane/ethyl acetate = 5/1 with 1% TEA); white solid; ^{1}**H-NMR** (400 MHz, $CDCl_3$) δ 7.72 (d, J = 8.7 Hz, 2H), 7.40 (d, J = 8.7 Hz, 2H), 6.33 (s, 1H), 4.79 (t, J = 3.7 Hz, 1H), 3.98-4.04 (m, 4H), 2.01-2.05 (m, 2H), 1.79-1.85 (m, 2H); ^{13}**C-NMR** (100 MHz, $CDCl_3$) δ 166.25, 149.94, 137.78, 133.06, 128.92, 128.57, 98.43, 66.62, 43.09, 22.31, 20.10; **HRMS** (ESI) calculated for $C_{13}H_{14}ClNNaO_2$ $[M+Na]^+$: 274.0605, found 274.0604; **IR** (KBr): 3281, 2969, 2925, 2852, 1632, 1547, 1489, 1237, 1064, 850 cm^{-1}.

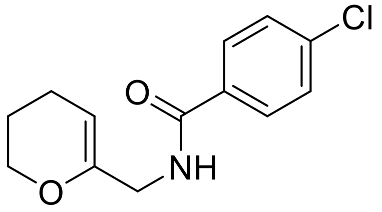

1b

4-Bromo-*N*-((3,4-dihydro-2*H*-pyran-6-yl)methyl)benzamide 1c: yield: 47%; (Silica gel column chromatography eluent, *n*-hexane/ethyl acetate = 6/1 with 1% TEA); white solid; ^{1}**H-NMR** (400 MHz, $CDCl_3$) δ 7.65 (d, J = 8.7 Hz, 2H), 7.56 (d, J = 8.2 Hz, 2H), 6.33 (s, 1H), 4.79 (t, J = 3.4 Hz, 1H), 4.03 (t, J = 5.3 Hz, 2H), 3.98 (d, J = 5.5 Hz, 2H), 2.01-2.05 (m, 2H), 1.79-1.85 (m, 2H); ^{13}**C-NMR** (100 MHz, $CDCl_3$) δ 166.35, 149.91, 133.52, 131.90, 128.75, 126.22, 98.45, 66.62, 43.10, 22.31, 20.10; **HRMS** (ESI) calculated for $C_{13}H_{14}BrNNaO_2$ $[M+Na]^+$: 318.0100, found 318.0099; **IR** (KBr): 3294, 3086, 2928, 2842, 1632, 1548, 1312, 1239, 1064, 849 cm^{-1}.

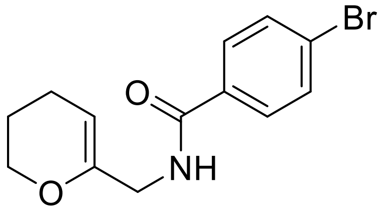

1c

***N*-((3,4-Dihydro-2*H*-pyran-6-yl)methyl)-4-methylbenzamide 1d**: yield: 65%; (Silica gel column chromatography eluent, *n*-hexane/ethyl acetate = 6/1 with 1% TEA); white solid; ^{1}**H-NMR** (400 MHz, $CDCl_3$) δ 7.68 (d, J = 8.2 Hz, 2H), 7.22 (d, J = 7.8 Hz, 2H), 6.34 (s, 1H), 4.78 (t, J = 3.7 Hz, 1H), 4.02 (t, J = 5.0 Hz, 2H), 3.98 (d, J = 5.5 Hz, 2H), 2.39 (s, 3H), 2.00-2.05 (m, 2H), 1.78-1.84 (m, 2H); ^{13}**C-NMR** (100 MHz, $CDCl_3$) δ 167.26, 150.26, 141.93, 131.84, 129.30, 127.09, 98.05, 66.57, 42.89, 22.34, 21.58, 20.10; **HRMS** (ESI) calculated for $C_{14}H_{17}NNaO_2$ $[M+Na]^+$: 254.1152, found 254.1145; **IR** (KBr): 3325, 2961, 2920, 2846, 1632, 1553, 1310, 1254, 1065, 759 cm^{-1}.

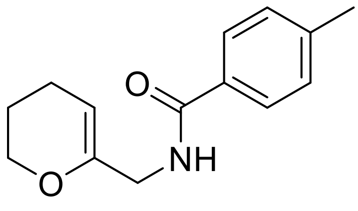

1d

***N*-((3,4-Dihydro-2*H*-pyran-6-yl)methyl)-3,5-bis(trifluoromethyl)benzamide 1e**: yield: 74%;

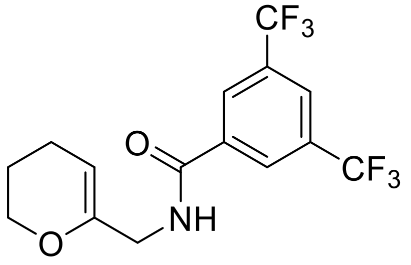

1e

(Silica gel column chromatography eluent, *n*-hexane/ethyl acetate = 7/1 with 2% TEA); white solid; ^{1}**H-NMR** (400 MHz, $CDCl_3$) δ 8.22 (s, 2H), 7.99 (s, 1H), 6.56 (s, 1H), 4.82 (t, J = 3.2 Hz, 1H), 4.02-4.06 (m, 4H), 2.02-2.07 (m, 2H), 1.80-1.86 (m, 2H); ^{13}**C-NMR** (100 MHz, $CDCl_3$) δ 164.47, 149.44, 136.76, 132.29 (d, J_{C-F} = 34.5 Hz), 127.50, 125.10, 123.06 (d, J_{C-F} = 273.2 Hz), 99.00, 66.67, 43.44, 22.23, 20.09; **HRMS** (ESI) calculated for $C_{15}H_{13}F_6NNaO_2$ $[M+Na]^+$: 376.0743, found 376.0738; **IR** (KBr): 3272, 3088, 2934, 2858, 1644, 1555, 1281, 1126, 908, 702 cm^{-1}.

***N*-((3,4-Dihydro-2*H*-pyran-6-yl)methyl)benzamide 1f**: yield: 66%; (Silica gel column

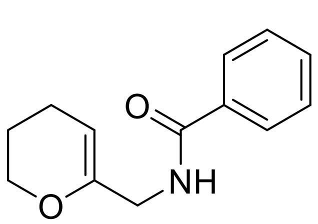

1f

chromatography eluent, *n*-hexane/ethyl acetate = 4/1 with 1% TEA); white solid; ^{1}**H-NMR** (400 MHz, $CDCl_3$) δ 7.78 (d, J = 7.3 Hz, 2H), 7.46-7.50 (m, 1H), 7.39-7.43 (m, 2H), 6.48 (s, 1H), 4.77 (s, 1H), 3.97-4.03 (m, 4H), 1.99-2.04 (s, 2H), 1.79-1.83 (m, 2H); ^{13}**C-NMR** (100 MHz, $CDCl_3$) δ 167.31, 150.17, 134.67, 131.47, 128.58, 127.08, 97.98, 66.51, 42.88, 22.29, 20.05; **HRMS** (ESI) calculated for $C_{13}H_{15}NNaO_2$ $[M+Na]^+$: 240.0995, found 240.1000; **IR** (KBr): 3313, 3064, 2928, 2871, 1645, 1547, 1235, 1154, 1062, 916 cm^{-1}.

3-Bromo-*N*-((3,4-dihydro-2*H*-pyran-6-yl)methyl)benzamide 1g: yield: 59%; (Silica gel column

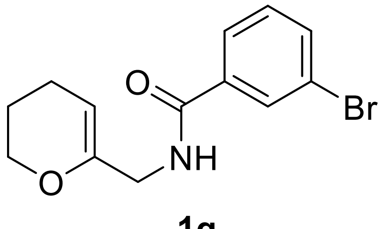

1g

chromatography eluent, *n*-hexane/ethyl acetate = 5/1 with 1% TEA); white solid; ^{1}**H-NMR** (400 MHz, $CDCl_3$) δ 7.92 (t, J = 1.8 Hz, 1H), 7.70 (dt, J = 7.8, 1.4 Hz, 1H), 7.61 (dq, J = 8.1, 1.0 Hz, 1H), 7.29 (t, J = 7.8 Hz, 1H), 6.38 (s, 1H), 4.78 (t, J = 3.7 Hz, 1H), 4.03 (t, J = 5.0 Hz, 2H), 3.97 (d, J = 5.0 Hz, 2H), 2.00-2.05 (m, 2H), 1.79-1.84 (m, 2H); ^{13}**C-NMR** (100 MHz, $CDCl_3$) δ 165.88, 149.87, 136.70, 134.49, 130.34, 130.23, 125.72, 122.84, 98.42, 66.61, 43.11, 22.29, 20.09; **HRMS** (ESI) calculated for $C_{13}H_{14}BrNNaO_2$ $[M+Na]^+$: 318.0100, found 318.0100; **IR** (KBr): 3296, 3076, 2949, 2848, 1633, 1549, 1318, 1233, 1068, 916 cm^{-1}.

N-((3,4-Dihydro-2H-pyran-6-yl)methyl)-2-naphthamide 1h: yield: 73%; (Silica gel column chromatography eluent, *n*-hexane/ethyl acetate = 5/1 with 1% TEA); white solid; **^{1}H-NMR** (400 MHz, $CDCl_3$) δ 8.30 (s, 1H), 7.83-7.93 (m, 4H), 7.51-7.58 (m, 2H), 6.56 (s, 1H), 4.83 (t, J = 3.9 Hz, 1H), 4.04-4.07 (m, 4H), 2.02-2.06 (m, 2H), 1.80-1.86 (m, 2H); **^{13}C-NMR** (100 MHz, $CDCl_3$) δ 167.38, 150.20, 134.84, 132.76, 131.93, 129.05, 128.50, 127.86, 127.70, 127.54, 126.82, 123.79, 98.19, 66.60, 43.08, 22.35, 20.11; **HRMS** (ESI) calculated for $C_{17}H_{17}NNaO_2$ $[M+Na]^+$: 290.1152, found 290.1143; **IR** (KBr): 3322, 3062, 2960, 2871, 1634, 1546, 1312, 1248, 1064, 913 cm^{-1}.

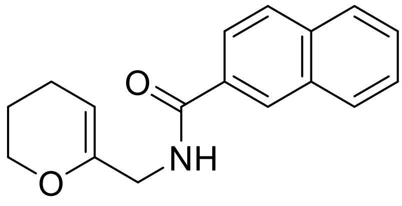

1h

N-((3,4-Dihydro-2H-pyran-6-yl)methyl)-4-iodobenzamide 1i: yield: 76%; (Silica gel column chromatography eluent, *n*-hexane/ethyl acetate = 8/1 with 1% TEA); white solid; **^{1}H-NMR** (400 MHz, $CDCl_3$) δ 7.78 (d, J = 8.7 Hz, 2H), 7.51 (d, J = 8.2 Hz, 2H), 6.32 (s, 1H), 4.79 (t, J = 3.7 Hz, 1H), 4.03 (t, J = 5.0 Hz, 2H), 3.98 (d, J = 5.5 Hz, 2H), 2.01-2.05 (m, 2H), 1.79-1.85 (m, 2H); **^{13}C-NMR** (100 MHz, $CDCl_3$) δ 166.54, 149.92, 137.84, 134.07, 128.75, 98.46, 98.36, 66.59, 43.04, 22.29, 20.08; **HRMS** (ESI) calculated for $C_{13}H_{14}INNaO_2$ $[M+Na]^+$: 365.9961, found 365.9956; **IR** (KBr): 3291, 3075, 2928, 2841, 1631, 1544, 1311, 1240, 1062, 849 cm^{-1}.

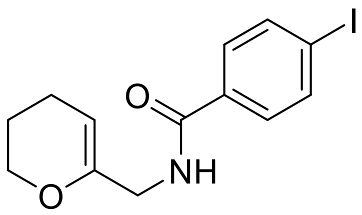

1i

N-((3,4-Dihydro-2H-pyran-6-yl)methyl)-4-fluorobenzamide 1j: yield: 43%; (Silica gel column chromatography eluent, *n*-hexane/ethyl acetate = 7/1 with 1% TEA); white solid; **^{1}H-NMR** (400 MHz, $CDCl_3$) δ 7.79 (dd, J = 8.7, 5.0 Hz, 2H), 7.10 (t, J = 8.7 Hz, 2H), 6.33 (s, 1H), 4.79 (t, J = 3.7 Hz, 1H), 4.03 (t, J = 5.0 Hz, 2H), 3.98 (d, J = 4.6 Hz, 2H), 2.01-2.05 (m, 2H), 1.79-1.85 (m, 2H); **^{13}C-NMR** (100 MHz, $CDCl_3$) δ 166.29, 164.83 (d, $J_{C\text{-}F}$ = 251.1 Hz), 150.03, 130.86 (d, $J_{C\text{-}F}$ = 2.9 Hz), 129.43 (d, $J_{C\text{-}F}$ = 8.6 Hz), 115.68 (d, $J_{C\text{-}F}$ = 22.0 Hz), 98.35, 66.61, 43.07, 22.32, 20.10; **HRMS** (ESI) calculated for $C_{13}H_{14}FNNaO_2$ $[M+Na]^+$: 258.0901, found 258.0899; **IR** (KBr): 3301, 3052, 2926, 2846, 1642, 1587, 1315, 1238, 1072, 856 cm^{-1}.

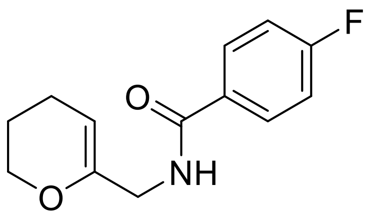

1j

4-(*tert*-Butyl)-*N*-((3,4-dihydro-2*H*-pyran-6-yl)methyl)benzamide 1k: yield: 67%; (Silica gel column chromatography eluent, *n*-hexane/ethyl acetate = 6/1 with 1% TEA); white solid; ^{1}**H-NMR** (400 MHz, $CDCl_3$) δ 7.72 (d, J = 8.7 Hz, 2H), 7.44 (d, J = 8.7 Hz, 2H), 6.34 (s, 1H), 4.78 (t, J = 3.7 Hz, 1H), 3.98-4.03 (m, 4H), 2.00-2.04 (m, 2H), 1.78-1.84 (m, 2H), 1.33 (s, 9H); ^{13}**C-NMR** (100 MHz, $CDCl_3$) δ 167.29, 155.01, 150.34, 131.90, 126.93, 125.59, 97.99, 66.58, 42.86, 35.04, 31.31, 22.37, 20.11; **HRMS** (ESI) calculated for $C_{16}H_{23}NNaO_2$ $[M+Na]^+$: 296.1621, found 296.1619; **IR** (KBr): 3328, 3020, 2928, 2852, 1635, 1548, 1315, 1250, 1062, 912 cm^{-1}.

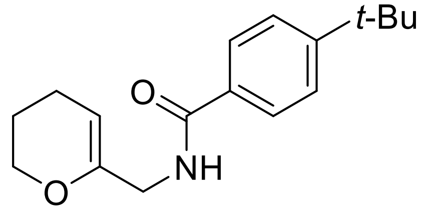

1k

(5*S*,10*R*)-10-Iodo-2-(4-nitrophenyl)-1,6-dioxa-3-azaspiro[4.5]dec-2-ene 2a: yield: 95%; (Silica gel column chromatography eluent, *n*-hexane/ethyl acetate = 9/1); white solid; $[\alpha]_D^{23}$ = -163.34 (c 0.0028 g/mL, $CHCl_3$); ^{1}**H-NMR** (400 MHz, $CDCl_3$) δ 8.28 (d, J = 9.2 Hz, 2H), 8.11 (d, J = 8.7 Hz, 2H), 4.47 (t, J = 3.2 Hz, 1H), 4.12 (td, J = 11.9, 2.6 Hz, 1H), 4.01 (s, 2H), 3.87-3.91 (m, 1H), 2.25-2.43 (m, 2H), 2.16-2.20 (m, 1H), 1.59-1.63 (m, 1H); ^{13}**C-NMR** (100 MHz, $CDCl_3$) δ 160.76, 149.72, 133.84, 129.16, 123.77, 108.40, 69.42, 63.31, 31.30, 30.55, 21.01; **HRMS** (ESI) calculated for $C_{13}H_{13}IN_2NaO_4$ $[M+Na]^+$: 410.9812, found 410.9810; **IR** (KBr): 3109, 3079, 2954, 2884, 1726, 1652, 1521, 1346, 1077, 865 cm^{-1}. **Enantiomeric ratio**: 90:10, determined by HPLC (Daicel Chiralpak IBN-5, *n*-hexane / isopropanol = 20/1, flow rate 1.0 mL/min, T = 25 ºC, 270 nm): t_{maj} = 16.32 min, t_{min} = 17.79 min.

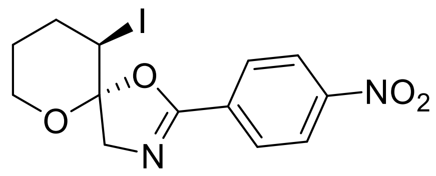

2a

(5*S*,10*R*)-2-(4-Chlorophenyl)-10-iodo-1,6-dioxa-3-azaspiro[4.5]dec-2-ene 2b: yield: 98%; (Silica gel column chromatography eluent, *n*-hexane/ethyl acetate = 10/1); white solid; $[\alpha]_D^{25}$ = -32.54 (c 0.0061 g/mL, $CHCl_3$); ^{1}**H-NMR** (400 MHz, $CDCl_3$) δ 7.86 (d, J = 8.7 Hz, 2H), 7.40 (d, J = 8.2 Hz, 2H), 4.45 (t, J = 2.7 Hz, 1H), 4.12 (td, J = 11.9, 2.6 Hz, 1H), 3.95 (s, 2H), 3.84-3.88 (m, 1H), 2.26-2.42 (m, 2H), 2.13-2.18 (m, 1H), 1.53-1.59 (1H); ^{13}**C-NMR** (100 MHz, $CDCl_3$) δ 161.68, 137.90, 129.48, 128.89, 126.56, 107.78, 69.24, 63.15, 31.83, 30.63, 21.10; **HRMS** (ESI) calculated for $C_{13}H_{13}ClINNaO_2$ $[M+Na]^+$: 399.9572, found 399.9570; **IR** (KBr): 3050, 2989, 2954, 2858, 1700, 1684, 1555, 1327, 1068, 850 cm^{-1}. **Enantiomeric ratio**: 57:43, determined by HPLC (Daicel Chiralpak IC, *n*-hexane / isopropanol = 20/1, flow rate 0.5

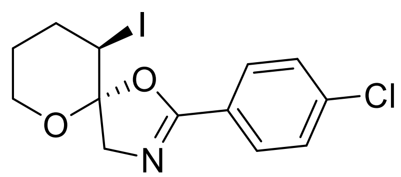

2b

mL/min, T = 25 ºC, 254 nm): t_{maj} = 11.26 min, t_{min} = 10.49 min.

(5*S*,10*R*)-2-(4-Bromophenyl)-10-iodo-1,6-dioxa-3-azaspiro[4.5]dec-2-ene 2c: yield: 97%; (Silica

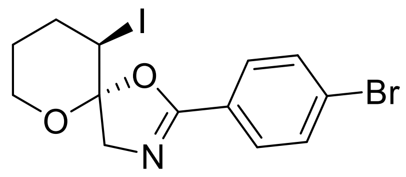

2c

gel column chromatography eluent, *n*-hexane/acetone = 12/1); white solid; **[α]$_D^{24}$** = -16.92 (c 0.0024 g/mL, $CHCl_3$); ^{1}**H-NMR** (400 MHz, $CDCl_3$) δ 7.79 (d, J = 8.2 Hz, 2H), 7.56 (d, J = 8.7 Hz, 2H), 4.45 (t, J = 3.2 Hz, 1H), 4.11 (td, J = 11.9, 2.6 Hz, 1H), 3.94 (s, 2H), 3.83-3.88 (m, 1H), 2.25-2.41 (m, 2H), 2.13-2.19 (m, 1H), 1.55-1.59 (m, 1H); ^{13}**C-NMR** (100 MHz, $CDCl_3$) δ 161.79, 131.85, 129.66, 127.01, 126.39, 107.79, 69.24, 63.15, 31.80, 30.62, 21.10; **HRMS** (ESI) calculated for $C_{13}H_{13}BrINNaO_2$ $[M+Na]^+$: 443.9067, found 443.9069; **IR** (KBr): 3022, 2927, 2880, 2865, 1668, 1524, 1312, 1087, 912, 845 cm^{-1}. **Enantiomeric ratio**: 64:36, determined by HPLC (Daicel Chiralpak IBN-5, *n*-hexane / isopropanol = 20/1, flow rate 0.75 mL/min, T = 25 ºC, 538 nm): t_{maj} = 9.56 min, t_{min} = 10.12 min.

(5*S*,10*R*)-10-Iodo-2-(*p*-tolyl)-1,6-dioxa-3-azaspiro[4.5]dec-2-ene 2d: yield: 89%; (Silica gel

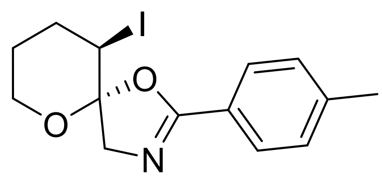

2d

column chromatography eluent, *n*-hexane/ethyl acetate = 10/1); white solid; **[α]$_D^{25}$** = -146.78 (c 0.0045 g/mL, $CHCl_3$); ^{1}**H-NMR** (400 MHz, $CDCl_3$) δ 7.81 (d, J = 8.2 Hz, 2H), 7.22 (d, J = 7.8 Hz, 2H), 4.46 (t, J = 3.0 Hz, 1H), 4.14 (td, J = 11.9, 2.7 Hz, 1H), 3.94 (s, 2H), 3.83-3.88 (m, 1H), 2.40 (s, 3H), 2.23-2.42 (m, 2H), 2.12-2.18 (m, 1H), 1.54-1.58 (m, 1H); ^{13}**C-NMR** (100 MHz, $CDCl_3$) δ 162.64, 142.12, 129.27, 128.10, 125.25, 107.31, 69.18, 63.05, 32.28, 30.67, 21.75, 21.15; **HRMS** (ESI) calculated for $C_{14}H_{16}INNaO_2$ $[M+Na]^+$: 380.0118, found 380.0121; **IR** (KBr): 2992, 2928, 2878, 2854, 1685, 1578, 1423, 1320, 1037, 916 cm^{-1}. **Enantiomeric ratio**: 80:20, determined by HPLC (Daicel Chiralpak IC-3, *n*-hexane / isopropanol = 20/1, flow rate 1.0 mL/min, T = 25 ºC, 254 nm): t_{maj} = 8.85 min, t_{min} = 7.46 min.

(5*S*,10*R*)-2-(3,5-Bis(trifluoromethyl)phenyl)-10-iodo-1,6-dioxa-3-azaspiro[4.5]dec-2-ene 2e:

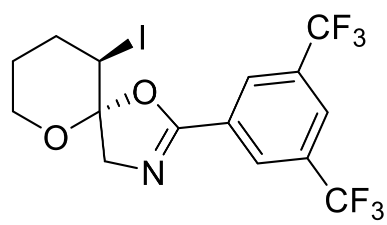

2e

yield: 93%; (Silica gel column chromatography eluent, *n*-hexane/ethyl acetate = 12/1); white solid; **[α]$_D^{24}$** = -187.52 (c 0.0043 g/mL, $CHCl_3$); ^{1}**H-NMR** (400 MHz, $CDCl_3$) δ 8.37 (s, 2H), 7.99 (s, 1H), 4.47 (t, J = 3.0 Hz, 1H), 4.12 (td, J = 11.9, 2.7 Hz, 1H), 4.01 (s, 2H), 3.87-3.91 (m, 1H), 2.16-2.45 (m, 3H), 1.62-1.65 (m, 1H); ^{13}**C-NMR** (100 MHz, $CDCl_3$) δ 160.08, 132.23 (d, J_{C-F} = 33.6 Hz), 130.33, 128.24, 125.04, 122.99 (d, J_{C-}

F = 272.2 Hz), 108.77, 69.25, 63.39, 31.15, 30.58, 20.99; **HRMS** (ESI) calculated for $C_{15}H_{12}F_6INNaO_2$ $[M+Na]^+$: 501.9709, found 501.9704; **IR** (KBr): 3083, 2965, 2932, 2878, 1658, 1444, 1303, 1285, 1174, 908 cm^{-1}. **Enantiomeric ratio**: 79:21, determined by HPLC (Daicel Chiralpak IC-3, *n*-hexane / isopropanol = 999/1, flow rate 0.5 mL/min, T = 25 ºC, 254 nm): t_{maj} = 27.87 min, t_{min} = 24.15 min.

(5*S*,10*R*)-10-Iodo-2-phenyl-1,6-dioxa-3-azaspiro[4.5]dec-2-ene 2f: yield: 96%; (Silica gel column chromatography eluent, *n*-hexane/acetone = 9/1); white solid; **$[\alpha]_D^{25}$** = -63.54 (c 0.0047 g/mL, $CHCl_3$); ^{1}**H-NMR** (400 MHz, $CDCl_3$) δ 7.92-7.95 (m, 2H), 7.47-7.51 (m, 1H), 7.40-7.44 (m, 2H), 4.46 (t, J = 3.0 Hz, 1H), 4.14 (td, J = 11.9, 2.7 Hz, 1H), 3.96 (s, 2H), 3.83-3.88 (m, 1H), 2.23-2.44 (m, 2H), 2.12-2.18 (m, 1H), 1.56-1.60 (m, 1H); ^{13}**C-NMR** (100 MHz, $CDCl_3$) δ 162.51, 131.67, 128.53, 128.12, 128.04, 107.44, 69.21, 63.07, 32.14, 30.64, 21.11; **HRMS** (ESI) calculated for $C_{13}H_{14}INNaO_2$ $[M+Na]^+$: 365.9961, found 365.9958; **IR** (KBr): 3062, 2954, 2926, 2854, 1698, 1558, 1319, 1279, 1165, 852 cm^{-1}. **Enantiomeric ratio**: 70:30, determined by HPLC (Daicel Chiralpak IBN-5, *n*-hexane / isopropanol = 20/1, flow rate 1.0 mL/min, T = 25 ºC, 254 nm): t_{maj} = 7.02 min, t_{min} = 12.71 min.

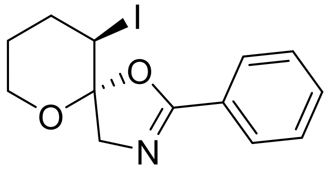

2f

(5*S*,10*R*)-2-(3-Bromophenyl)-10-iodo-1,6-dioxa-3-azaspiro[4.5]dec-2-ene 2g: yield: 94%; (Silica gel column chromatography eluent, *n*-hexane/acetone = 10/1); white solid; **$[\alpha]_D^{24}$** = -20.08 (c 0.0052 g/mL, $CHCl_3$); ^{1}**H-NMR** (400 MHz, $CDCl_3$) δ 8.05 (t, J = 1.8 Hz, 1H), 7.86-7.89 (dt, J = 7.8, 1.4 Hz, 1H), 7.61 (dq, J = 8.0, 1.1 Hz, 1H), 7.30 (t, J = 8.0 Hz, 1H), 4.44 (t, J = 3.0 Hz, 1H), 4.12 (td, J = 12.0, 2.4 Hz, 1H), 3.96 (s, 2H), 3.84-3.88 (m, 1H), 2.23-2.43 (m, 2H), 2.12-2.19 (m, 1H), 1.57-1.61 (m, 1H); ^{13}**C-NMR** (100 MHz, $CDCl_3$) δ 161.23, 134.62, 131.05, 130.13, 130.03, 126.75, 122.57, 107.87, 69.21, 63.18, 31.78, 30.59, 21.05; **HRMS** (ESI) calculated for $C_{13}H_{13}BrINNaO_2$ $[M+Na]^+$: 443.9067, found 443.9067; **IR** (KBr): 3017, 2925, 2882, 2859, 1672, 1525, 1317, 1070, 917, 850 cm^{-1}. **Enantiomeric ratio**: 58:42, determined by HPLC (Daicel Chiralpak IC-3, *n*-hexane / isopropanol = 20/1, flow rate 0.5 mL/min, T = 25 ºC, 273 nm): t_{maj} = 16.79 min, t_{min} = 14.63 min.

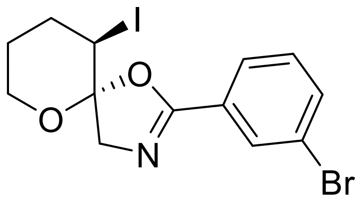

2g

(5*S*,10*R*)-10-Iodo-2-(naphthalen-2-yl)-1,6-dioxa-3-azaspiro[4.5]dec-2-ene 2h: yield: 91%; (Silica gel column chromatography eluent, *n*-hexane/acetone = 9/1); white solid; **[α]D^{25}** = -96.27 (c 0.0038 g/mL, $CHCl_3$); ^{1}**H-NMR** (400 MHz, $CDCl_3$) δ 8.41 (s, 1H), 8.02 (dd, J = 8.7, 1.8 Hz, 1H), 7.92 (dd, J = 8.3, 1.8 Hz, 1H), 7.85-7.88 (m, 2H), 7.51-7.58 (m, 2H), 4.51 (t, J = 3.2 Hz, 1H), 4.21 (td, J = 11.9, 2.6 Hz, 1H), 4.02 (s, 2H), 3.87-3.91 (m, 1H), 2.44-2.52 (m, 1H), 2.26-2.38 (m, 1H), 2.16-2.23 (m, 1H), 1.60-1.65 (m, 1H); ^{13}**C-NMR** (100 MHz, $CDCl_3$) δ 162.69, 134.90, 132.74, 128.97, 128.64, 128.40, 127.97, 127.80, 126.79, 125.35, 124.59, 107.59, 69.30, 63.14, 32.18, 30.72, 21.17; **HRMS** (ESI) calculated for $C_{17}H_{16}INNaO_2$ $[M+Na]^+$: 416.0118, found 416.0120; **IR** (KBr): 2969, 2946, 2923, 2879, 1645, 1433, 1316, 1250, 1076, 899 cm^{-1}. **Enantiomeric ratio**: 78:22, determined by HPLC (Daicel Chiralpak IBN-5, *n*-hexane / isopropanol = 20/1, flow rate 1.0 mL/min, T = 25 ºC, 255 nm): t_{maj} = 8.74 min, t_{min} = 11.99 min.

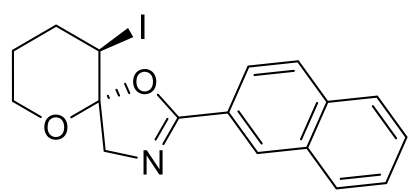

2h

(5*S*,10*R*)-10-Iodo-2-(4-iodophenyl)-1,6-dioxa-3-azaspiro[4.5]dec-2-ene 2i: yield: 99%; (Silica gel column chromatography eluent, *n*-hexane/ethyl acetate = 15/1); white solid; **[α]D^{24}** = -19.76 (c 0.0034 g/mL, $CHCl_3$); ^{1}**H-NMR** (400 MHz, $CDCl_3$) δ 7.77 (d, J = 8.2 Hz, 2H), 7.64 (d, J = 8.2 Hz, 2H), 4.45 (t, J = 3.0 Hz, 1H), 4.11 (td, J = 11.9, 2.7 Hz, 1H), 3.94 (s, 2H), 3.83-3.88 (m, 1H), 2.23-2.42 (m, 2H), 2.13-2.18 (m, 1H), 1.57-1.61 (m, 1H); ^{13}**C-NMR** (150 MHz, $CDCl_3$) δ 161.93, 137.81, 129.63, 127.57, 107.76, 98.66, 69.23, 63.14, 31.81, 30.62, 21.09; **HRMS** (ESI) calculated for $C_{13}H_{13}I_2NNaO_2$ $[M+Na]^+$: 491.8928, found 491.8934; **IR** (KBr): 2951, 2910, 2887, 2848, 1650, 1584, 1391, 1310, 978, 895 cm^{-1}. **Enantiomeric ratio**: 57:43, determined by HPLC (Daicel Chiralpak IBN-5, *n*-hexane / isopropanol = 20/1, flow rate 1.0 mL/min, T = 25 ºC, 254 nm): t_{maj} = 7.41 min, t_{min} = 8.63 min.

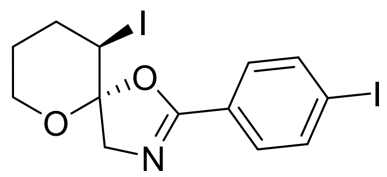

2i

(5*S*,10*R*)-2-(4-Fluorophenyl)-10-iodo-1,6-dioxa-3-azaspiro[4.5]dec-2-ene 2j: yield: 91%; (Silica gel column chromatography eluent, *n*-hexane/ethyl acetate = 12/1); white solid; **[α]D^{24}** = -24.51 (c 0.0041 g/mL, $CHCl_3$); ^{1}**H-NMR** (400 MHz, $CDCl_3$) δ 7.93 (dd, J = 9.2, 5.5 Hz, 2H), 7.10 (t, J = 8.7 Hz, 2H), 4.45 (t, J = 3.0 Hz, 1H), 4.12 (td, J = 11.9, 2.5 Hz, 1H), 3.95 (s, 2H), 3.83-3.88 (m, 1H), 2.23-2.42 (m, 2H), 2.13-2.19 (m, 1H), 1.55-1.61 (m, 1H); ^{13}**C-NMR** (100 MHz, $CDCl_3$) δ 164.93 (d, J_{C-F} = 252.1 Hz), 161.62, 130.41 (d, J_{C-F} =

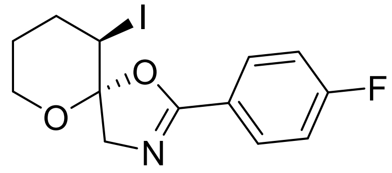

2j

8.6 Hz), 124.32 (d, $J_{C\text{-}F}$ = 2.9 Hz), 115.72 (d, $J_{C\text{-}F}$ = 22.0 Hz), 107.71, 69.21, 63.13, 31.89, 30.66, 21.13; **HRMS** (ESI) calculated for $C_{13}H_{13}FINNaO_2$ $[M+Na]^+$: 383.9867, found 383.9870; **IR** (KBr): 2986, 2932, 2892, 2852, 1665, 1558, 1342, 1289, 1065, 846 cm^{-1}. **Enantiomeric ratio**: 59:41, determined by HPLC (Daicel Chiralpak IC, *n*-hexane / isopropanol = 20/1, flow rate 1.0 mL/min, T = 25 ºC, 225 nm): t_{maj} = 6.22 min, t_{min} = 5.25 min.

(5*S*,10*R*)-2-(4-(*tert*-Butyl)phenyl)-10-iodo-1,6-dioxa-3-azaspiro[4.5]dec-2-ene 2k: yield: 97%;

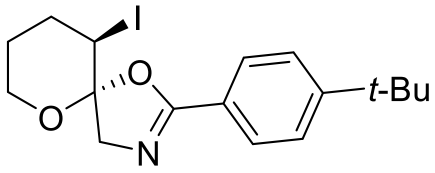

2k

(Silica gel column chromatography eluent, *n*-hexane/ethyl acetate = 10/1); white solid; **[α]$_D$^{23}** = -200.38 (c 0.0023 g/mL, $CHCl_3$); **^{1}H-NMR** (400 MHz, $CDCl_3$) δ 7.86 (d, *J* = 8.7 Hz, 2H), 7.44 (d, *J* = 8.7 Hz, 2H), 4.45 (t, *J* = 3.0 Hz, 1H), 4.13 (td, *J* = 12.0, 2.9 Hz, 1H), 3.94 (s, 2H), 3.82-3.86 (m, 1H), 2.23-2.44 (m, 2H), 2.12-2.18 (m, 1H), 1.55-1.59 (m, 1H), 1.33 (s, 9H); **^{13}C-NMR** (100 MHz, $CDCl_3$) δ 162.55, 155.21, 127.97, 125.52, 125.24, 107.29, 69.20, 63.03, 35.13, 32.29, 31.30, 30.71, 21.18; **HRMS** (ESI) calculated for $C_{17}H_{22}INNaO_2$ $[M+Na]^+$: 422.0587, found 422.0582; **IR** (KBr): 2961, 2952, 2908, 2886, 1650, 1316, 1242, 1076, 895, 688 cm^{-1}. **Enantiomeric ratio**: 83:17, determined by HPLC (Daicel Chiralpak IC, *n*-hexane / isopropanol = 20/1, flow rate 1.0 mL/min, T = 25 ºC, 260 nm): t_{maj} = 10.72 min, t_{min} = 7.22 min.

(2*S*,3*R*)-2-(Benzyloxy)-3-iodotetrahydro-2*H*-pyran 6a (Li et al., 2018): yield: 90%; (Silica gel

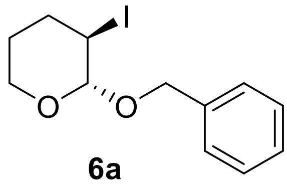

6a

column chromatography eluent, *n*-hexane/ethyl acetate = 9/1); colorless oil; **^{1}H-NMR** (600 MHz, $CDCl_3$) δ 7.39 (d, *J* = 7.3 Hz, 1H), 7.35 (t, *J* = 7.5 Hz, 1H), 7.30 (m, 1H), 4.80 (d, *J* = 11.8 Hz, 1H), 4.73 (d, *J* = 5.2 Hz, 1H), 4.56 (d, *J* = 11.8 Hz, 1H), 4.17-4.12 (m, 1H), 4.04-4.00 (m, 1H), 3.63-3.58 (m, 1H), 2.44-2.32 (m, 1H), 2.06-1.98 (m, 1H), 1.82-1.75 (m, 1H), 1.62-1.55 (m, 1H). **Enantiomeric ratio**: 69:31, determined by HPLC (Daicel Chiralpak IB, *n*-hexane / isopropanol = 99/1, flow rate 1.0 mL/min, T = 25 ºC, 275 nm): t_{maj} = 9.67 min, t_{min} = 10.93 min.

(2*S*,3*R*)-3-Iodo-2-(naphthalen-2-ylmethoxy)tetrahydro-2*H*-pyran 6b: yield: 89%; (Silica gel

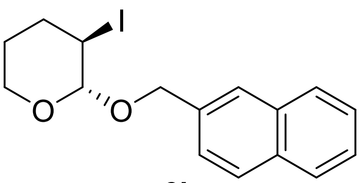

6b

column chromatography eluent, *n*-hexane/ethyl acetate = 7/1); colorless oil; **[α]$_D$^{25}** = -160.38 (c 0.0053 g/mL, $CHCl_3$); **^{1}H-NMR** (400 MHz, $CDCl_3$) δ 7.83-7.85 (m, 4H), 7.53 (dd, *J* = 8.7, 1.4 Hz, 1H), 7.46-7.51 (m, 2H), 4.97 (d, *J* = 11.9 Hz, 1H), 4.77 (d, *J* = 5.0 Hz, 1H), 4.74

(d, J = 11.9 Hz, 1H), 4.16-4.20 (m, 1H), 4.04-4.09 (m, 1H), 3.60-3.66 (m, 1H), 2.38-2.46 (m, 1H), 1.99-2.07 (m, 1H), 1.75-1.84 (m, 1H), 1.57-1.66 (m, 1H); ^{13}**C-NMR** (100 MHz, $CDCl_3$) δ 134.82, 133.33, 133.15, 128.28, 128.06, 127.82, 127.00, 126.22, 126.20, 126.08, 101.57, 70.02, 63.76, 32.86, 29.25, 25.70; **HRMS** (ESI) calculated for $C_{16}H_{17}INaO_2$ $[M+Na]^+$: 391.0165, found 391.0163; **IR** (KBr): 3053, 2944, 2923, 2850, 1123, 1066, 1028, 816, 753, 475 cm^{-1}. **Enantiomeric ratio**: 74:26, determined by HPLC (Daicel Chiralpak OJ-H, *n*-hexane / ethanol = 99/1, flow rate 2.0 mL/min, T = 25 ºC, 275 nm): t_{maj} = 28.43 min, t_{min} = 26.11 min.

(2*S*,3*R*)-3-Iodo-2-((4-nitrobenzyl)oxy)tetrahydro-2*H*-pyran 6c (Li et al., 2018): yield: 96%; (Silica

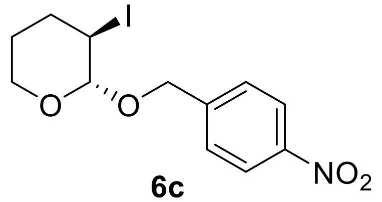

6c

gel column chromatography eluent, *n*-hexane/ethyl acetate = 7/1); white solid; ^{1}**H-NMR** (400 MHz, $CDCl_3$) δ 8.22 (d, J = 7.7 Hz, 1H), 7.57 (d, J = 8.0 Hz, 1H), 4.92 (d, J = 13.1 Hz, 1H), 4.72 (d, J = 5.8 Hz, 1H), 4.68 (d, J = 13.1 Hz, 1H), 4.16-4.12 (m, 1H), 4.07-4.01 (m, 1H), 3.65-3.60 (m, 1H), 2.47-2.41 (m, 1H), 2.14-2.06 (m, 1H), 1.79-1.72 (m, 1H), 1.69-1.62 (m, 1H). **Enantiomeric ratio**: 85:15, determined by HPLC (Daicel Chiralpak IB, *n*-hexane / isopropanol = 99/1, flow rate 1.0 mL/min, T = 25 ºC, 254 nm): t_{maj} = 24.12 min, t_{min} = 22.41 min.

(2*S*,3*R*)-3-Iodo-2-((4-nitrobenzyl)oxy)tetrahydrofuran 6d (Li et al., 2018): yield: 84%; (Silica gel

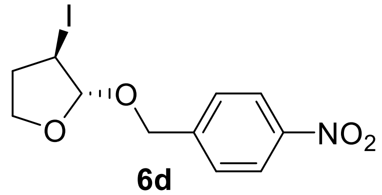

6d

column chromatography eluent, *n*-hexane/ethyl acetate = 10/1); white solid; ^{1}**H-NMR** (600 MHz, $CDCl_3$) δ 8.20 (d, J = 8.7 Hz, 2H), 7.47 (d, J = 8.7 Hz, 2H), 5.45 (s, 1H), 4.78 (d, J = 13.1 Hz, 1H), 4.60 (d, J = 13.1 Hz, 1H), 4.24 (dd, J = 6.3, 2.2 Hz, 1H), 4.18-4.14 (m, 1H), 4.03 (td, J = 8.3, 3.7 Hz, 1H), 2.69-2.63 (m, 1H), 2.27-2.20 (m, 1H). **Enantiomeric ratio**: 66:34, determined by HPLC (Daicel Chiralpak IBN-5, *n*-hexane / isopropanol = 99/1, flow rate 1.0 mL/min, T = 25 ºC, 230 nm): t_{maj} = 19.86 min, t_{min} = 21.55 min.

(2*S*,3*R*)-2-((2,4-Dinitrobenzyl)oxy)-3-iodotetrahydro-2*H*-pyran 6e: yield: 87%; (Silica gel

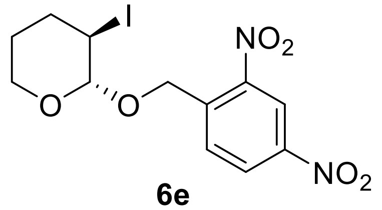

6e

column chromatography eluent, *n*-hexane/ethyl acetate = 6/1); yellowish white solid; $[\alpha]_D^{25}$ = -133.37 (c 0.0092 g/mL, $CHCl_3$); ^{1}**H-NMR** (600 MHz, $CDCl_3$) δ 8.95 (d, J = 2.1 Hz, 1H), 8.51 (dd, J = 8.9, 2.1 Hz, 1H), 8.23 (d, J = 8.9 Hz, 1H), 5.31 (d, J = 16.5 Hz, 1H), 5.08 (d, J = 16.5 Hz, 1H), 4.75 (d, J = 6.9 Hz, 1H), 4.12-4.16 (m, 1H), 4.03-4.07 (m, 1H), 3.63-3.67 (m, 1H), 2.46-2.51 (m, 1H), 2.10-2.16 (m, 1H), 1.68-171 (m, 2H); ^{13}**C-NMR** (150 MHz, $CDCl_3$) δ 147.10,

146.71, 141.67, 130.47, 127.92, 120.29, 103.71, 66.91, 65.36, 34.45, 28.01, 26.90; **HRMS** (ESI) calculated for $C_{12}H_{13}IN_2NaO_6$ $[M+Na]^+$: 430.9711, found 430.9707; **IR** (KBr): 3092, 3074, 2943, 2866, 1530, 1343, 1063, 1026, 730, 699 cm^{-1}. **Enantiomeric ratio**: 78:22, determined by HPLC (Daicel Chiralpak IBN-5, *n*-hexane / isopropanol = 20/1, flow rate 1.0 mL/min, T = 25 ºC, 254 nm): t_{maj} = 32.41 min, t_{min} = 28.55 min.

(2*S*,3*R*)-3-Iodo-2-((4-methoxybenzyl)oxy)tetrahydro-2*H*-pyran 6f (Li et al., 2018): yield: 99%;

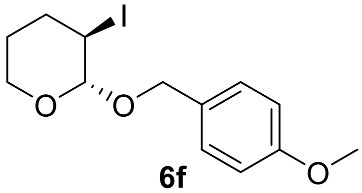

6f

(Silica gel column chromatography eluent, *n*-hexane/ethyl acetate = 10/1); colorless oil; ^{1}**H-NMR** (600 MHz, $CDCl_3$) δ 7.31 (d, J = 8.5 Hz, 2H), 6.88 (d, J = 8.5 Hz, 2H), 4.73 (d, J = 11.4 Hz, 1H), 4.70 (d, J = 5.3 Hz, 1H), 4.50 (d, J = 11.4 Hz, 1H), 4.16-4.09 (m, 1H), 4.04-3.99 (m, 1H), 3.81 (s, 3H), 3.62-3.57 (m, 1H), 2.40-2.36 (m, 1H), 2.04-1.98 (m, 1H), 1.81-1.75 (m, 1H), 1.63-1.56 (m, 1H). **Enantiomeric ratio**: 70:30, determined by HPLC (Daicel Chiralpak IBN-5, *n*-hexane / isopropanol = 200/1, flow rate 1.0 mL/min, T = 25 ºC, 235 nm): t_{maj} = 19.86 min, t_{min} = 17.55 min.

(2*S*,3*R*)-2-((4-Bromobenzyl)oxy)-3-iodotetrahydro-2*H*-pyran 6g: yield: 91%; (Silica gel column

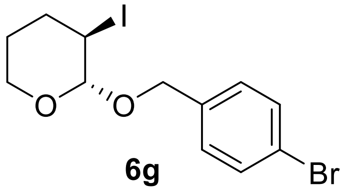

6g

chromatography eluent, *n*-hexane/ethyl acetate = 10/1); colorless oil; $[\alpha]_D^{20}$ = -148.14 (c 0.006 g/mL, $CHCl_3$); ^{1}**H-NMR** (600 MHz, $CDCl_3$) δ 7.47 (d, J = 8.2 Hz, 2H), 7.27 (d, J = 8.2 Hz, 2H), 4.75 (d, J = 11.7 Hz, 1H), 4.69 (d, J = 5.5 Hz, 1H), 4.51 (d, J = 11.7 Hz, 1H), 4.10-4.13 (m, 1H), 3.99-4.03 (m, 1H), 3.58-3.62 (m, 1H), 2.37-2.42 (m, 1H), 2.00-2.06 (m, 1H), 1.73-1.79 (m, 1H), 1.57-1.64 (m, 1H); ^{13}**C-NMR** (150 MHz, $CDCl_3$) δ 136.41, 131.60, 129.74, 121.82, 101.85, 69.23, 63.97, 33.06, 29.00, 25.86; **HRMS** (ESI) calculated for $C_{12}H_{14}BrINaO_2$ $[M+Na]^+$: 418.9114, found 418.9113; **IR** (KBr): 2938, 2861, 1696, 1586, 1484, 1151, 1066, 1010, 826, 800 cm^{-1}. **Enantiomeric ratio**: 78:22, determined by HPLC (Daicel Chiralpak OJ-H, *n*-hexane / isopropanol = 200/1, flow rate 0.75 mL/min, T = 25 ºC, 225 nm): t_{maj} = 40.43 min, t_{min} = 44.51 min.

(2*S*,3*R*)-2-((3-Bromobenzyl)oxy)-3-iodotetrahydro-2*H*-pyran 6h: yield: 93%; (Silica gel column

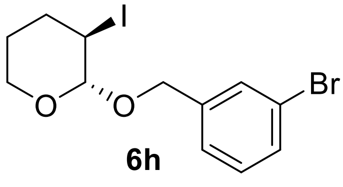

6h

chromatography eluent, *n*-hexane/ethyl acetate = 20/1); colorless oil; $[\alpha]_D^{24.8}$ = -160.14 (c 0.007 g/mL, $CHCl_3$); ^{1}**H-NMR** (400 MHz, $CDCl_3$) δ 7.55 (s, 1H), 7.42 (d, J = 7.8 Hz, 1H), 7.31 (d, J = 7.8 Hz, 1H), 7.22 (t, J = 7.8 Hz, 1H), 4.77 (d, J = 12.4 Hz, 1H), 4.71 (d, J = 5.5 Hz, 1H), 4.53

(d, J = 12.4 Hz, 1H), 4.11-4.16 (m, 1H), 3.98-4.04 (m, 1H), 3.58-3.64 (m, 1H), 2.36-2.43 (m, 1H), 1.99-2.07 (m, 1H), 1.73-1.82 (m, 1H), 1.56-1.65 (m, 1H); ^{13}**C-NMR** (100 MHz, $CDCl_3$) δ 139.74, 130.91, 130.05, 126.45, 122.55, 101.85, 69.04, 63.84, 32.87, 28.89, 25.69 (One carbon overlapped); **HRMS** (ESI) calculated for $C_{12}H_{14}BrINaO_2$ $[M+Na]^+$: 418.9114, found 418.9114; **IR** (KBr): 3066, 2945, 2888, 2848, 1206, 1132, 1065, 1033, 865, 745 cm^{-1}. **Enantiomeric ratio**: 79:21, determined by HPLC (Daicel Chiralpak IBN-5, n-hexane / isopropanol = 200/1, flow rate 1.0 mL/min, T = 25 ºC, 250 nm): t_{maj} = 12.60 min, t_{min} = 13.75 min.

(2*S*,3*R*)-2-((2-Bromobenzyl)oxy)-3-iodotetrahydro-2*H*-pyran 6i: yield: 90%; (Silica gel column

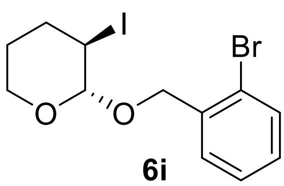

6i

chromatography eluent, n-hexane/ethyl acetate = 15/1); colorless oil; **$[\alpha]_D^{25}$** = -123.46 (c 0.0026 g/mL, $CHCl_3$); ^{1}**H-NMR** (400 MHz, $CDCl_3$) δ 7.53-7.58 (m, 2H), 7.33 (ddd, J = 7.8, 7.3, 0.9 Hz, 1H), 7.16 (ddd, J = 7.8, 7.6, 1.5 Hz, 1H), 4.88 (d, J = 13.3 Hz, 1H), 4.80 (d, J = 5.0 Hz, 1H), 4.63 (d, J = 12.8 Hz, 1H), 4.17-4.22 (m, 1H), 4.02-4.07 (m, 1H), 3.61-3.67 (m, 1H), 2.38-2.45 (m, 1H), 2.00-2.09 (m, 1H), 1.77-1.86 (m, 1H), 1.56-1.66 (m, 1H); ^{13}**C-NMR** (100 MHz, $CDCl_3$) δ 136.97, 132.57, 129.43, 129.14, 127.50, 122.74, 102.19, 69.45, 63.74, 32.70, 28.92, 25.52; **HRMS** (ESI) calculated for $C_{12}H_{14}BrINaO_2$ $[M+Na]^+$: 418.9114, found 418.9112; **IR** (KBr): 3065, 2943, 2874, 2848, 1438, 1204, 1127, 1066, 1023, 750 cm^{-1}. **Enantiomeric ratio**: 78:22, determined by HPLC (Daicel Chiralpak IC-3, n-hexane / isopropanol = 300/1, flow rate 1.0 mL/min, T = 25 ºC, 230 nm): t_{maj} = 9.98 min, t_{min} = 9.28 min.

(2*S*,3*R*)-2-(Benzhydryloxy)-3-iodotetrahydro-2*H*-pyran 6j: yield: 91%; (Silica gel column

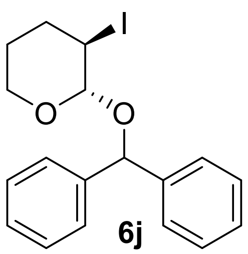

6j

chromatography eluent, n-hexane/ethyl acetate = 10/1); colorless oil; **$[\alpha]_D^{20}$** = -173.16 (c 0.0076 g/mL, $CHCl_3$); ^{1}**H-NMR** (400 MHz, $CDCl_3$) δ 7.24-7.43 (m, 9H), 7.18-7.22 (m, 1H), 5.82 (s, 1H), 4.65 (d, J = 5.0 Hz, 1H), 4.19-4.23 (m, 1H), 3.93-3.98 (m, 1H), 3.48-3.53 (m, 1H), 2.36-2.43 (m, 1H), 1.92-2.00 (m, 1H), 1.72-1.81 (m, 1H), 1.50-1.59 (m, 1H); ^{13}**C-NMR** (100 MHz, $CDCl_3$) δ 142.25, 140.53, 128.58, 128.31, 128.07, 128.02, 127.30, 126.67, 98.98, 79.12, 63.34, 32.32, 29.26, 25.30; **HRMS** (ESI) calculated for $C_{18}H_{19}INaO_2$ $[M+Na]^+$: 417.0322, found 417.0318; **IR** (KBr): 3025, 2973, 2951, 2863, 1494, 1454, 1386, 1125, 942, 750 cm^{-1}. **Enantiomeric ratio**: 83:17, determined by HPLC (Daicel Chiralpak IBN-5, n-hexane, flow rate 1.0 mL/min, T = 25 ºC, 230 nm): t_{maj} = 20.80 min, t_{min} = 24.10 min.

(2*S*,3*R*)-2-((9*H*-fluoren-9-yl)oxy)-3-iodotetrahydro-2*H*-pyran 6k: yield: 89%; (Silica gel column chromatography eluent, *n*-hexane/ethyl acetate = 10/1); white solid; **[α]$_D^{20}$** = -109.25 (c 0.0059 g/mL, $CHCl_3$); ^{1}**H-NMR** (400 MHz, $CDCl_3$) δ 7.73 (dd, J = 7.3, 2.7 Hz, 2H), 7.64 (dd, J = 7.8, 3.7 Hz, 2H), 7.36-7.41 (m, 2H), 7.28-7.32 (m, 2H), 5.64 (s, 1H), 5.20 (d, J = 5.5 Hz, 1H), 4.16-4.24 (m, 2H), 3.71-3.76 (m, 1H), 2.41-2.48 (m, 1H), 2.07-2.16 (m, 1H), 1.78-1.87 (m, 1H), 1.62-1.72 (m, 1H); ^{13}**C-NMR** (100 MHz, $CDCl_3$) δ 143.44, 143.28, 140.76, 140.56, 129.26, 129.19, 127.78, 127.64, 126.58, 126.12, 120.02, 119.92, 103.65, 81.16, 64.23, 33.29, 29.73, 25.90; **HRMS** (ESI) calculated for $C_{18}H_{17}INaO_2$ [M+Na]$^+$: 415.0165, found 415.0163; **IR** (KBr): 3040, 2971, 2936, 2856, 1714, 1610, 1449, 1320, 1133, 767 cm^{-1}. **Enantiomeric ratio**: 75:25, determined by HPLC (Daicel Chiralpak IF-3, *n*-hexane / isopropanol = 200/1, flow rate 0.75 mL/min, T = 25 ºC, 260 nm): t_{maj} = 17.12 min, t_{min} = 18.61 min.

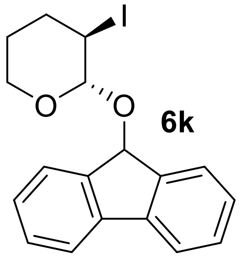

(2*S*,3*R*)-2-(Bis(4-chlorophenyl)methoxy)-3-iodotetrahydro-2*H*-pyran 6l: yield: 90%; (Silica gel column chromatography eluent, *n*-hexane/ethyl acetate = 15/1); white solid; **[α]$_D^{20}$** = -215.89 (c 0.0046 g/mL, $CHCl_3$); ^{1}**H-NMR** (400 MHz, $CDCl_3$) δ 7.30-7.35 (m, 4H), 7.24-7.27 (m, 4H), 5.77 (s, 1H), 4.56 (d, J = 5.5 Hz, 1H), 4.13-4.18 (m, 1H), 3.93-3.98 (m, 1H), 3.48-3.54 (m, 1H), 2.37-2.43 (m, 1H), 1.95-2.04 (m, 1H), 1.70-1.74 (m, 1H), 1.55-1.64 (m, 1H); ^{13}**C-NMR** (100 MHz, $CDCl_3$) δ 140.35, 138.61, 134.10, 133.30, 129.44, 128.93, 128.58, 128.01, 99.48, 77.93, 64.05, 33.09, 28.71, 25.89; **HRMS** (ESI) calculated for $C_{18}H_{17}Cl_2INaO_2$ [M+Na]$^+$: 484.9543, found 484.9537; **IR** (KBr): 3084, 3027, 2942, 2865, 1719, 1592, 1490, 1090, 1013, 796 cm^{-1}. **Enantiomeric ratio**: 90:10, determined by HPLC (Daicel Chiralpak IF-3, *n*-hexane only, flow rate 1.0 mL/min, T = 25 ºC, 235 nm): t_{maj} = 37.24 min, t_{min} = 43.36 min.

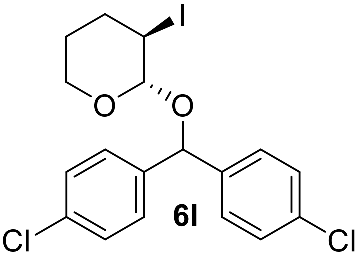

(2*S*,3*R*)-3-Iodo-2-((*S*)-1-phenylethoxy)tetrahydro-2*H*-pyran 6m: Two inseparable diastereomers yield: 93%; (Silica gel column chromatography eluent, *n*-hexane/ethyl acetate = 10/1); colorless oil; **[α]$_D^{25}$** = -99.81 (c 0.0045 g/mL, $CHCl_3$); ^{1}**H-NMR** (600 MHz, $CDCl_3$) δ 7.24-7.39 (m, 10H), 4.81-4.88 (m, 3H), 4.41 (d, J = 5.5 Hz, 1H), 4.14-4.17 (m, 1H), 4.05-4.08 (m, 1H), 4.01-4.04 (m, 1H), 3.82-3.85 (m, 1H), 3.50-3.53 (m, 1H), 3.45-3.48 (m, 1H), 2.36-2.41 (m, 2H), 2.00-2.06 (m, 1H), 1.92-1.98 (m, 1H), 1.70-1.74 (m, 2H), 1.57-1.62 (m, 1H), 1.50-1.55 (m, 1H), 1.48 (s, 3H), 1.48 (s, 3H);

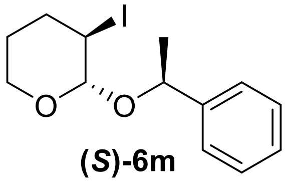

^{13}**C-NMR** (150 MHz, $CDCl_3$) δ 143.66, 142.31, 128.47, 128.34, 127.87, 127.39, 127.03, 126.14, 100.78, 99.34, 75.39, 74.33, 63.74, 33.12, 32.99, 30.37, 29.49, 25.99, 25.66, 24.30, 22.02 (one carbon overlapped) (representing two inseparable diastereomers); **HRMS** (ESI) calculated for $C_{13}H_{17}INaO_2$ $[M+Na]^+$: 355.0165, found 355.0163; **IR** (KBr): 2973, 2951, 2930, 2849, 2350, 1135, 1023, 867, 697, 481 cm^{-1}. **Diastereomeric ratio**: 71:29, determined by HPLC (Daicel Chiralpak IBN-5, *n*-hexane / isopropanol = 99/1, flow rate 1.0 mL/min, T = 25 ºC, 210 nm): t_{maj} = 21.17 min, t_{min} = 34.18 min.

(2*S*,3*R*)-3-Iodo-2-((*R*)-1-phenylethoxy)tetrahydro-2*H*-pyran 6m: yield: 97%; (Silica gel column

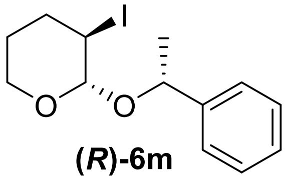

(*R*)-6m

chromatography eluent, *n*-hexane/ethyl acetate = 10/1); colorless oil; **$[\alpha]_D^{25}$** = -61.23 (c 0.0052 g/mL, $CHCl_3$); ^{1}**H-NMR** (600 MHz, $CDCl_3$) δ 7.24-7.39 (m, 10H), 4.81-4.88 (m, 3H), 4.41 (d, *J* = 6.2 Hz, 1H), 4.14-4.17 (m, 1H), 4.01-4.08 (m, 2H), 3.82-3.85 (m, 1H), 3.49-3.53 (m, 1H), 3.44-3.48 (m, 1H), 2.37-2.40 (m, 2H), 2.00-2.06 (m, 1H), 1.92-1.98 (m, 1H), 1.69-1.74 (m, 2H), 1.57-1.62 (m, 1H), 1.50-1.55 (m, 1H), 1.49 (s, 3H), 1.47 (s, 3H); ^{13}**C-NMR** (150 MHz, $CDCl_3$) δ 143.66, 142.31, 128.47, 128.34, 127.87, 127.39, 127.03, 126.14, 100.78, 99.35, 75.39, 74.33, 63.74, 33.12, 32.99, 30.37, 29.48, 25.99, 25.66, 24.30, 22.02 (One carbon overlapped) (representing two inseparable diastereomers); **HRMS** (ESI) calculated for $C_{13}H_{17}INaO_2$ $[M+Na]^+$: 355.0165, found 355.0164; **IR** (KBr): 2964, 2944, 2923, 2850, 2307, 1169, 1064, 1014, 702, 479 cm^{-1}. **Diastereomeric ratio**: 80:20, determined by HPLC (Daicel Chiralpak IBN-5, *n*-hexane / isopropanol = 99/1, flow rate 1.0 mL/min, T = 25 ºC, 210 nm): t_{maj} = 25.81 min, t_{min} = 20.75 min.

Methyl 2-(((2*S*,3*R*)-3-iodotetrahydro-2*H*-pyran-2-yl)oxy)-2-phenylacetate 6n: yield: 83%; two

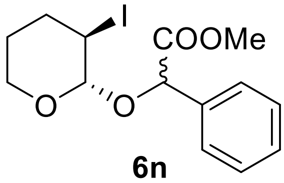

6n

diastereomers that can be separated easily *via* (Silica gel column chromatography eluent, *n*-hexane/ethyl acetate = 9/1); **Upper diastereomer**; colorless oil; **$[\alpha]_D^{24.9}$** = -161.29 (c 0.0031 g/mL, $CHCl_3$); ^{1}**H-NMR** (600 MHz, $CDCl_3$) δ 7.47 (d, *J* = 6.2 Hz, 2H), 7.33-7.38 (m, 3H), 5.29 (s, 1H), 5.02 (d, *J* = 3.4 Hz, 1H), 4.37 (q, *J* = 4.1 Hz, 1H), 3.76-3.80 (m, 1H), 3.72 (s, 3H), 3.56-3.59 (m, 1H), 2.29-2.33 (m, 1H), 1.93-1.99 (m, 2H), 1.44-1.48 (m, 1H); ^{13}**C-NMR** (150 MHz, $CDCl_3$) δ 171.15, 136.22, 128.80, 128.72, 127.34, 100.06, 76.13, 62.33, 52.49, 30.15, 28.28, 23.64; **HRMS** (ESI) calculated for $C_{14}H_{17}INaO_4$ $[M+Na]^+$: 399.0064, found 399.0061; **IR** (KBr): 3032, 2950, 2880, 2851, 1750, 1435, 1211, 1066, 1052, 697 cm^{-1}. **Enantiomeric ratio**: 88:12, determined by HPLC (Daicel Chiralpak IBN-5, *n*-hexane / isopropanol = 99/1, flow rate 1.0 mL/min, T = 25 ºC, 270 nm): t_{maj} =

10.29 min, t_{min} = 8.33 min. **Lower diastereomer**; colorless oil; **$[\alpha]_D^{24.8}$** = -298.54 (c 0.0041 g/mL, $CHCl_3$); ^{1}**H-NMR** (600 MHz, $CDCl_3$) δ 7.48 (dd, J = 7.9, 1.7 Hz, 2H), 7.35-7.39 (m, 3H), 5.21 (s, 1H), 4.64 (d, J = 4.8 Hz, 1H), 4.21-4.24 (m, 1H), 4.02-4.06 (m, 1H), 3.70 (s, 3H), 3.56-3.60 (m, 1H), 2.40-2.44 (m, 1H), 1.96-1.99 (m, 1H), 1.81-1.88 (m, 1H), 1.56-1.62 (m, 1H); ^{13}**C-NMR** (150 MHz, $CDCl_3$) δ 170.79, 135.41, 129.07, 128.80, 127.79, 100.19, 77.44, 63.34, 52.52, 31.74, 28.30, 24.78; **HRMS** (ESI) calculated for $C_{14}H_{17}INaO_4$ $[M+Na]^+$: 399.0064, found 399.0055; **IR** (KBr): 3030, 2950, 2929, 2850, 1757, 1454, 1209, 1129, 1067, 698 cm^{-1}. **Enantiomeric ratio**: 84:16, determined by HPLC (Daicel Chiralpak IBN-5, *n*-hexane / isopropanol = 99/1, flow rate 1.0 mL/min, T = 25 ºC, 270 nm): t_{maj} = 10.42 min, t_{min} = 11.38 min. The configuration of these two diastereomers **6n** is difficult to be assigned by X-ray crystallographic analysis after recrystallization as they are oily not solids.

(2*S*,3*R*)-2-(1,1-Diphenylethoxy)-3-iodotetrahydro-2*H*-pyran 6o: yield: 91%; (Silica gel column

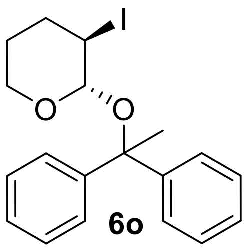
6o

chromatography eluent, *n*-hexane/ethyl acetate = 15/1); colorless oil; **$[\alpha]_D^{25}$** = -184.33 (c 0.003 g/mL, $CHCl_3$); ^{1}**H-NMR** (600 MHz, $CDCl_3$) δ 7.40-7.41 (m, 2H), 7.33-7.37 (m, 4H), 7.28-7.31 (m, 3H), 7.22 (tt, J = 7.3, 1.82 Hz, 1H), 4.68 (d, J = 3.4 Hz, 1H), 4.18-4.20 (m, 1H), 4.10-4.14 (m, 1H), 3.52-3.55 (m, 1H), 2.44-2.49 (m, 1H), 1.90-2.00 (m, 5H), 1.54-1.58 (m, 1H); ^{13}**C-NMR** (150 MHz, $CDCl_3$) δ 148.20, 144.39, 128.23, 127.99, 127.92, 127.73, 126.74, 126.12, 96.98, 82.65, 62.19, 30.93, 30.87, 28.02, 24.11; **HRMS** (ESI) calculated for $C_{19}H_{21}INaO_2$ $[M+Na]^+$: 431.0478, found 431.0475; **IR** (KBr): 3058, 3024, 2945, 2875, 1492, 1445, 1122, 1064, 989, 698 cm^{-1}. **Enantiomeric ratio**: 92:8, determined by HPLC (Daicel Chiralpak OJ-H, *n*-hexane / ethanol = 99/1, flow rate 1.0 mL/min, T = 25 ºC, 210 nm): t_{maj} = 6.55 min, t_{min} = 8.56 min.

(2*S*,3*R*)-3-Iodo-2-((2-phenylpropan-2-yl)oxy)tetrahydro-2*H*-pyran 6p: yield: 99%; (Silica gel

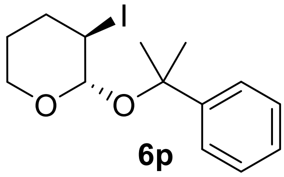
6p

column chromatography eluent, *n*-hexane/ethyl acetate = 10/1); colorless oil; **$[\alpha]_D^{25.5}$** = -303.94 (c 0.0071 g/mL, $CHCl_3$); ^{1}**H-NMR** (600 MHz, $CDCl_3$) δ 7.50 (dd, J = 8.2, 1.4 Hz, 2H), 7.34 (ddd, J = 8.3, 7.6, 1.4 Hz, 2H), 7.26 (ddd, J = 7.6, 6.9, 1.4 Hz, 1H), 4.71 (d, J = 4.8 Hz, 1H), 4.14 (dt, J = 8.2, 3.8 Hz, 1H), 4.02-4.06 (m, 1H), 3.49-3.53 (m, 1H), 2.38-2.43 (m, 1H), 1.96-2.01 (m, 1H), 1.77-1.82 (m, 1H), 1.66 (s, 3H), 1.58 (s, 3H), 1.51-1.57 (m, 1H); ^{13}**C-NMR** (150 MHz, $CDCl_3$) δ 146.07, 128.19, 127.26, 125.96, 97.45, 78.74, 62.76, 32.22, 31.46, 30.60, 27.44, 25.12; **HRMS** (ESI) calculated for

$C_{14}H_{19}INaO_2$ $[M+Na]^+$: 369.0322, found 369.0320; **IR** (KBr): 2977, 2944, 2875, 2849, 1265, 1117, 1064, 1015, 764, 698 cm^{-1}. **Enantiomeric ratio**: 89:11, determined by HPLC (Daicel Chiralpak IBN-5, *n*-hexane / isopropanol = 30/1, flow rate 0.4 mL/min, T = 25 ºC, 262 nm): t_{maj} = 11.92 min, t_{min} = 12.77 min.

4-(((2*S*,3*R*)-3-Iodotetrahydro-2*H*-pyran-2-yl)oxy)-4-methylcyclohexa-2,5-dien-1-one 6q: yield: 98%; (Silica gel column chromatography eluent, *n*-hexane/ethyl acetate = 7/1); colorless oil; $[\alpha]_D^{24.8}$ = -85.93 (c 0.0027 g/mL, $CHCl_3$); ^{1}**H-NMR** (400 MHz, $CDCl_3$) δ 7.02 (dd, *J* = 10.1, 3.2 Hz, 1H), 6.97 (dd, *J* = 10.1, 3.2 Hz, 1H), 6.32 (dd, *J* = 10.1, 1.8 Hz, 1H), 6.15 (dd, *J* = 10.1, 1.8 Hz, 1H), 4.37 (d, *J* = 7.8 Hz, 1H), 3.92-4.02 (m, 2H), 3.40-3.46 (m, 1H), 2.43-2.50 (m, 1H), 2.00-2.10 (m, 1H), 1.60-1.70 (m, 1H), 1.50-1.56 (m, 4H); ^{13}**C-NMR** (100 MHz, $CDCl_3$) δ 185.62, 152.01, 150.31, 130.04, 127.39, 100.66, 73.64, 65.55, 35.51, 29.43, 27.47, 26.41; **HRMS** (ESI) calculated for $C_{12}H_{15}INaO_3$ $[M+Na]^+$: 356.9958, found 356.9959; **IR** (KBr): 2979, 2954, 2924, 2850, 1665, 1627, 1381, 1053, 863, 691 cm^{-1}. **Enantiomeric ratio**: 86:14, determined by HPLC (Daicel Chiralpak OJ-H, *n*-hexane / ethanol = 99/1, flow rate 1.0 mL/min, T = 25 ºC, 265 nm): t_{maj} = 17.60 min, t_{min} = 20.11 min.

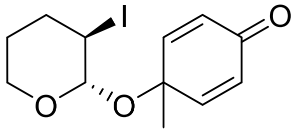

6q

4-(((2*S*,3*R*)-3-Iodotetrahydro-2*H*-pyran-2-yl)oxy)-3,4-dimethylcyclohexa-2,5-dien-1-one 6r: yield: 92%; two diastereomers that can be separated easily *via* (Silica gel column chromatography eluent, *n*-hexane/ethyl acetate = 10/1); **Upper diastereomer**; colorless oil; $[\alpha]_D^{24.5}$ = -63.98 (c .0042 g/mL, $CHCl_3$); ^{1}**H-NMR** (600 MHz, $CDCl_3$) δ 6.99 (d, *J* = 10.3 Hz, 1H), 6.30 (dd, *J* = 10.3, 2.1 Hz, 1H), 6.02 (d, *J* = 1.4 Hz, 1H), 4.42 (d, *J* = 7.6 Hz, 1H), 3.91-3.96 (m, 2H), 3.35-3.39 (m, 1H), 2.43-2.47 (m, 1H), 2.01-2.08 (m, 4H), 1.58-1.66 (m, 1H), 1.47-1.52 (m, 4H); ^{13}**C-NMR** (150 MHz, $CDCl_3$) δ 185.90, 162.00, 150.51, 129.91, 126.75, 100.92, 75.21, 65.77, 35.60, 30.05, 27.51, 25.43, 18.95; **HRMS** (ESI) calculated for $C_{13}H_{17}INaO_3$ $[M+Na]^+$: 371.0115, found 371.0117; **IR** (KBr): 2987, 2943, 2863, 1668, 1632, 1291, 1058, 1026, 896, 692 cm^{-1}. **Enantiomeric ratio**: 71:29, determined by HPLC (Daicel Chiralpak IBN-5, *n*-hexane / isopropanol = 99/1, flow rate 1.0 mL/min, T = 25 ºC, 255 nm): t_{maj} = 15.73 min, t_{min} = 21.46 min. **Lower diastereomer**; colorless oil; $[\alpha]_D^{24.9}$ = -32.54 (c 0.0067 g/mL, $CHCl_3$); ^{1}**H-NMR** (600 MHz, $CDCl_3$) δ 6.92 (d, *J* = 10.3 Hz, 1H), 6.17 (d, *J* = 2.1 Hz, 1H), 6.11 (dd, *J* = 10.3, 2.1 Hz, 1H), 4.38 (d, *J* = 5.5 Hz, 1H), 4.02-4.05 (m, 1H), 3.96-4.00 (m, 1H), 3.45-3.49 (m, 1H), 2.35-2.40 (m, 1H), 2.10 (s, 3H), 1.95-2.01 (m, 1H), 1.72-1.78 (m, 1H),

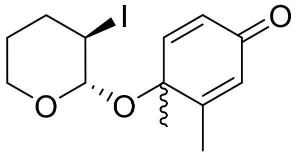

6r

1.53-1.59 (m, 1H), 1.46 (s, 3H); ^{13}**C-NMR** (150 MHz, $CDCl_3$) δ 186.06, 158.22, 152.60, 129.45, 126.58, 99.22, 75.18, 63.36, 32.80, 28.85, 25.78, 25.37, 19.08; **HRMS** (ESI) calculated for $C_{13}H_{17}INaO_3$ $[M+Na]^+$: 371.0115, found 371.0117; **IR** (KBr): 2970, 2950, 2858, 1671, 1634, 1298, 1062, 1022, 887, 693 cm^{-1}. **Enantiomeric ratio**: 78:22, determined by HPLC (Daicel Chiralpak IBN-5, *n*-hexane / isopropanol = 99/1, flow rate 1.0 mL/min, T = 25 ºC, 260 nm): t_{maj} = 40.16 min, t_{min} = 21.68 min.

4-(((2*S*,3*R*)-3-Iodotetrahydro-2*H*-pyran-2-yl)oxy)-3,4,5-trimethylcyclohexa-2,5-dien-1-one 6s:

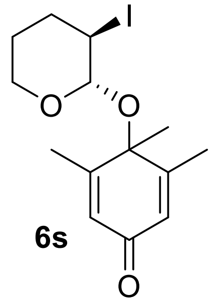

6s

yield: 95%; (Silica gel column chromatography eluent, *n*-hexane/ethyl acetate = 8/1); colorless oil; $[\alpha]_D^{24.8}$ = -84.29 (c 0.0021 g/mL, $CHCl_3$); ^{1}**H-NMR** (400 MHz, $CDCl_3$) δ 6.18-6.19 (m, 1H), 5.98-5.99 (m, 1H), 4.32 (d, J = 6.0 Hz, 1H), 3.97-4.02 (m, 1H), 3.87-3.92 (m, 1H), 3.34-3.40 (m, 1H), 2.34-2.42 (m, 1H), 2.15 (d, J = 1.4 Hz, 3H), 2.05 (d, J = 1.4 Hz, 3H), 1.96-2.03 (m, 1H), 1.51-1.66 (m, 2H), 1.45 (s, 3H); ^{13}**C-NMR** (100 MHz, $CDCl_3$) δ 185.69, 162.69, 157.70, 130.23, 126.19, 100.02, 77.10, 64.53, 33.89, 29.17, 26.28, 24.62, 19.17, 19.14; **HRMS** (ESI) calculated for $C_{14}H_{19}INaO_3$ $[M+Na]^+$: 385.0271, found 385.0273; **IR** (KBr): 2948, 2925, 2854, 1672, 1633, 1378, 1303, 1061, 894, 697 cm^{-1}. **Enantiomeric ratio**: 90:10, determined by HPLC (Daicel Chiralpak IBN-5, *n*-hexane / isopropanol = 99/1, flow rate 1.0 mL/min, T = 25 ºC, 245 nm): t_{maj} = 27.13 min, t_{min} = 23.23 min.

5. Optimization of iodocyclization reaction conditions

Table S1. Screening of different catalysts **3** and catalyst loadings. [a]

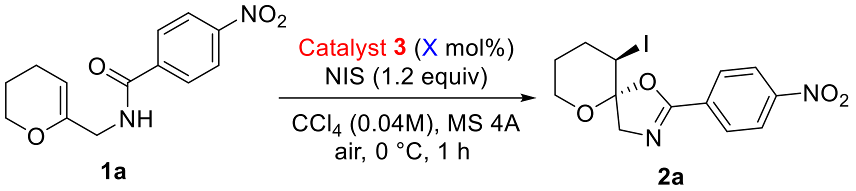

entry	Catalyst (X mol%)	yield (%)[b]	dr[d]	er[d]
**1**	Λ-(*S*,*S*)-**3a** (10 mol%)	91	>20:1	50:50
**2**	Λ-(*S*,*S*)-**3b** (10 mol%)	96	>20:1	51:49
**3**	Λ-(*S*,*S*)-**3c** (10 mol%)	97	>20:1	52:48
**4**	Δ-(*S*,*S*)-**3b** (10 mol%)	93	>20:1	51:49
**5**	Δ-(*S*,*S*)-**3c** (10 mol%)	90	>20:1	50:50
**6**	Λ-(*S*,*S*)-**3d** (10 mol%)	98	>20:1	53:47
**7**	Λ-(*S*,*R*,*S*,*R*)-**3e** (10 mol%)	92	>20:1	65:35
**8**	Λ-(*S*,*S*,*S*,*S*)-**3f** (10 mol%)	97	>20:1	76:24
**9**	Δ-(*S*,*S*,*S*,*S*)-**3f** (10 mol%)	87	>20:1	55:45
**10**	Λ-(*S*,*S*,*S*,*S*)-**3g** (10 mol%)	96 (93)[c]	>20:1	79:21
**11**	Λ-(*S*,*S*,*S*,*S*)-**3g** (5 mol%)	95	>20:1	73:27
**12**	Λ-(*S*,*S*,*S*,*S*)-**3g** (20 mol%)	99	>20:1	75:25

[a]The reaction of **1a** (0.04 mmol), NIS (0.048 mmol, 1.2 equiv), **3** (0.004 mmol) was conducted in the solvent (1.0 mL). [b]Yields were determined *via* ^{1}H NMR spectroscopy using 1,3,5-trimethoxybenzene as an internal standard. [c]Isolated yield. [d]Determined by HPLC.

Table S2. Screening of different solvents, concentrations, and temperatures. [a]

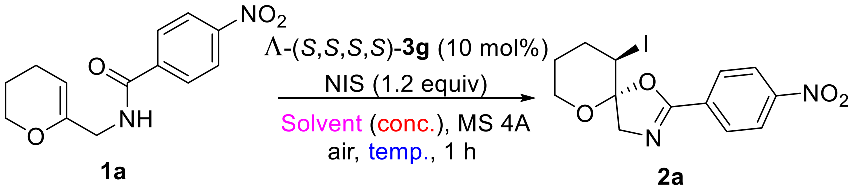

entry	Solvent (conc.)	Temp. (X °C)	yield (%)[b]	dr[c]	er[c]
**1**	$CHCl_3$ (0.04 M)	0	45	>20:1	50:50
**2**	TFE (0.04 M)	0	89	>20:1	51:49
**3**	Toluene (0.04 M)	0	91	>20:1	53:47
**4**	Cyclohexane (0.04 M)	0	54	>20:1	50:50
**5**	DCM (0.04 M)	0	98	>20:1	53:47
**6**	EtOH (0.04 M)	0	84	>20:1	51:49

**7**	1,4-Dioxane (0.04 M)	0	93	>20:1	52:48
**8**	Tetrachloroethane (0.04 M)	0	99	>20:1	55:45
**9**	C_6H_5F (0.04 M)	0	97	>20:1	51:49
**10**	C_6H_5Cl (0.04 M)	0	99	>20:1	50:50
**11**	C_6H_5I (0.04 M)	0	96	>20:1	52:48
**12**	CCl_4 (0.04 M)	0	96	>20:1	79:21
**13**	CCl_4 (0.02 M)	0	92	>20:1	65:35
**14**	CCl_4 (0.06 M)	0	95	>20:1	80:20
**15**	CCl_4 (0.08 M)	0	97	>20:1	84:16
**16**	CCl_4 (0.1 M)	0	98	>20:1	78:22
**17**	CCl_4 (0.08 M)	25	99	>20:1	50:50
**18**	CCl_4 (0.08 M)	-20	95	>20:1	90:10
**19**	$CCl_4/CHCl_3$ 9/1 (0.08 M)	-30	94	>20:1	70:30
**20**	C_6H_5I (0.08 M)	-25	99	>20:1	53:47
**21**	C_6H_5F (0.08 M)	-40	93	>20:1	54:46
**22**	C_6H_5Cl (0.08 M)	-40	96	>20:1	50:50
**23**	Toluene (0.08 M)	-78	82	>20:1	58:42
**24**	DCM (0.08 M)	-78	90	>20:1	52:48

[a]The reaction of **1a** (0.04 mmol), NIS (0.048 mmol, 1.2 equiv), Λ-**3g** (0.004 mmol) was conducted in the solvent (X mL). [b]Yields were determined *via* ^{1}H NMR spectroscopy using 1,3,5-trimethoxybenzene as an internal standard. [c]Determined by HPLC.

Table S3. Screening of different iodinating agents, and additives. [a]

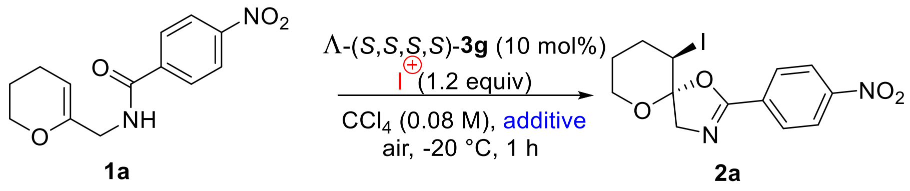

entry	I^+	additive	yield (%)[b]	dr[c]	er[c]
**1**	NIS	MS 4A	95	>20:1	90:10
**2**	NIP	MS 4A	89	>20:1	59:41
**3**	DIH	MS 4A	99	>20:1	78:22
**4**	NIS	MS 3A	98	>20:1	87:13
**5**	NIS	---	91	>20:1	69:31

[a]The reaction of **1a** (0.04 mmol), Iodinating agent (0.048 mmol, 1.2 equiv), Λ-**3g** (0.004 mmol) was conducted in the CCl_4 (0.5 mL). [b]Yields were determined *via* ^{1}H NMR spectroscopy using 1,3,5-trimethoxybenzene as an internal standard. [c]Determined by HPLC.

6. Optimization of iodoacetalization reaction conditions

Table S4. Screening of different catalysts **3**, catalyst loadings, temperatures, and additives. [a]

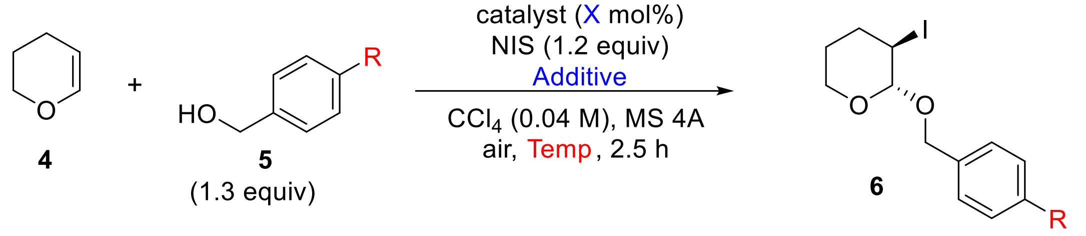

entry	Catalyst (X mol%)	R	Temp. (°C)	Additives	yield (%)[b]	dr[c]	er[c]
**1**	--	H	0 (12 h)	--	70	>20:1	50:50
**2**	Λ-(*S*,*S*)-**3a** (10 mol%)	H	0	--	82	>20:1	50:50
**3**	Λ-(*S*,*S*)-**3b** (10 mol%)	H	0	--	88	>20:1	65:35
**4**	Δ-(*S*,*S*)-**3b** (10 mol%)	H	0	--	82	>20:1	60:40
**5**	Λ-(*S*,*S*)-**3d** (10 mol%)	H	0	--	84	>20:1	62:38
**6**	Λ-(*S*,*S*)-**3d** (10 mol%)	H	-20	--	75	>20:1	56:44
**7**	Λ-(*S*,*S*)-**3d** (10 mol%)	H	20	--	86	1:1	61:39
**8**	Λ-(*S*,*S*)-**3d** (50 mol%)	H	0	--	93	>20:1	57:43
**9**	Λ-(*S*,*R*,*S*,*R*)-**3e** (10 mol%)	H	0	--	95	>20:1	64:36
**10**	Λ-(*S*,*S*)-**3b** (10 mol%)	NO_2	0	--	83	>20:1	66:34
**11**	Λ-(*S*,*S*)-**3d** (10 mol%)	NO_2	0	--	93	>20:1	59:41
**12**	Λ-(*S*,*R*,*S*,*R*)-**3e** (10 mol%)	NO_2	0	--	92	>20:1	73:27
**13**	Λ-(*S*,*R*,*S*,*R*)-**3e** (10 mol%)	NO_2	0	$NaHCO_3$ (0.2 eq)	80	>20:1	71:29
**14**	Λ-(*S*,*R*,*S*,*R*)-**3e** (10 mol%)	NO_2	0	$NaHCO_3$ (1.0 eq)	84	>20:1	69:31
**15**	Λ-(*S*,*R*,*S*,*R*)-**3e** (10 mol%)	NO_2	0	TEA (1.0 eq)	41	>20:1	50:50
**16**	Λ-(*S*,*S*,*S*,*S*)-**3f** (10 mol%)	NO_2	0	--	93	>20:1	84:16
**17**	Δ-(*S*,*S*,*S*,*S*)-**3f** (10 mol%)	NO_2	0	--	91	>20:1	75:25
**18**	Λ-(*S*,*S*,*S*,*S*)-**3g** (10 mol%)	NO_2	0	--	96	>20:1	85:15

[a]The reaction of **4** (0.04 mmol), NIS (0.048 mmol, 1.2 equiv), **3** (0.004 mmol), and alcohol **5** (0.052 mmol) was conducted in the CCl_4 (1.0 mL). [b]Yields were determined *via* ^1H NMR spectroscopy using 1,3,5-trimethoxybenzene as an internal standard. [c]Determined by HPLC.

Table S5. Screening of different solvents and iodinating agents. [a]

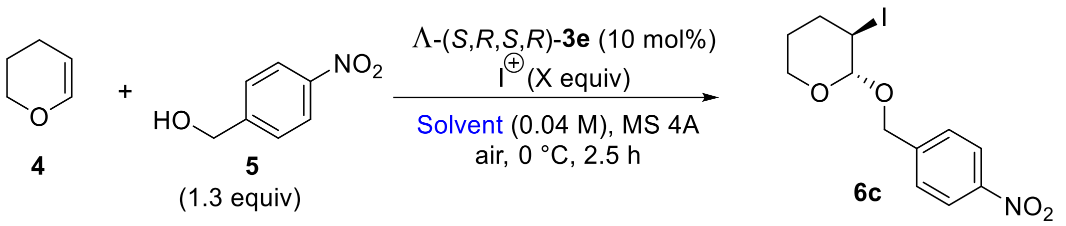

entry	Solvent	I^+ (X eq.)	yield (%)[b]	dr[c]	er[c]
**1**	*n*-hexane	NIS (1.2 eq.)	79	>20:1	56:44
**2**	Cyclohexane	NIS (1.2 eq.)	59	>20:1	58:42
**3**	MeCN	NIS (1.2 eq.)	81	>20:1	50:50
**4**	CH_2Cl_2	NIS (1.2 eq.)	96	>20:1	50:50
**5**	THF	NIS (1.2 eq.)	64	>20:1	50:50
**6**	Toluene	NIS (1.2 eq.)	76	>20:1	55:45
**7**	CCl_4	NIS (1.2 eq.)	92	>20:1	73:27
**8**	CCl4/cyclohexane (1/1)	NIS (1.2 eq.)	88	>20:1	73:27
**9**	CCl4/cyclohexane (1/3)	NIS (1.2 eq.)	85	>20:1	73:27
**10**	CCl_4	DIH (1.2 eq.)	90	>20:1	73:27
**11**	CCl_4	DIH (1.8 eq.)	98	>20:1	65:35
**12**	CCl_4	DIH (0.6 eq.)	48	>20:1	69:31
**13**	CCl_4	NIS (2.4 eq.)	95	>20:1	73:27
**14**	CCl_4	NIP (1.2 eq.)	88	>20:1	67:33
**15**	CCl_4	NIA (1.2 eq.)	80	>20:1	63:37
**16**[d]	CCl_4	NIS (1.2 eq.)	85	>20:1	73:27

[a]The reaction of **4** (0.04 mmol), I^+ (X equiv), Λ-**3e** (0.004 mmol), and alcohol **5c** (0.052 mmol) was conducted in solvent (1.0 mL). [b]Yields were determined *via* ^1H NMR spectroscopy using 1,3,5-trimethoxybenzene as an internal standard. [c]Determined by HPLC. [d]With phthalimide (15 mol%)

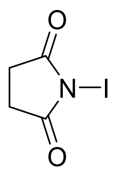

NIS
N-Iodosuccinimide

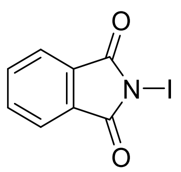

NIP
N-Iodophthalimide

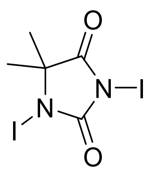

DIH
Diiodohydantoin

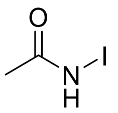

NIA
N-Iodoacetamide

7. Extended substrate scope of iodoacetalization reaction

7.1. Scheme S1: Other substrates as mixture of four isomers

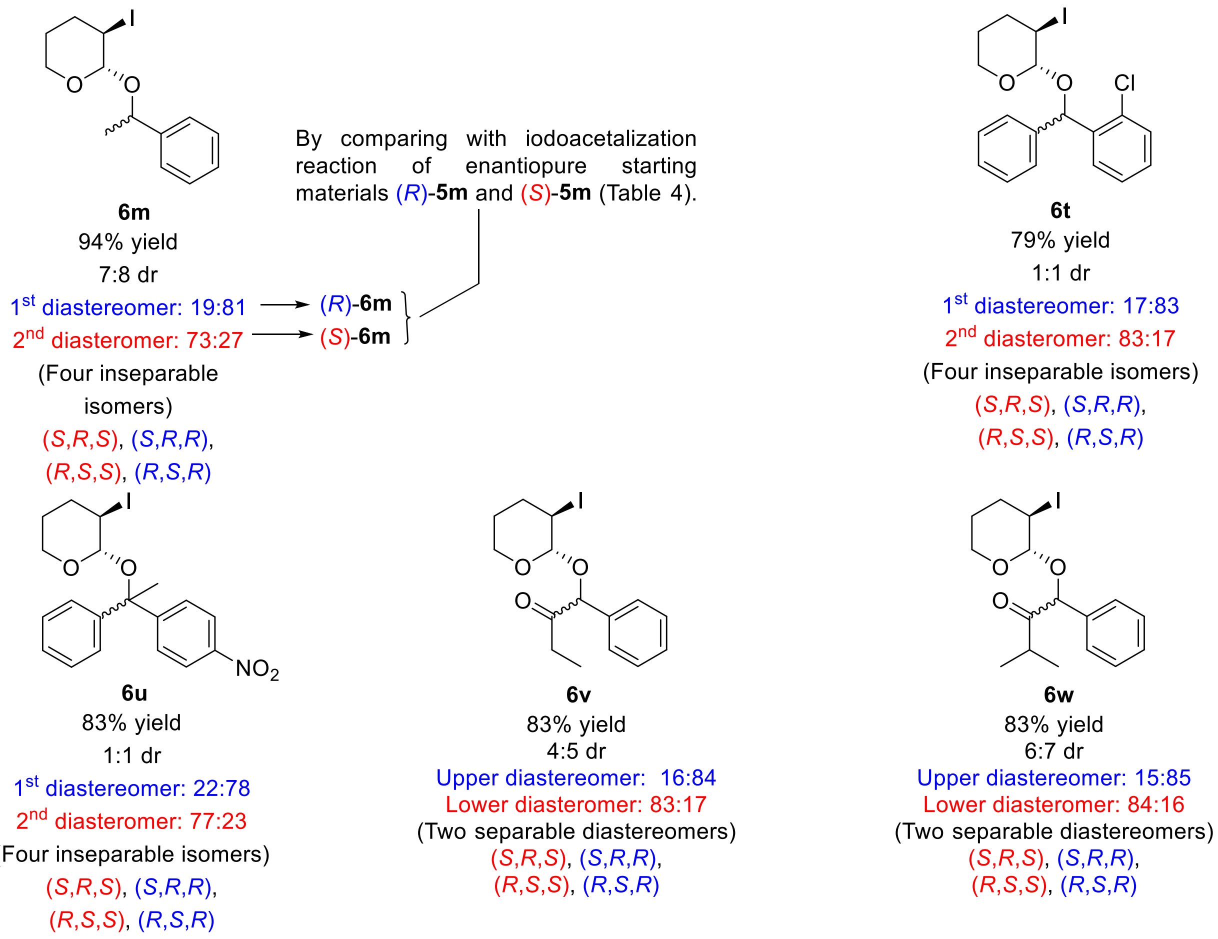

- *__6m__, __6t__, and __6u__ can't be separated on silica coulmn, but __6v__ and __6w__ successfully separated.*
- *The configuration of __6m__ was assigned roughly via comparing the same products upon using known enantiopure alcohols (R)-__5m__ and (S)-__5m__ (Table 4).*
- *The absolute configuration of all these isomers can not be assign accurately due to their oily nature (hard to be recrystallized).*

7.2. Scheme S2: Trying other nucleophiles (different from alcohols) in iodoacetalization reaction

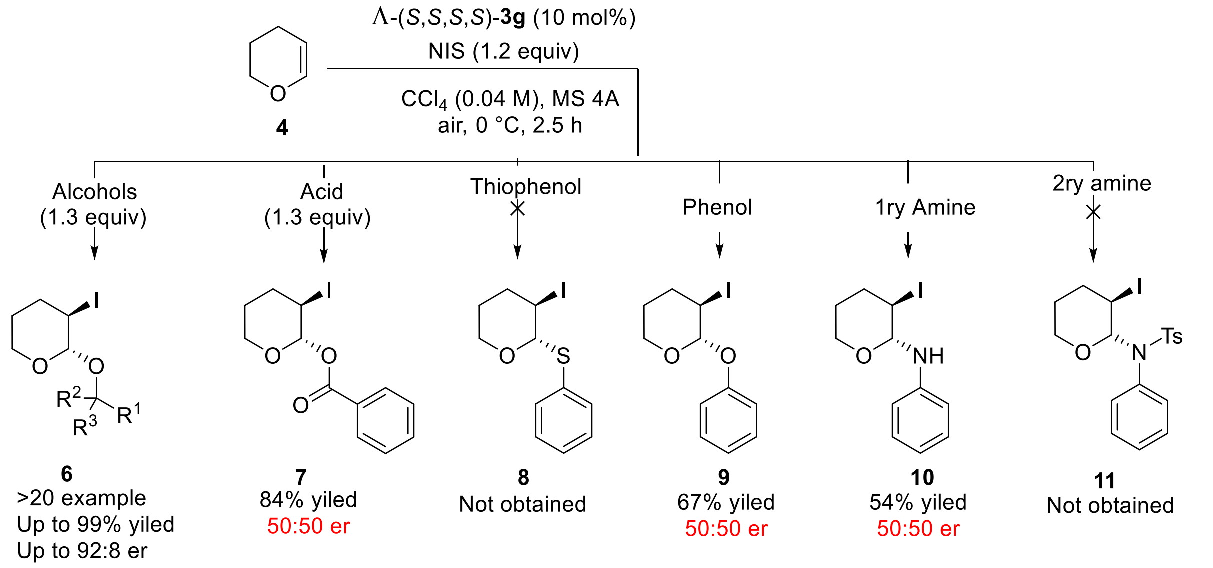

8. Unsuccessful cobalt(III)-catalyzed enantioselective transformations

8.1. Scheme S3: Chlorocyclization and Bromocyclization reactions of **1e**

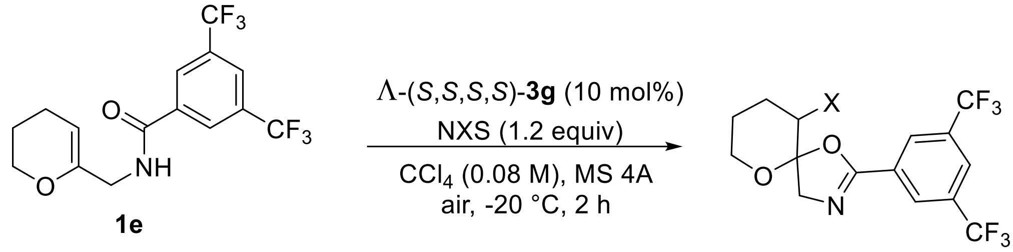

2e' (X=Br): 91% yield, 50:50 er
2e'' (X=Cl): 96% yield, 50:50 er

8.2. Scheme S4: Bromoacetalization reaction of **4**

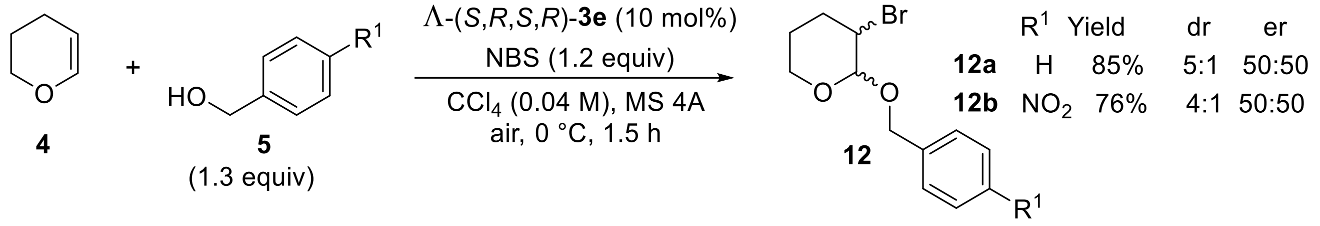

8.3. Scheme S5: Bromoenocyclization reaction of **13**

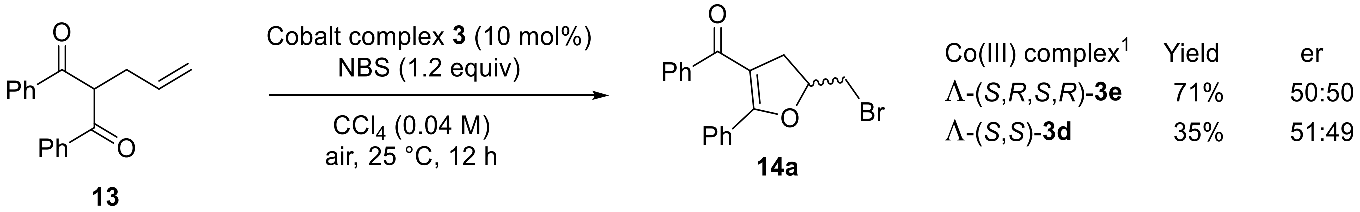

8.4. Scheme S6: Iodocyclization reaction for different substrate **15**

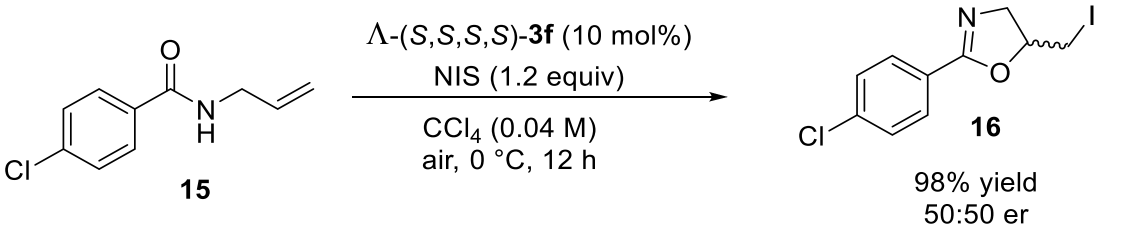

9. Suboptimal cobalt(III)-catalyzed enantioselective transformations

9.1. Scheme S7: Iodoenocyclization reaction of 13

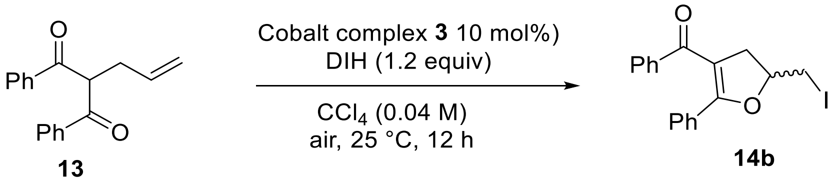

Co(III) complex[1]	Yield	er
Λ-(*S*,*R*,*S*,*R*)-**3e**	42%	50:50
Λ-(*S*,*S*)-**3d**	23%	55:45

9.2. Scheme S8: Iodocyclization reaction of 17

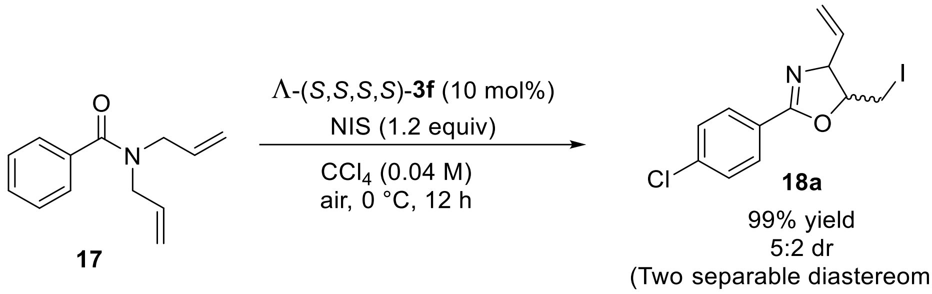

99% yield
5:2 dr
(Two separable diastereomers)
Upper diastereomer: 62:38
Lower diasteromer: 30:70

9.3. Scheme S9: Bromocyclization reaction of 17

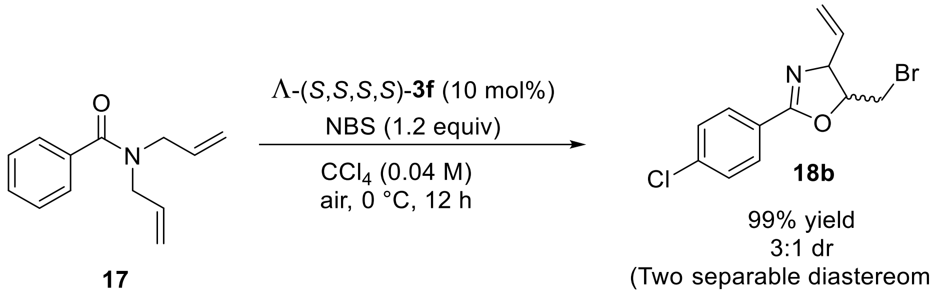

99% yield
3:1 dr
(Two separable diastereomers)
Upper diastereomer: 63:37
Lower diasteromer: 24:76

10. Preliminary DFT calculations

To obtain an insight on the role of the bulkiness and configuration of the amino acid's side chain on the diastereoselective complexation of octahedral cobalt complexes **3**, we conducted a preliminary DFT calculation study to find out the energy gap between Λ and Δ *mer* isomers in each case. Using gaussian 16 package at B3LYP function (Basis set = Wachters-Hay basis set for Co and D95** for other atoms), we observed that less bulky side chains (H or Me) in complexes **3h**-**3j** resulted in very low energy gaps between the two *mer* isomers Λ and Δ; which can explain the formation of both isomer upon complexation in almost 1:1 ratio. Bulkier side chains (*iso*-propyl, *sec*-butyl, or *tert*-butyl) broaden the energy gap between the two isomers which affects on the ratio of the formed complexes. Using tridentate ligands with *S*- configuration affording Λ-(*S*,*S*)-**3** as the major product, while using ligands with *R*-configuration affording Δ-(*R*,*R*)-**3** as the major. These experimental data are in a good agreement and can be explained with our calculations that show that Λ-(*S*,*S*)-**3** complexes are more stable compared to the corresponding Δ-(*S*,*S*)-**3**. The same can be also claimed for Δ-(*R*,*R*)-**3** complexes as well. Few complexes such as Δ-(*R*,*R*)-**3d**, Λ-(*S*,*S*)-**3d**, and Λ-(*S*,*R*,*S*,*R*)-**3e** were obtained as a pure *mer* isomer due to the wide energy gap between the two Λ and Δ isomers.

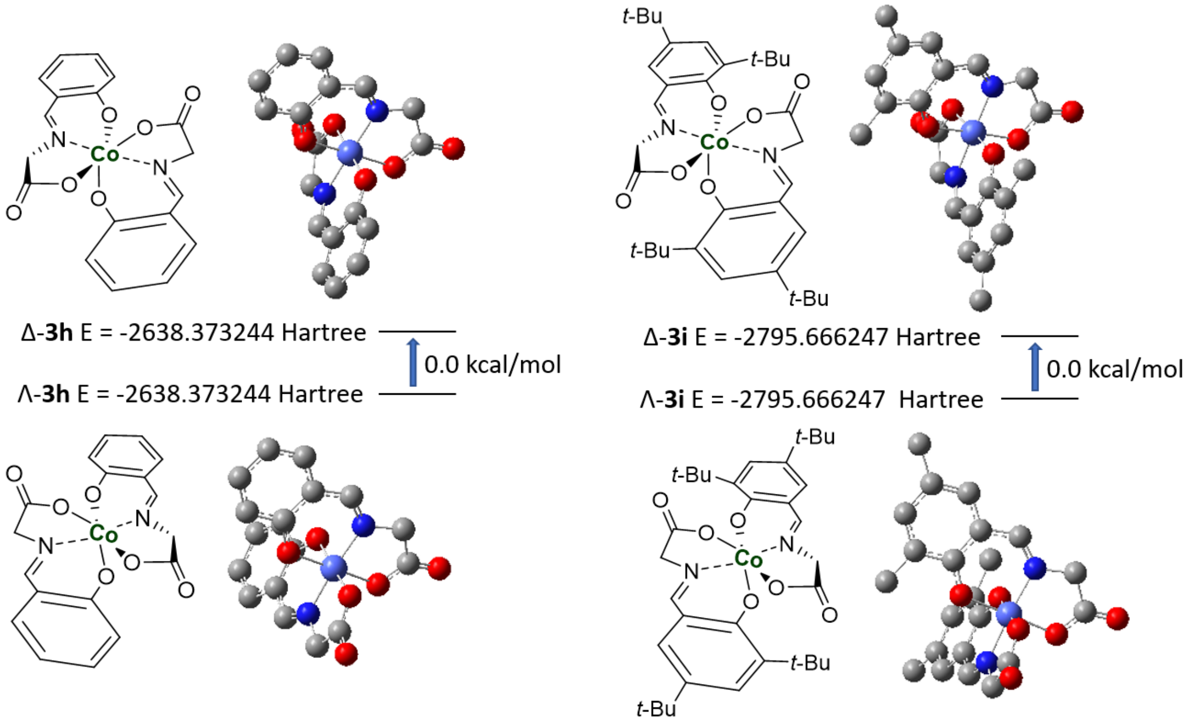

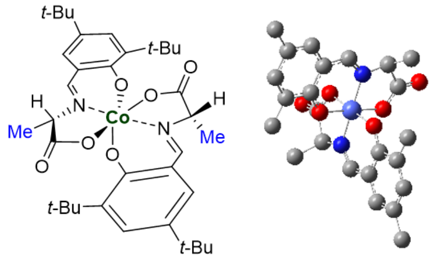

Δ-(*S*,*S*)-**3j** E = -2874.309642 Hartree

1.2 kcal/mol

Λ-(*S*,*S*)-**3j** E = -2874.311551 Hartree

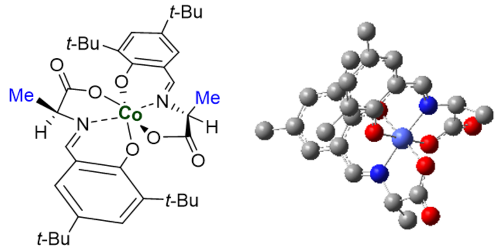

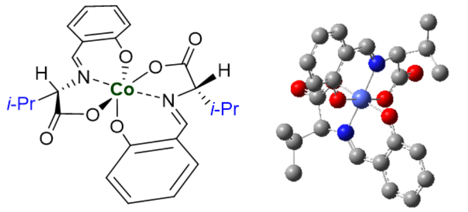

Δ-(*S*,*S*)-**3a** E = -2874.286906 Hartree

1.6 kcal/mol

Λ-(*S*,*S*)-**3a** E = -2874.289479 Hartree

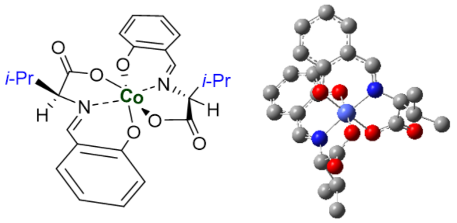

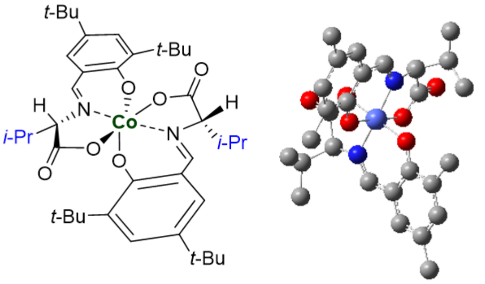

Δ-(*S*,*S*)-**3c** E = -3031.577803 Hartree

2.9 kcal/mol

Λ-(*S*,*S*)-**3c** E = -3031.582458 Hartree

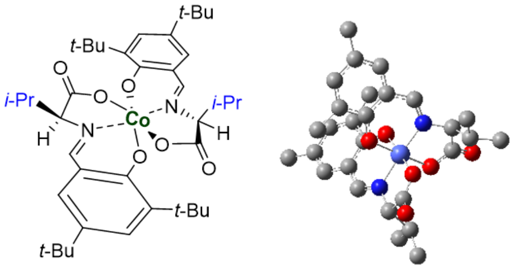

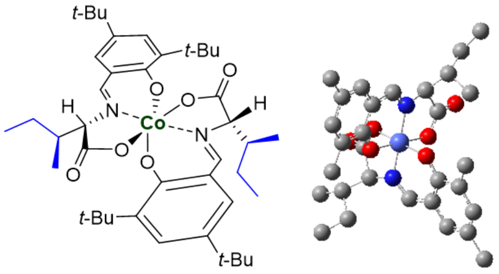

Δ-(*S*,*S*,*S*,*S*)-**3f** E = -3110.214844 Hartree

2.7 kcal/mol

Λ- (*S*,*S*,*S*,*S*)-**3f** E = -3110.219128 Hartree

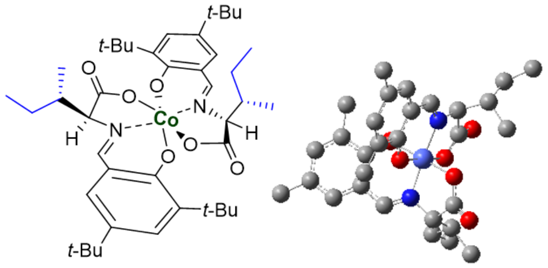

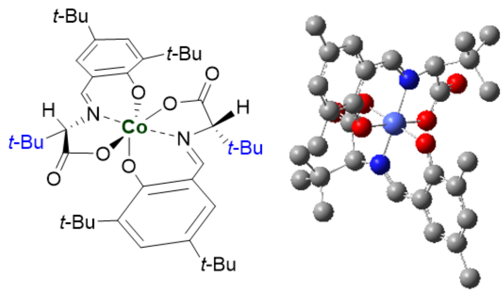

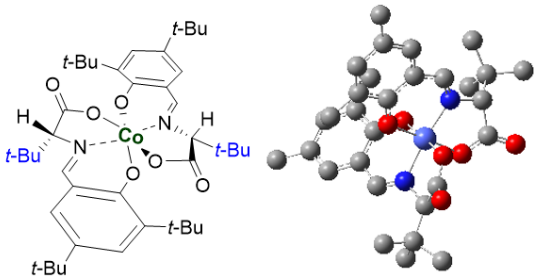

Δ-(*S*,*S*)-**3d** E = -3110.207509 Hartree

3.5 kcal/mol

Λ-(*S*,*S*)-**3d** E = -3110.213016 Hartree

Λ-(*R*,*R*)-**3d** E = -3110.201960 Hartree

6.9 kcal/mol

Δ-(*R*,*R*)-**3d** E = -3110.213010 Hartree

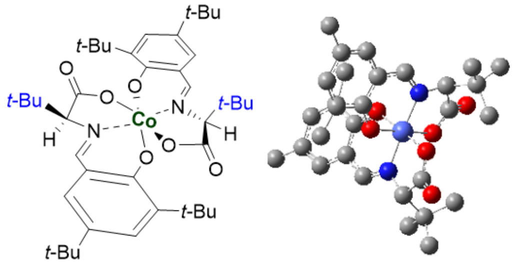

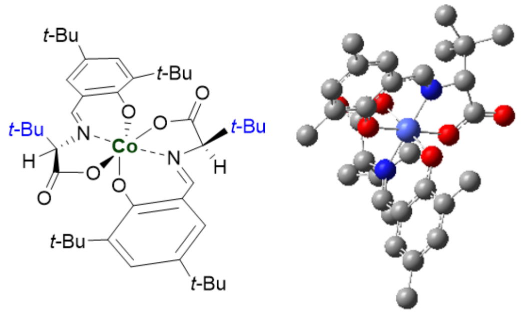

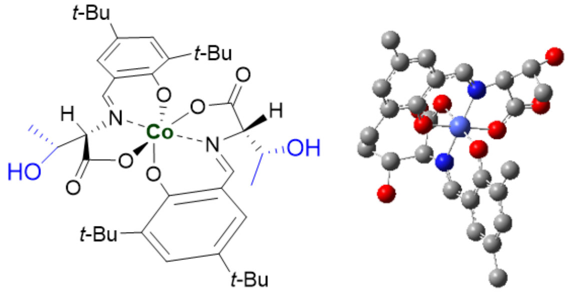

Δ-(*S*,*R*,*S*,*R*)-**3e** E = -3103.429889 Hartree

6.3 kcal/mol

Λ-(*S*,*R*,*S*,*R*)-**3e** E = -3103.439989 Hartree

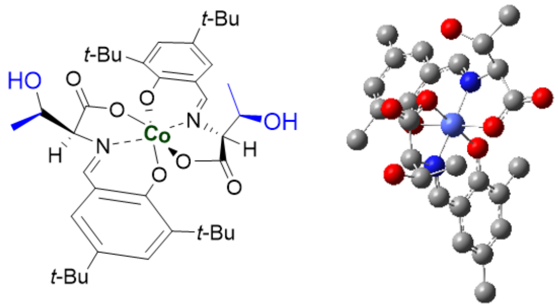

Λ-(*S*,*S*)-**3a** Charge = -1 Multiplicity = 1

Co	-0.211159	0.000055	-0.000043
O	1.169313	-0.376405	1.277983
O	1.169423	0.376079	-1.278063
O	-1.529772	0.382245	-1.328813
N	-0.285279	-1.780344	-0.679026
O	-1.529843	-0.381731	1.328793
N	-0.284677	1.780486	0.67894
C	1.952552	1.407121	-1.229376
O	-2.585044	0.543401	3.086074
C	-1.897636	0.626541	2.065089
C	0.643647	2.662786	0.529398
C	2.713076	3.60789	-0.381528
C	1.952064	-1.407735	1.229329
O	-2.585533	-0.542559	-3.085927
C	3.801924	3.613697	-1.243993
C	-1.456535	-2.010657	-1.523161
C	2.711828	-3.608774	0.381503
C	-1.455809	2.011147	1.523155
C	3.073887	1.452656	-2.12089
C	-2.637548	-2.663475	-0.727226
C	1.78188	-2.539599	0.349557
C	3.966252	2.514995	-2.122698
C	3.965314	-2.51635	2.122745
C	3.800644	-3.614973	1.244007
C	0.642739	-2.662959	-0.529452
C	-2.636654	2.664305	0.727256
C	1.782748	2.539046	-0.349609
C	-3.807831	-2.997829	-1.668965
C	-2.204196	-3.921964	0.04232
C	3.073327	-1.453693	2.120912
C	-1.898017	-0.625916	-2.065029
C	-2.202927	3.922736	-0.042176
C	-3.806882	2.99891	1.668971
H	0.560772	3.601474	1.08905
H	2.552674	4.448249	0.295281
H	-1.198908	-2.665209	-2.368766
H	2.551155	-4.449072	-0.295319
H	-1.197947	2.665652	2.368724
H	4.80919	2.4996	-2.813949
H	4.80822	-2.501268	2.814042
H	0.559538	-3.601641	-1.089067
H	-3.521292	-3.779863	-2.386658
H	-4.125175	-2.123424	-2.241106
H	-4.658432	-3.373822	-1.085941
H	-1.797606	-4.689323	-0.632958
H	-1.456152	-3.696045	0.807011
H	-3.073014	-4.358136	0.550745
H	-1.796192	4.689942	0.633188
H	-1.454877	3.696679	-0.806823
H	-3.071588	4.359169	-0.550646
H	-4.124552	2.124541	2.240986
H	-3.520143	3.780755	2.386789
H	-4.657326	3.375249	1.085939
H	-2.963287	1.923985	-0.011315
H	-2.964013	-1.923019	0.011286
H	3.201773	0.607632	-2.793519
H	4.506416	4.442193	-1.250979
H	3.201474	-0.608732	2.793569
H	4.504846	-4.443715	1.251004

Δ-(*S*,*S*)-**3a** Charge = -1 Multiplicity = 1

Co	-0.00018	-0.00034	0.408901
O	0.107686	-1.3332	-0.96309
O	0.149471	-1.33417	1.757988
O	-0.15112	1.334804	1.756523
N	1.89206	0.274213	0.553038
O	-0.10689	1.330851	-0.96485
N	-1.89257	-0.27453	0.551686
C	-0.96453	-1.80831	2.227243
C	-2.26017	-1.28842	1.551243
C	1.196711	-1.89094	-1.38186
O	1.053552	2.638702	3.135158
C	2.258728	1.288385	1.552639
C	3.643104	-2.27733	-1.42132
C	3.07521	2.507708	1.03141
C	2.523842	-1.56862	-0.91607
C	2.218192	-3.58296	-2.85721
C	3.515707	-3.27548	-2.37855
C	2.773953	-0.52065	0.044661
C	2.357918	3.277721	-0.08869
C	4.519503	2.166977	0.624234
C	1.099366	-2.91607	-2.38058
C	0.962474	1.808442	2.227287
C	-2.77381	0.52103	0.043269
C	-2.52276	1.569026	-0.91719
C	-1.19537	1.890079	-1.38311
C	-1.09711	2.915311	-2.38162
C	-2.21532	3.583466	-2.85794

C	-3.51308	3.277218	-2.37916
C	-3.64137	2.278969	-1.42214
O	-1.05646	-2.63875	3.13487
C	-3.07652	-2.50764	1.029666
C	-2.35849	-3.27822	-0.08957
C	-4.52029	-2.16645	0.621071
H	-2.85295	-0.80802	2.342885
H	2.85096	0.808325	2.344889
H	4.631022	-2.01483	-1.04034
H	2.092551	-4.36022	-3.61139
H	4.387594	-3.8066	-2.75326
H	3.816302	-0.40573	0.355641
H	2.188601	2.642369	-0.96401
H	1.379448	3.637412	0.243915
H	2.958858	4.145289	-0.39241
H	5.03674	1.584445	1.398485
H	5.089253	3.091909	0.466825
H	4.560384	1.603168	-0.31556
H	0.102971	-3.15686	-2.74429
H	-3.81631	0.406734	0.353979
H	-0.10053	3.155148	-2.74545
H	-2.08898	4.360773	-3.61194
H	-4.38447	3.809327	-2.75361
H	-4.62951	2.017382	-1.0411
H	-1.38038	-3.63801	0.243975
H	-2.95939	-4.14575	-0.39348
H	-2.18829	-2.64322	-0.96497
H	-5.03811	-1.58377	1.394826
H	-4.56005	-1.60257	-0.31873
H	-5.09019	-3.09119	0.463058
H	-3.1243	-3.15588	1.913936
H	3.121977	3.15621	1.915545

Λ-(*S*,*S*)-**3c** Charge = -1 Multiplicity = 1

Co	-0.657176	-0.000038	0.00002
O	0.721895	-0.519806	1.226589
O	0.721883	0.519794	-1.226518
O	-1.974959	0.526418	-1.280696
N	-0.730296	-1.695244	-0.869421
O	-1.974855	-0.526605	1.280806
N	-0.730451	1.695181	0.869417
C	1.499689	1.547378	-1.071553
O	-3.013375	0.19716	3.139764
C	-2.33555	0.393572	2.127601
C	0.193507	2.593913	0.809303
C	2.252784	3.650563	0.000373
C	1.499835	-1.547273	1.071552
O	-3.01332	-0.197365	-3.139739
C	3.337233	3.76279	-0.855948
C	-1.898597	-1.830033	-1.737796
C	2.253141	-3.650356	-0.000428
C	-1.89876	1.82988	1.737802
C	2.618141	1.681135	-1.972441
C	-3.084831	-2.561655	-1.022208
C	1.329339	-2.573651	0.080956
C	3.489095	2.753407	-1.844502
C	3.48944	-2.753049	1.844386
C	3.337622	-3.762472	0.855855
C	0.19375	-2.59389	-0.80932
C	-3.085062	2.561395	1.022215
C	1.329105	2.573759	-0.08097
C	-4.252861	-2.786937	-1.998551
C	-2.658604	-3.898226	-0.393171
C	2.618356	-1.680899	1.97239
C	-2.335507	-0.393752	-2.127564
C	-2.658928	3.897941	0.393056
C	-4.253049	2.78669	1.998602
H	0.107457	3.467223	1.46604
H	2.087453	4.411421	0.764842
H	-1.639229	-2.389664	-2.648593
H	2.08789	-4.411198	-0.764931
H	-1.639434	2.389553	2.648586
H	4.330525	2.821708	-2.536644
H	4.330957	-2.821226	2.536436
H	0.10777	-3.467202	-1.466063
H	-3.96624	-3.487206	-2.7962
H	-4.56519	-1.85427	-2.473212
H	-5.107026	-3.220974	-1.462946
H	-2.252239	-4.588771	-1.146934
H	-1.912146	-3.759592	0.393499
H	-3.530617	-4.38398	0.062146
H	-2.25254	4.588556	1.146743
H	-1.912517	3.759275	-0.393653
H	-3.53099	4.383628	-0.062237
H	-4.565301	1.854041	2.473349
H	-3.966425	3.487037	2.796182
H	-5.107266	3.220638	1.463007
H	-3.411624	1.904571	0.208433
H	-3.41138	-1.904902	-0.208362

C	2.802474	0.62323	-3.031702
H	2.921138	-0.36664	-2.573918
H	1.915703	0.558916	-3.674264
H	3.679414	0.835514	-3.65413
C	4.324054	4.907643	-0.761467
H	5.341246	4.551194	-0.54646
H	4.374761	5.479352	-1.69860
H	4.043113	5.604506	0.036370
C	2.802652	-0.622924	3.031588
H	2.921156	0.366941	2.573752
H	1.915924	-0.558691	3.674217
H	3.679666	-0.835077	3.653955
C	4.324348	-4.907426	0.761621
H	5.342159	-4.55082	0.549903
H	4.372536	-5.481031	1.697746
H	4.045217	-5.602646	-0.038282

Δ-(*S*,*S*)-**3c** Charge = -1 Multiplicity = 1

Co	-0.000042	-0.000053	0.772846
O	-0.637328	1.1715330	-0.608395
O	-0.683403	1.153432	2.121804
O	0.683089	-1.153395	2.122062
N	-1.619523	-1.014520	0.929107
O	0.637492	-1.171793	-0.60819
N	1.619410	1.014433	0.929262
C	0.156350	1.997401	2.638382
C	1.574271	2.016456	2.00638
C	-1.82823	1.150319	-1.120208
O	0.098183	-2.774900	3.562141
C	-1.574493	-2.016595	2.006175
C	-4.189696	0.4117010	-1.268176
C	-1.922297	-3.481332	1.613002
C	-2.901659	0.305111	-0.676428
C	-3.384594	2.098312	-2.7632
C	-4.460757	1.292828	-2.302755
C	-2.730614	-0.686033	0.357207
C	-1.063888	-3.99758	0.448309
C	-3.417223	-3.741096	1.352983
C	-2.111600	2.049338	-2.214327
C	-0.156688	-1.997468	2.6384300
C	2.730535	0.686069	0.357360
C	2.901684	-0.305018	-0.67631
C	1.828366	-1.150387	-1.120056
C	2.111848	-2.049371	-2.21418
C	3.384833	-2.09815	-2.763089
C	4.460886	-1.292499	-2.30268
C	4.189722	-0.411418	-1.26809
O	-0.098638	2.77481	3.562078
C	1.92223	3.481196	1.613357
C	1.064098	3.997529	0.448491
C	3.417224	3.740958	1.353717
H	2.266255	1.708263	2.803402
H	-2.266648	-1.708513	2.803093
H	-4.982236	-0.235996	-0.889958
H	-3.565923	2.792150	-3.586365
H	-3.644086	-1.200859	0.666196
H	-1.249043	-3.422076	-0.464782
H	0.002717	-3.91236	0.678484
H	-1.291524	-5.052789	0.247293
H	-4.048574	-3.316915	2.144969
H	-3.605798	-4.822143	1.319776
H	-3.749898	-3.331506	0.391598
H	3.643957	1.200958	0.666385
H	3.566244	-2.791962	-3.586258
H	4.982176	0.236395	-0.889889
H	-0.002561	3.912295	0.678409
H	1.291782	5.052751	0.247602
H	1.249474	3.422094	-0.46460
H	4.048379	3.316646	2.145789
H	3.750120	3.331504	0.392351
H	3.605833	4.822005	1.320712
H	1.643094	4.037776	2.516692
H	-1.643358	-4.03795	2.516375
C	0.994300	-2.922063	-2.728701
H	0.652302	-3.617771	-1.953288
H	0.122485	-2.315349	-3.002184
H	1.316285	-3.500188	-3.602580
C	5.834514	-1.402535	-2.93051
H	5.807828	-1.190437	-4.008515
H	6.534561	-0.69524	-2.471467
H	6.256657	-2.410239	-2.811088
C	-0.993924	2.921843	-2.728885
H	-0.651702	3.617406	-1.953444
H	-0.122259	2.314973	-3.002509
H	-1.315873	3.500104	-3.602687
C	-5.834398	1.403103	-2.930515
H	-5.80774	1.191351	-4.008588
H	-6.534453	0.695682	-2.471682
H	-6.256513	2.41078	-2.810760

Λ-(*S*,*S*)-**3d** Charge = -1 Multiplicity = 1

Co	-0.028297	0.473403	-0.001827
O	-0.339637	-0.919824	1.280561
O	0.450049	-0.873734	-1.28227
O	0.31275	1.789459	-1.334206
N	-1.790239	0.423501	-0.756128
O	-0.527131	1.740217	1.327997
N	1.726341	0.638216	0.753174
C	1.565014	-1.534624	-1.288091
O	0.179989	2.719613	3.218093
C	0.390681	2.077642	2.184804
C	2.660351	-0.237546	0.575378
C	3.82874	-2.11759	-0.47023
C	-1.366439	-1.710928	1.287732
O	-0.512216	2.681244	-3.219824
C	3.960971	-3.138383	-1.3977
C	-2.024696	1.426394	-1.804226
C	-3.542309	-2.565781	0.470588
C	1.835947	1.661123	1.802405
C	1.714936	-2.588179	-2.262471
C	-3.042199	2.584879	-1.433207
C	-2.48563	-1.617133	0.393696
C	2.875616	-3.346304	-2.292035
C	-2.449134	-3.664668	2.29668
C	-3.550454	-3.592803	1.400609
C	-2.610855	-0.559153	-0.577644
C	2.704997	2.935083	1.434191
C	2.664887	-1.303782	-0.394943
C	-3.49965	3.242576	-2.754538
C	-2.390434	3.648543	-0.526686
C	-4.288236	2.027514	-0.716186
C	-1.388842	-2.771492	2.265642
C	-0.641112	2.015442	-2.188045
C	1.929497	3.913145	0.528631
C	4.010769	2.534663	0.718188
C	3.078115	3.64141	2.756901
H	3.551774	-0.173367	1.207838
H	4.642133	-1.91423	0.228033
H	-2.432096	0.921873	-2.691471
H	-4.374116	-2.463875	-0.228168
H	2.300693	1.208966	2.689708
H	2.956183	-4.140694	-3.036412
H	-2.434168	-4.460133	3.044098
H	-3.503973	-0.603905	-1.209425
H	-4.054935	2.530226	-3.380916
H	-2.639811	3.599155	-3.326738
H	-4.164879	4.088822	-2.537144
H	-1.567973	4.154425	-1.043818
H	-1.99258	3.203989	0.389583
H	-3.136619	4.408097	-0.256674
H	-4.808591	1.270176	-1.318134
H	-4.040541	1.589522	0.255545
H	-4.996993	2.846846	-0.541036
H	1.052408	4.316002	1.046582
H	1.586893	3.424997	-0.387831
H	2.578662	4.757221	0.258925
H	4.618204	1.844887	1.319776
H	3.82011	2.072005	-0.255008
H	4.615013	3.43442	0.546050
H	2.181217	3.888054	3.330111
H	3.717013	3.00157	3.381846
H	3.634068	4.563512	2.541158
C	0.575523	-2.831345	-3.220812
H	-0.344136	-3.076277	-2.675024
H	0.357393	-1.925795	-3.800402
H	0.8086	-3.649606	-3.912138
C	5.203554	-4.000834	-1.476779
H	4.971481	-5.063313	-1.318129
H	5.692327	-3.920492	-2.457926
H	5.935669	-3.704696	-0.716926
C	-0.230022	-2.87105	3.226371
H	0.713573	-3.003838	2.682622
H	-0.124872	-1.943854	3.803252
H	-0.362938	-3.70945	3.920068
C	-4.677169	-4.602074	1.478314
H	-4.319782	-5.625136	1.294496
H	-5.156339	-4.600617	2.467264
H	-5.451707	-4.383669	0.734344

Δ-(*S*,*S*)-**3d** Charge = -1 Multiplicity = 1

Co	0.000079	0.000189	0.592559
O	-0.695681	1.131899	-0.797289
O	-0.710309	1.115803	1.959609
O	0.711303	-1.115645	1.958978
N	-1.576579	-1.080291	0.767293
O	0.694991	-1.131215	-0.797953
N	1.576865	1.08059	0.766535
C	0.130802	1.915623	2.545229
C	1.475076	2.127637	1.795577

C	-1.917762	1.114627	-1.232709
O	0.102932	-2.528564	3.590106
C	-1.474323	-2.127155	1.796482
C	-4.29478	0.416397	-1.191795
C	-1.567945	-3.616949	1.263349
C	-2.967442	0.291967	-0.699598
C	-3.571296	2.051387	-2.782658
C	-4.626908	1.28672	-2.218102
C	-2.726043	-0.719391	0.299595
C	-0.233938	-4.058195	0.628206
C	-2.687843	-3.782475	0.215915
C	-2.260434	1.985024	-2.332133
C	-0.129631	-1.915093	2.545368
C	2.726167	0.719315	0.298719
C	2.967145	-0.29231	-0.700306
C	1.917102	-1.11457	-1.233326
C	2.259379	-1.98520	-2.332686
C	3.570213	-2.052196	-2.783202
C	4.626166	-1.287947	-2.218719
C	4.294421	-0.417361	-1.192509
O	-0.101266	2.529374	3.589918
C	1.568422	3.617337	1.262156
C	0.234032	4.058585	0.627826
C	2.687647	3.782573	0.213963
H	2.28884	1.992069	2.520809
H	-2.287711	-1.991424	2.522103
H	-5.067361	-0.211521	-0.745468
H	-3.799129	2.725181	-3.61088
H	-3.617459	-1.239148	0.664435
H	0.078932	-3.352042	-0.145599
H	0.561206	-4.107673	1.379016
H	-0.343474	-5.05533	0.180505
H	-3.670025	-3.491697	0.611462
H	-2.754241	-4.83956	-0.07175
H	-2.495069	-3.202124	-0.691789
H	3.617772	1.238954	0.663288
H	3.797746	-2.726178	-3.611351
H	5.06728	0.210259	-0.746249
H	-0.560616	4.108168	1.379155
H	0.343335	5.055674	0.179965
H	-0.079361	3.35237	-0.145706
H	3.670071	3.491838	0.608939
H	2.494252	3.201985	-0.693458
H	2.753917	4.839582	-0.074011
C	1.163058	-2.803355	-2.970362
H	0.761767	-3.54595	-2.270241
H	0.319699	-2.163512	-3.256406
H	1.531721	-3.328031	-3.85933
C	6.042704	-1.422733	-2.736706
H	6.104025	-1.213683	-3.813778
H	6.716446	-0.72606	-2.225186
H	6.43705	-2.43696	-2.582487
C	-1.164493	2.803643	-2.969866
H	-0.763636	3.546566	-2.269843
H	-0.320787	2.164185	-3.255746
H	-1.533367	3.327985	-3.858944
C	-6.043497	1.420806	-2.736126
H	-6.104727	1.211511	-3.813156
H	-6.716953	0.723958	-2.224468
H	-6.438265	2.434901	-2.582117
C	-1.899598	-4.530795	2.463926
H	-2.892104	-4.298226	2.874374
H	-1.906658	-5.580842	2.141703
H	-1.163178	-4.402388	3.260409
C	1.900896	4.531341	2.462385
H	2.893658	4.298797	2.872223
H	1.907776	5.581346	2.140018
H	1.164978	4.403063	3.259356

Δ-(*R*,*R*)-**3d** Charge = -1 Multiplicity = 1

Co	0.00745	-0.475585	0.000370
O	-0.408717	0.889695	1.282956
O	-0.389581	-1.777666	1.330974
O	0.446505	-1.76294	-1.331307
N	-1.753892	-0.560711	-0.752371
O	0.379032	0.903352	-1.281315
N	1.770676	-0.504033	0.753099
C	0.553057	-2.043303	2.185918
C	1.961182	-1.516295	1.801038
C	-1.491977	1.601763	1.289291
O	-0.308074	-2.710684	-3.219264
C	-1.911635	-1.578245	-1.800619
C	-3.725836	2.28955	0.471180
C	-2.600994	1.423033	0.395487
C	-2.718253	3.470071	2.295438
C	-3.811016	3.31465	1.399808
C	-2.646168	0.357443	-0.574677
C	-1.593758	2.658895	2.265696
C	-0.48727	-2.059348	-2.185819

C	2.633102	0.442208	0.575305
C	2.55385	1.505641	-0.395028
C	1.439377	1.649091	-1.288419
C	1.507471	2.708501	-2.265285
C	2.605903	3.554606	-2.29583
C	3.703408	3.433944	-1.400627
C	3.650839	2.407104	-0.471511
O	0.394824	-2.699961	3.219406
H	-4.54748	2.124866	-0.227631
H	-2.76256	4.266016	3.041249
H	-3.540198	0.334059	-1.206343
H	3.527522	0.447484	1.20684
H	2.624912	4.351179	-3.042041
H	4.477575	2.268582	0.226972
C	0.353733	2.859958	-3.224917
H	0.207229	1.939311	-3.80318
H	-0.583144	3.032914	-2.680826
H	0.522057	3.692624	-3.917521
C	4.873637	4.391924	-1.478681
H	4.560026	5.431701	-1.310239
H	5.631568	4.149346	-0.725225
H	5.361848	4.358342	-2.462658
C	-0.44571	2.847009	3.225661
H	-0.270234	1.931562	3.804105
H	0.48537	3.049538	2.681825
H	-0.640555	3.674026	3.918087
C	-5.010665	4.235613	1.476773
H	-4.729704	5.284528	1.307343
H	-5.760505	3.968723	0.723438
H	-5.497748	4.187754	2.460715
H	2.391699	-1.030593	2.687871
C	2.925205	-2.718782	1.428793
C	2.225919	-3.751337	0.52171
H	1.382128	-4.220592	1.039065
H	1.847212	-3.288707	-0.393745
H	2.937247	-4.543133	0.250259
C	4.194738	-2.216918	0.711827
H	4.866818	-3.066628	0.536903
H	3.966802	-1.768549	-0.260019
H	4.747758	-1.483041	1.313763
C	3.353244	-3.397396	2.749387
H	3.940894	-2.711475	3.375726
H	2.478631	-3.714784	3.322283
H	3.979036	-4.272925	2.530891
H	-2.357548	-1.106259	-2.687255
C	-2.836358	-2.8113	-1.429164
C	-4.121428	-2.351172	-0.711709
H	-4.765949	-3.222216	-0.537997
H	-3.908269	-1.896921	0.260779
H	-4.697582	-1.634689	-1.31289
C	-3.242645	-3.502104	-2.750322
H	-3.852686	-2.835005	-3.375588
H	-2.358375	-3.790079	-3.323924
H	-3.839446	-4.397809	-2.532507
C	-2.104146	-3.821775	-0.523114
H	-1.24608	-4.263428	-1.041292
H	-1.739891	-3.348188	0.392630
H	-2.789785	-4.636051	-0.252079

Λ-(*R*,*R*)-**3d** Charge = -1 Multiplicity = 1

Co	0.007838	-0.659816	0.029869
O	0.172508	1.004012	0.959614
O	0.006828	0.34195	-1.610923
O	-0.176564	-2.3373	-0.853031
N	1.890989	-1.00562	-0.079521
O	-0.017762	-1.530128	1.729416
N	-1.894209	-0.560862	0.251747
C	-0.880252	1.261473	-1.863755
O	-1.420323	-2.081848	3.385156
C	-1.198544	-1.762041	2.214871
C	-2.614028	0.379959	-0.255807
C	-3.068126	2.385265	-1.585701
C	1.262042	1.447528	1.504146
O	0.936921	-4.226898	-1.324865
C	-2.767111	3.334535	-2.55257
C	2.232512	-2.254619	-0.776451
C	3.667865	1.373132	2.085803
C	-2.330718	-1.6631	1.127042
C	-0.578885	2.239231	-2.877869
C	2.545355	0.809504	1.419351
C	-1.502574	3.230123	-3.186799
C	2.313488	3.182852	2.878100
C	3.583779	2.54785	2.815751
C	2.761846	-0.392695	0.654344
C	-2.158883	1.358819	-1.220842
C	1.182019	2.675139	2.257663
C	0.90922	-3.038125	-0.993372
H	-3.6626	0.458918	0.022662
H	-4.034783	2.419585	-1.080503

H	4.624396	0.854239	2.006652
H	-1.238422	3.962501	-3.952081
H	2.219766	4.110092	3.446627
H	3.774078	-0.806799	0.706397
C	0.757789	2.162866	-3.573438
H	1.575475	2.162828	-2.842444
H	0.854531	1.230184	-4.143029
H	0.893193	3.006325	-4.260224
C	-3.73484	4.437736	-2.927208
H	-3.315634	5.433133	-2.723895
H	-3.99185	4.408692	-3.995253
H	-4.668482	4.351373	-2.359822
C	-0.159014	3.358626	2.354019
H	-0.555848	3.583006	1.356508
H	-0.893663	2.699558	2.833537
H	-0.088277	4.289083	2.929129
C	4.781518	3.144076	3.52505
H	5.001694	4.160213	3.16888
H	4.61894	3.211227	4.609863
H	5.678073	2.535049	3.362896
H	2.878983	-2.85905	-0.125414
C	2.997062	-2.083001	-2.15388
C	3.66092	-3.435341	-2.495917
H	2.918176	-4.236504	-2.511072
H	4.146789	-3.37369	-3.478985
H	4.430384	-3.696685	-1.756125
C	4.105795	-1.016028	-2.054607
H	3.700321	-0.017667	-1.863179
H	4.835052	-1.250138	-1.267673
H	4.653557	-0.977968	-3.00492
C	2.026687	-1.682853	-3.28362
H	1.311844	-2.486686	-3.488671
H	1.457748	-0.788986	-3.014559
H	2.590582	-1.48577	-4.205722
C	-3.807116	-1.761825	1.623291
H	-2.145069	-2.558995	0.514068
C	-3.963675	-3.127837	2.340596
H	-4.998494	-3.238832	2.690932
H	-3.753112	-3.955481	1.650049
H	-3.284009	-3.202883	3.189503
C	-4.806173	-1.801911	0.434517
H	-5.032682	-0.823581	0.002188
H	-4.431126	-2.442402	-0.373442
H	-5.759389	-2.223431	0.778139
C	-4.170844	-0.635764	2.613525
H	-3.529022	-0.697704	3.497042
H	-4.048527	0.362402	2.177154
H	-5.217228	-0.734487	2.932410

Λ-(*S*,*R*,*S*,*R*)-**3e** Charge = -1 Multiplicity = 1

Co	-0.031124	-0.502209	0.103699
O	0.74157	0.918835	1.100596
O	-0.621579	0.773688	-1.19523
O	-0.867052	-1.913373	-0.909261
N	1.49919	-0.823238	-0.991706
O	0.599179	-1.723345	1.436274
N	-1.597344	-0.389066	1.166887
C	-1.639079	1.571263	-1.049076
O	0.014559	-2.616627	3.423793
C	-0.237069	-1.966348	2.406893
C	-2.555241	0.462896	1.017775
C	-3.694859	2.405463	0.050648
C	1.935681	1.397037	0.93027
O	-0.495073	-3.367595	-2.582742
C	-3.860455	3.405199	-0.895592
C	1.28695	-1.868195	-1.994603
C	4.154018	1.572491	-0.159855
C	-1.668998	-1.467375	2.136743
C	-1.821076	2.606585	-2.034864
C	2.326764	-3.033212	-1.957959
C	2.878596	0.950893	-0.057061
C	-2.896616	3.478113	-1.9362
C	3.605584	3.037825	1.65347
C	4.541887	2.608603	0.673205
C	2.586577	-0.125461	-0.968313
C	-2.481008	-2.719398	1.581316
C	-2.609288	1.490351	0.005864
C	2.348209	2.472378	1.799268
C	-0.136719	-2.438867	-1.83928
H	-3.404738	0.407737	1.705810
H	-4.417065	2.30423	0.861900
H	1.346955	-1.434729	-3.002621
H	4.84206	1.206989	-0.923584
H	-2.139434	-1.122319	3.066010
H	-3.00525	4.253748	-2.696577
H	3.887895	3.850778	2.324935
H	3.36803	-0.357193	-1.700887
O	2.152114	-3.83556	-3.119622
H	1.181869	-3.980258	-3.175872

H	3.326164	-2.584915	-2.051600
C	2.270223	-3.857921	-0.66663
H	2.356398	-3.225108	0.222818
H	1.318774	-4.397227	-0.600839
H	3.080786	-4.595478	-0.672473
C	-0.815535	2.703794	-3.155266
H	0.197329	2.841299	-2.757182
H	-0.791722	1.775184	-3.739209
H	-1.05098	3.53759	-3.826483
C	-5.015633	4.382935	-0.841239
H	-4.665926	5.420362	-0.745601
H	-5.632691	4.333175	-1.74904
H	-5.668113	4.173813	0.013910
C	1.37438	2.945372	2.849302
H	0.440227	3.287915	2.387437
H	1.099712	2.122379	3.520519
H	1.799551	3.762596	3.442999
C	5.901422	3.266163	0.563722
H	5.816808	4.341352	0.353121
H	6.477322	3.164313	1.493977
H	6.492565	2.81652	-0.241968
H	-1.743269	-3.43295	1.192566
O	-3.37976	-2.367482	0.544622
H	-2.820743	-2.206861	-0.239361
C	-3.266884	-3.374124	2.712671
H	-3.73251	-4.301983	2.361776
H	-2.59494	-3.600537	3.547184
H	-4.06029	-2.701729	3.063515

Δ-(*S*,*R*,*S*,*R*)-**3e** Charge = -1 Multiplicity = 1

Co	-0.00748	-0.047538	0.7476530
O	-0.870204	1.225414	-0.392669
O	-0.756223	0.769347	2.301138
O	0.891123	-1.309646	1.868594
N	-1.455822	-1.296237	0.747880
O	0.731764	-0.903608	-0.795221
N	1.443092	1.1685600	0.983860
C	-0.036408	1.6985320	2.852603
C	1.169563	2.1792570	2.004157
C	-1.99864	1.0428770	-1.009334
O	0.611241	-3.264036	2.938545
C	-1.265599	-2.379326	1.719030
C	-4.103088	-0.159119	-1.524467
C	-1.630234	-3.809940	1.218778
C	-2.871237	-0.081239	-0.819479
C	-3.629737	1.917574	-2.617155
C	-4.505103	0.815365	-2.423942
C	-2.558453	-1.154464	0.091643
C	-2.421369	2.052639	-1.948991
C	0.193999	-2.331555	2.225007
C	2.580600	1.094008	0.377393
C	2.901786	0.186671	-0.696916
C	1.943166	-0.736165	-1.236715
C	2.360058	-1.530686	-2.365873
C	3.647790	-1.404838	-2.867349
C	4.60877	-0.511617	-2.323667
C	4.207662	0.268559	-1.250743
O	-0.261015	2.221688	3.946460
C	0.890817	3.554695	1.324503
H	2.027979	2.300088	2.680987
H	-1.894889	-2.180757	2.598165
H	-4.742900	-1.024273	-1.344379
H	-3.919709	2.697822	-3.323363
H	-3.344853	-1.904349	0.226460
H	3.3704000	1.787583	0.676627
H	3.932829	-2.024546	-3.719652
H	4.906109	0.981369	-0.810211
H	-2.685779	-3.806684	0.910701
C	-1.518787	3.2401740	-2.174050
H	-1.421686	3.839462	-1.259380
H	-0.505116	2.915586	-2.436712
H	-1.906485	3.8853400	-2.970600
C	-5.815341	0.726388	-3.177804
H	-5.658063	0.710009	-4.265243
H	-6.362915	-0.183988	-2.909216
H	-6.468461	1.582701	-2.959511
C	1.365074	-2.490226	-2.969666
H	1.784907	-2.991761	-3.849172
H	1.065695	-3.252006	-2.239690
H	0.446564	-1.966707	-3.261758
C	6.003436	-0.425454	-2.907283
H	5.982387	-0.161946	-3.97389
H	6.599787	0.334742	-2.390197
H	6.539358	-1.381108	-2.821811
O	-1.551922	-4.712335	2.312015
H	-0.704321	-4.47318	2.758150
C	-0.772762	-4.266459	0.030495
H	-0.855810	-3.570526	-0.811248
H	0.281169	-4.326192	0.323124

H	-1.09949	-5.261764	-0.291278
C	0.320023	4.6211780	2.260377
H	0.984353	4.7926490	3.118079
H	0.211557	5.5583100	1.701251
H	-0.64852	4.3226250	2.665630
O	2.094804	4.0402100	0.698700
H	2.662221	4.3743060	1.409504
H	0.202314	3.3672260	0.496000

Λ-(*S*,*S*,*S*,*S*)-**3f** Charge = -1 Multiplicity = 1

Co	-0.138166	-0.316985	-0.141833
O	0.822511	0.43088	1.341796
O	0.212957	1.291599	-1.120398
O	-1.06565	-0.987316	-1.671932
N	1.409642	-1.071496	-0.958209
O	-0.446471	-1.915213	0.846959
N	-1.755807	0.30549	0.656992
C	-0.4971	2.37464	-1.07458
O	-1.76816	-2.843669	2.405633
C	-1.477273	-1.921688	1.637072
C	-2.222334	1.503762	0.541164
C	-2.366954	3.801021	-0.295985
C	2.104603	0.636823	1.359097
O	-0.697158	-1.934267	-3.678524
C	-1.941914	4.872824	-1.064241
C	1.076691	-2.010844	-2.02644
C	4.420547	0.436887	0.502246
C	-2.385129	-0.668474	1.565019
C	-0.064612	3.491788	-1.879628
C	1.033161	-3.505293	-1.541939
C	3.035792	0.142485	0.383114
C	-0.775821	4.681734	-1.854839
C	4.005416	1.669179	2.513456
C	4.9302	1.197017	1.543888
C	2.623076	-0.718295	-0.699673
C	-3.850596	-1.019081	1.161125
C	-1.680396	2.555869	-0.281422
C	2.643635	1.410513	2.450126
C	-0.330153	-1.61281	-2.545159
C	-4.62407	-1.608645	2.359062
H	-3.118179	1.766766	1.116013
H	-3.261217	3.900557	0.321354
H	1.805967	-1.929758	-2.845553
H	5.096105	0.0408	-0.257593
H	-2.40772	-0.240052	2.577611
H	-0.421877	5.509142	-2.472755
H	4.380735	2.264768	3.34782
H	3.422956	-1.119301	-1.332872
H	-4.163864	-2.5607	2.642313
H	-4.497084	-0.938185	3.222959
C	1.168672	3.313533	-2.729237
H	2.034106	3.051927	-2.107926
H	1.035634	2.483297	-3.43386
H	1.39636	4.22593	-3.292343
C	-2.67686	6.196763	-1.078557
H	-2.03317	7.02312	-0.745929
H	-3.034028	6.452427	-2.085912
H	-3.549225	6.169802	-0.415762
C	1.683523	1.924879	3.493723
H	0.916026	2.560023	3.034263
H	1.148162	1.095359	3.972153
H	2.208342	2.50093	4.264526
C	6.406048	1.515324	1.662552
H	6.592924	2.597784	1.626854
H	6.826953	1.147049	2.608503
H	6.974567	1.055347	0.846319
C	0.920496	-4.433124	-2.765444
H	0.128181	-4.09504	-3.437591
H	1.863353	-4.446519	-3.331855
H	-4.331185	-0.059451	0.91175
C	-3.906717	-1.910018	-0.092145
H	-3.273322	-1.511402	-0.890733
H	-3.552857	-2.921031	0.142582
C	2.236825	-3.873673	-0.652447
H	3.172893	-3.743113	-1.220383
H	2.275055	-3.184821	0.196523
H	0.134474	-3.607335	-0.92265
C	-6.127667	-1.793006	2.097379
H	-6.598522	-0.851693	1.781161
H	-6.314243	-2.535728	1.313824
H	-6.641734	-2.134846	3.004923
H	-4.932426	-1.987345	-0.471474
H	0.701066	-5.460862	-2.455302
C	2.176175	-5.304642	-0.093744
H	2.236885	-6.063413	-0.882623
H	3.005193	-5.481969	0.603111
H	1.240557	-5.46061	0.457049

Δ-(*S*,*S*,*S*,*S*)-**3f** Charge = -1 Multiplicity = 1

Co	-0.059111	-0.049550	0.660228
O	0.510838	1.479327	-0.354888
O	-0.022103	0.943762	2.285883
O	-0.586612	-1.586001	1.643809
N	-1.928275	0.321226	0.524586
O	-0.112574	-1.070331	-0.968238
N	1.757560	-0.473053	1.063529
C	1.080970	0.869599	2.970602
C	2.293751	0.295739	2.187342
C	-0.275532	2.320725	-0.951448
O	-2.339008	-2.579629	2.629063
C	-2.759434	-0.568326	1.360640
C	-2.470146	3.322389	-1.517802
C	-4.003042	-1.137947	0.618336
C	-1.708076	2.325540	-0.850503
C	-0.468462	4.284566	-2.410066
C	-1.884611	4.306107	-2.299078
C	-2.430676	1.347310	-0.074837
C	0.326923	3.343427	-1.772926
C	-1.846519	-1.681944	1.939347
C	2.391520	-1.476895	0.557361
C	1.978287	-2.247272	-0.589914
C	0.771613	-1.956365	-1.314823
C	0.541974	-2.702719	-2.528280
C	1.438451	-3.686754	-2.920793
C	2.608583	-4.010665	-2.184085
C	2.854194	-3.272769	-1.036770
O	1.216995	1.263885	4.131728
C	3.226323	1.477479	1.736712
H	2.869616	-0.361745	2.854624
H	-3.108138	0.012775	2.226567
H	-3.555307	3.297717	-1.406224
H	0.019739	5.044512	-3.023207
H	-3.510801	1.514377	-0.001906
H	3.349471	-1.761766	1.006771
H	1.230063	-4.237929	-3.839829
H	3.752707	-3.470259	-0.450153
H	-4.455049	-0.294660	0.073191
H	2.574991	2.227900	1.270142
C	4.261669	1.040116	0.679563
H	4.879481	0.224459	1.088500
H	3.737465	0.634696	-0.191327
C	3.911309	2.098043	2.968807
H	4.667541	1.411894	3.377304
H	3.181735	2.301161	3.755322
C	-3.598748	-2.198646	-0.419846
H	-2.780139	-1.827181	-1.044630
H	-3.253203	-3.110176	0.080402
C	-5.071712	-1.634664	1.614027
H	-5.256214	-0.839195	2.351764
H	-4.665054	-2.482120	2.173772
C	1.828757	3.352759	-1.907989
H	2.306724	3.513815	-0.933535
H	2.193479	2.384134	-2.270727
H	2.159588	4.139940	-2.595114
C	-2.701296	5.364004	-3.011133
H	-2.551180	5.330069	-4.099284
H	-3.772006	5.228487	-2.820951
H	-2.431832	6.376784	-2.680370
C	-0.688105	-2.386091	-3.342454
H	-0.689954	-2.944565	-4.285643
H	-1.601172	-2.635889	-2.788237
H	-0.744786	-1.313074	-3.561011
C	3.538961	-5.110385	-2.651144
H	3.903498	-4.929236	-3.671875
H	4.413842	-5.190030	-1.995979
H	3.040113	-6.089693	-2.656180
C	-6.407087	-2.011140	0.951913
H	-6.299238	-2.870267	0.279961
H	-7.154743	-2.278727	1.709438
H	-6.813485	-1.177142	0.362496
H	-4.440226	-2.458463	-1.072854
H	4.415981	3.034068	2.704023
C	5.187379	2.169674	0.198374
H	5.840643	2.538877	0.996812
H	5.828189	1.819367	-0.620730
H	4.605066	3.020227	-0.177934

Λ-**3h** Charge = -1 Multiplicity = 1

Co	0.000025	-0.733274	-0.000013
O	0.879685	0.639020	1.011683
O	-0.880243	0.638330	-1.012133
O	-0.927422	-2.072704	-0.992341
N	1.331041	-0.822485	-1.356007
O	0.928072	-2.071916	0.992821
N	-1.330967	-0.822622	1.355985
C	-1.942544	1.279605	-0.644897
O	0.758277	-3.351417	2.842473

C	0.322539	-2.508850	2.056067
C	-2.354196	-0.047210	1.466244
C	-3.857902	1.785264	0.846141
C	1.942093	1.280196	0.644590
O	-0.757220	-3.352647	-2.841645
C	-4.291360	2.810926	0.016302
C	1.078255	-1.908065	-2.297578
C	3.857775	1.785592	-0.846120
C	-1.078026	-1.908201	2.297498
C	-2.420531	2.340951	-1.483232
C	2.705927	1.013111	-0.551874
C	-3.550723	3.077653	-1.161793
C	3.550087	3.078416	1.161457
C	4.291014	2.811444	-0.016400
C	2.354218	-0.046997	-1.466223
C	-2.706096	1.012756	0.551800
C	2.419839	2.341754	1.482793
C	-0.321834	-2.509586	-2.055571
H	-3.030472	-0.201269	2.316423
H	-4.412176	1.553502	1.756750
H	1.148987	-1.572428	-3.339478
H	4.412262	1.553656	-1.756555
H	-1.149606	-1.572867	3.339435
H	-3.872668	3.876020	-1.830845
H	3.871853	3.876938	1.830410
H	3.030589	-0.201066	-2.316325
H	-1.802180	-2.719661	2.145262
H	1.803014	-2.719102	-2.146000
H	-1.852462	2.545993	-2.387785
H	-5.177295	3.392539	0.260380
H	5.176991	3.393026	-0.260400
H	1.851553	2.546981	2.387166

Δ-**3h** Charge = -1 Multiplicity = 1

Co	0.000416	0.733157	-0.000139
O	0.876328	-0.643441	1.008837
O	0.932248	2.067122	0.995564
O	-0.923510	2.077260	-0.989394
N	1.331562	0.821583	-1.356120
O	-0.883704	-0.634160	-1.014735
N	-1.330240	0.823381	1.356116
C	0.327130	2.504507	2.058871
C	-1.076046	1.908782	2.297377
C	1.939297	-1.284097	0.642476

O	-0.750985	3.359627	-2.836785
C	1.079896	1.907549	-2.297583
C	3.857022	-1.787744	-0.846167
C	2.704870	-1.015467	-0.552500
C	3.546213	-3.083328	1.159148
C	4.288924	-2.814784	-0.017237
C	2.354382	0.045590	-1.466192
C	2.415598	-2.346966	1.479847
C	-0.317629	2.513760	-2.052597
C	-2.353591	0.048235	1.466934
C	-2.707013	-1.010586	0.551720
C	-1.945691	-1.275603	-0.646793
C	-2.425725	-2.335097	-1.486289
C	-3.555597	-3.071989	-1.164127
C	-4.293918	-2.807206	0.015851
C	-3.858566	-1.783200	0.846756
O	0.764958	3.344318	2.847060
H	-1.796822	2.722655	2.141865
H	1.807838	2.716254	-2.148894
H	4.412832	-1.554657	-1.755500
H	3.866883	-3.882832	1.827453
H	3.031365	0.199841	-2.315773
H	-3.028886	0.201865	2.317967
H	-3.879097	-3.868960	-1.834094
H	-4.411139	-1.552840	1.758754
H	1.146496	1.570860	-3.339431
H	-1.152064	1.575013	3.339499
H	5.175193	-3.396125	-0.260742
H	1.845940	-2.553382	2.383081
H	-1.859433	-2.538657	-2.392285
H	-5.179608	-3.388944	0.260513

Λ-**3i** Charge = -1 Multiplicity = 1

Co	-0.000012	-1.163563	-0.000018
O	0.912736	0.208181	0.981228
O	-0.912798	0.208117	-0.981278
O	-0.954910	-2.502217	-0.968998
N	1.292976	-1.252083	-1.391862
O	0.954998	-2.502092	0.969087
N	-1.293026	-1.252124	1.391805
C	-1.960040	0.857105	-0.576616
O	0.835114	-3.777118	2.825793
C	0.376461	-2.939127	2.047140
C	-2.307524	-0.469262	1.535625

C	-3.819960	1.377232	0.977493
C	1.960075	0.857044	0.576656
O	-0.835081	-3.777228	-2.825717
C	-4.279328	2.406719	0.171200
C	1.022638	-2.346984	-2.316789
C	3.819954	1.377228	-0.977495
C	-1.022668	-2.347027	2.316729
C	-2.449369	1.925240	-1.415024
C	2.681547	0.596701	-0.638775
C	-3.564093	2.655867	-1.031518
C	3.564103	2.655844	1.031532
C	4.279334	2.406702	-0.171195
C	2.307496	-0.469238	-1.535647
C	-2.681553	0.596711	0.638774
C	2.449396	1.925205	1.415048
C	-0.376433	-2.939184	-2.047127
H	-2.958981	-0.623692	2.404987
H	-4.342333	1.145558	1.907121
H	1.082866	-2.026430	-3.364156
H	4.342289	1.145597	-1.907154
H	-1.082991	-2.026498	3.364096
H	-3.906898	3.458952	-1.686759
H	3.906917	3.458920	1.686779
H	2.958934	-0.623637	-2.405031
C	-1.710857	2.208506	-2.699270
H	-0.659526	2.448394	-2.497736
H	-1.703188	1.321995	-3.345548
H	-2.169300	3.042004	-3.243866
C	-5.491225	3.238027	0.536628
H	-5.238290	4.302496	0.640845
H	-6.278143	3.167540	-0.227263
H	-5.923444	2.906667	1.487675
C	1.710893	2.208456	2.699302
H	0.659565	2.448386	2.497785
H	1.703202	1.321923	3.345545
H	2.169358	3.041926	3.243921
C	5.491205	3.238038	-0.536643
H	5.238269	4.302512	-0.640819
H	6.278149	3.167531	0.227222
H	5.923394	2.906702	-1.487711
H	-1.744387	-3.160620	2.162863
H	1.744429	-3.160528	-2.162997

Δ-3i Charge = -1 Multiplicity = 1

Co	0.000406	1.163397	0.000310
O	0.911028	-0.211143	0.979564
O	0.957676	2.498986	0.971232
O	-0.952471	2.505141	-0.966313
N	1.293407	1.251852	-1.391559
O	-0.914772	-0.205484	-0.982702
N	-1.292357	1.252022	1.392318
C	0.379379	2.936017	2.049450
C	-1.021055	2.346378	2.317593
C	1.958836	-0.859419	0.575299
O	-0.831532	3.782062	-2.821632
C	1.023116	2.346589	-2.316789
C	3.820778	-1.377100	-0.977201
C	2.681734	-0.597317	-0.638896
C	3.562557	-2.658618	1.029682
C	4.279392	-2.407521	-0.171687
C	2.308099	0.469205	-1.535228
C	2.447165	-1.928789	1.412735
C	-0.374032	2.942027	-2.044499
C	-2.307166	0.469567	1.536076
C	-2.682089	-0.595550	0.638570
C	-1.961648	-0.854863	-0.577689
C	-2.451754	-1.922221	-1.416631
C	-3.566298	-2.653024	-1.032932
C	-4.280586	-2.404799	0.170537
C	-3.820404	-1.376140	0.977425
O	0.839230	3.772331	2.829200
H	-1.741157	3.161221	2.162781
H	1.747232	3.158629	-2.166165
H	4.344240	-1.144099	-1.905883
H	3.904606	-3.462627	1.684180
H	2.959908	0.624086	-2.404242
H	-2.958136	0.623690	2.405855
H	-3.909665	-3.455540	-1.688572
H	-4.342002	-1.145247	1.907680
C	-1.714210	-2.204517	-2.701664
H	-1.707334	-1.317611	-3.347427
H	-0.662670	-2.444226	-2.501101
H	-2.172934	-3.037761	-3.246412
C	-5.492473	-3.236114	0.536062
H	-5.239932	-4.300861	0.638290
H	-5.923405	-2.906010	1.488125
H	-6.280222	-3.163997	-0.226819
C	1.706938	-2.214090	2.695557

H	1.698714	-1.328680	3.343355
H	0.655820	-2.453331	2.492256
H	2.164541	-3.048593	3.239320
C	5.492203	-3.237801	-0.536495
H	5.240222	-4.302468	-0.640883
H	5.924652	-2.905982	-1.487273
H	6.278622	-3.166640	0.227849
H	1.079571	2.024624	-3.363946
H	-1.083172	2.026019	3.364908

Λ-**3j** Charge = -1 Multiplicity = 1

Co	-0.000407	-0.987096	-0.000020
O	0.789686	0.386135	1.080357
O	-0.787873	0.388672	-1.079055
O	-0.823478	-2.323061	-1.086613
N	1.446157	-1.071793	-1.233308
O	0.820280	-2.325637	1.085165
N	-1.447159	-1.070502	1.233145
C	-1.837758	1.082273	-0.764334
O	0.471637	-3.602238	2.907770
C	0.106451	-2.765219	2.078800
C	-2.430683	-0.238793	1.295374
C	-3.798552	1.683160	0.627084
C	1.839665	1.079416	0.765189
O	-0.476334	-3.599576	-2.909570
C	-4.141530	2.732501	-0.211221
C	1.326157	-2.193955	-2.168673
C	3.799879	1.679857	-0.627238
C	-1.328665	-2.193158	2.168140
C	-2.207958	2.168557	-1.639227
C	2.335431	-3.311531	-1.841867
C	2.672505	0.849943	-0.382984
C	-3.317705	2.948363	-1.348962
C	3.321137	2.944299	1.349806
C	4.144054	2.728553	0.211389
C	2.430398	-0.240946	-1.295591
C	-2.339931	-3.308960	1.841447
C	-2.671522	0.852679	0.383143
C	2.211118	2.165001	1.640412
C	-0.110003	-2.763541	-2.080109
H	-3.166262	-0.368367	2.099048
H	-4.408397	1.474370	1.507600
H	1.483117	-1.845912	-3.199097
H	4.409004	1.471162	-1.508277
H	-1.484572	-1.845139	3.198730
H	-3.567934	3.765511	-2.028267
H	3.572312	3.760948	2.029363
H	3.165559	-0.370865	-2.099594
C	-1.351292	2.418093	-2.855321
H	-0.311997	2.613968	-2.563861
H	-1.325574	1.530906	-3.500209
H	-1.726012	3.269255	-3.435530
C	-5.340764	3.618270	0.054663
H	-5.048801	4.669431	0.188004
H	-6.061337	3.587131	-0.774520
H	-5.868141	3.304429	0.962692
C	1.355424	2.414413	2.857215
H	0.316037	2.610984	2.566551
H	1.329665	1.526937	3.501704
H	1.730999	3.265097	3.437573
C	5.343616	3.613766	-0.054862
H	5.052115	4.665095	-0.187903
H	6.064526	3.582131	0.774008
H	5.870450	3.299824	-0.963172
H	-3.370408	-2.955554	1.973309
H	-2.210544	-3.628808	0.803062
H	-2.162977	-4.157105	2.510217
H	2.157346	-4.159193	-2.510952
H	3.366531	-2.959754	-1.973237
H	2.205106	-3.631432	-0.803620

Δ-**3j** Charge = -1 Multiplicity = 1

Co	-0.000011	0.907971	-0.000026
O	1.005084	-0.452286	0.908388
O	1.029698	2.251956	0.869799
O	-1.030001	2.251512	-0.870200
N	1.163312	1.007993	-1.504620
O	-1.004872	-0.452716	-0.908119
N	-1.163346	1.008139	1.504530
C	0.585844	2.655101	2.021413
C	-0.707797	1.964570	2.521498
C	2.139068	-0.943234	0.514835
O	-1.114020	3.514268	-2.731734
C	0.707733	1.964323	-2.521681
C	4.074009	-1.175763	-1.015594
C	0.449079	1.302336	-3.885636
C	2.826846	-0.576422	-0.691939
C	3.980792	-2.495206	0.980429

C	4.671898	-2.128650	-0.205792
C	2.286846	0.385342	-1.621729
C	2.763694	-1.941560	1.349308
C	-0.586173	2.654549	-2.021856
C	-2.286860	0.385454	1.621713
C	-2.826803	-0.576457	0.692051
C	-2.138925	-0.943517	-0.514592
C	-2.763527	-1.941961	-1.348951
C	-3.980696	-2.495465	-0.980104
C	-4.671903	-2.128660	0.205986
C	-4.074019	-1.175698	1.015702
O	1.113520	3.515089	2.731088
C	-0.448668	1.302584	3.885368
H	-1.459664	2.757765	2.637937
H	1.459426	2.757716	-2.637912
H	4.567096	-0.866347	-1.938556
H	4.427697	-3.245681	1.635339
H	2.899652	0.587453	-2.508339
H	-2.899683	0.587677	2.508292
H	-4.427602	-3.245994	-1.634951
H	-4.567180	-0.866088	1.938558
C	-2.055518	-2.347791	-2.617450
H	-1.938761	-1.488001	-3.289518
H	-1.041857	-2.706006	-2.400346
H	-2.607924	-3.134098	-3.144719
C	-6.003391	-2.761316	0.552685
H	-5.924221	-3.853792	0.642280
H	-6.384672	-2.378070	1.506010
H	-6.763289	-2.555401	-0.214136
C	2.055749	-2.347206	2.617900
H	1.939149	-1.487343	3.289900
H	1.042028	-2.705333	2.400924
H	2.608118	-3.133524	3.145190
C	6.003307	-2.761455	-0.552527
H	5.924091	-3.853953	-0.641797
H	6.384450	-2.378490	-1.506021
H	6.763334	-2.555342	0.214113
H	-0.035729	2.053342	4.565415
H	0.273935	0.487919	3.770989
H	-1.369660	0.893479	4.318838
H	1.370270	0.893531	-4.318963
H	0.036047	2.053015	-4.565714
H	-0.273314	0.487454	-3.771457

11. Kinetic study and data analysis

To get insight into the reaction mechanism, kinetic studies on the iodocyclization reaction of **1a** and **1c** were performed (Figure S1). Comparing both the catalytic and background reactions of two substrates **1a** and **1c** can explain the huge variance in enantioselectivities.

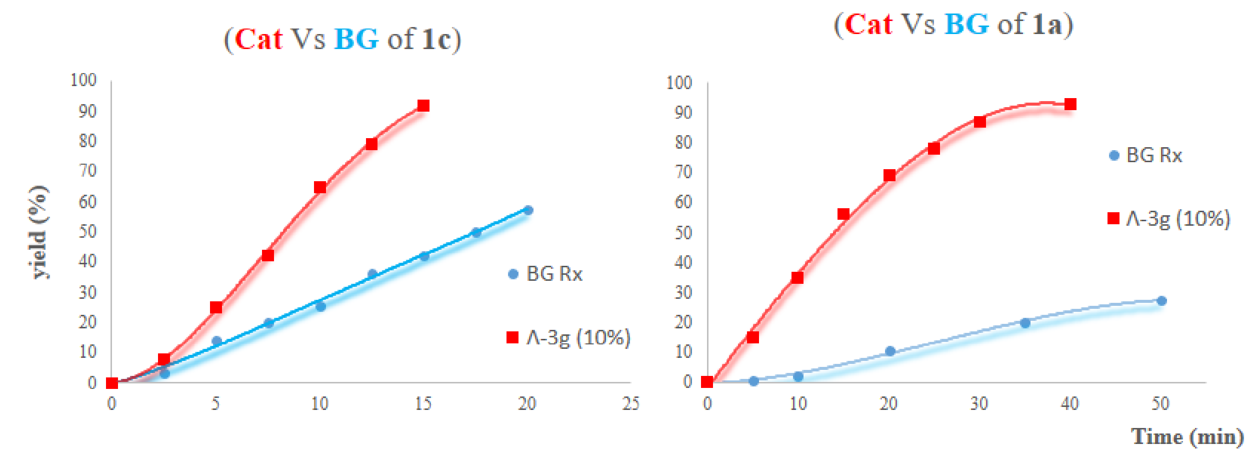

Figure S1. Kinetic Studies on the Iodocyclization Reaction of Enol Ethers: (a) The progress of iodocyclization reaction of background and catalytic pathways of **1c** vs time, (b) The progress of iodocyclization reaction of background and catalytic pathways of **1a** vs time

---

The calculated rate constants *k* for the formation of **2c** showed that the catalytic pathway proceeded 2.25 times faster than the background pathway, while the rate constants *k* for the formation of **2a** showed around 5 times faster catalytic than the background pathway.

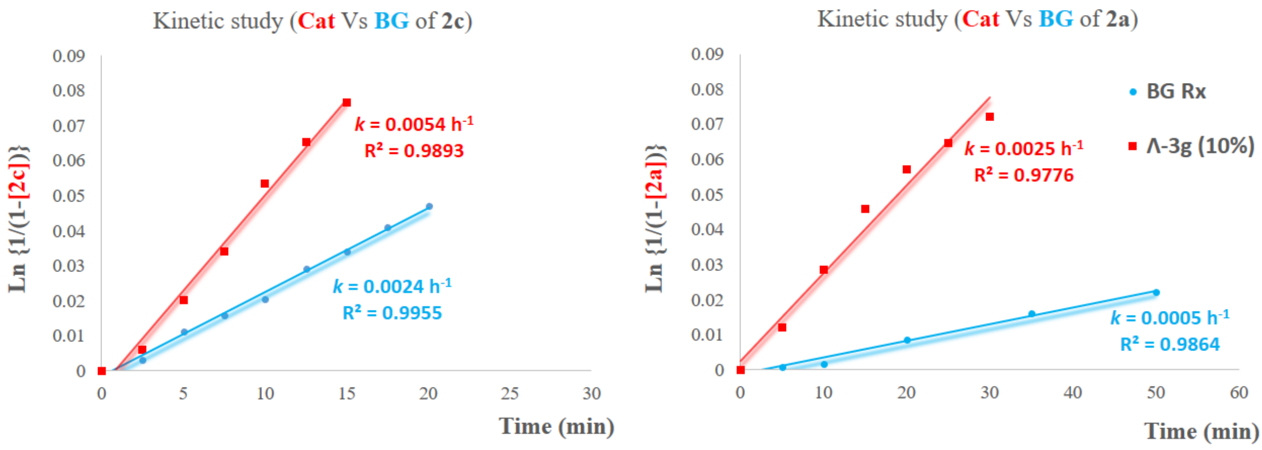

Figure S2. Kinetic Studies on the Iodocyclization Reaction of Enol Ethers: (a) Plotting Ln{1/(1-[**2c**])} vs time for both BG and catalytic pathways, (b) Plotting Ln{1/(1-[**2a**])} vs time for both BG and catalytic pathways to analyze the results and calculate the reaction rates *k*.

12. Stability of halocyclization products

Figure S3: stability of **2a** compound at rt and 50 °C

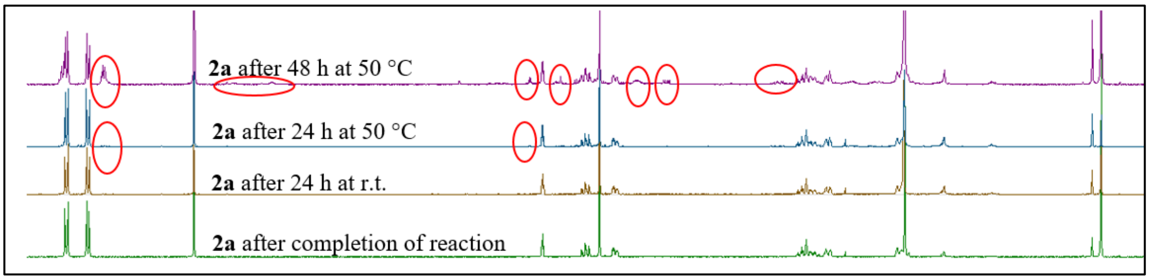

Figure S4: stability of **2e** compound compared to **2a** (**2a** is more stable compared to **2e**)

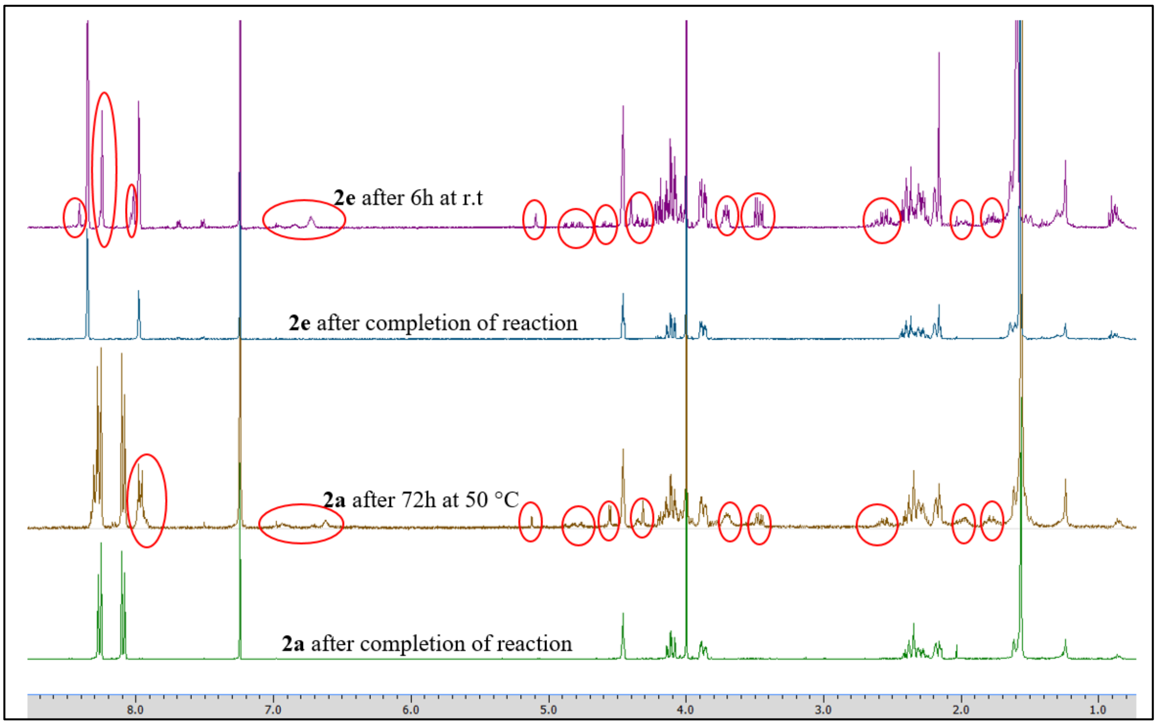

Figure S5: stability of **2e'** (bromocyclization product)

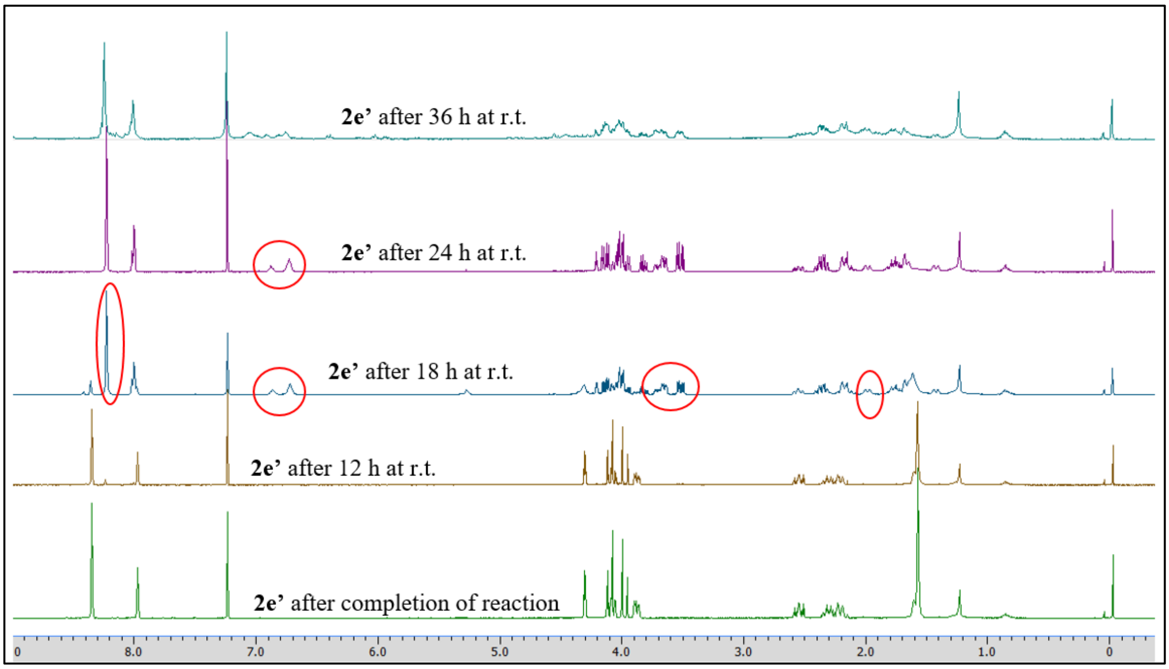

Figure S6: stability of **2e''** (chlorocyclization product) which is highly stable even at high temperatures

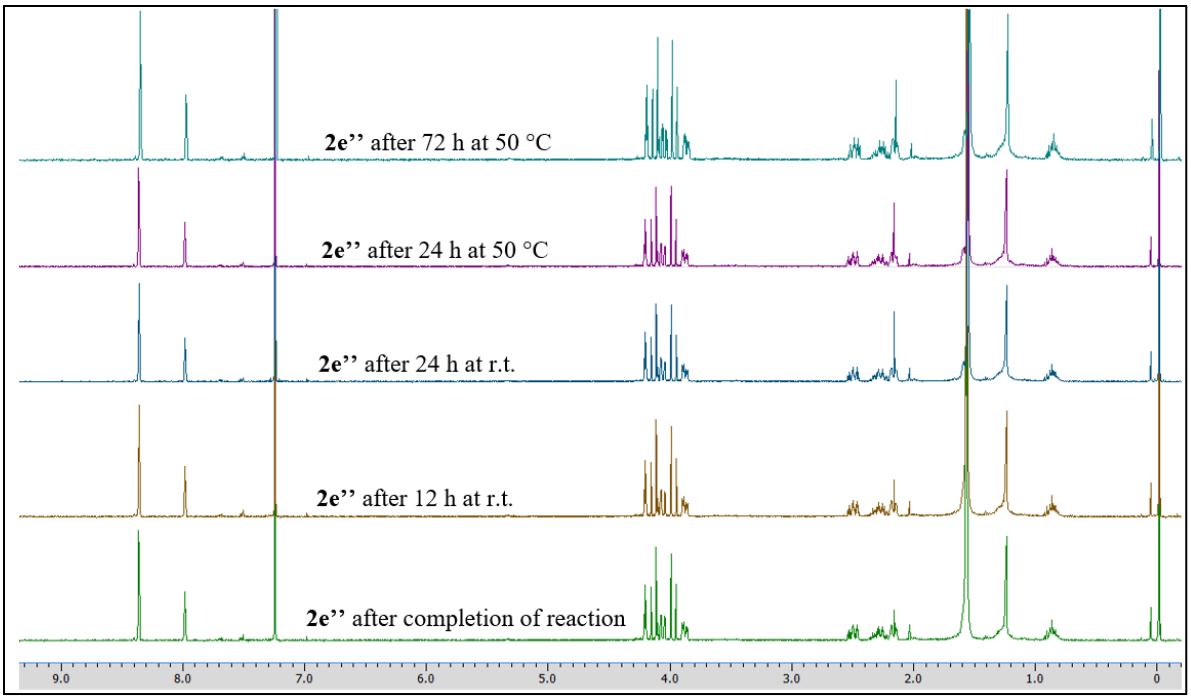

We believe that the higher stability of chloroderivatives compared to bromo- and iododerivatives can be explained by the smaller size of chlorine. After a preliminary study to elucidate the structures of the degradation products we could propose the following structures **A**, **B**, and **C** in which both **B** & **C** *in equilibria*

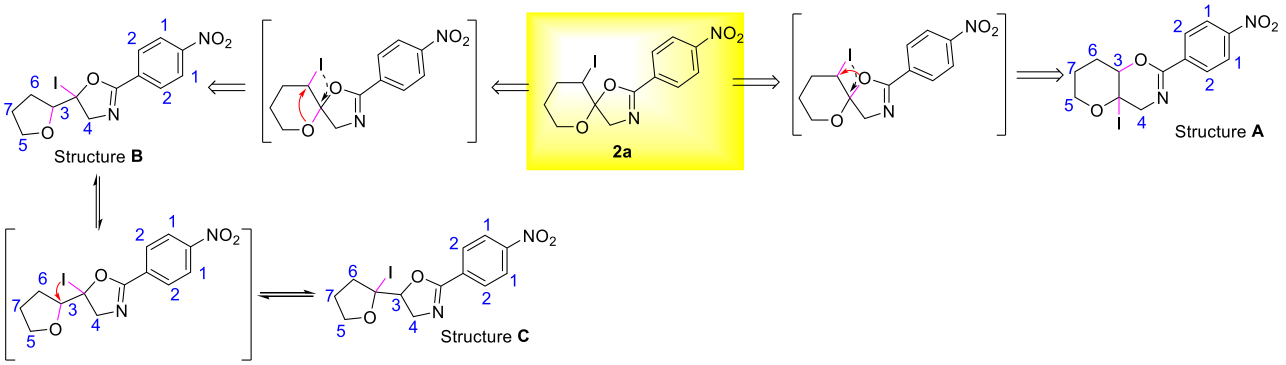

13. CD spectra

Assignment of Λ-(*S*,*S*,*S*,*S*)-**3f** configuration compared to the previously reported Λ-(*S*,*S*)-**3d**

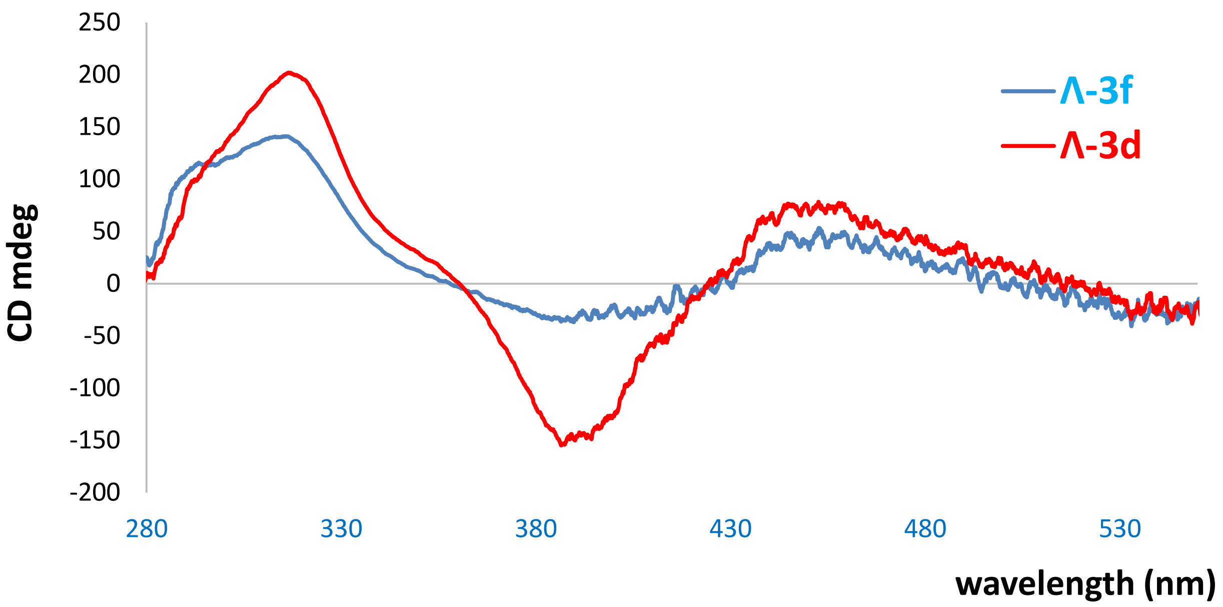

14. References

Belokon, Y.N., Maleev, V.I., Kataev, D.A., Mal'fanov, I.L., Bulychev, A.G., Moskalenko, M.A., Tat'yana, F.S., Tat'yana, V.S., Lyssenko, K.A., Godovikov, I.A., and North, M. (2008). Potassium and silver chiral cobaltate (III) complexes as precatalysts for asymmetric C–C bond formation. *Tetrahedron: Asymmetry* 19(7), 822-831.

Belokon, Y.N., Maleev, V.I., Kataev, D.A., Saveleva, T.F., Skrupskaya, T.V., Nelyubina, Y.V., and North, M. (2009). Chiral ion pairs in catalysis: lithium salts of chiral metallocomplex anions as catalysts for asymmetric C–C bond formation. *Tetrahedron: Asymmetry* 20(15)**,** 1746-1752.

Jiang, H.J., Liu, K., Yu, J., Zhang, L., and Gong, L.Z. (2017). Switchable stereoselectivity in bromoaminocyclization of olefins: using Brønsted acids of anionic chiral cobalt (III) complexes. *Angewandte Chemie* 129(39)**,** 12093-12097.

Li, N., Yu, H., Wang, R., Shen, J., Wu, W.-Q., Liu, K., Sun, T.-T., Zhang, Z.-Z., Yao, C.-Z., and Yu, J. (2018). Enantioselective intermolecular iodoacetalization of enol ethers catalyzed by chiral Co (III)-complex-templated Brønsted acids. *Tetrahedron Letters* 59(40)**,** 3605-3608.

Rauniyar, V., Lackner, A.D., Hamilton, G.L., and Toste, F.D. (2011). Asymmetric electrophilic fluorination using an anionic chiral phase-transfer catalyst. *Science* 334(6063)**,** 1681-1684.

Yu, .J, Jiang, H.J., Zhou, Y., Luo, S.W., and Gong, L.Z. (2015). Sodium salts of anionic chiral cobalt (III) complexes as catalysts of the enantioselective Povarov reaction. *Angewandte Chemie* 127(38), 11361-11365.

15. NMR Spectra

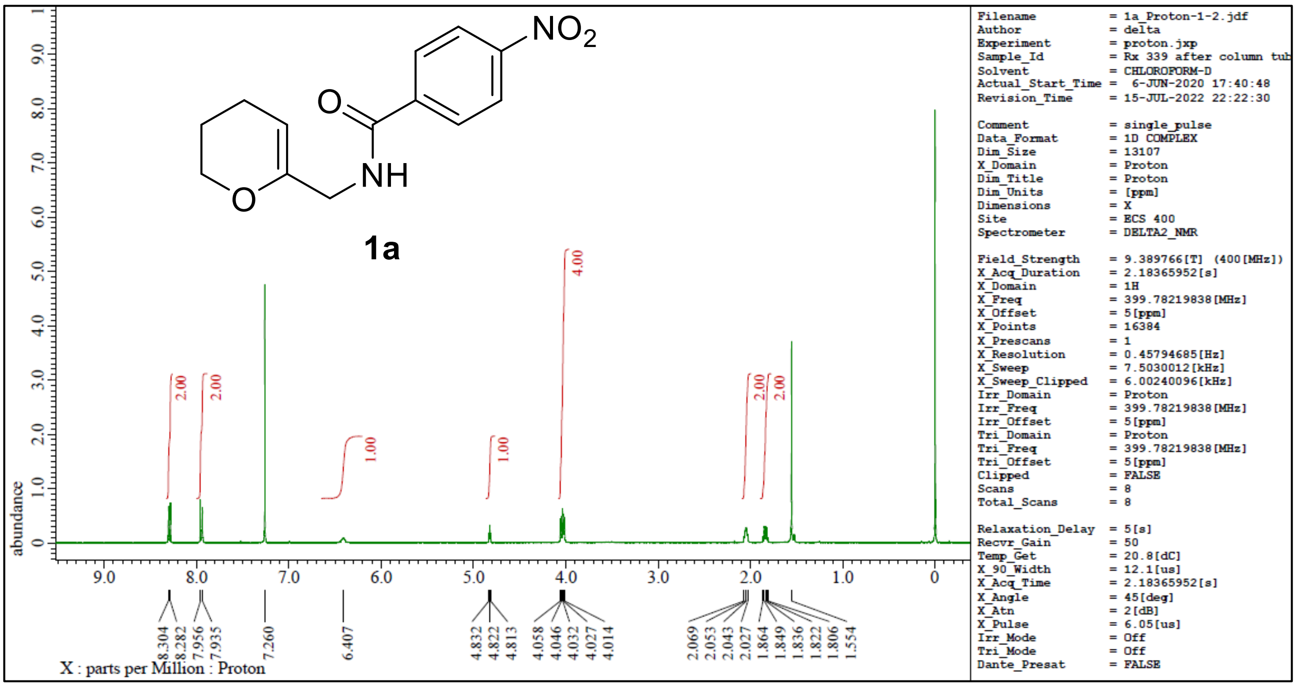

Compound **1a** (^{1}H NMR, 400 MHz, $CDCl_3$).

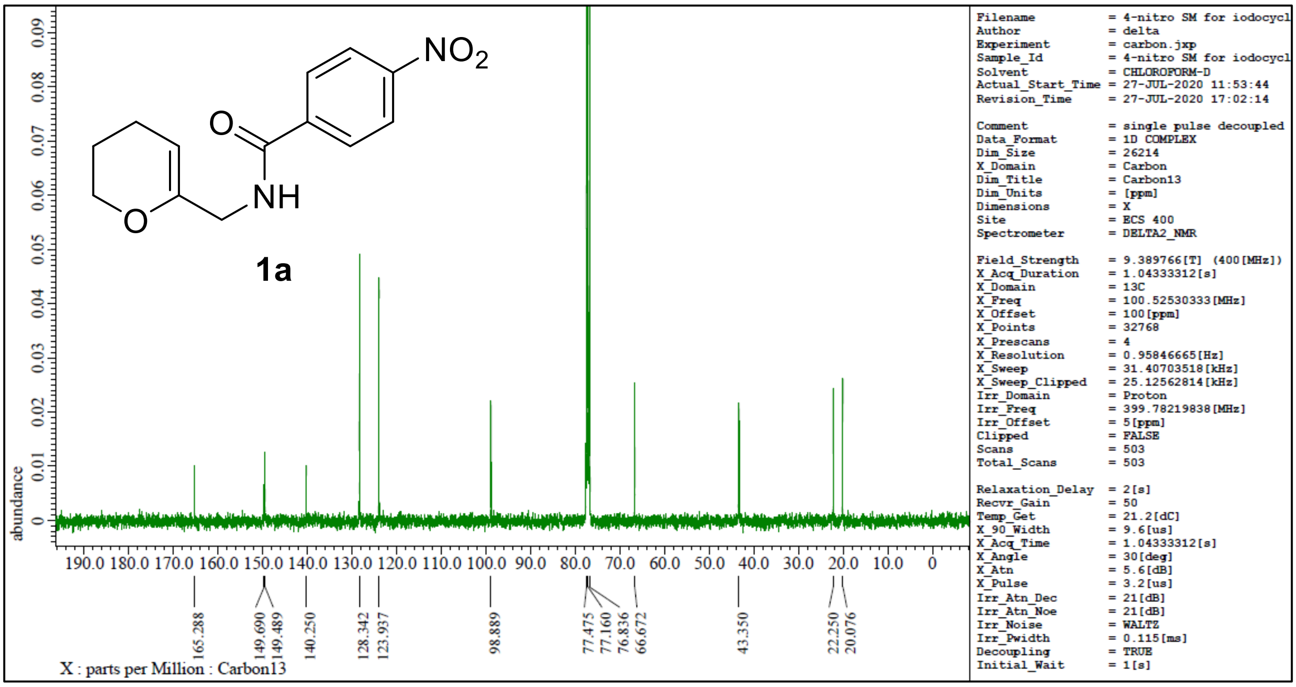

Compound **1a** (^{13}C NMR, 100 MHz, $CDCl_3$).

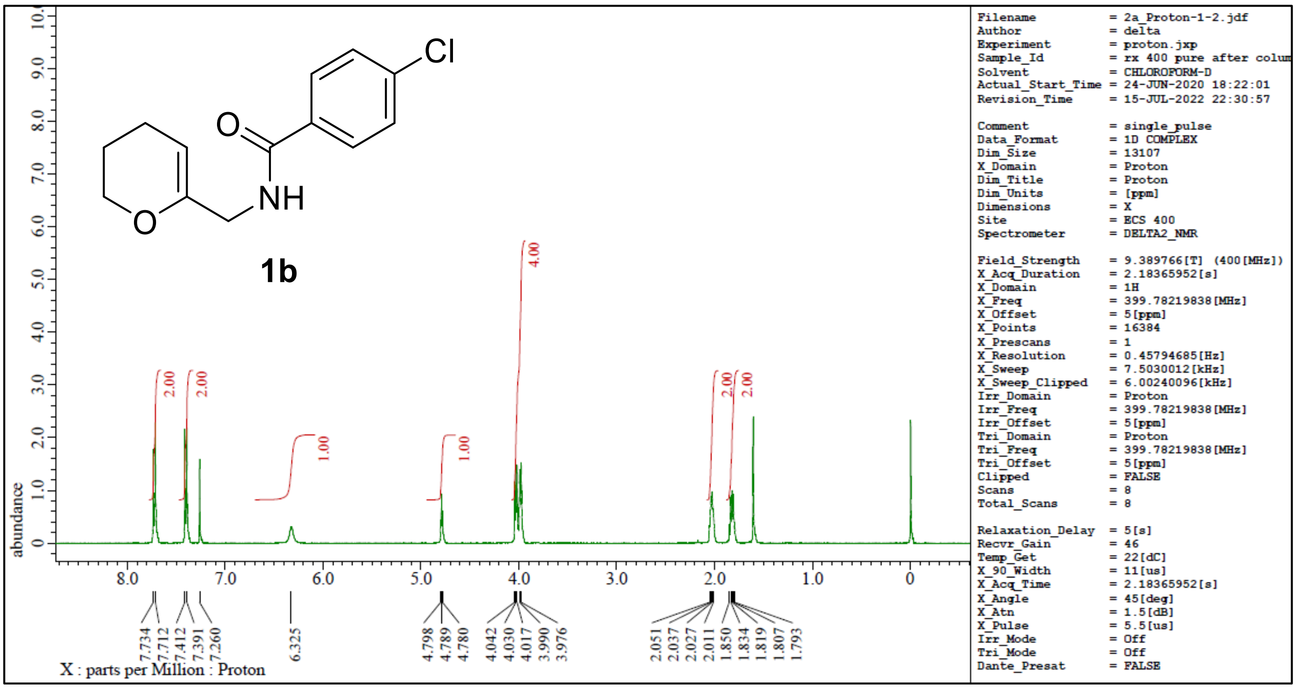

Compound **1b** (^{1}H NMR, 400 MHz, $CDCl_3$).

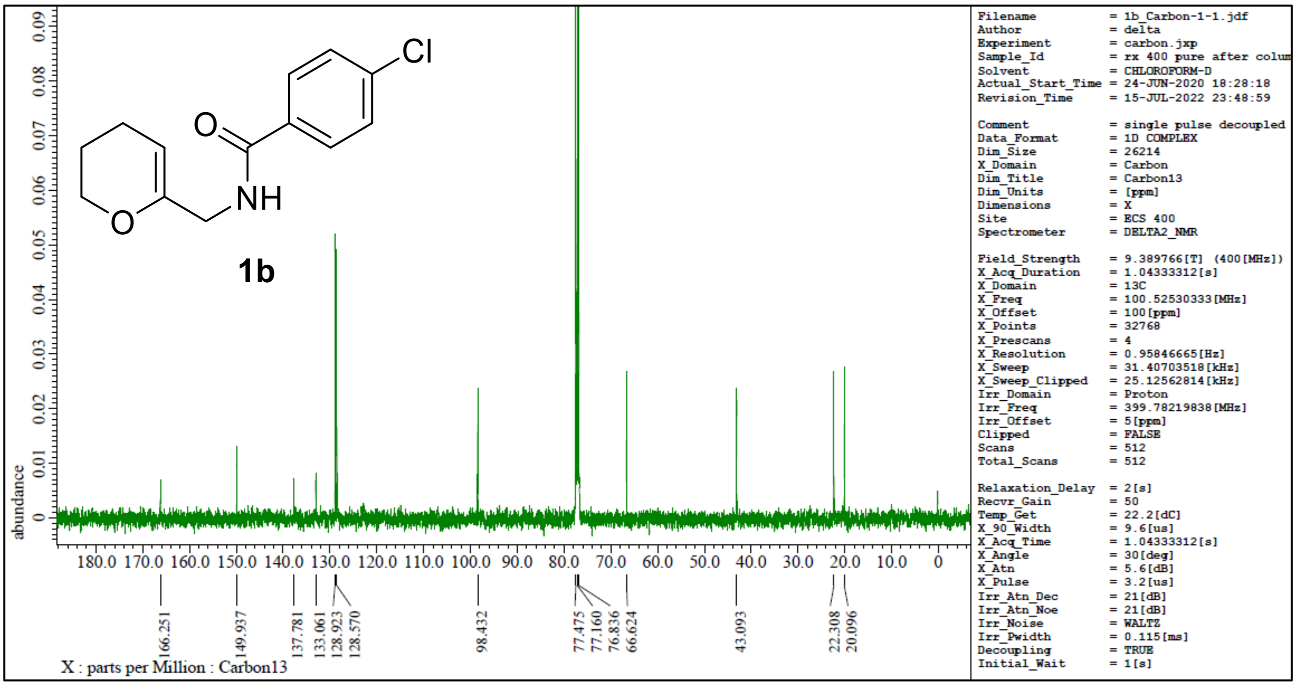

Compound **1b** (^{13}C NMR, 100 MHz, $CDCl_3$).

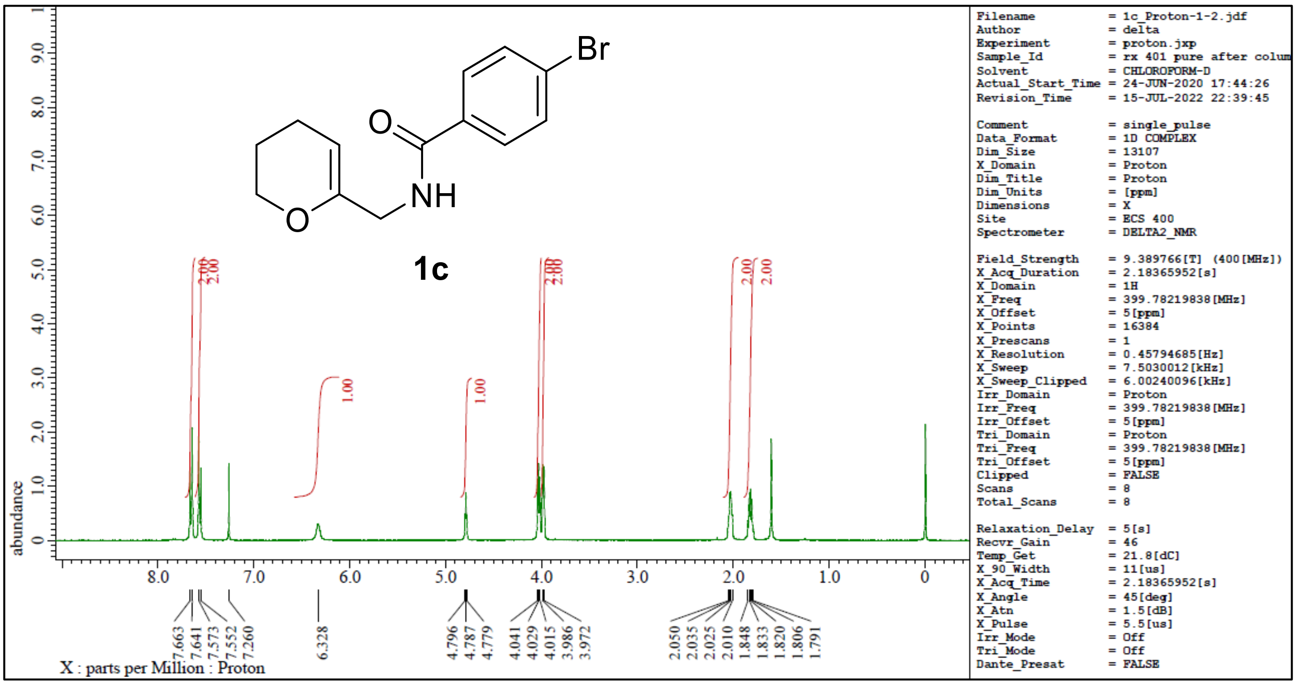

Compound **1c** (^{1}H NMR, 400 MHz, $CDCl_3$).

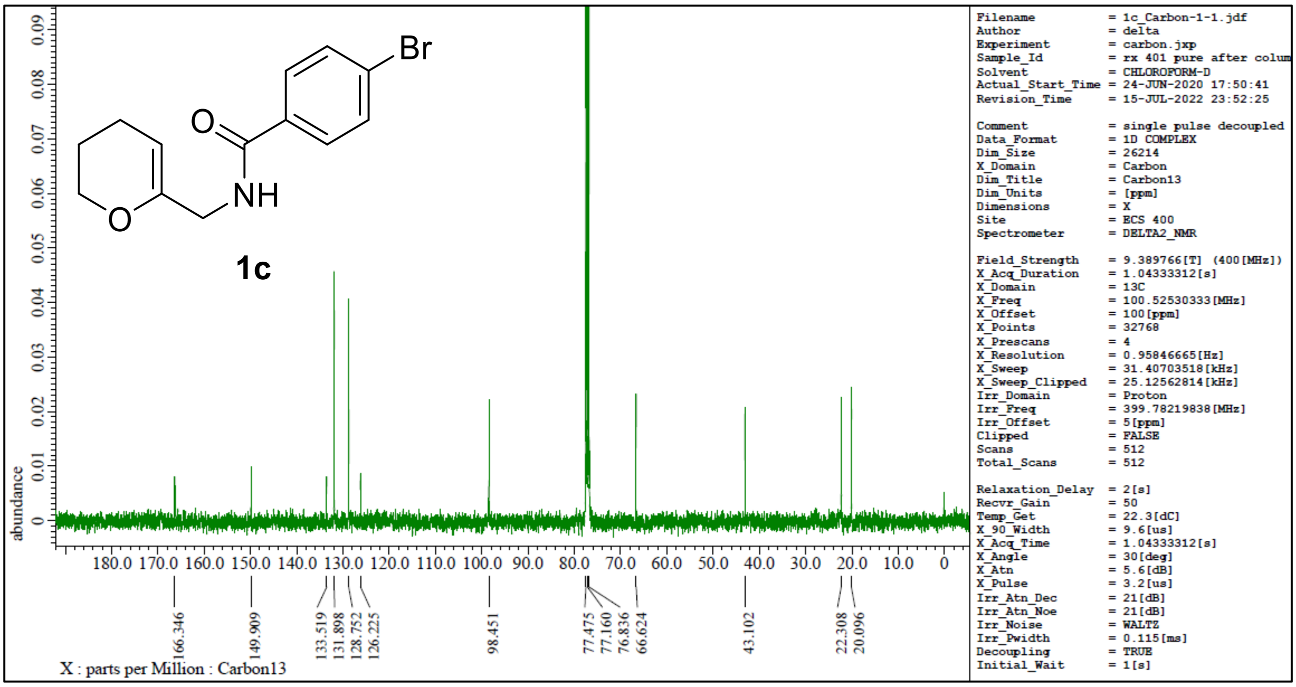

Compound **1c** (^{13}C NMR, 100 MHz, $CDCl_3$).

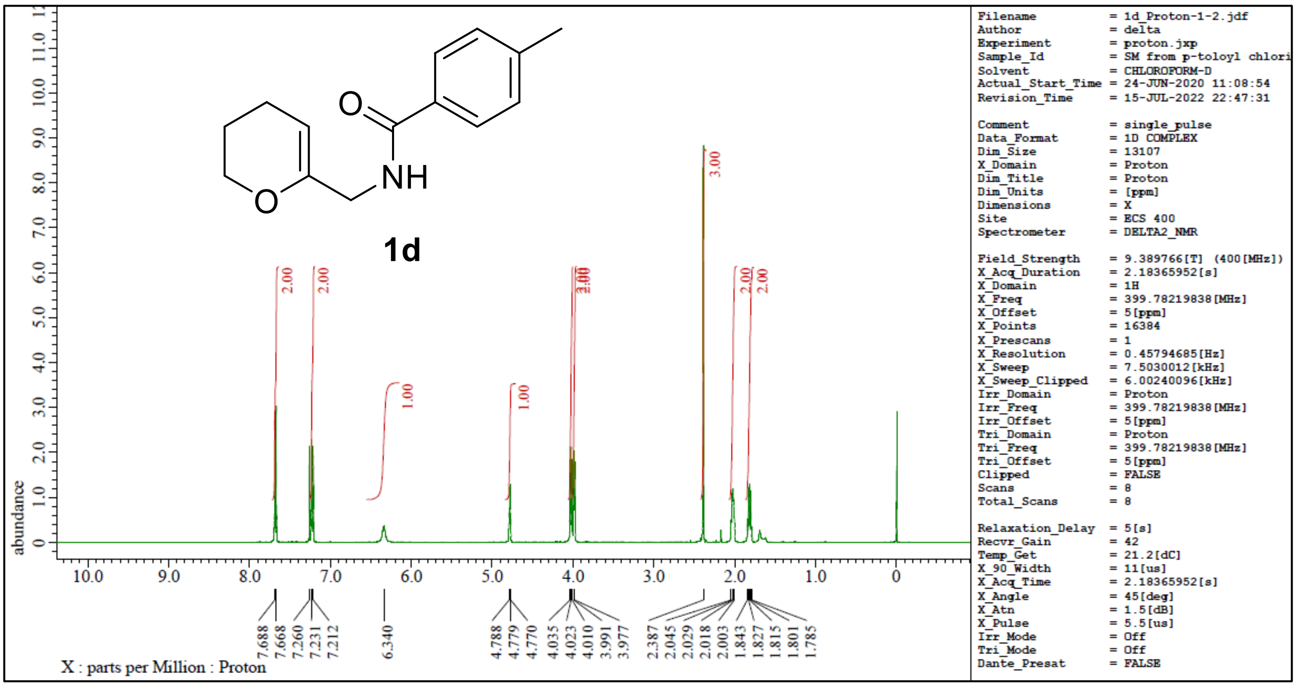

Compound **1d** (^{1}H NMR, 400 MHz, $CDCl_3$).

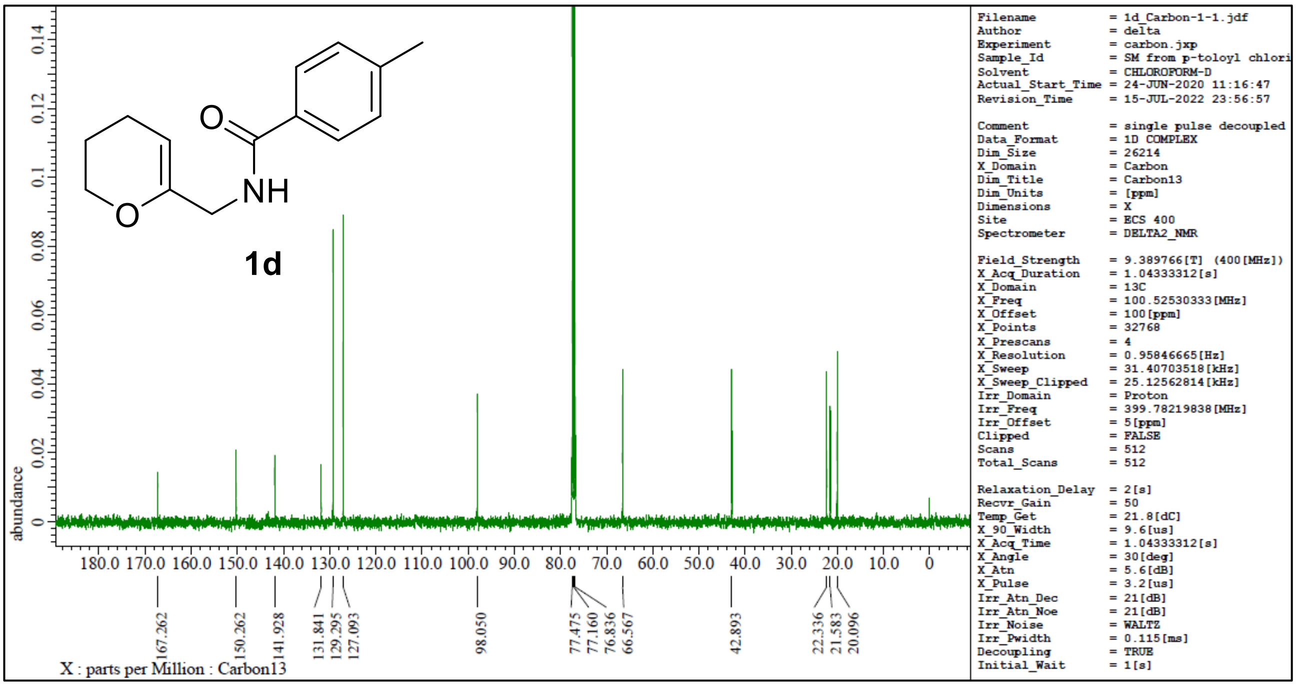

Compound **1d** (^{13}C NMR, 100 MHz, $CDCl_3$).

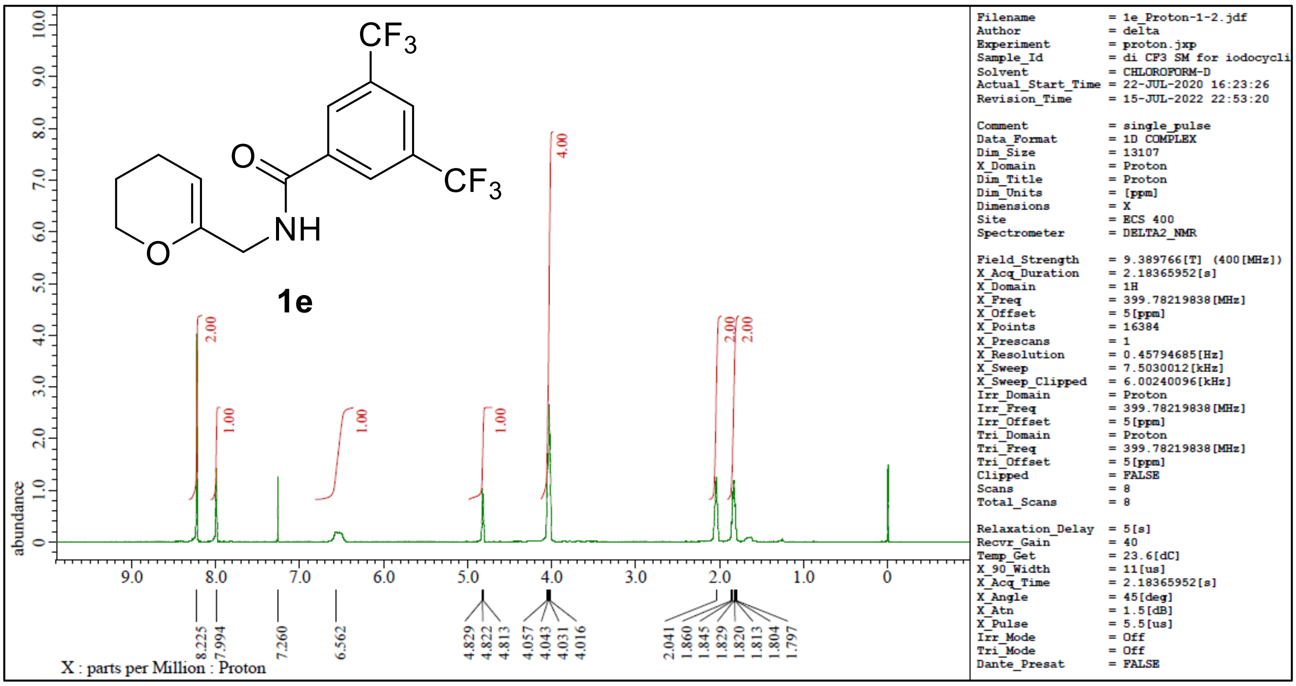

Compound **1e** (^{1}H NMR, 400 MHz, $CDCl_3$).

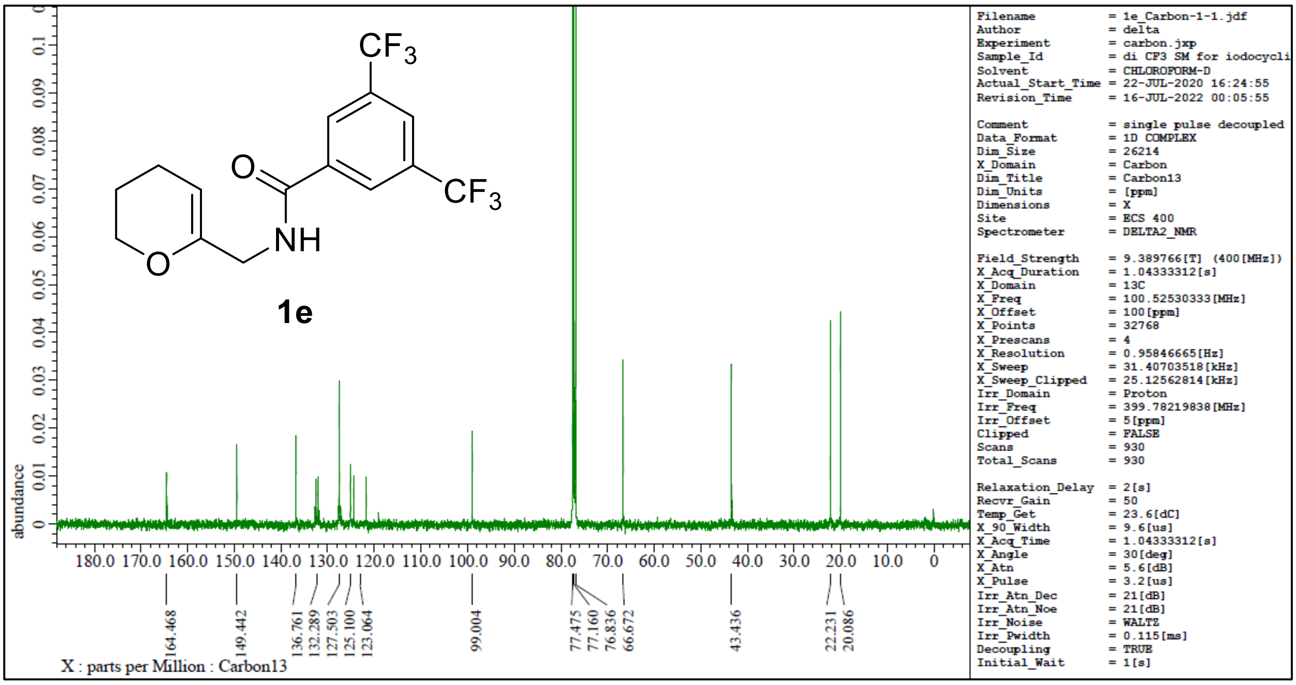

Compound **1e** (^{13}C NMR, 100 MHz, $CDCl_3$).

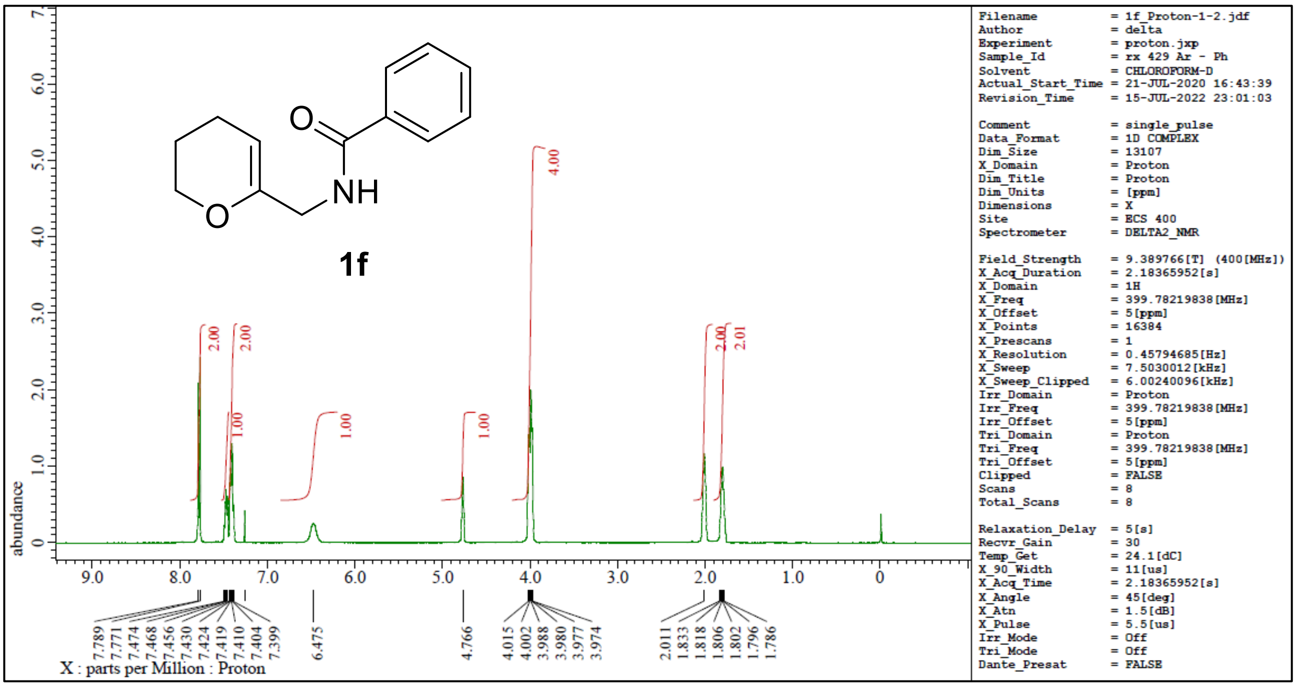

Compound **1f** (^{1}H NMR, 400 MHz, $CDCl_3$).

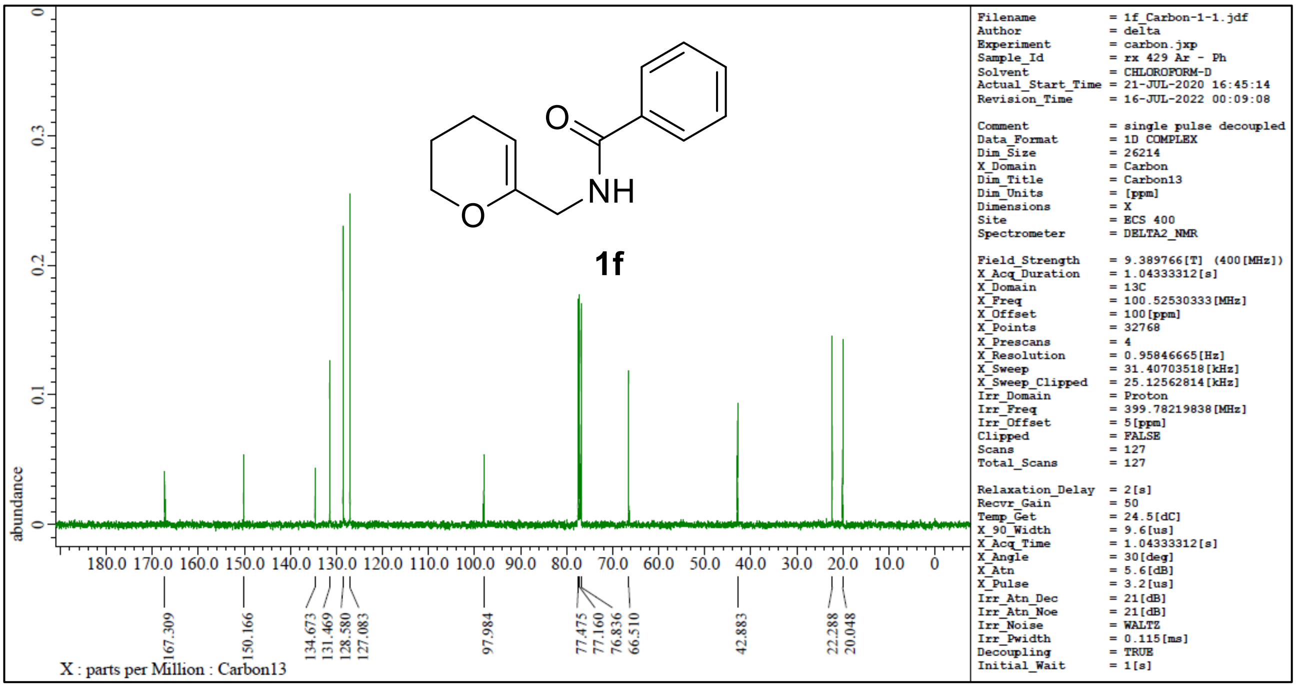

Compound **1f** (^{13}C NMR, 100 MHz, $CDCl_3$).

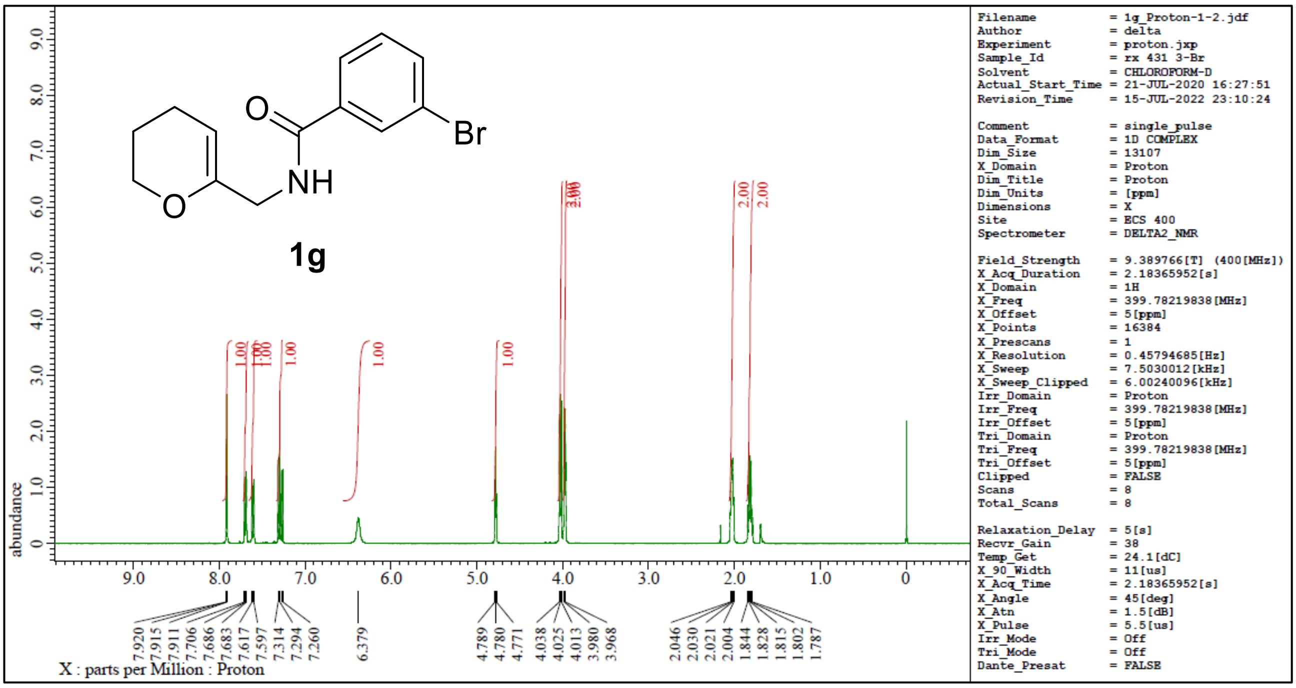

Compound **1g** (^{1}H NMR, 400 MHz, $CDCl_3$).

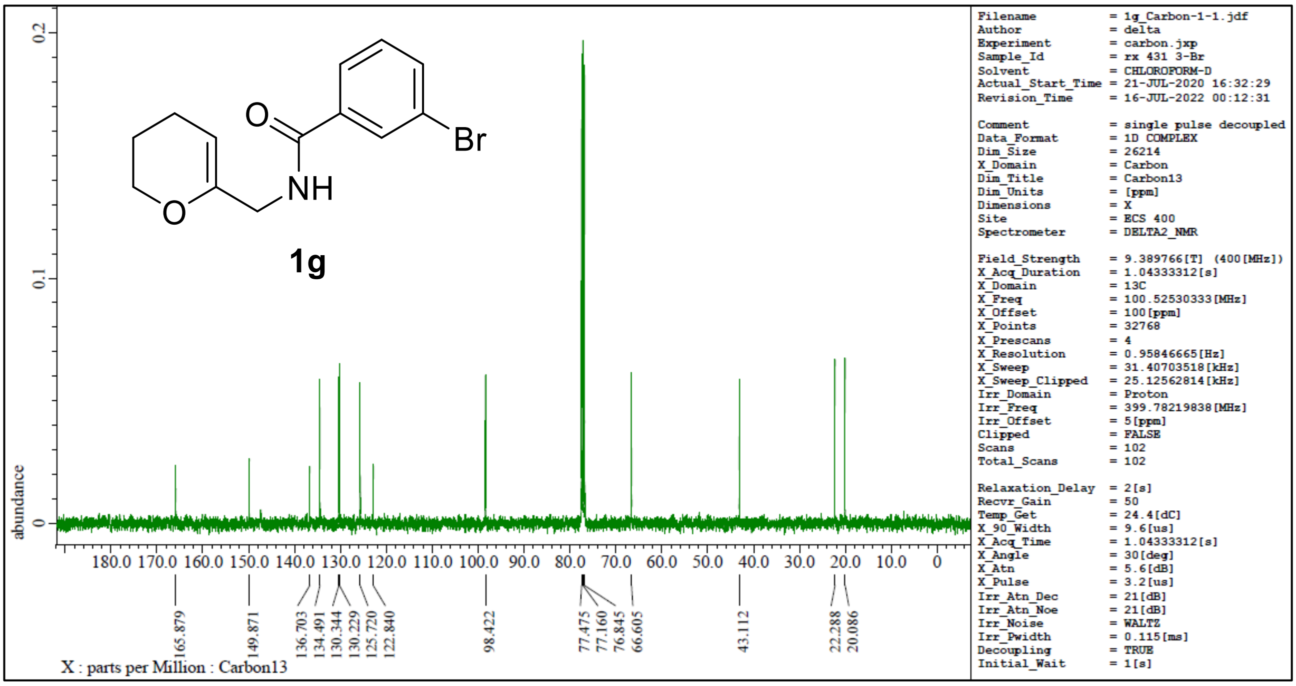

Compound **1g** (^{13}C NMR, 100 MHz, $CDCl_3$).

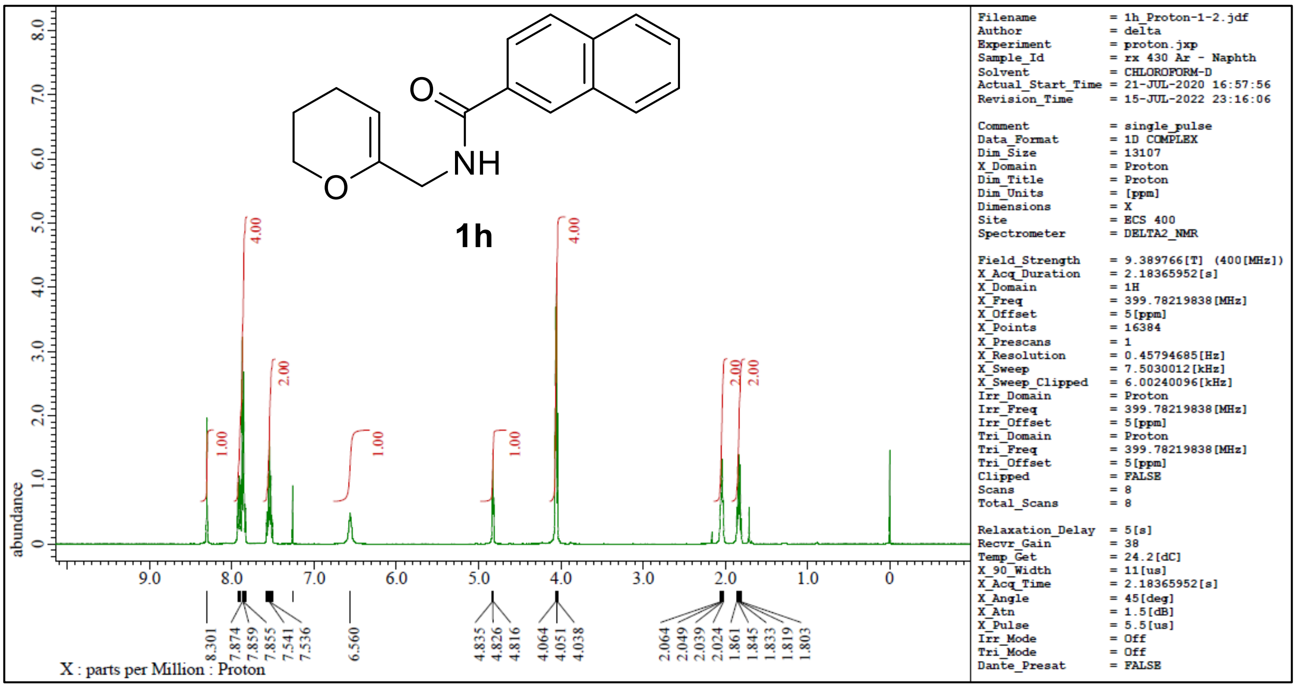

Compound **1h** (^{1}H NMR, 400 MHz, $CDCl_3$).

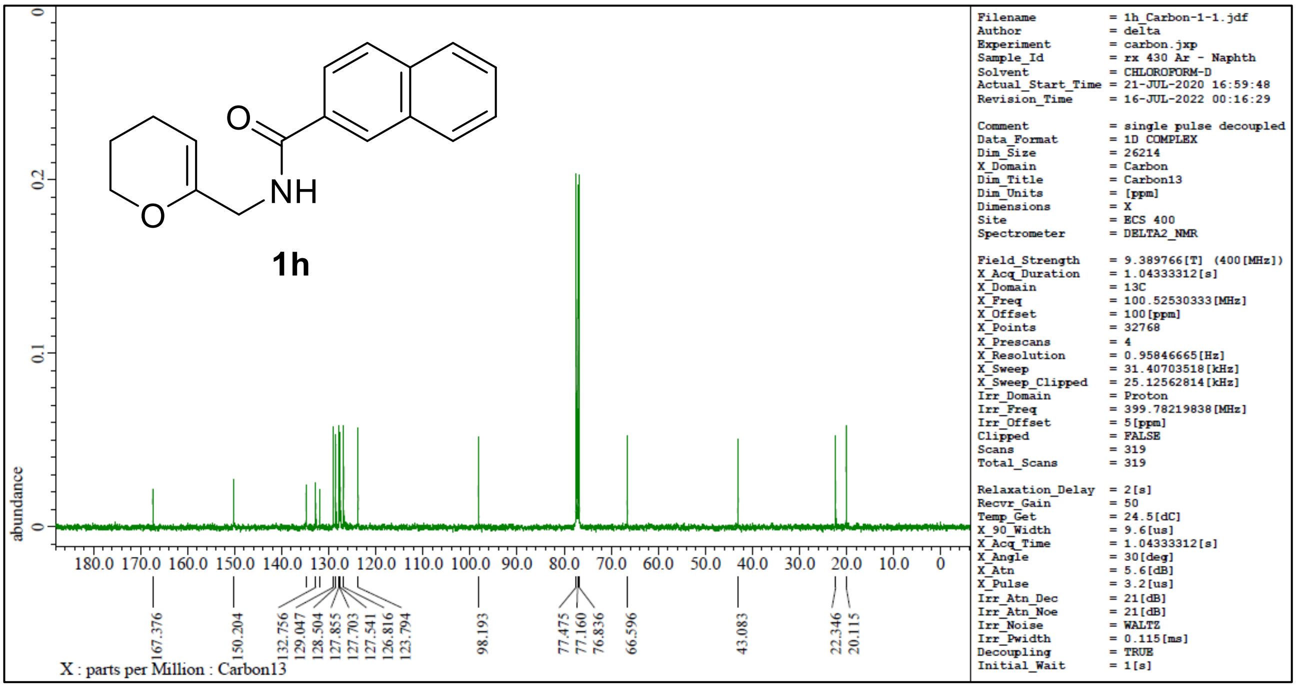

Compound **1h** (^{13}C NMR, 100 MHz, $CDCl_3$).

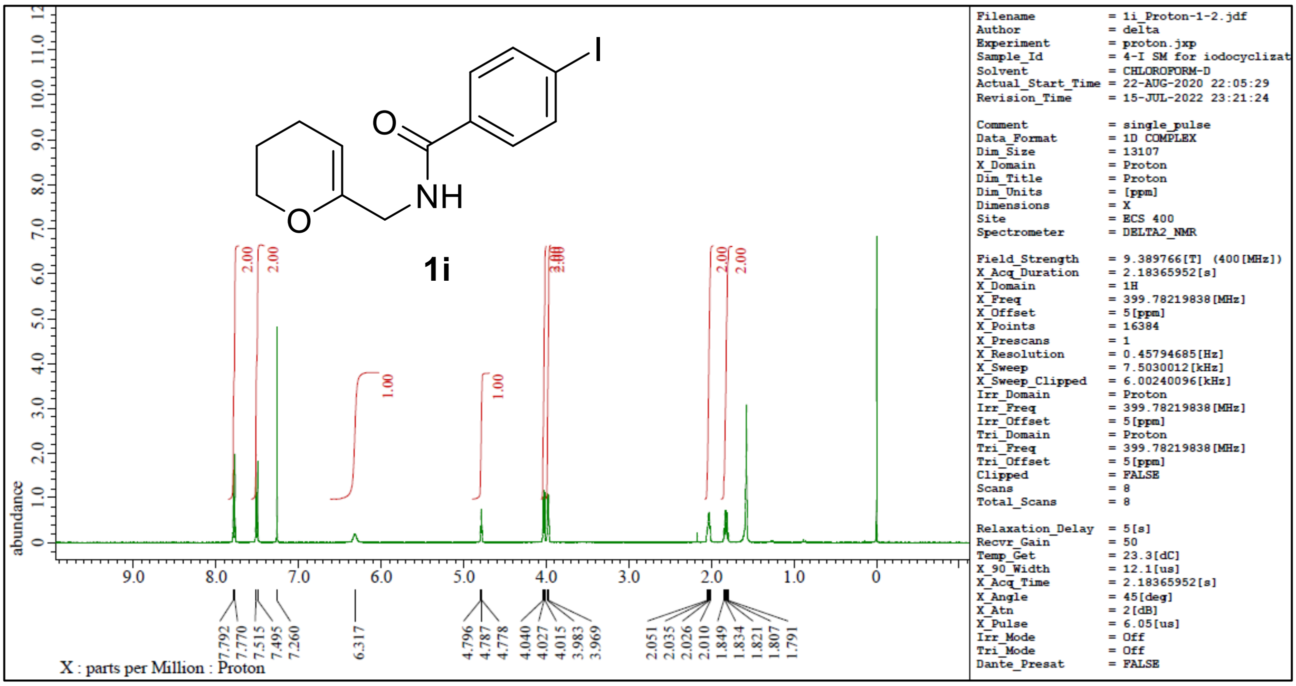

Compound **1i** (^{1}H NMR, 400 MHz, $CDCl_3$).

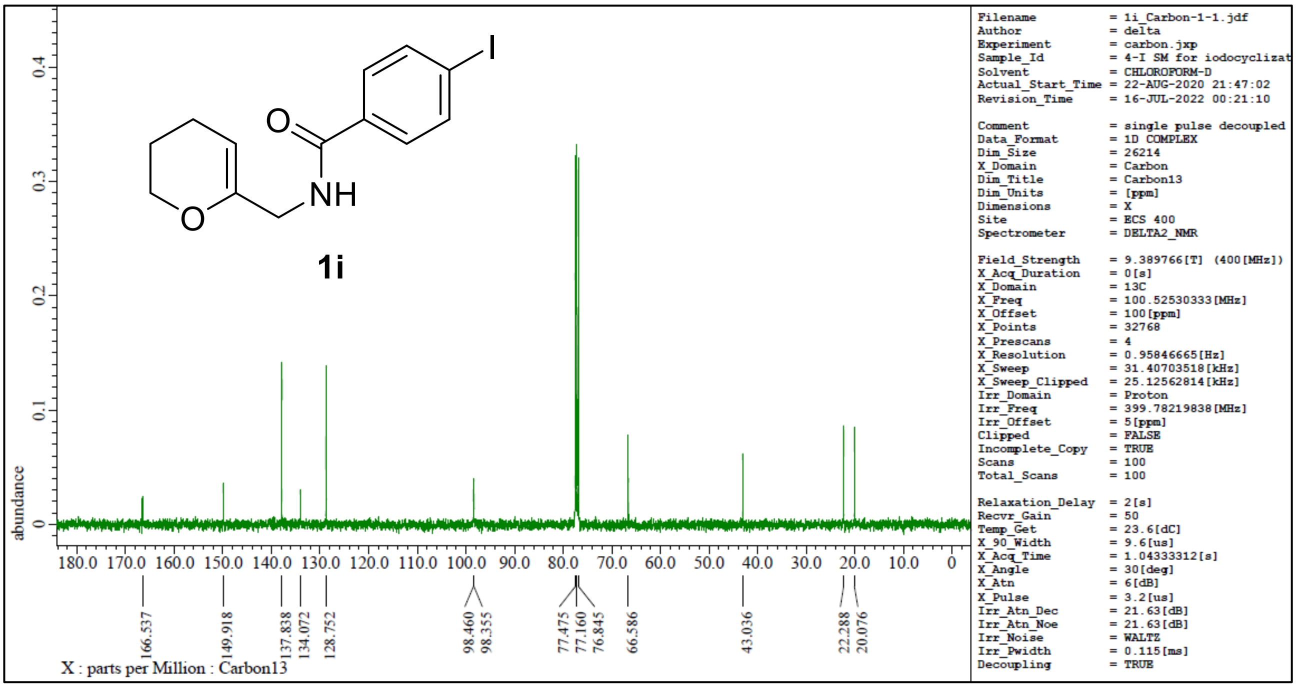

Compound **1i** (^{13}C NMR, 100 MHz, $CDCl_3$).

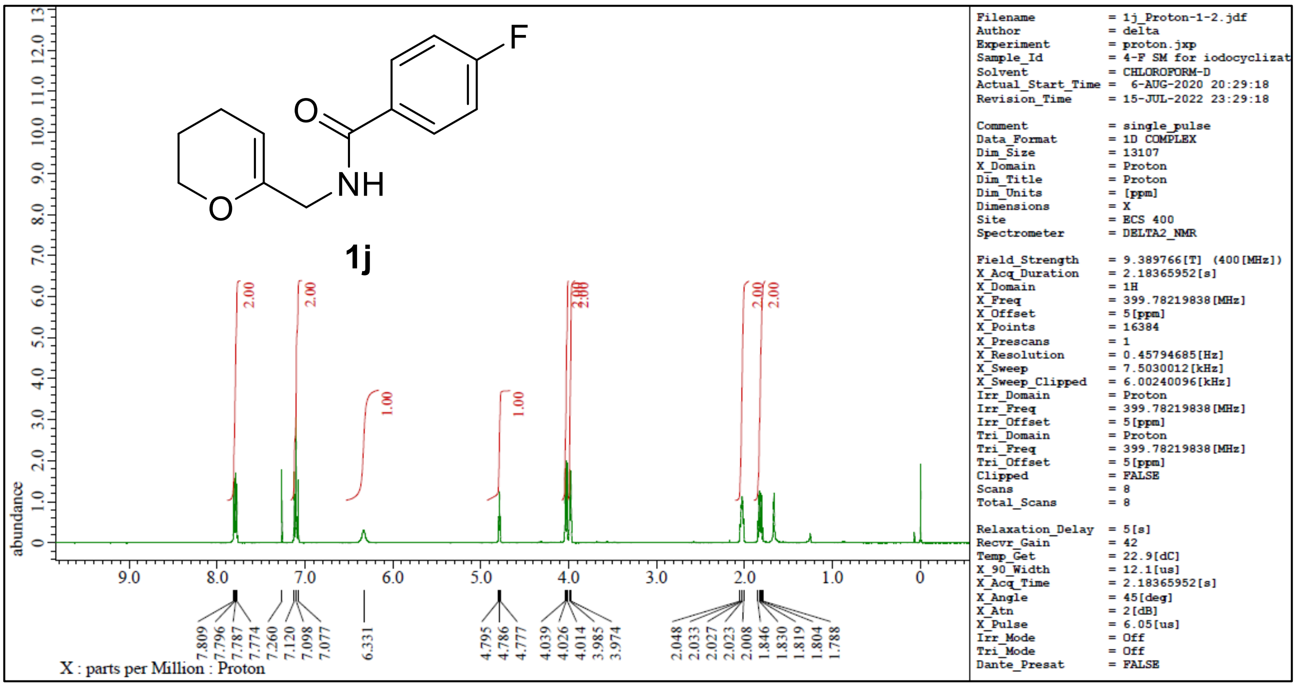

Compound **1j** (^{1}H NMR, 400 MHz, $CDCl_3$).

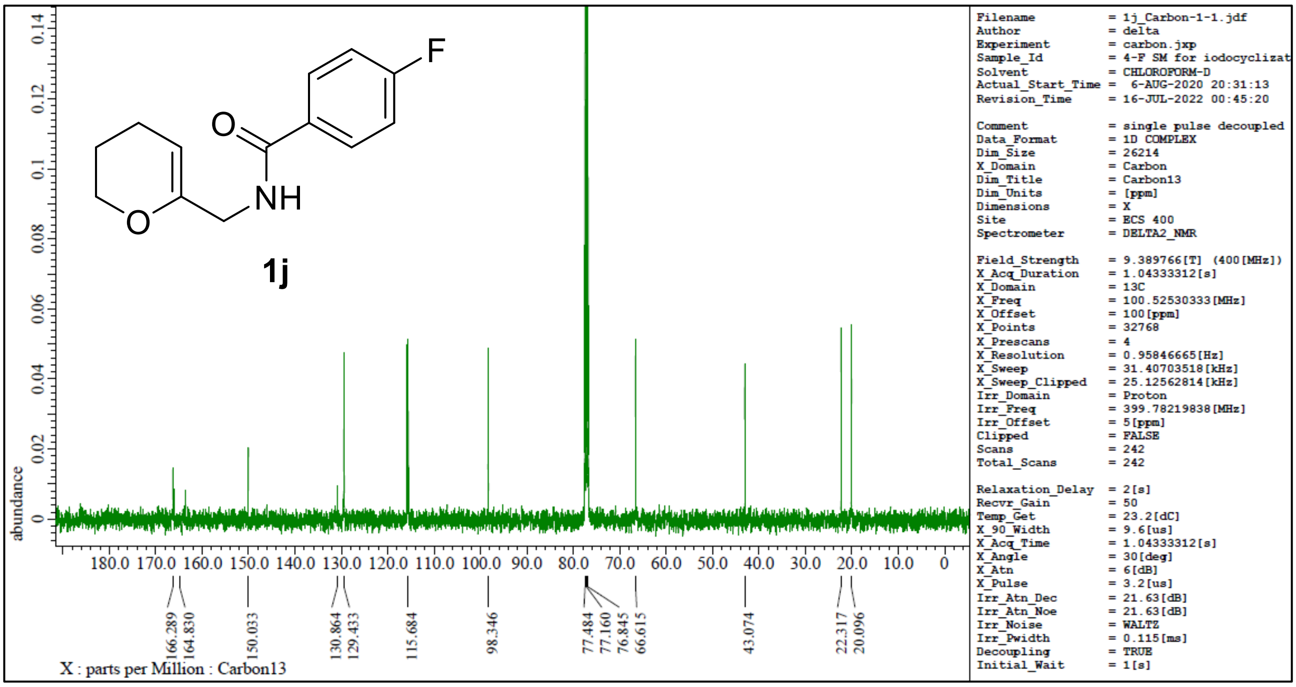

Compound **1j** (^{13}C NMR, 100 MHz, $CDCl_3$).

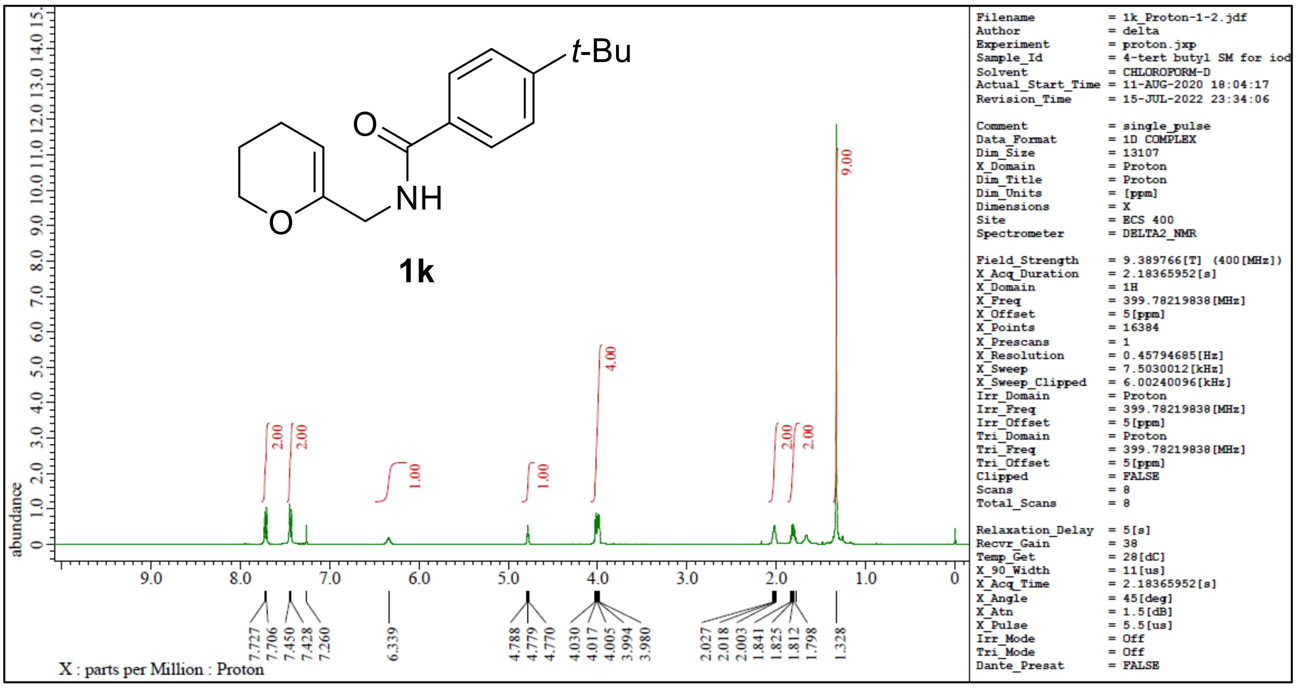

Compound **1k** (^{1}H NMR, 400 MHz, $CDCl_3$).

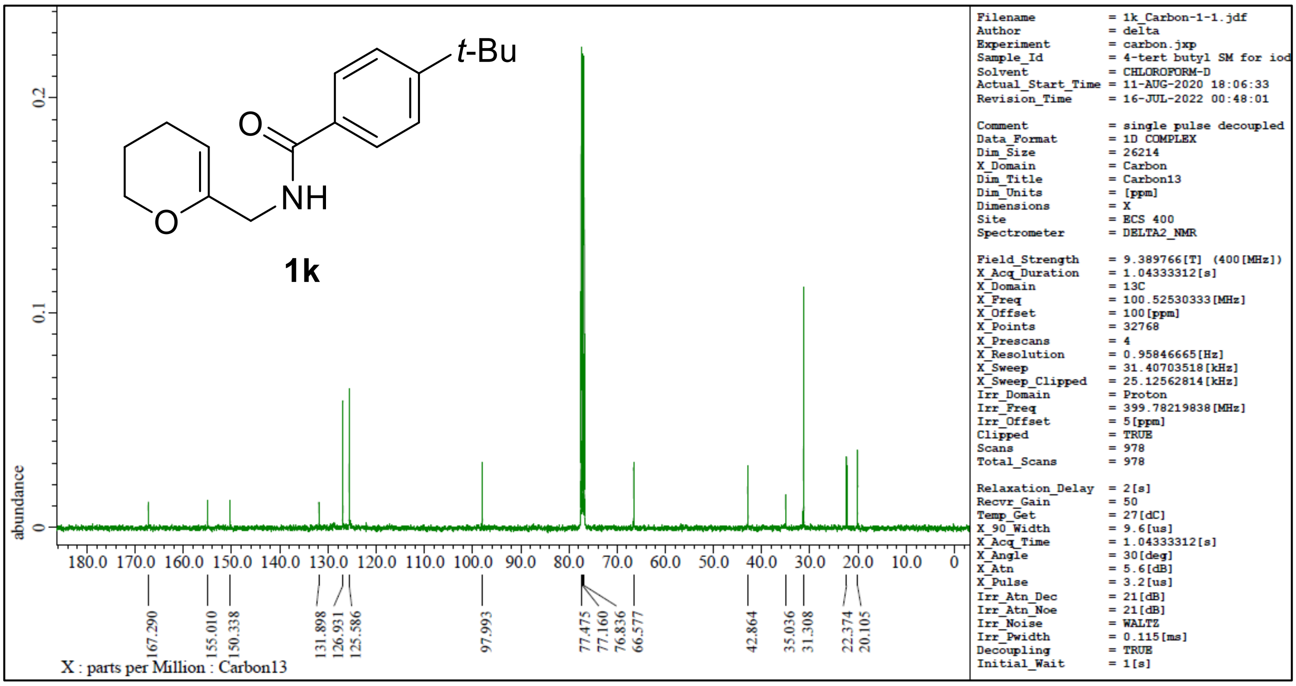

Compound **1k** (^{13}C NMR, 100 MHz, $CDCl_3$).

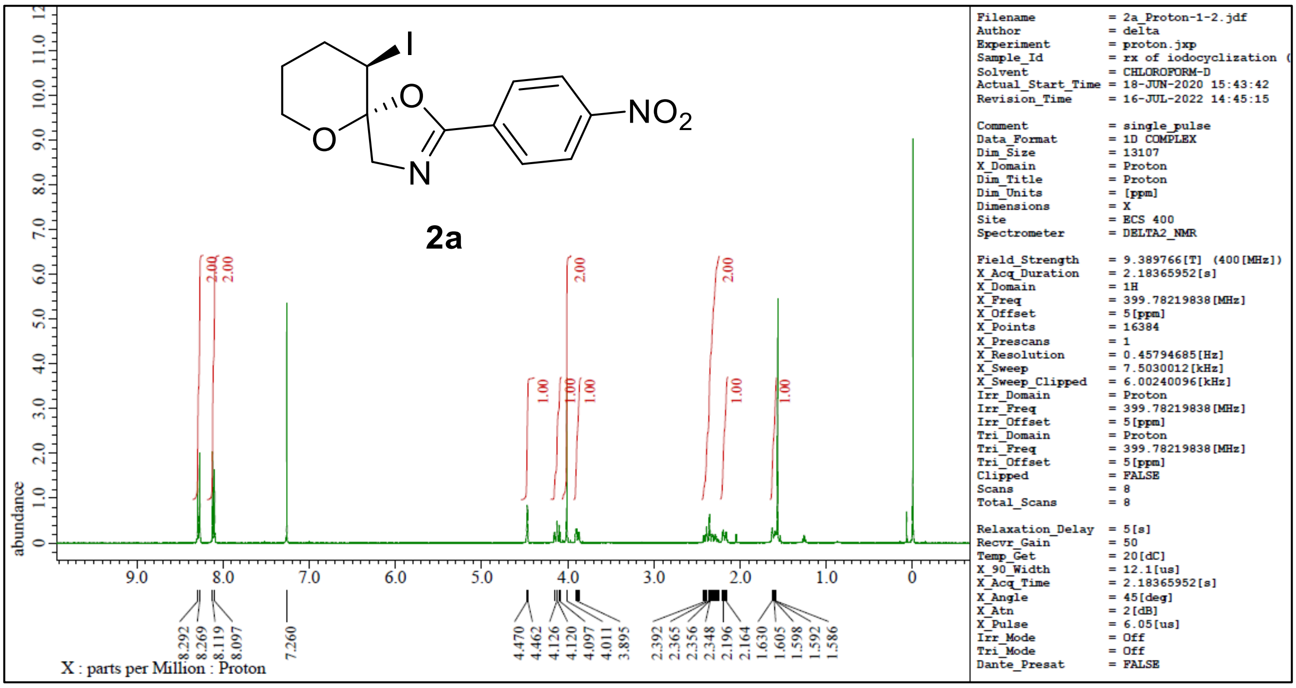

Compound **2a** (^{1}H NMR, 400 MHz, $CDCl_3$).

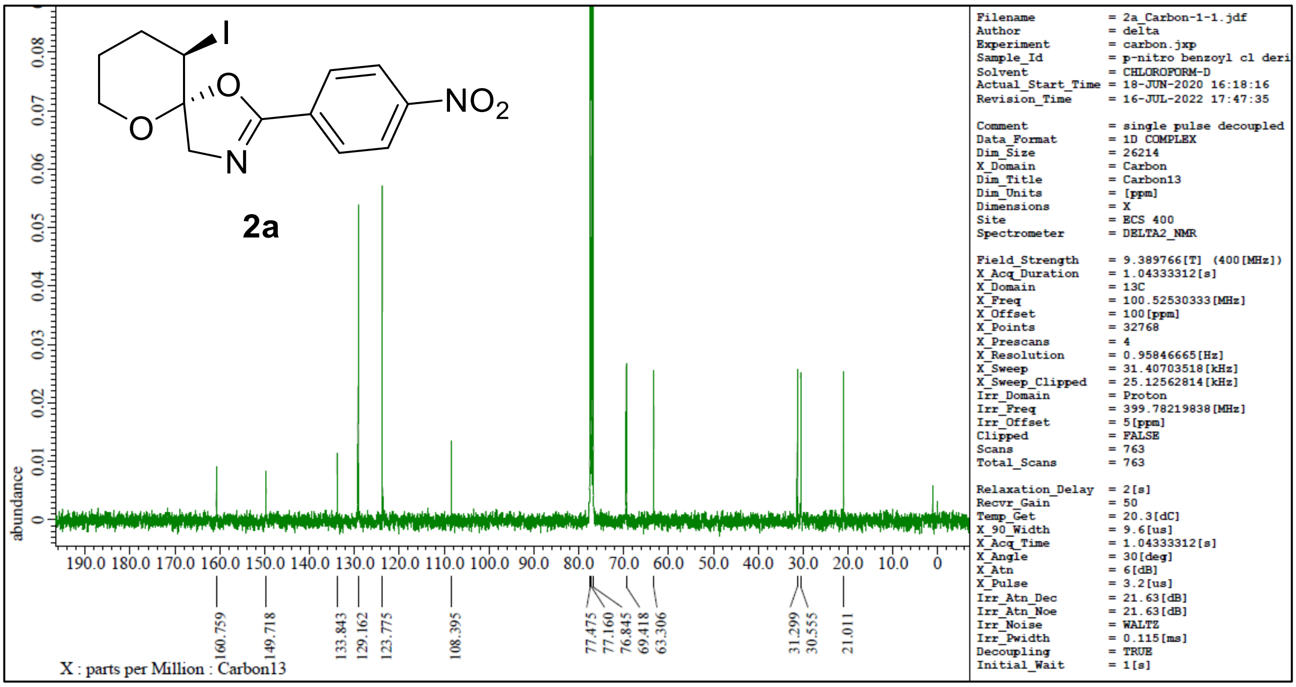

Compound **2a** (^{13}C NMR, 100 MHz, $CDCl_3$).

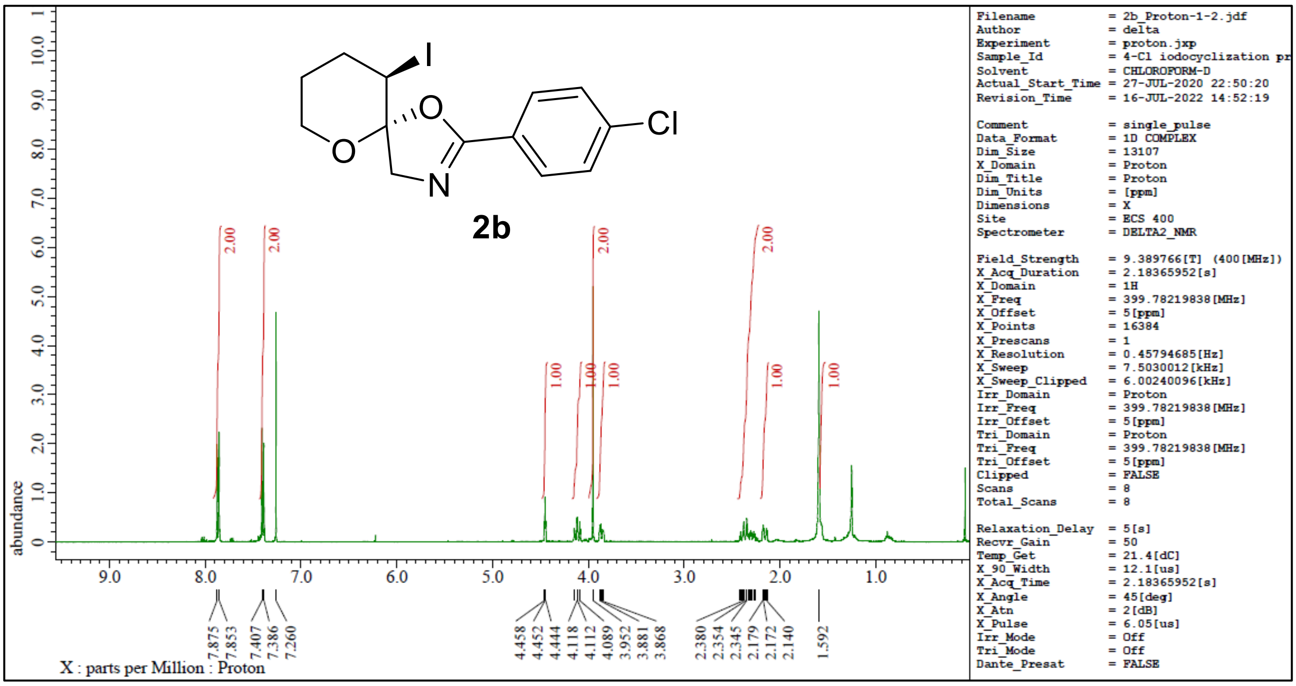

Compound **2b** (^{1}H NMR, 400 MHz, $CDCl_3$).

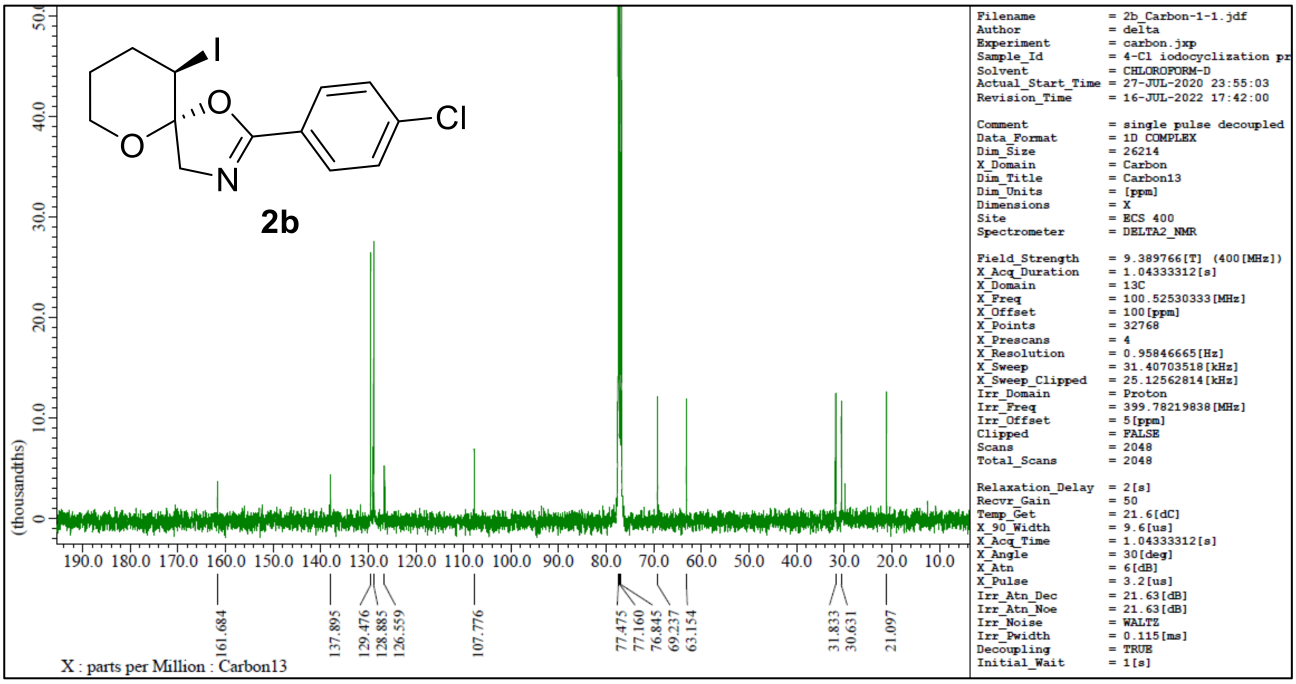

Compound **2b** (^{13}C NMR, 100 MHz, $CDCl_3$).

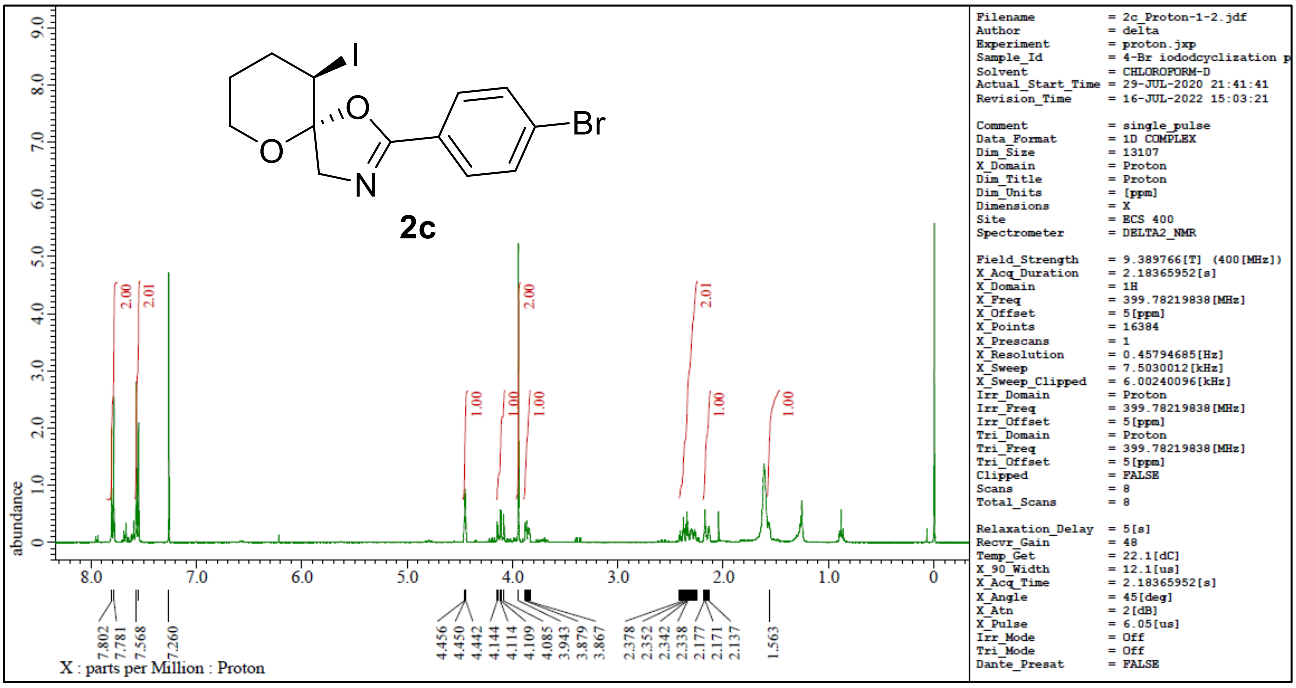

Compound **2c** (^{1}H NMR, 400 MHz, $CDCl_3$).

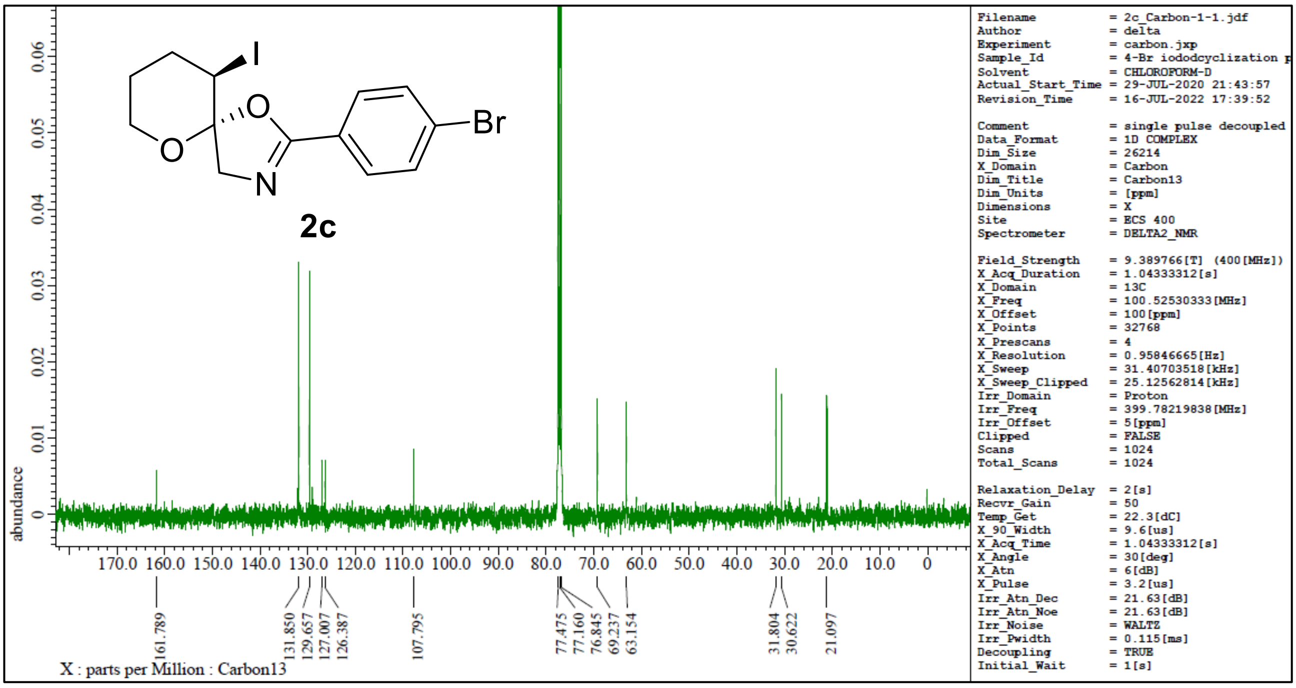

Compound **2c** (^{13}C NMR, 100 MHz, $CDCl_3$).

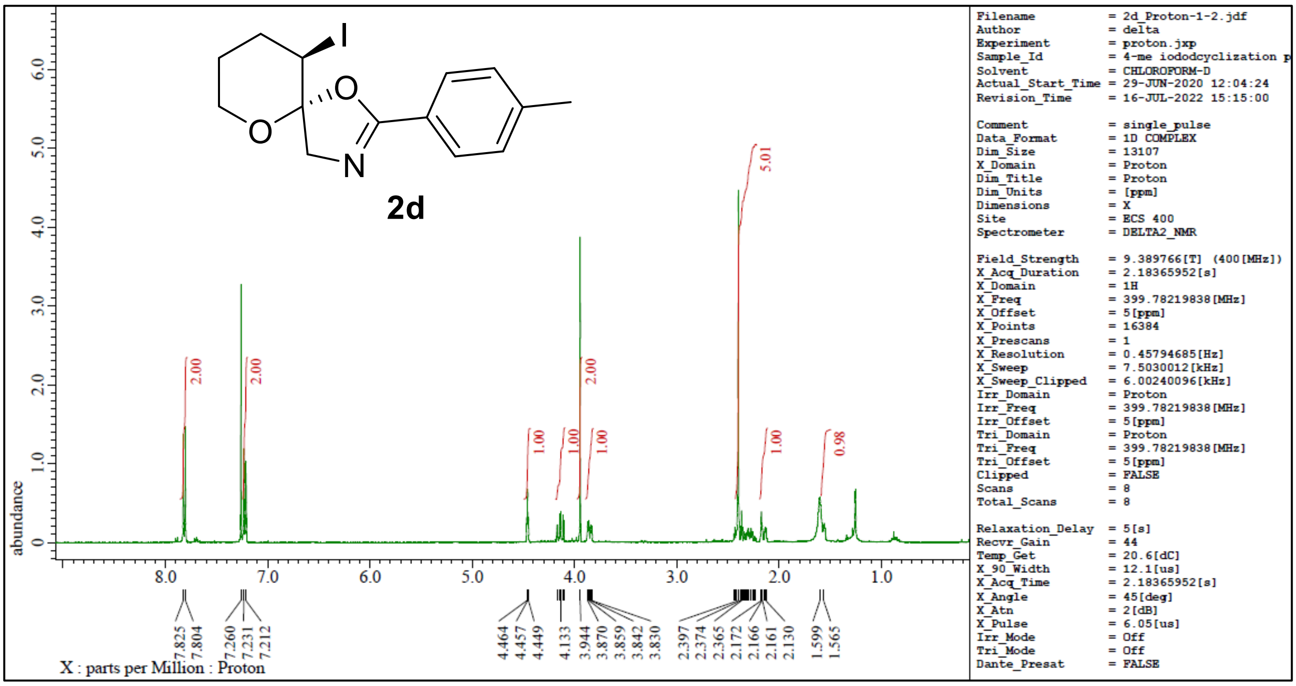

Compound **2d** (^{1}H NMR, 400 MHz, $CDCl_3$).

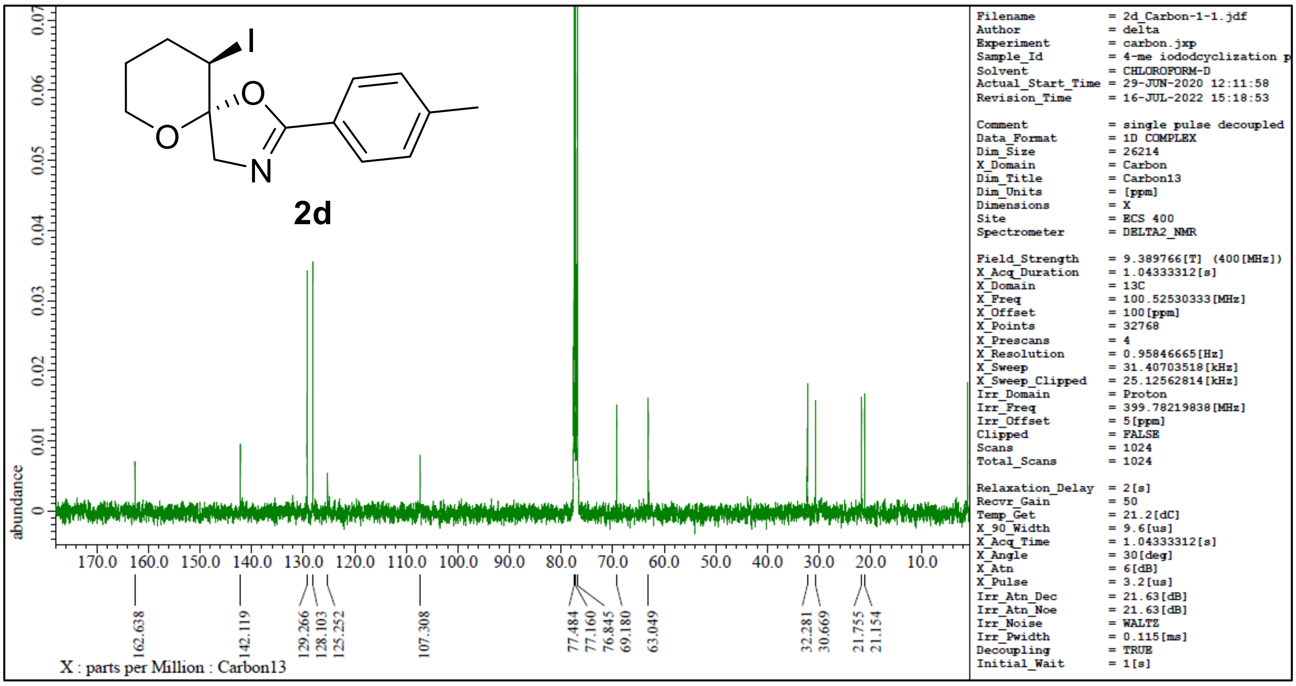

Compound **2d** (^{13}C NMR, 100 MHz, $CDCl_3$).

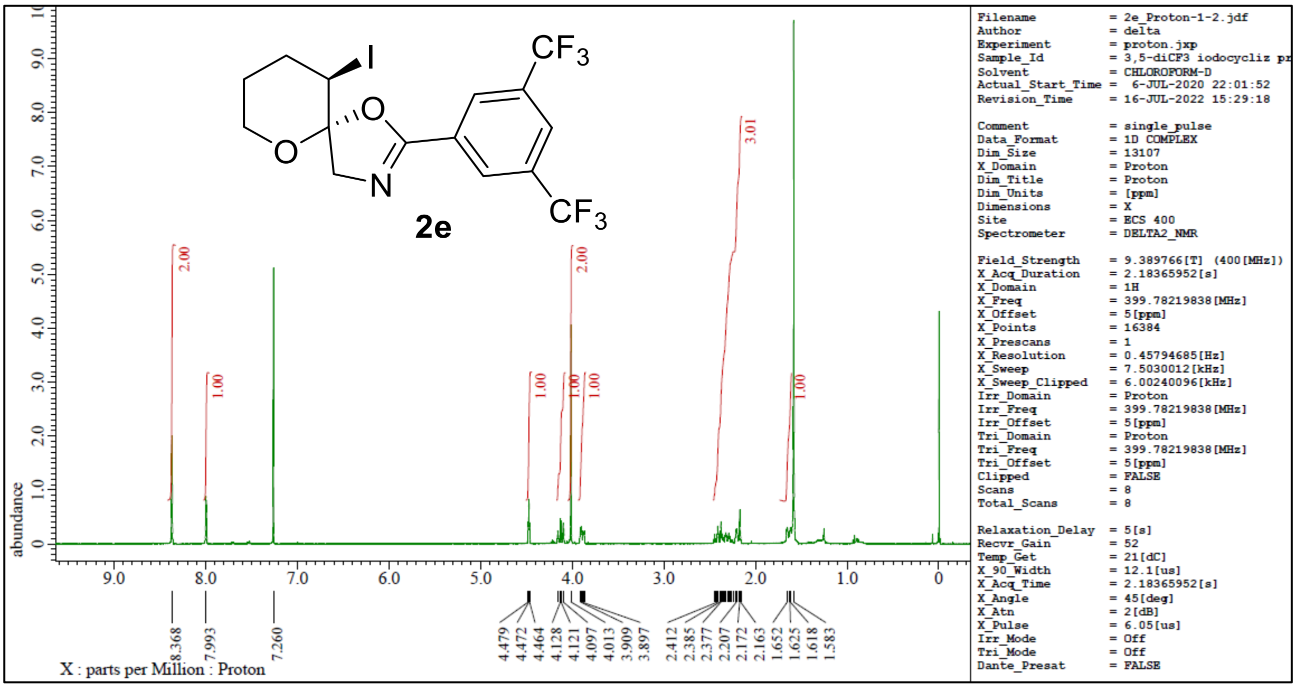

Compound **2e** (^{1}H NMR, 400 MHz, $CDCl_3$).

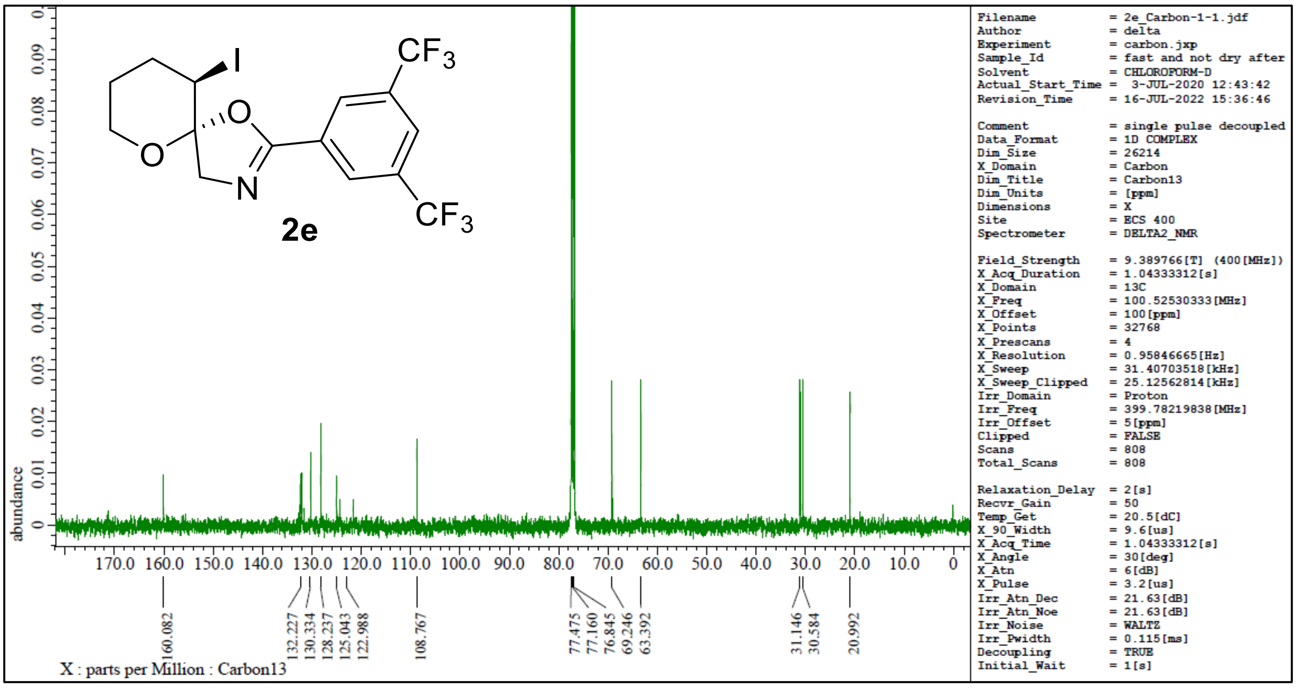

Compound **2e** (^{13}C NMR, 100 MHz, $CDCl_3$).

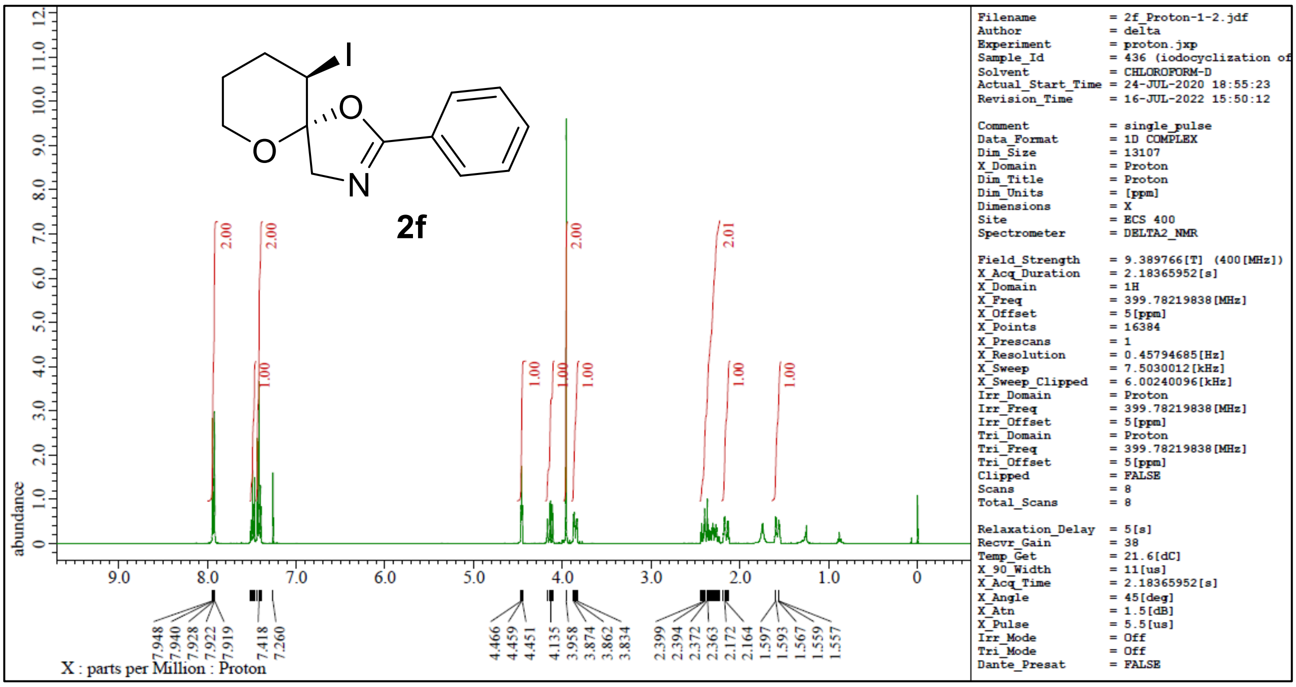

Compound **2f** (^{1}H NMR, 400 MHz, $CDCl_3$).

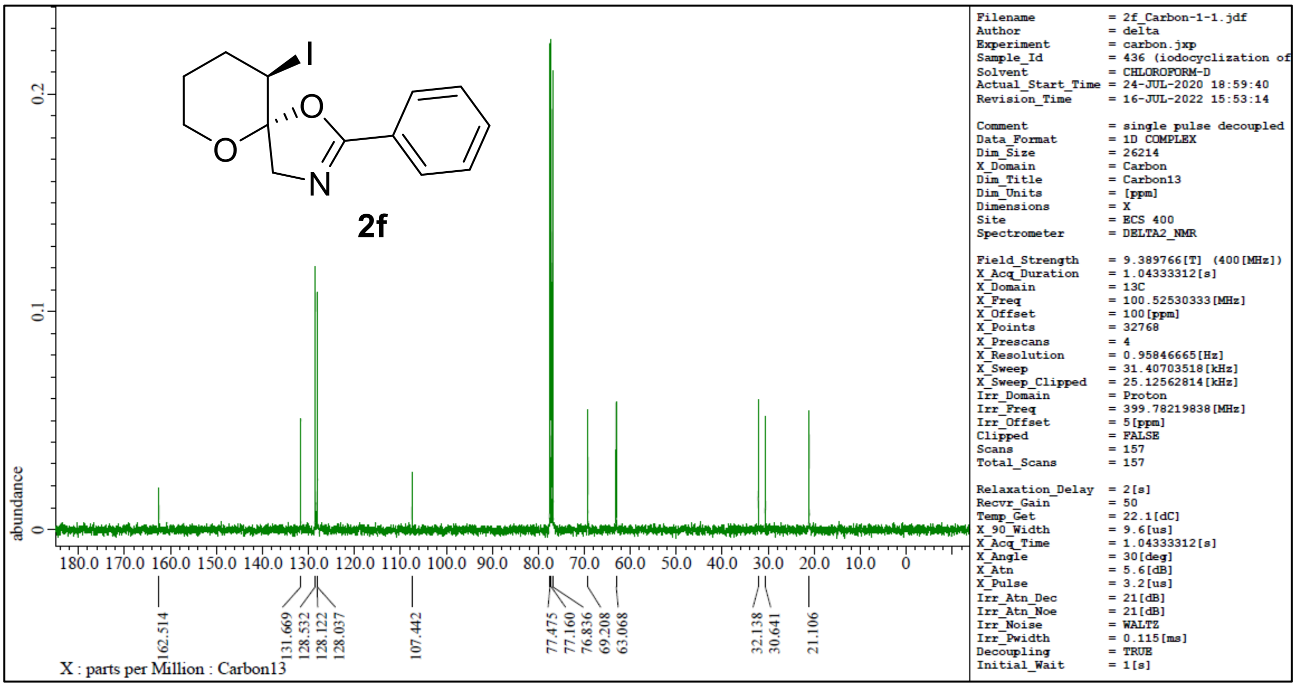

Compound **2f** (^{13}C NMR, 100 MHz, $CDCl_3$).

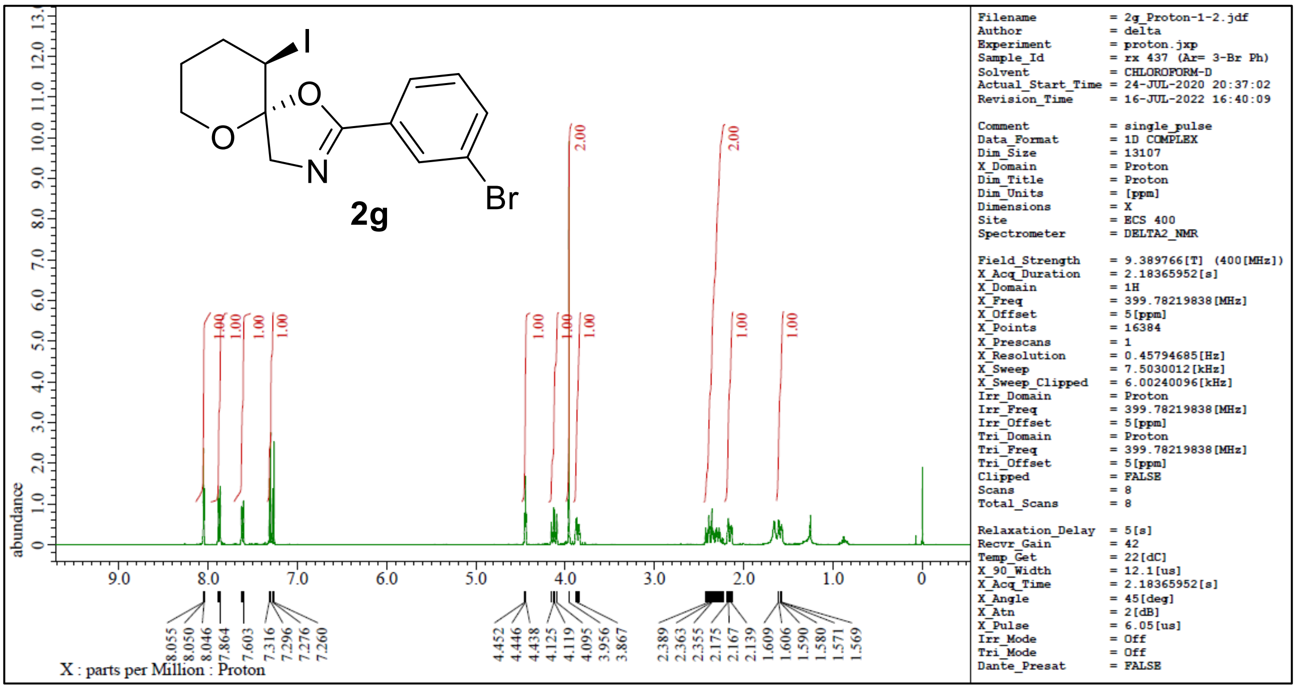

Compound **2g** (^{1}H NMR, 400 MHz, $CDCl_3$).

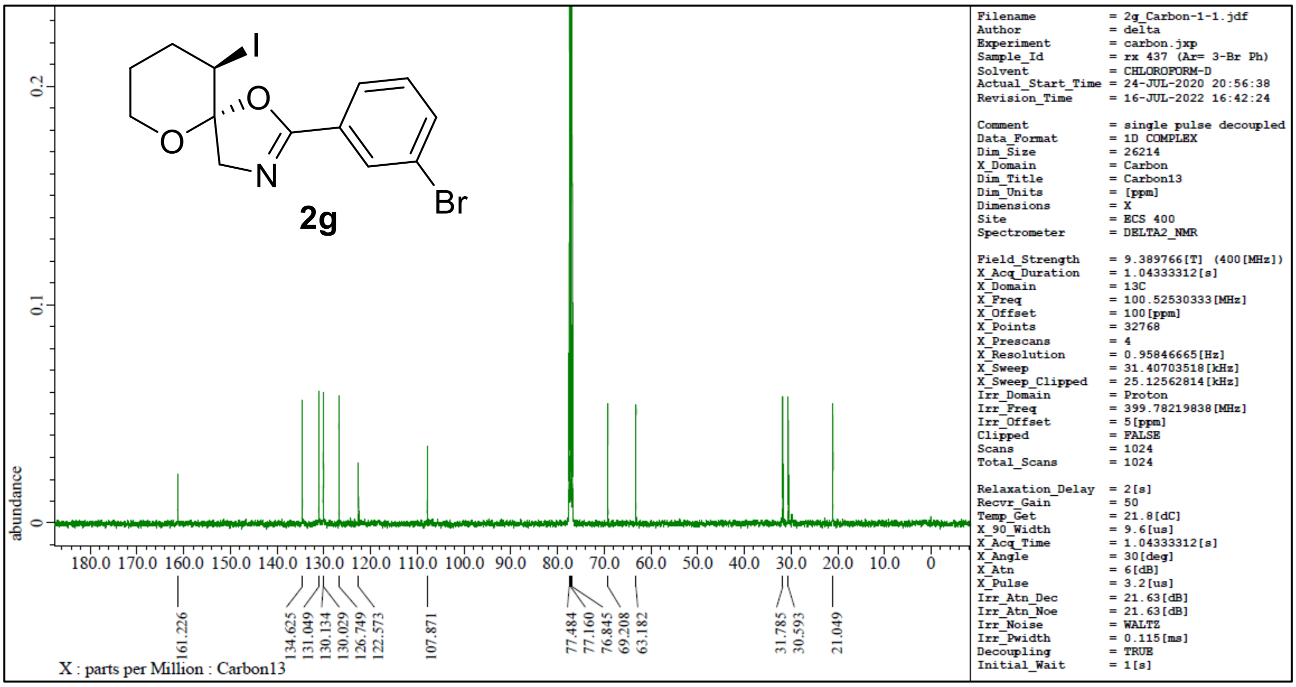

Compound **2g** (^{13}C NMR, 100 MHz, $CDCl_3$).

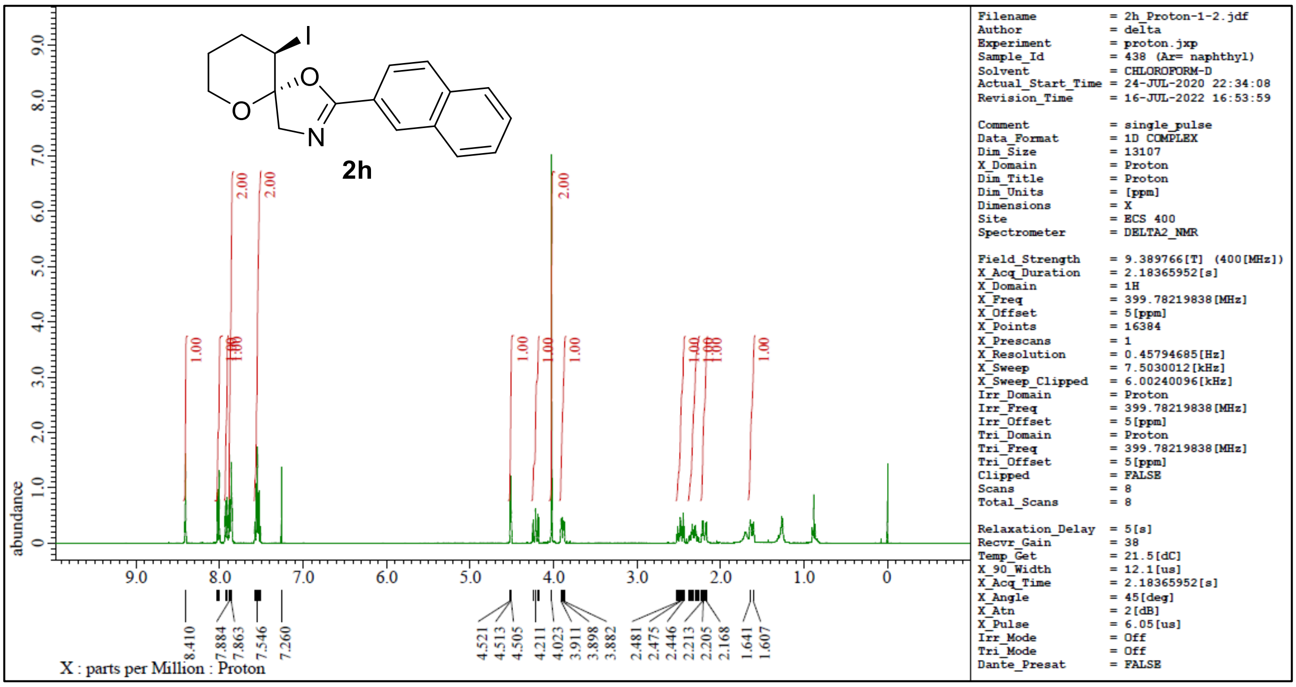

Compound **2h** (^{1}H NMR, 400 MHz, $CDCl_3$).

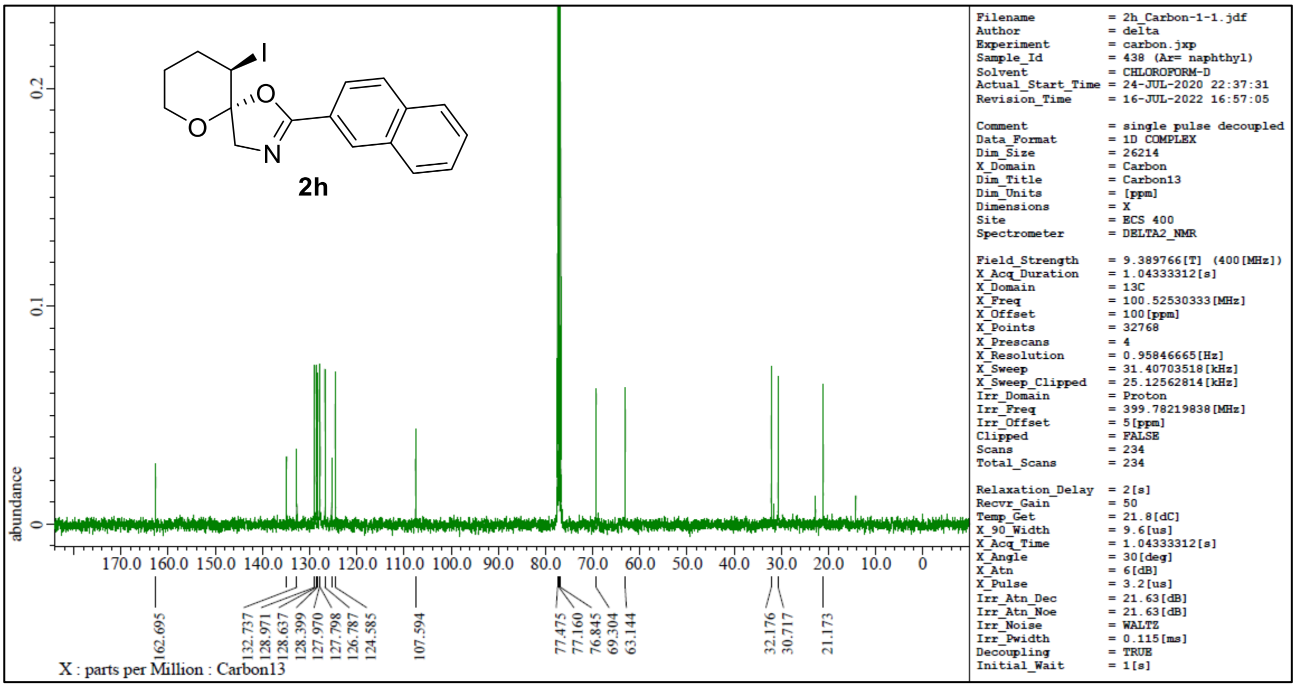

Compound **2h** (^{13}C NMR, 100 MHz, $CDCl_3$).

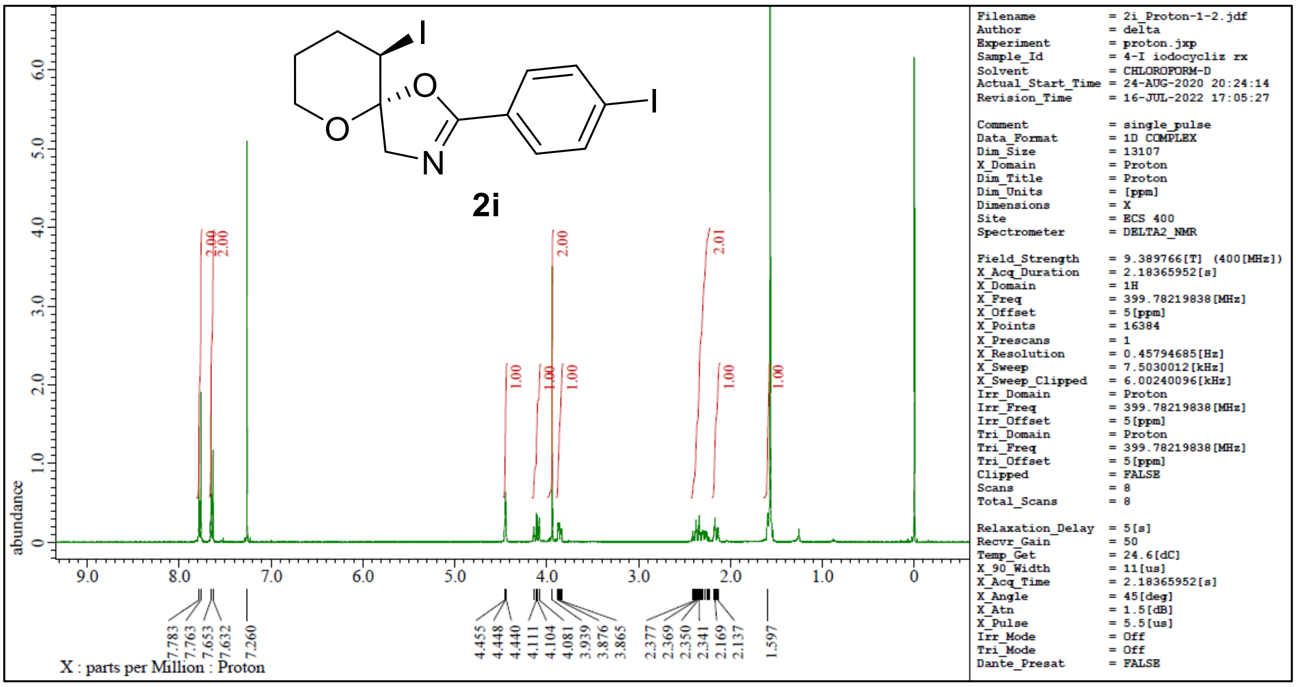

Compound **2i** (^{1}H NMR, 400 MHz, $CDCl_3$).

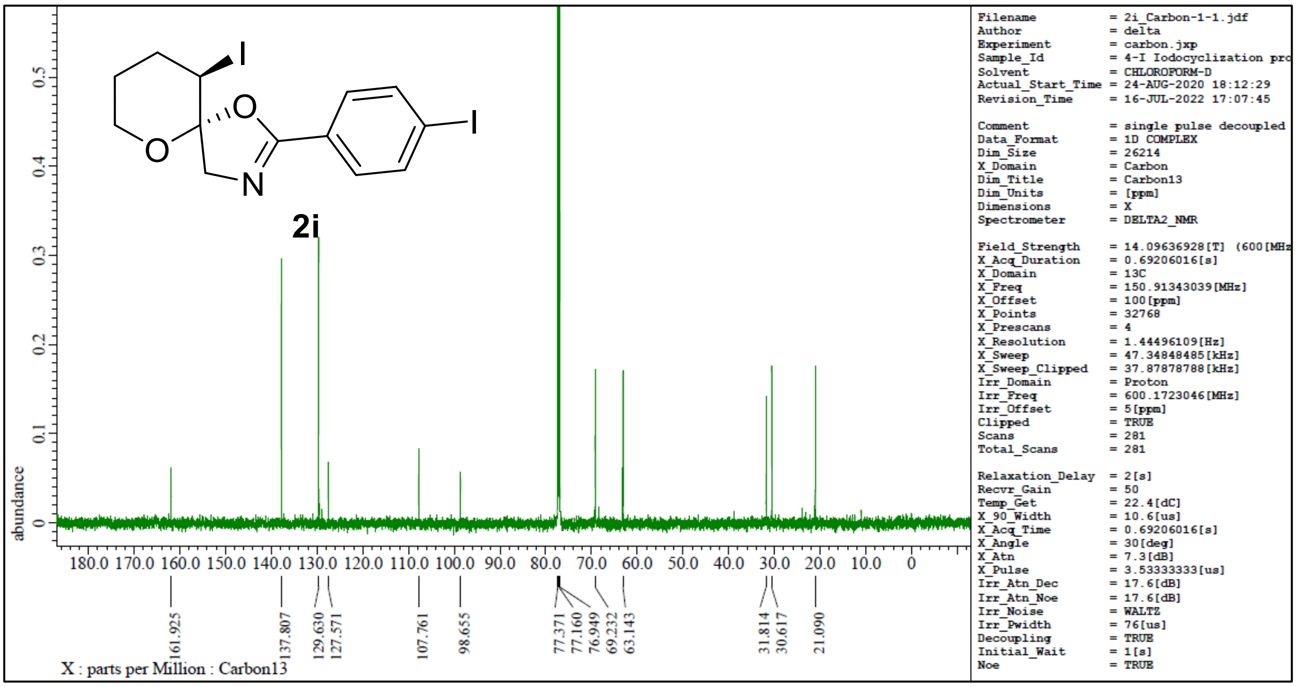

Compound **2i** (^{13}C NMR, 150 MHz, $CDCl_3$).

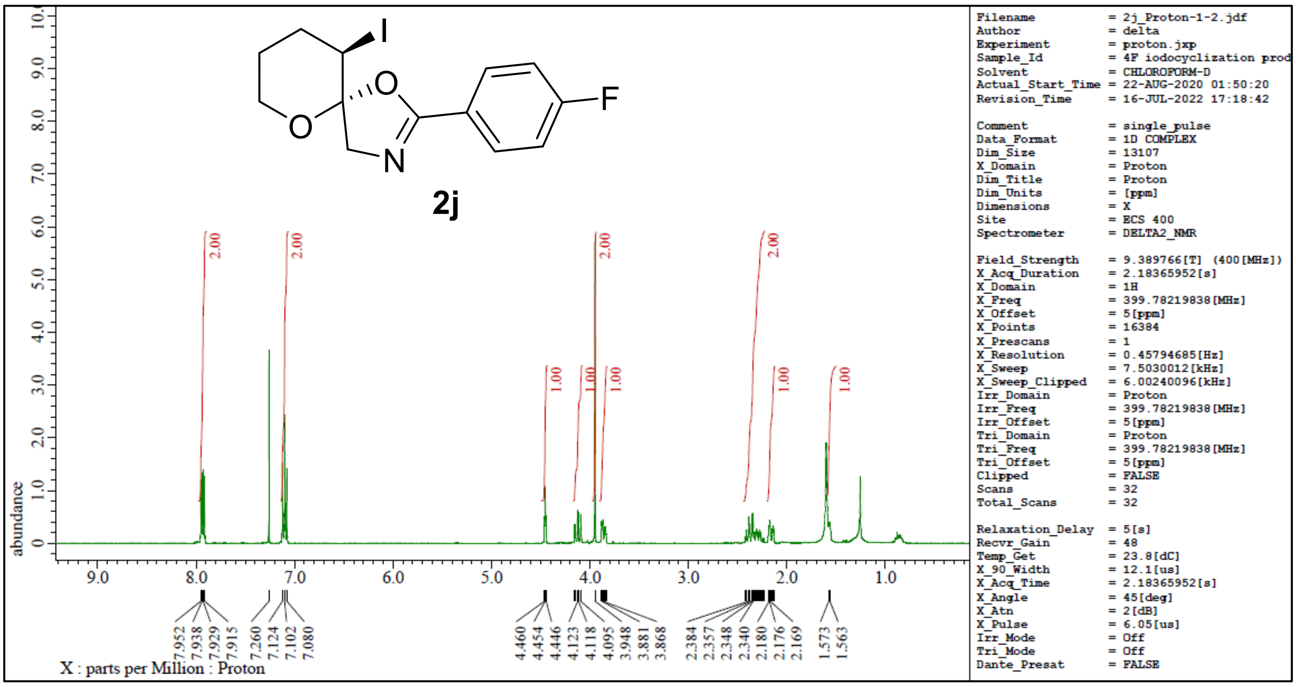

Compound **2j** (^{1}H NMR, 400 MHz, $CDCl_3$).

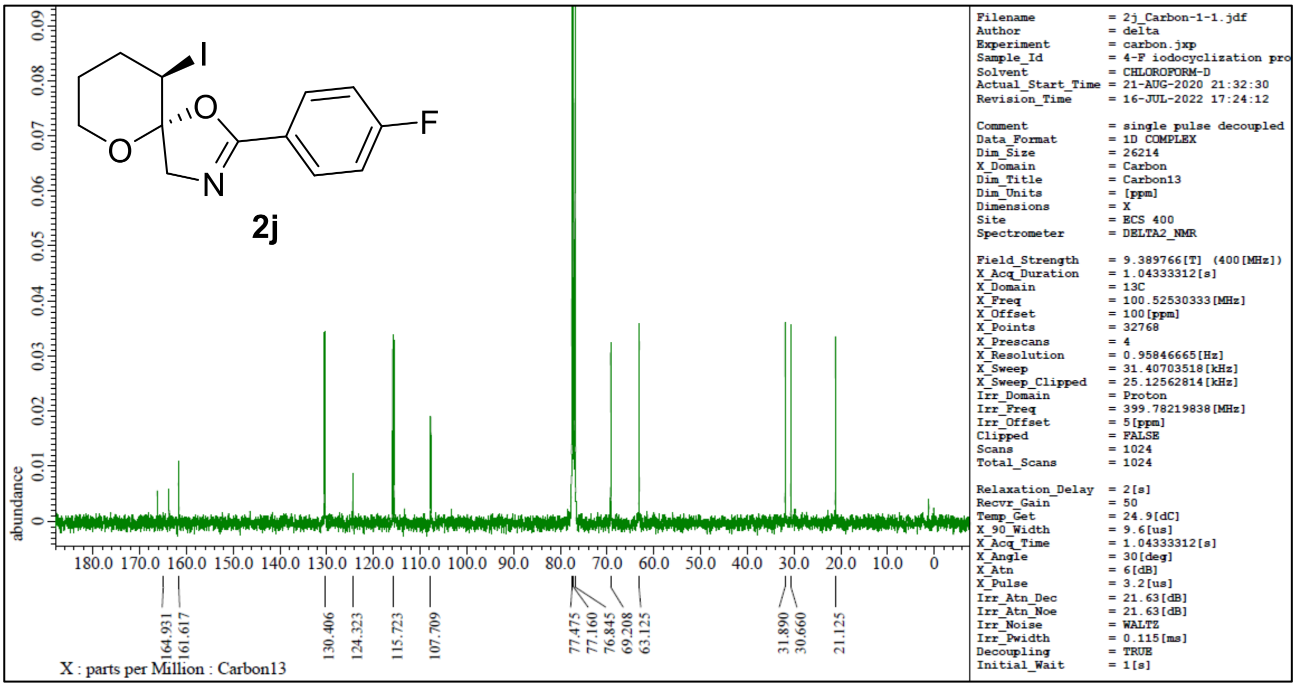

Compound **2j** (^{13}C NMR, 100 MHz, $CDCl_3$).

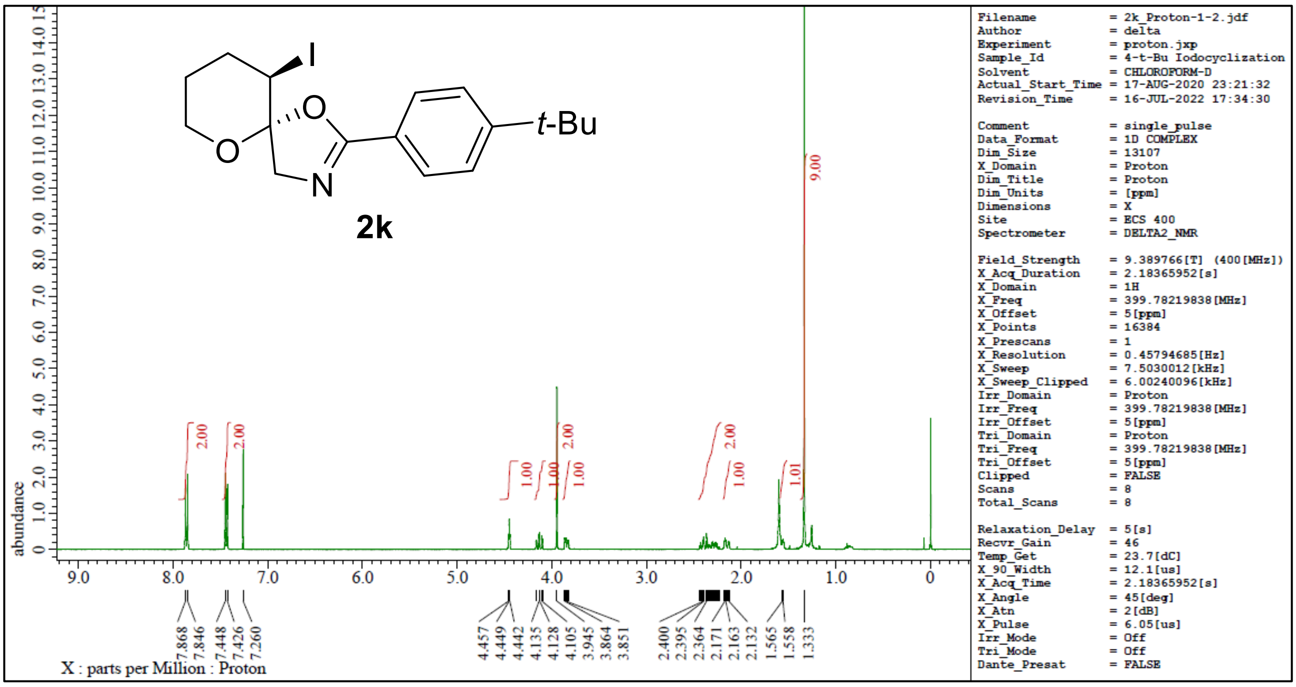

Compound **2k** (^{1}H NMR, 400 MHz, $CDCl_3$).

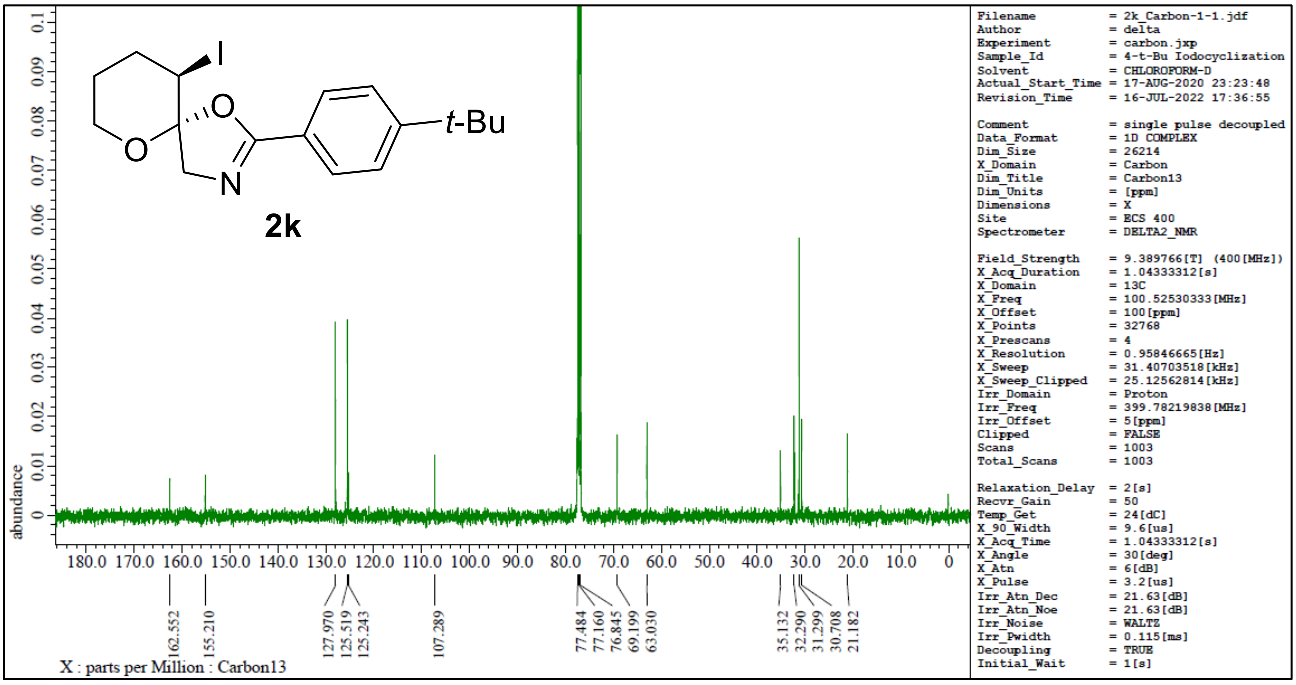

Compound **2k** (^{13}C NMR, 100 MHz, $CDCl_3$).

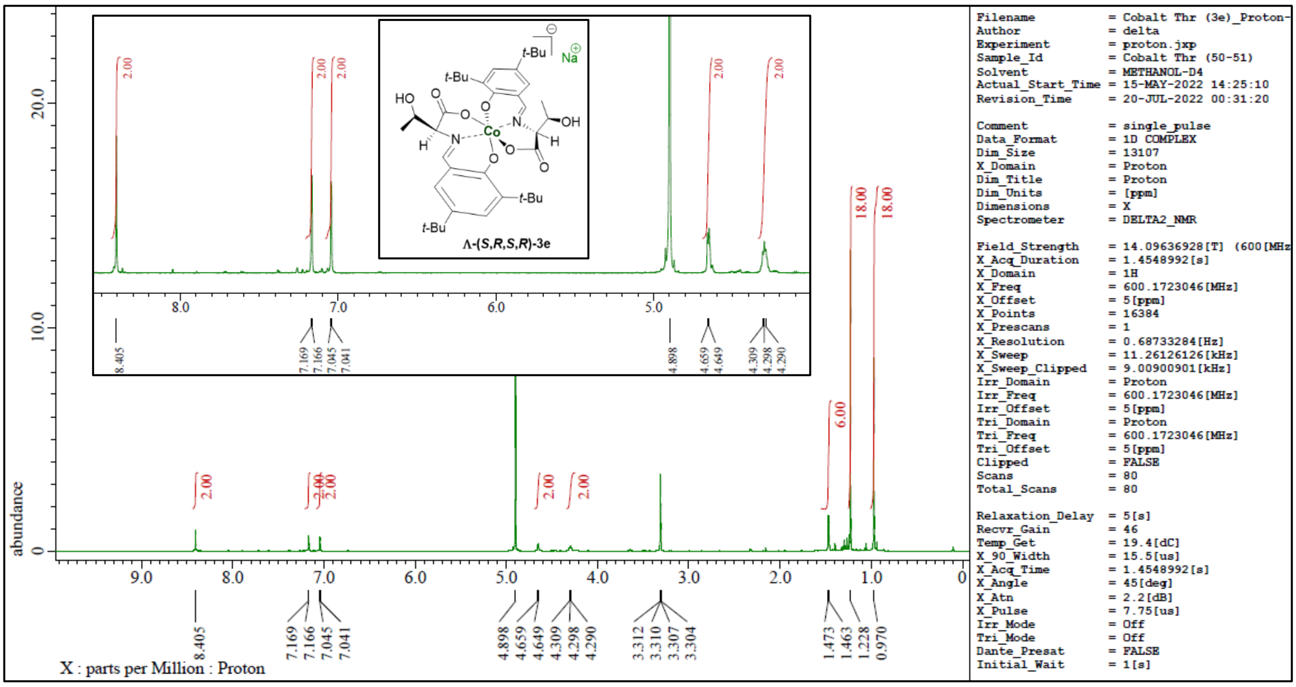

Complex Λ-(*S*,*R*,*S*,*R*)-**3e** (^{1}H NMR, 600 MHz, CD_3OD).

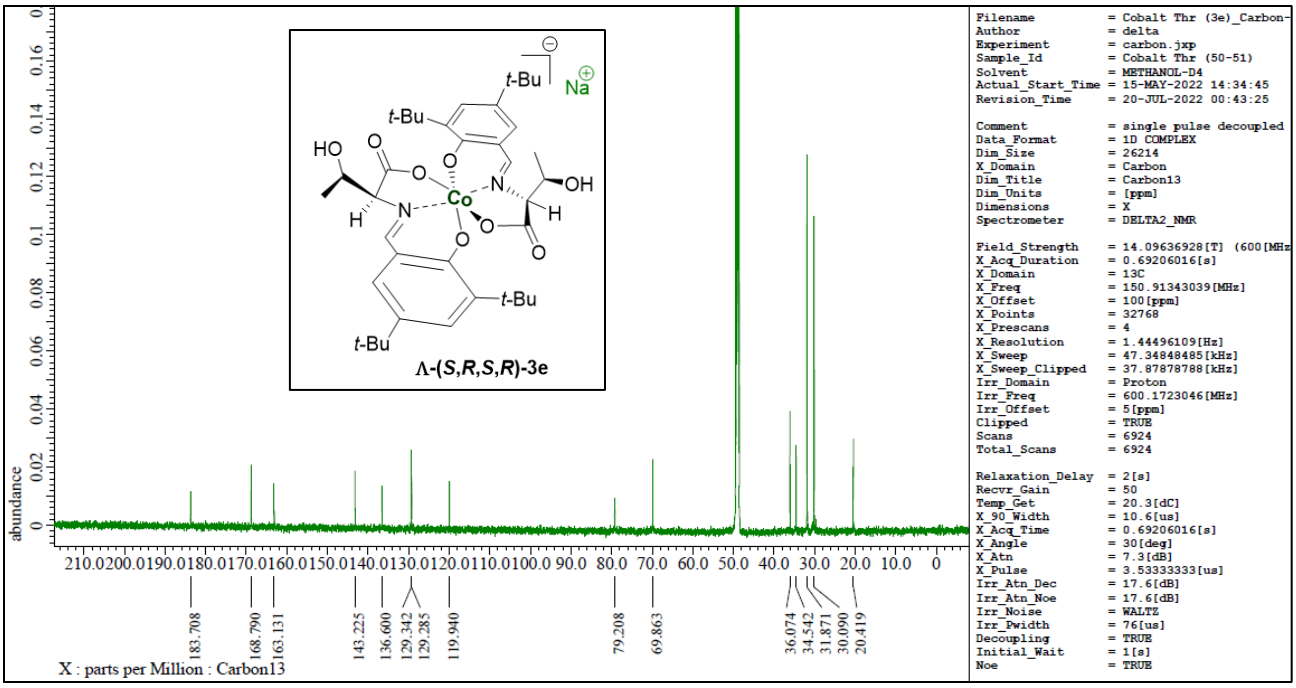

Complex Λ-(*S*,*R*,*S*,*R*)-**3e** (^{13}C NMR, 150 MHz, CD_3OD).

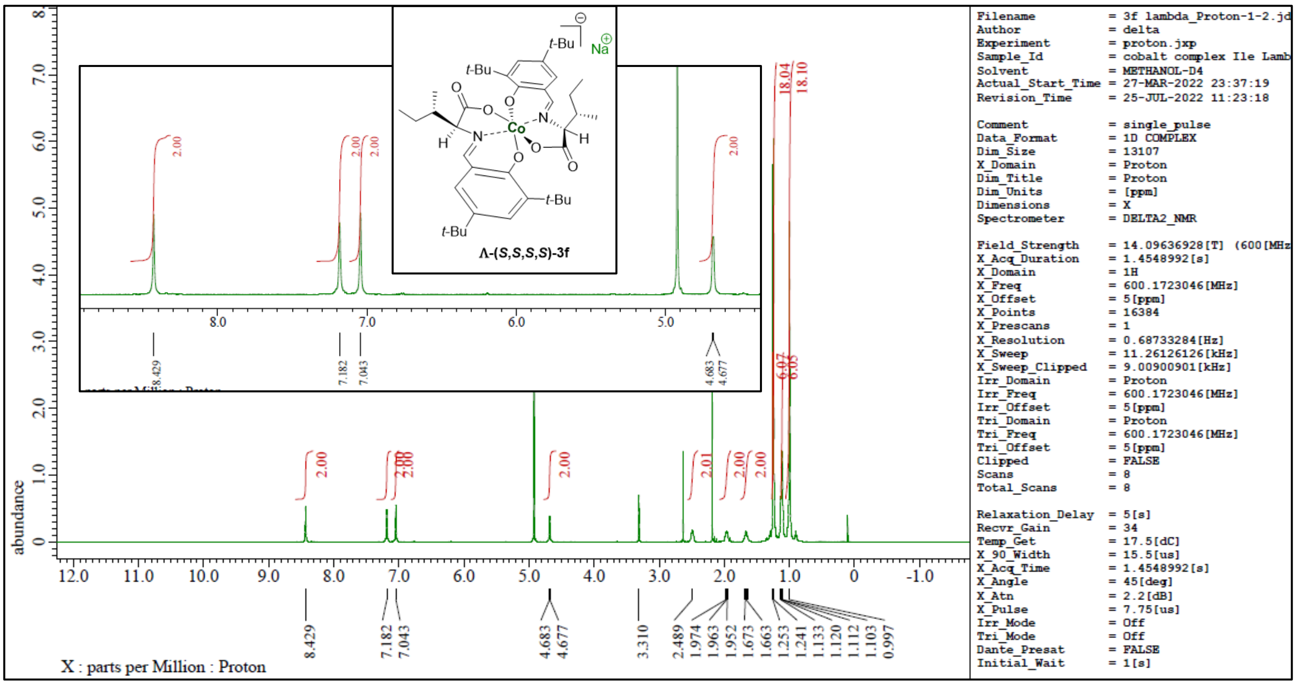

Complex Λ-(*S*,*S*,*S*,*S*)-**3f** (^{1}H NMR, 600 MHz, CD_3OD).

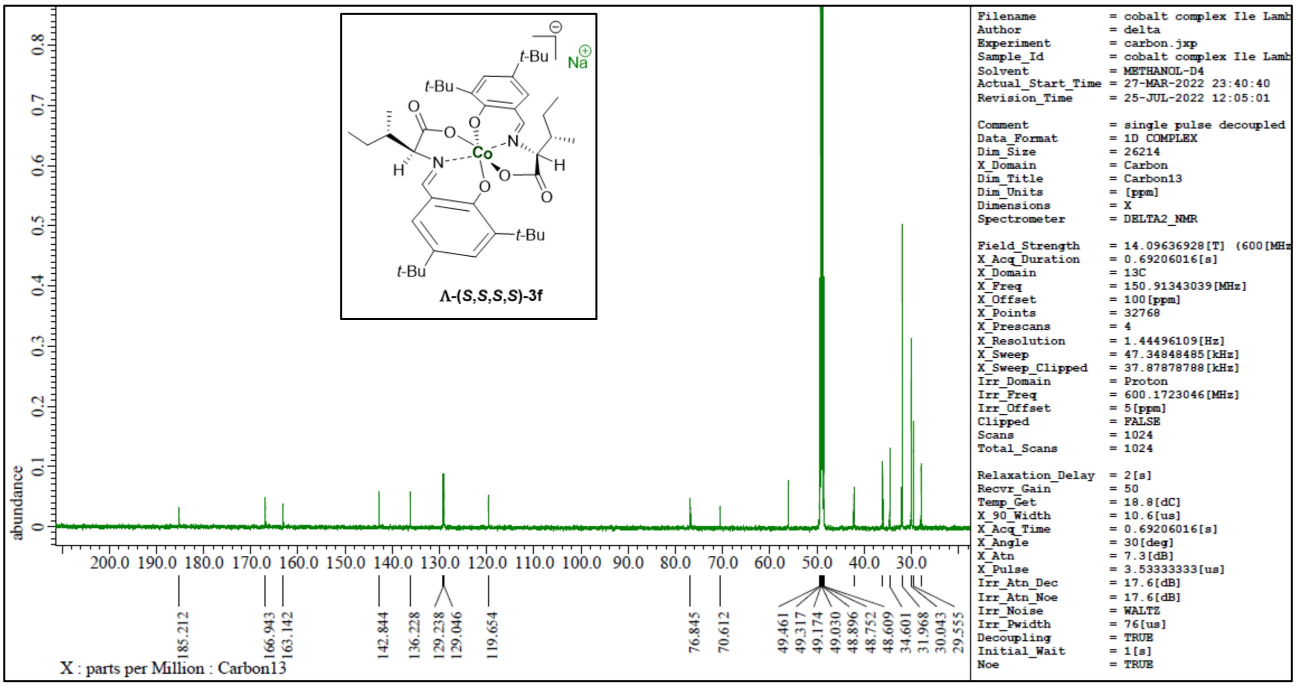

Complex Λ-(*S*,*S*,*S*,*S*)-**3f** (^{13}C NMR, 150 MHz, CD_3OD).

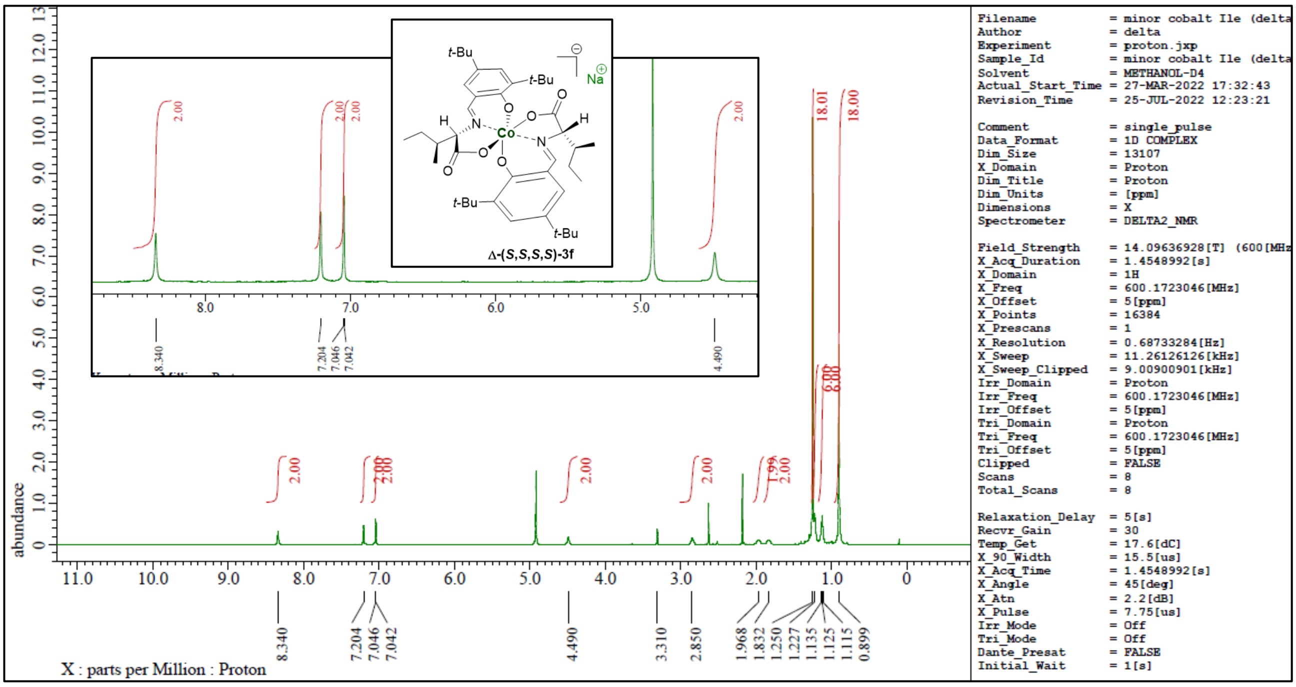

Complex Δ-(*S*,*S*,*S*,*S*)-**3f** (^{1}H NMR, 600 MHz, CD_3OD).

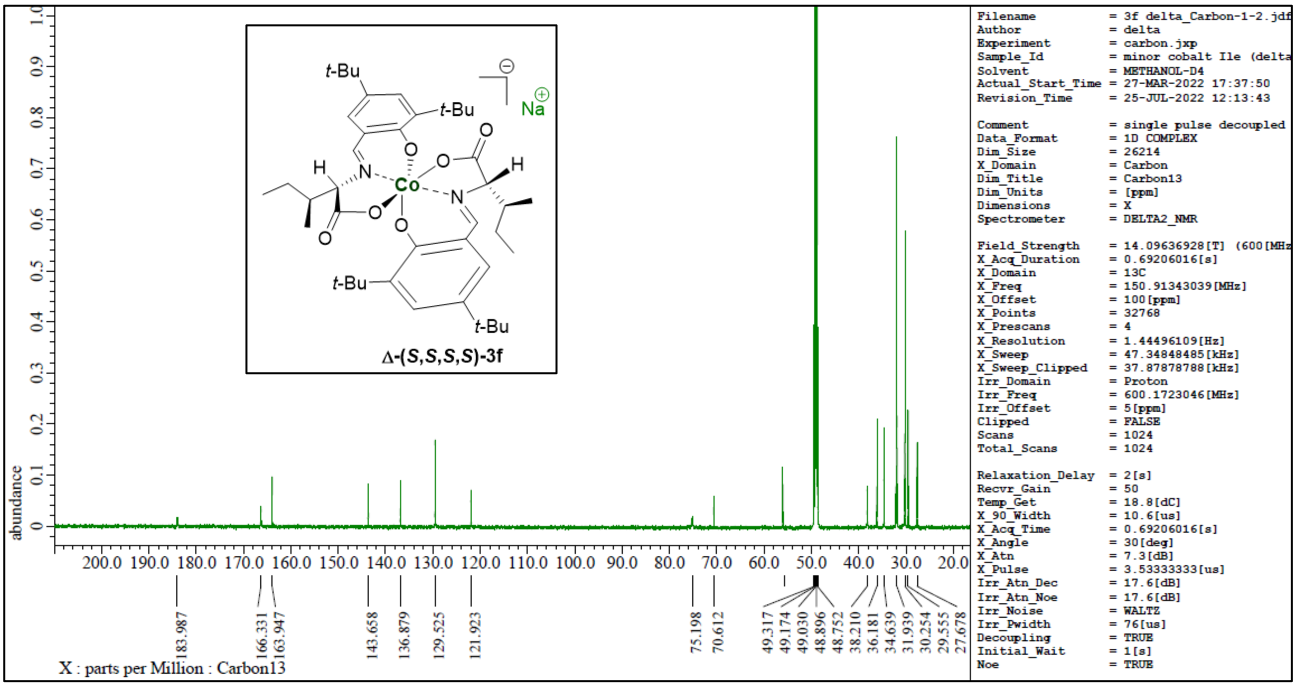

Complex Δ-(*S*,*S*,*S*,*S*)-**3f** (^{13}C NMR, 150 MHz, CD_3OD).

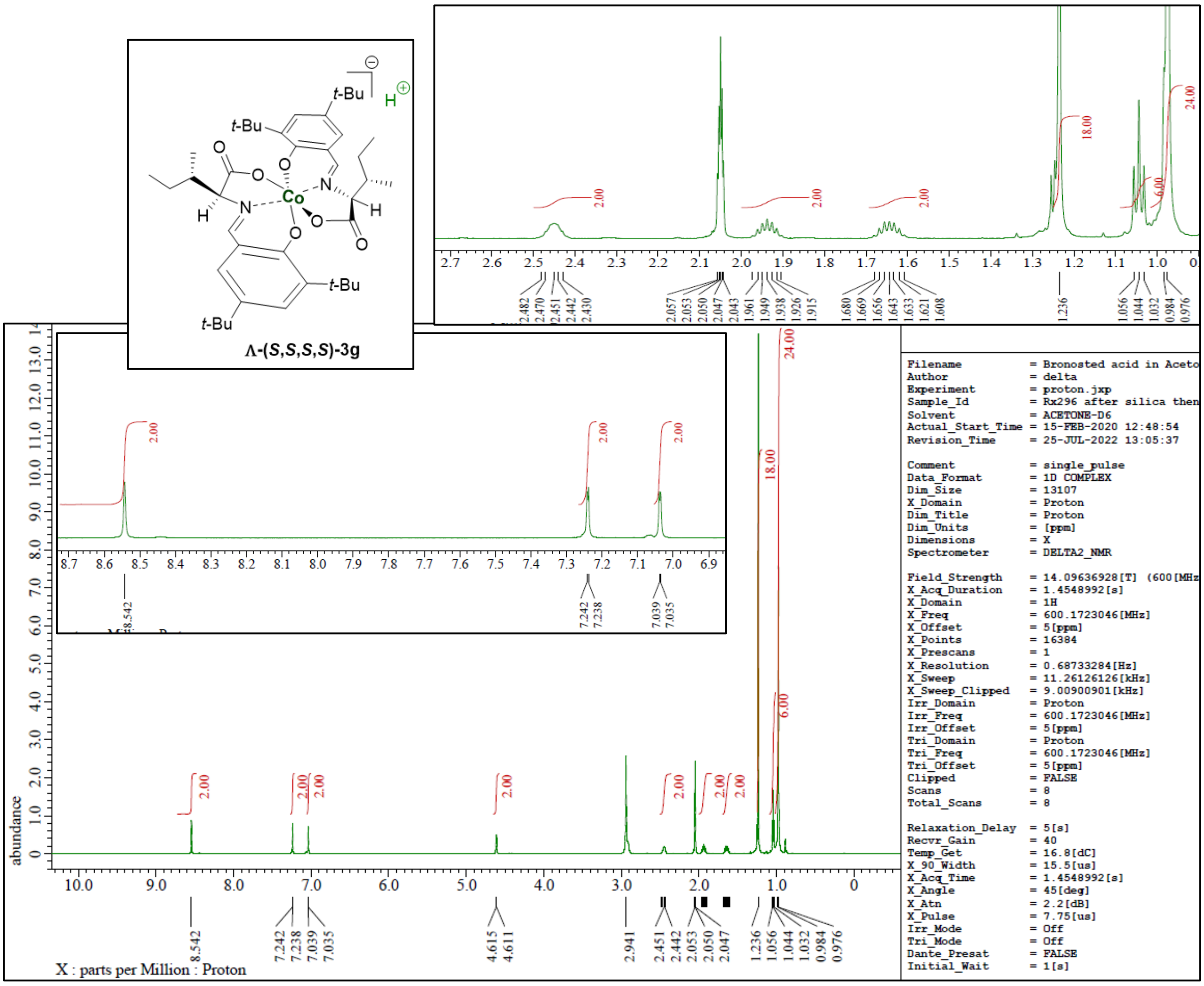

Complex Λ-(*S*,*S*,*S*,*S*)-**3g** (^{1}H NMR, 150 MHz, $(CD_3)_2CO$).

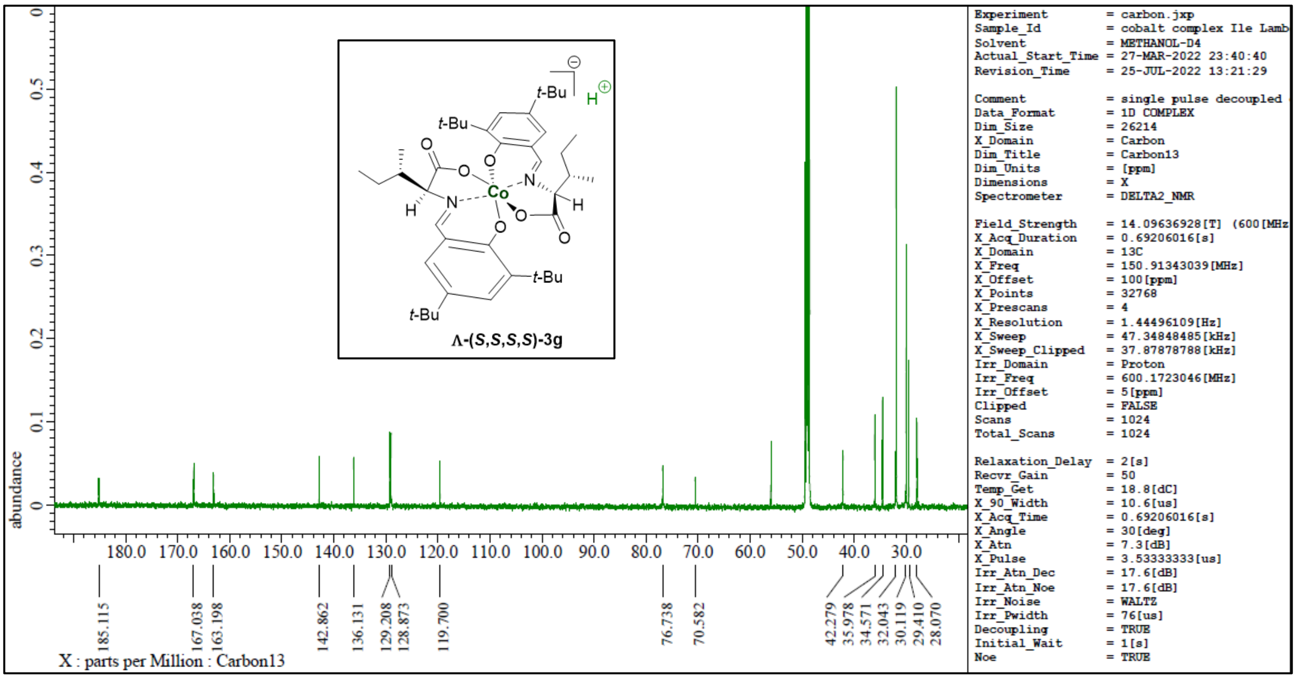

Complex Λ-(*S*,*S*,*S*,*S*)-**3g** (^{13}C NMR, 150 MHz, CD_3OD).

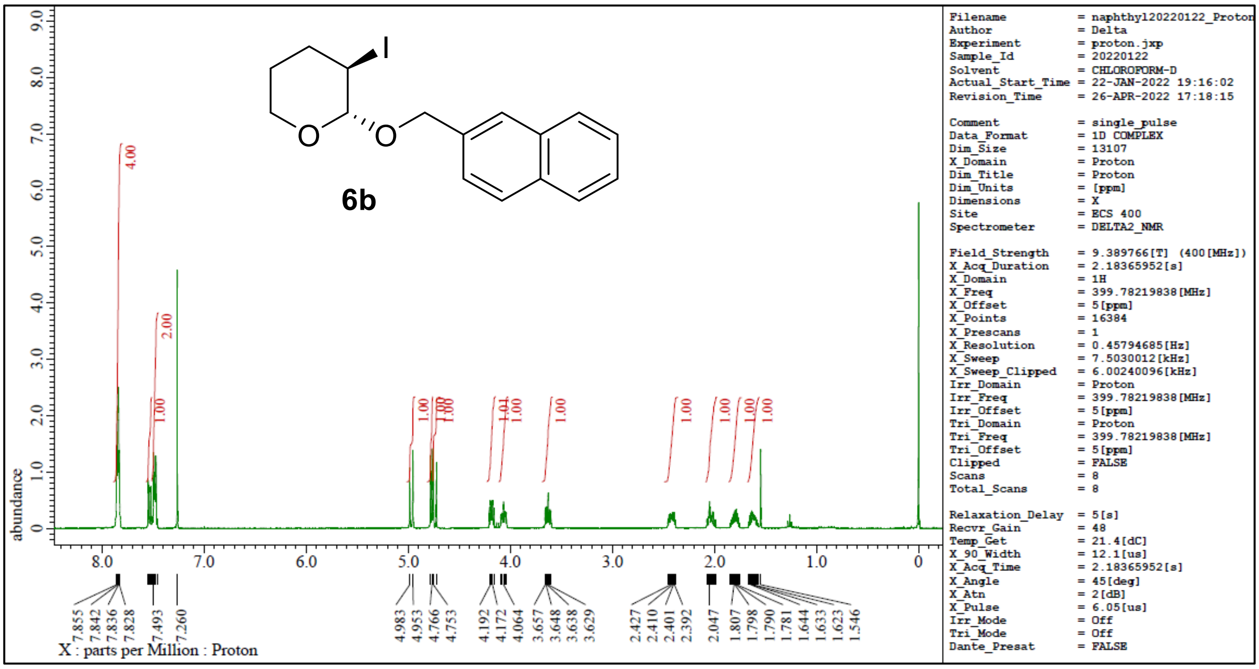

Compound **6b** (^{1}H NMR, 400 MHz, $CDCl_3$).

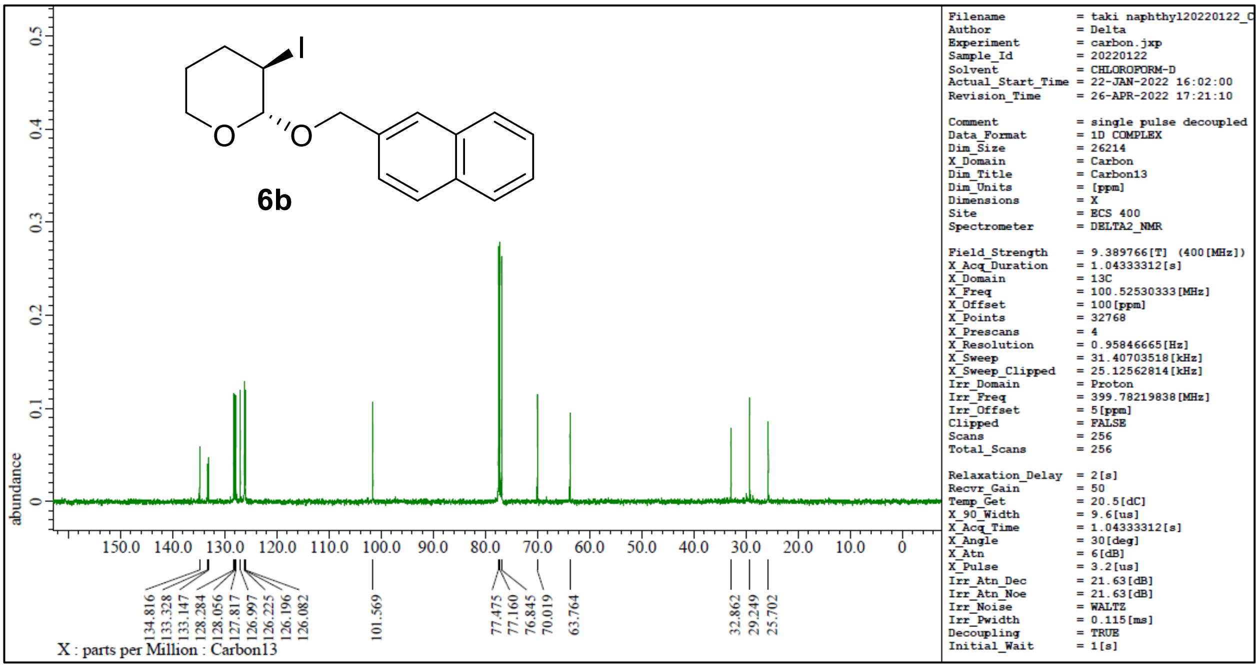

Compound **6b** (^{13}C NMR, 100 MHz, $CDCl_3$).

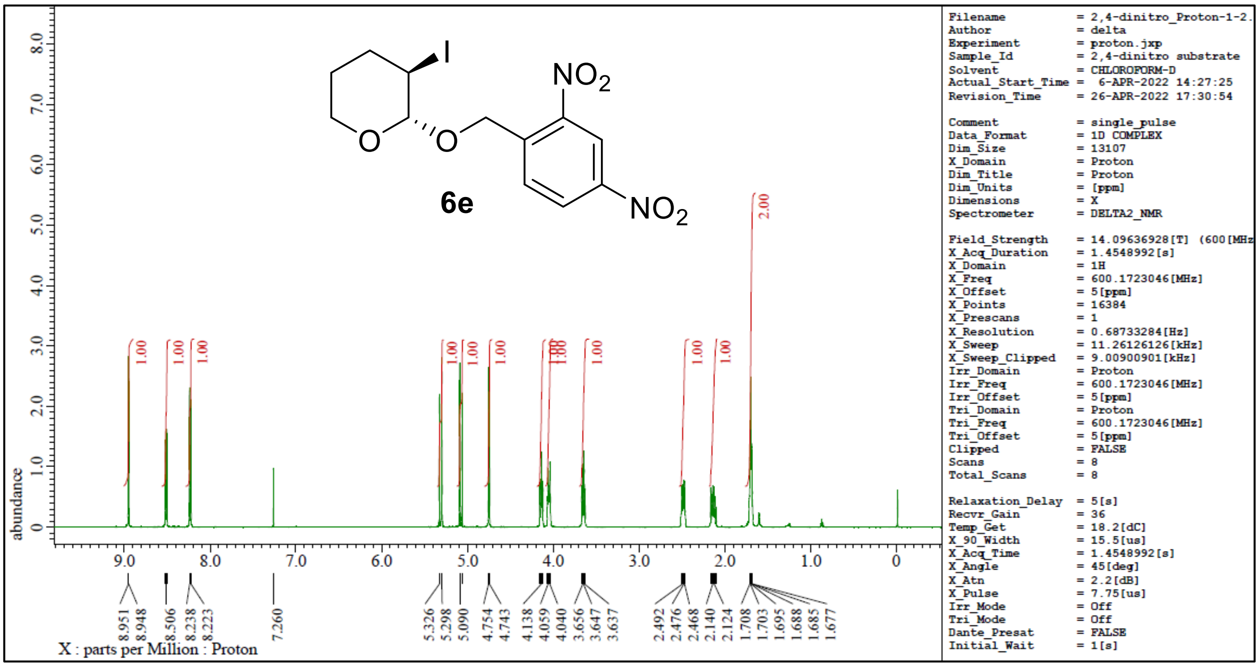

Compound **6e** (^{1}H NMR, 600 MHz, $CDCl_3$).

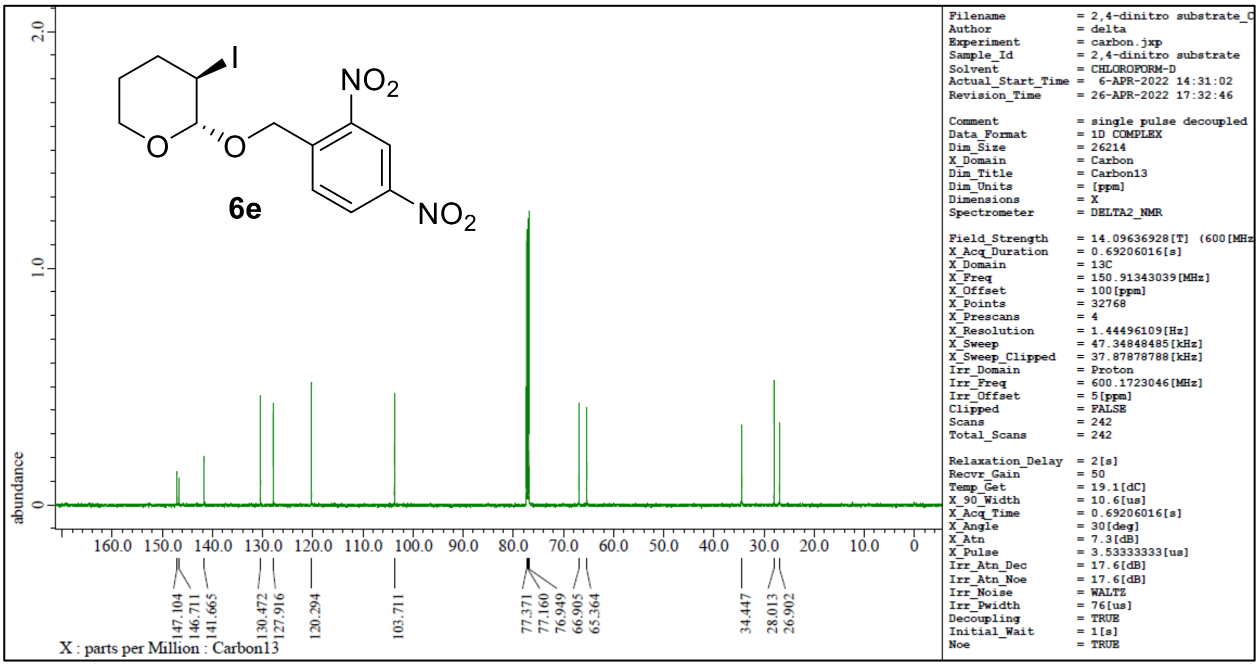

Compound **6e** (^{13}C NMR, 150 MHz, $CDCl_3$).

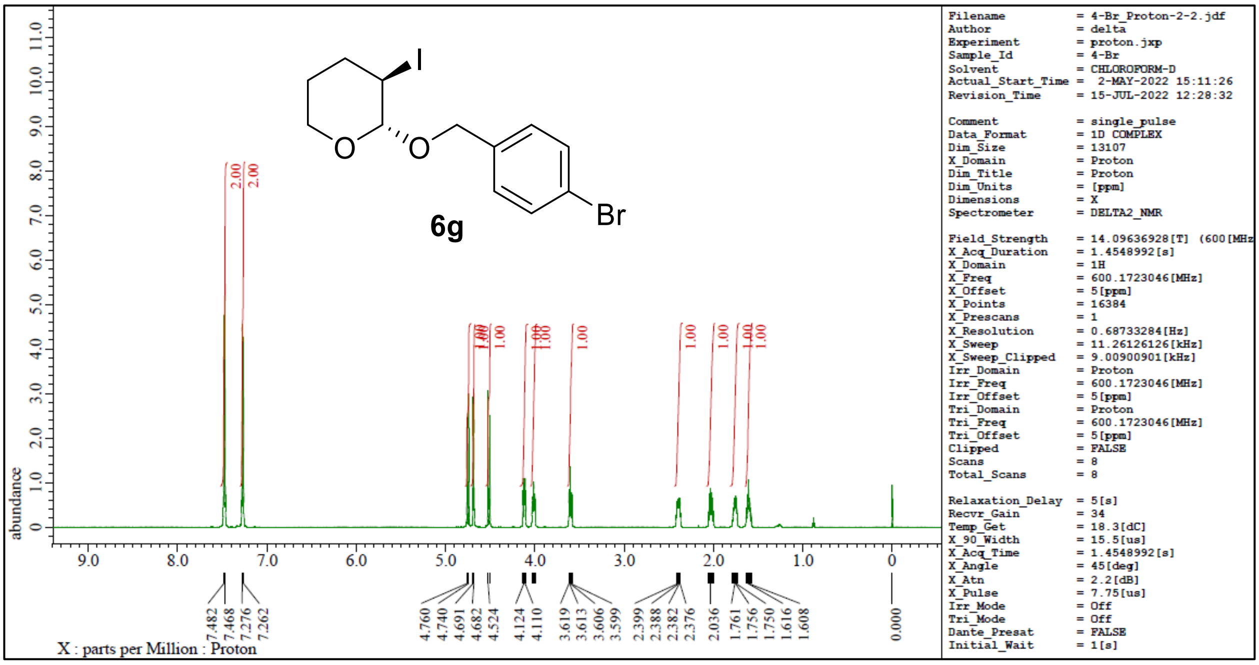

Compound **6g** (^{1}H NMR, 600 MHz, $CDCl_3$).

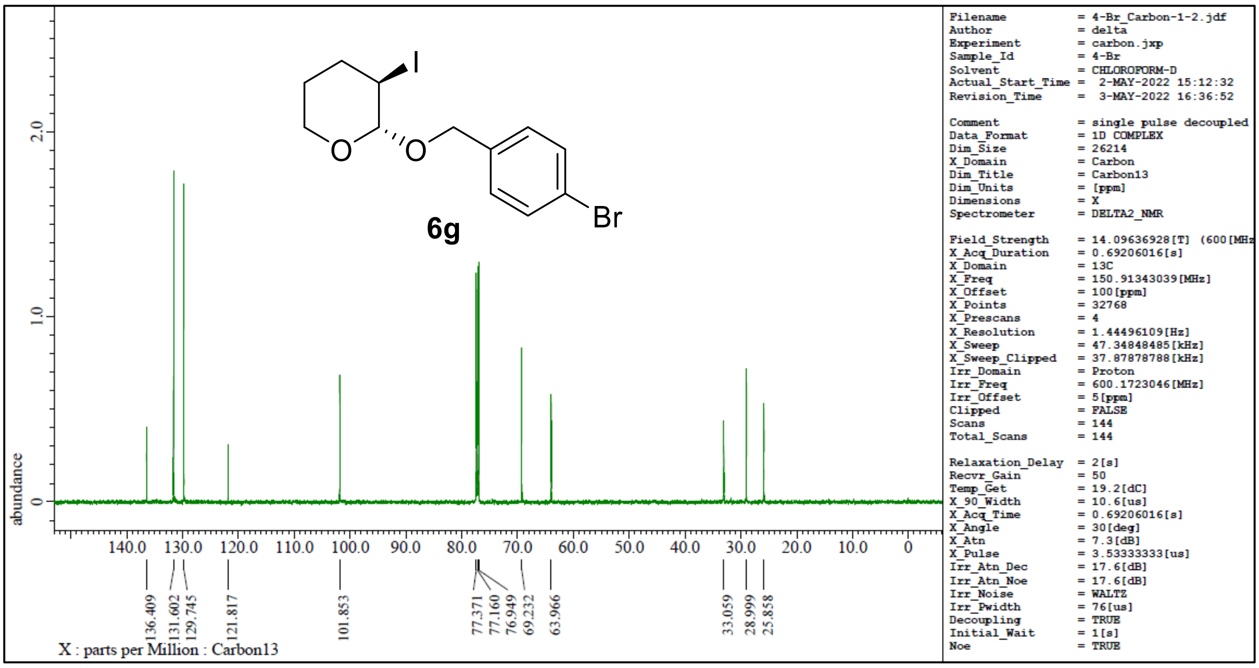

Compound **6g** (^{13}C NMR, 150 MHz, $CDCl_3$).

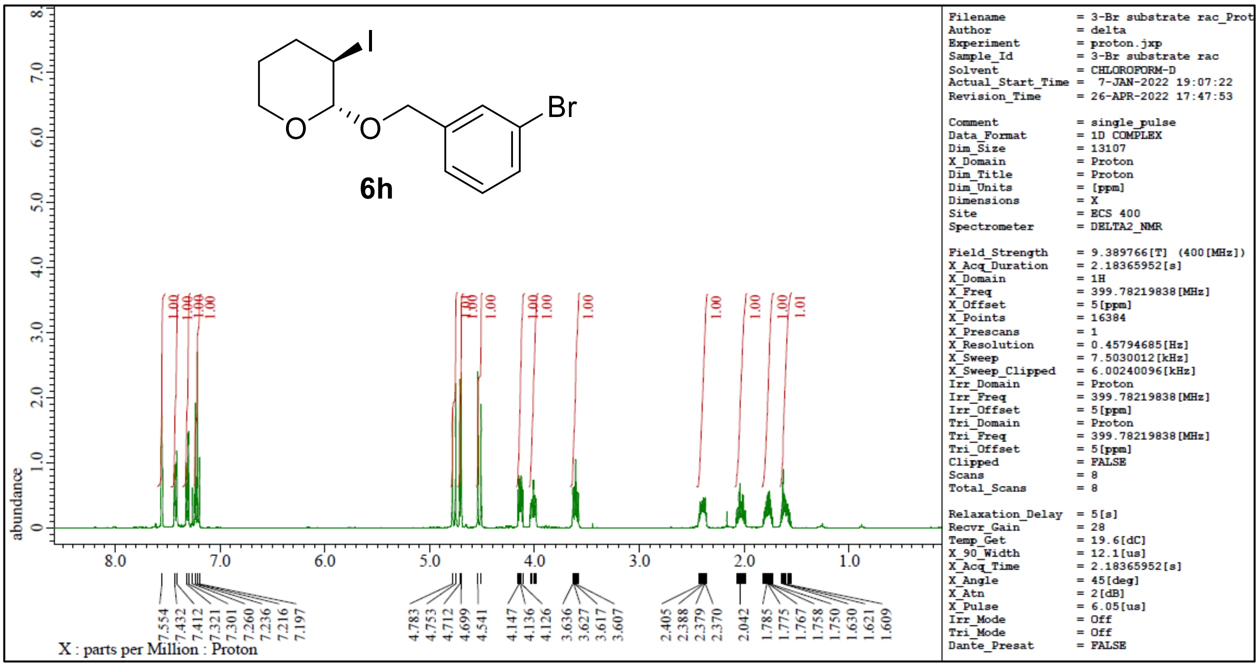

Compound **6h** (^{1}H NMR, 400 MHz, $CDCl_3$).

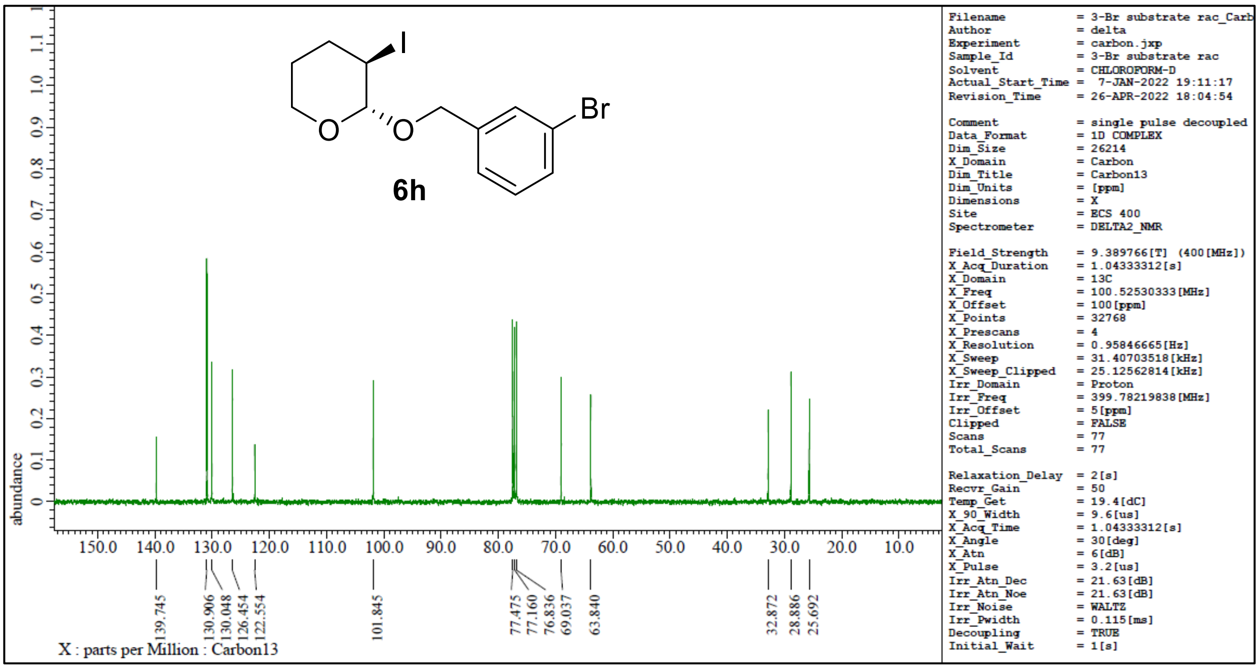

Compound **6h** (^{13}C NMR, 100 MHz, $CDCl_3$).

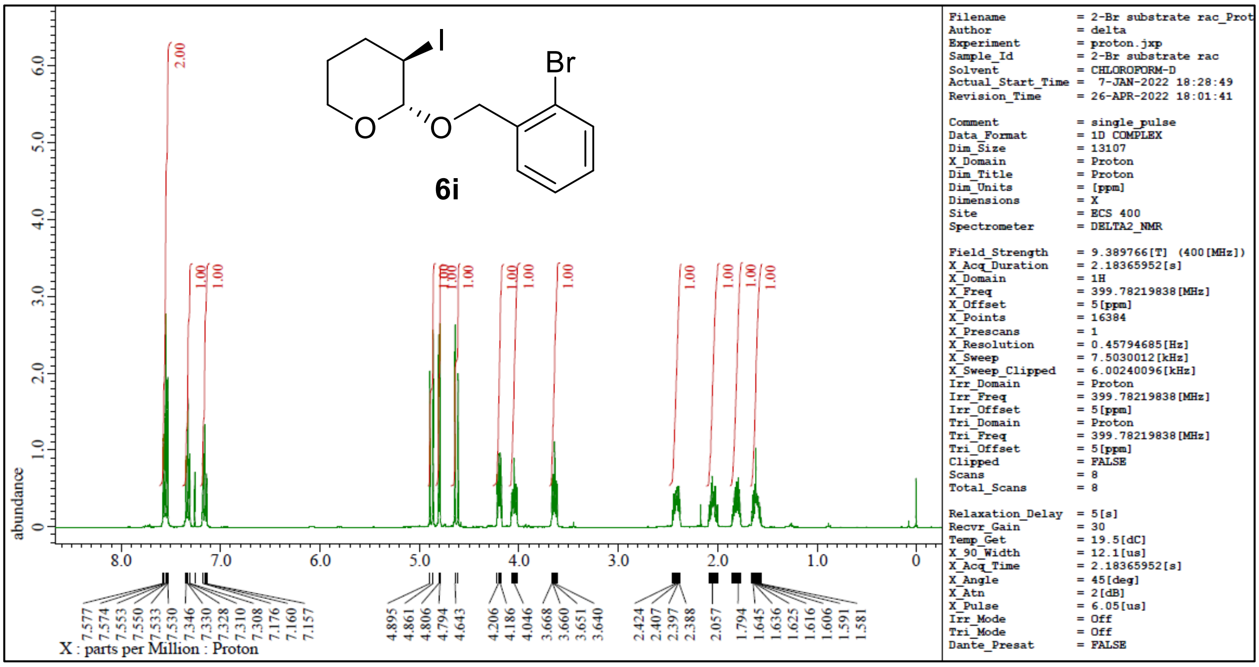

Compound **6i** (^{1}H NMR, 400 MHz, $CDCl_3$).

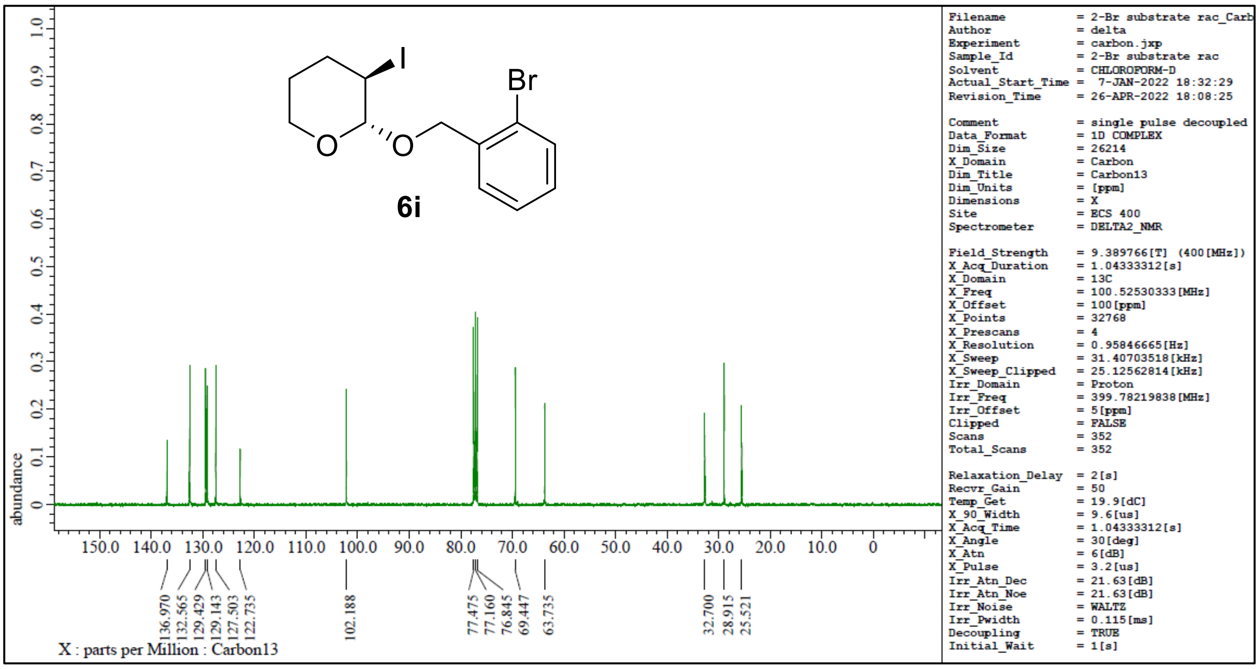

Compound **6i** (^{13}C NMR, 100 MHz, $CDCl_3$).

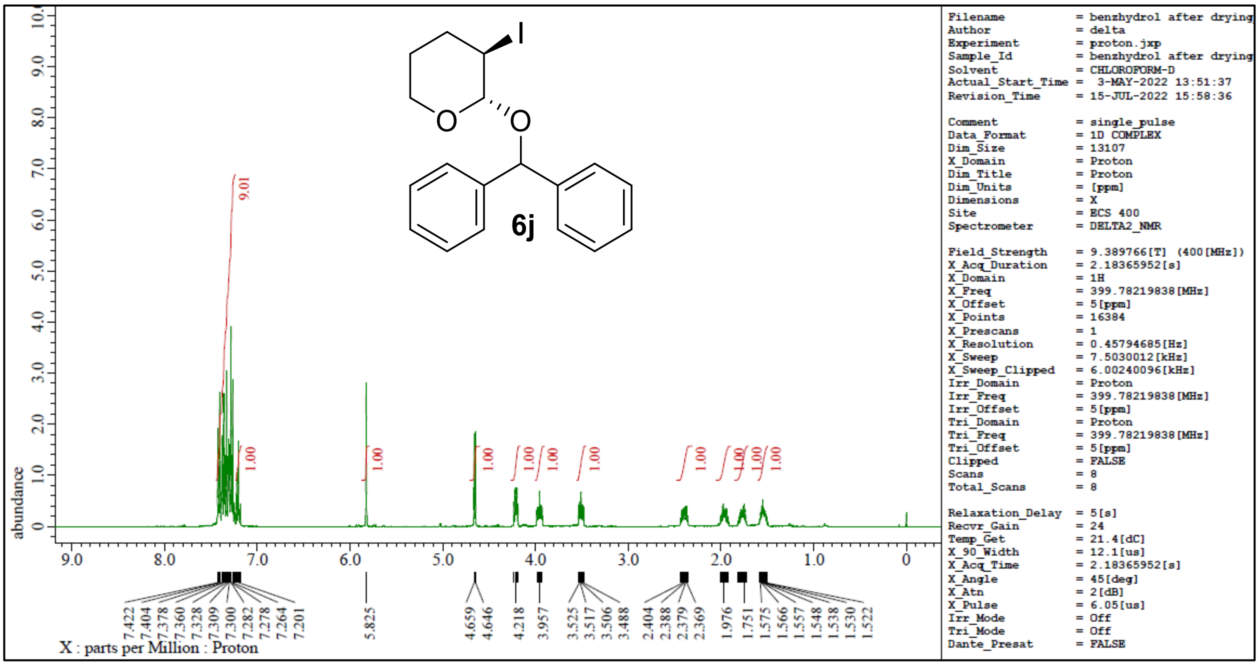

Compound **6j** (^{1}H NMR, 400 MHz, $CDCl_3$).

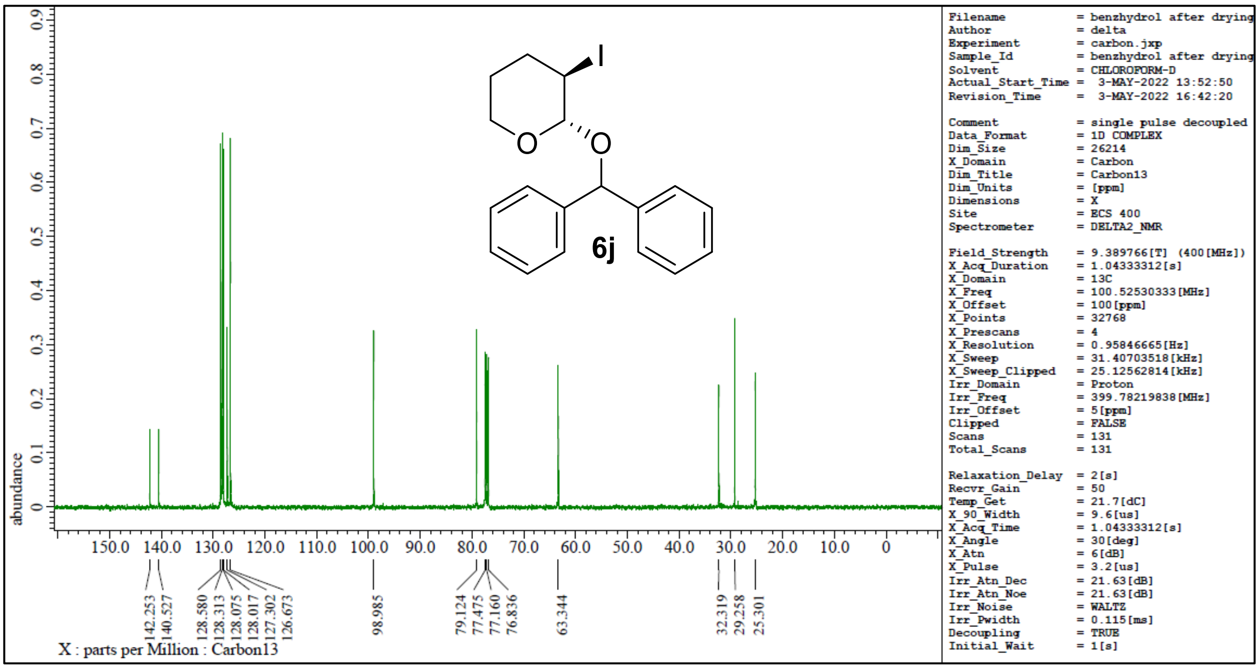

Compound **6j** (^{13}C NMR, 100 MHz, $CDCl_3$).

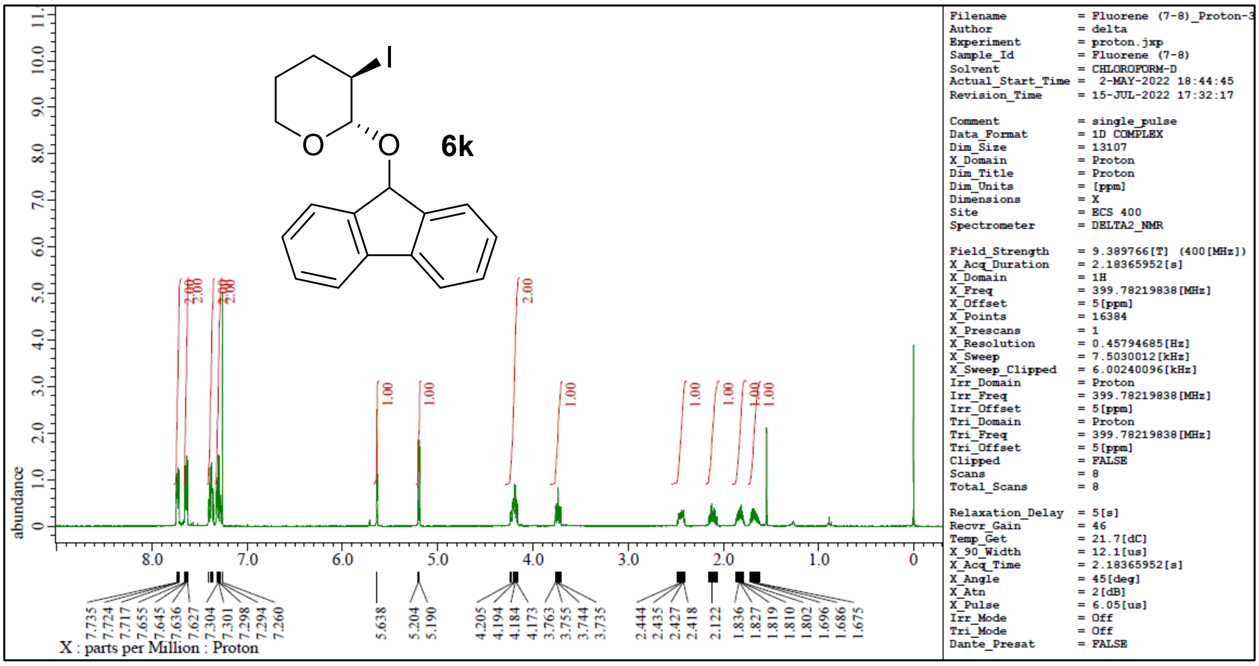

Compound **6k** (^{1}H NMR, 400 MHz, $CDCl_3$).

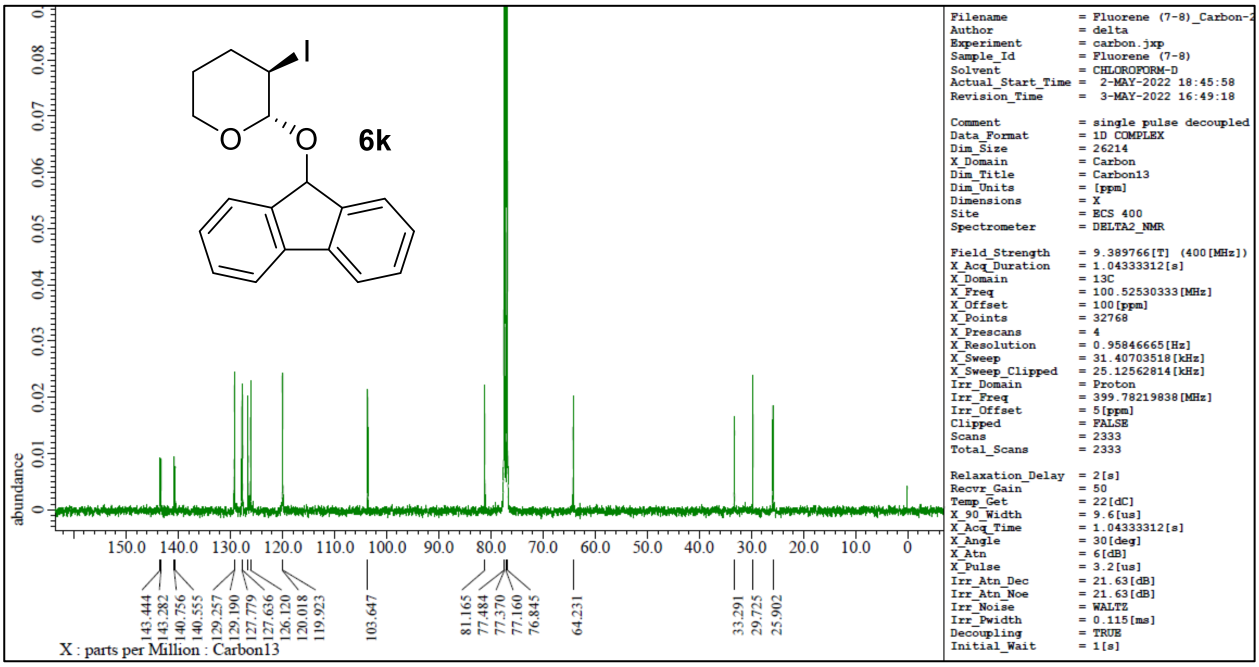

Compound **6k** (^{13}C NMR, 100 MHz, $CDCl_3$).

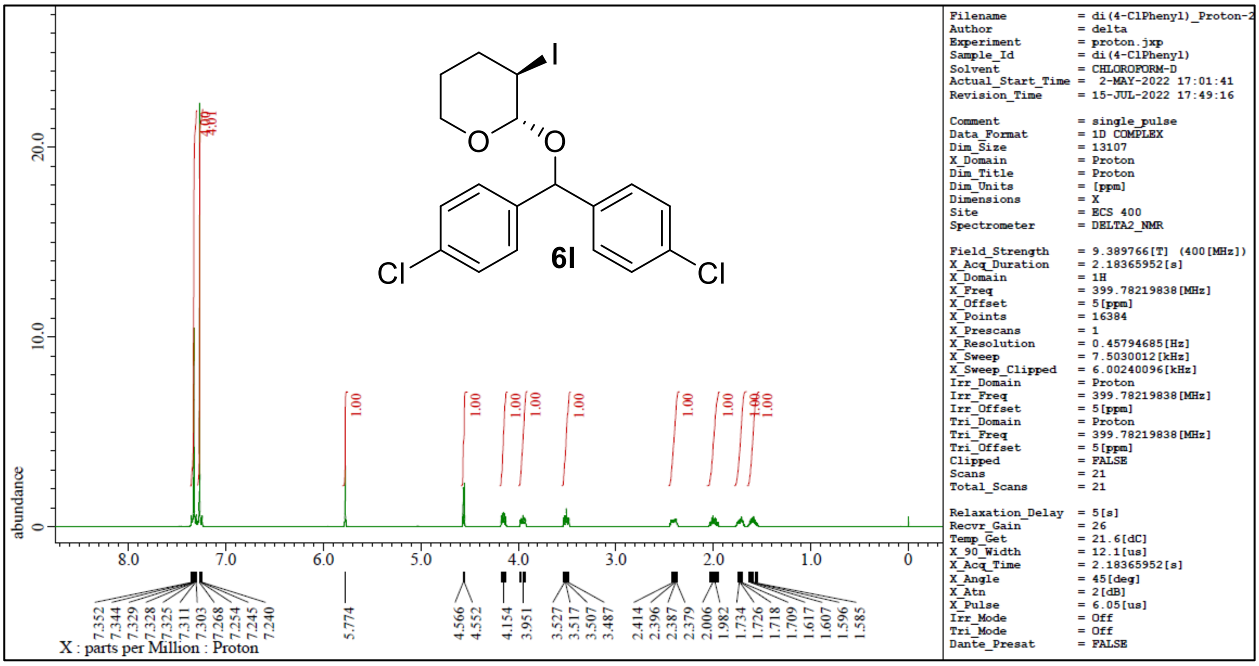

Compound **6l** (^{1}H NMR, 400 MHz, $CDCl_3$).

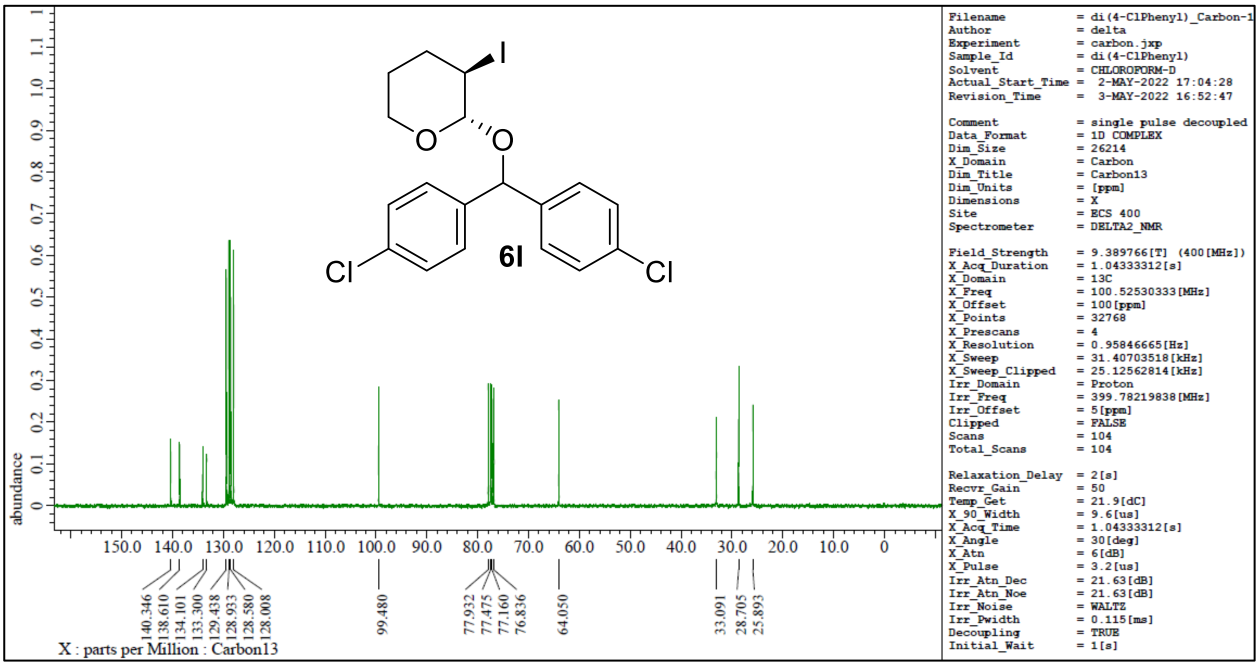

Compound **6l** (^{13}C NMR, 100 MHz, $CDCl_3$).

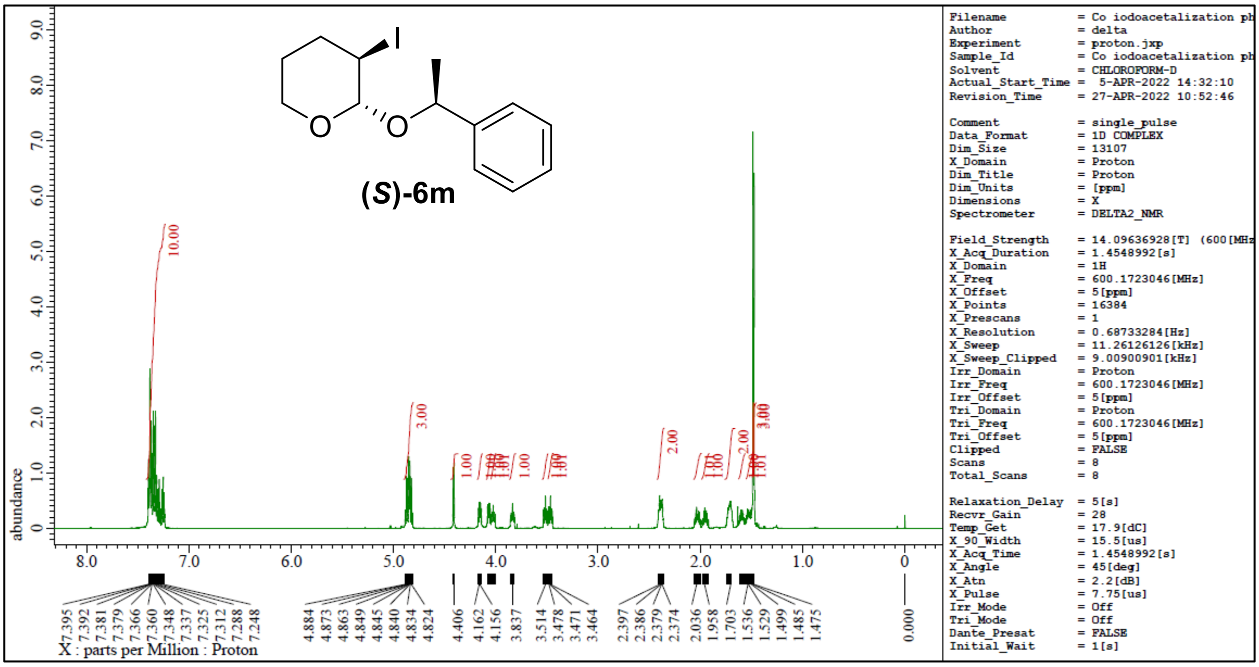

Compound (*S*)-**6m** (^{1}H NMR, 600 MHz, $CDCl_3$).

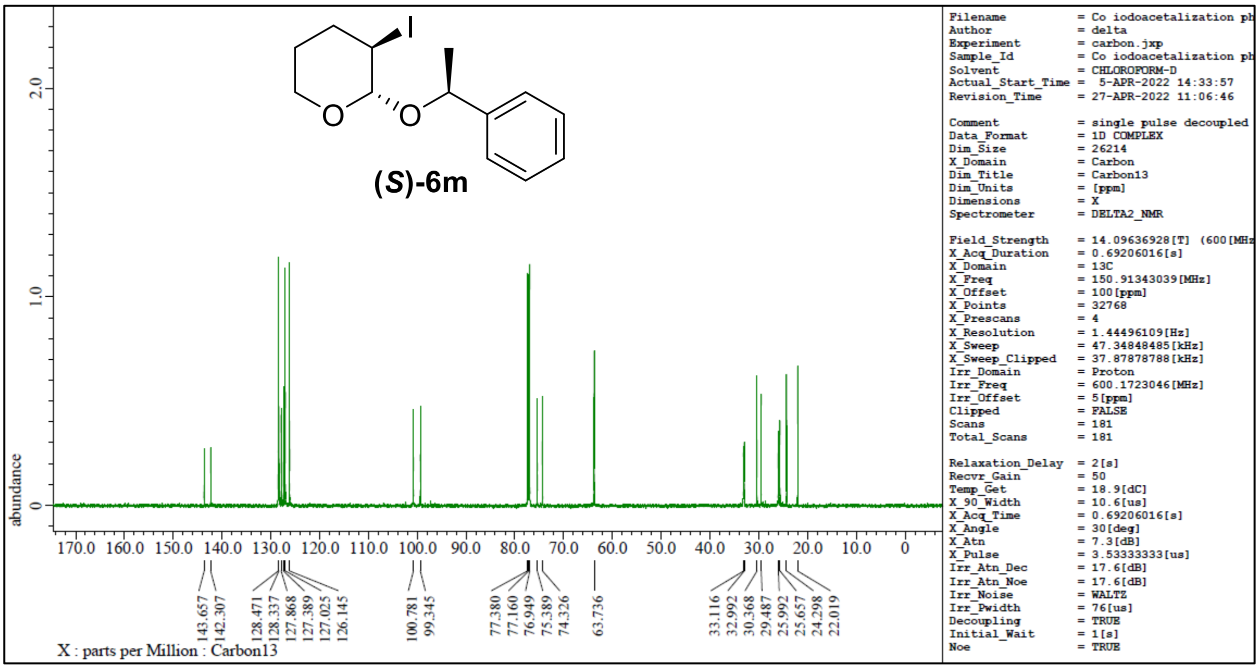

Compound (*S*)-**6m** (^{13}C NMR, 150 MHz, $CDCl_3$).

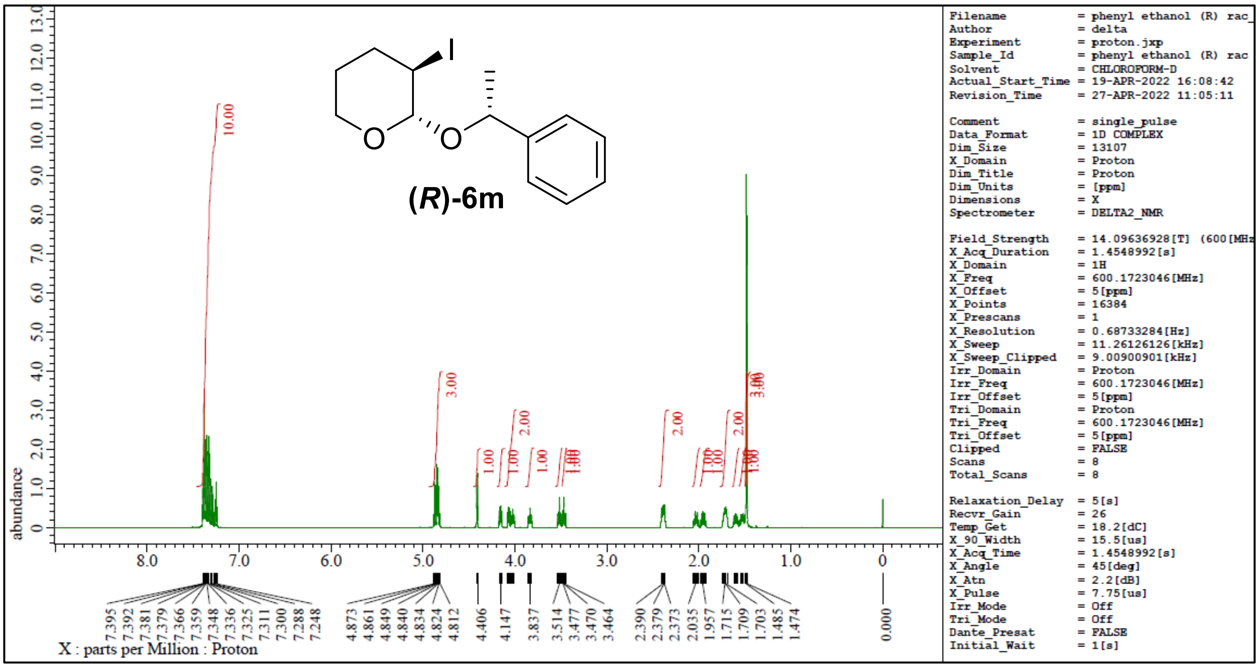

Compound (*R*)-**6m** (^{1}H NMR, 600 MHz, $CDCl_3$).

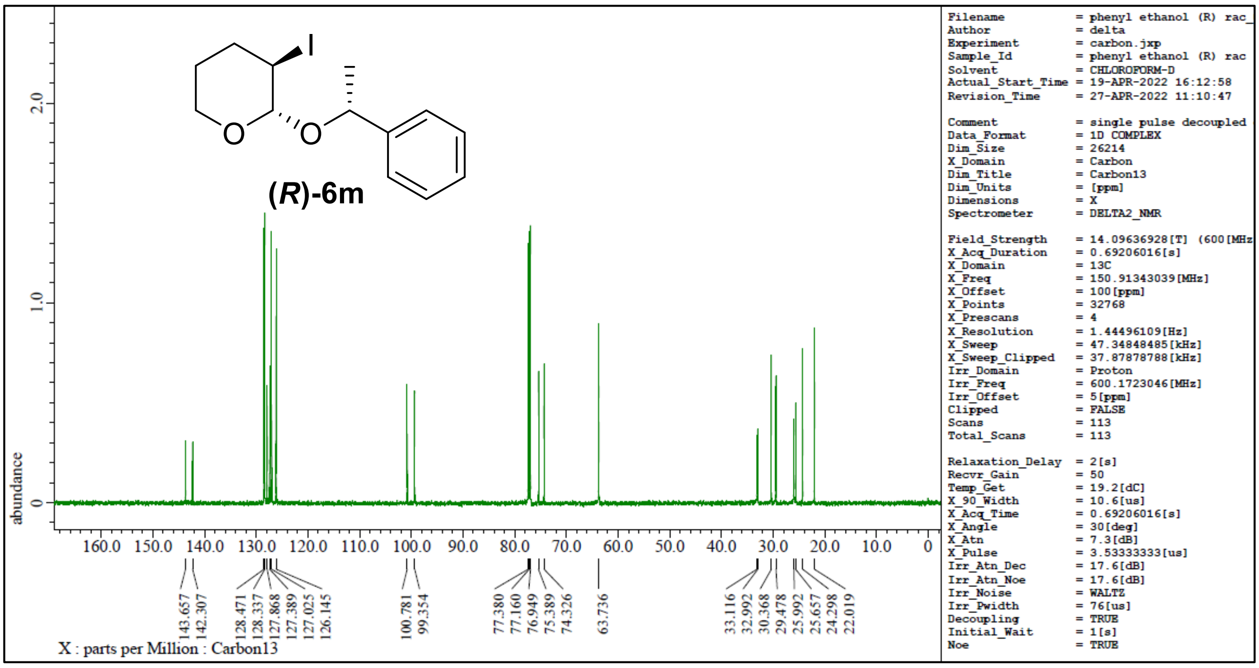

Compound (*R*)-**6m** (^{13}C NMR, 150 MHz, $CDCl_3$).

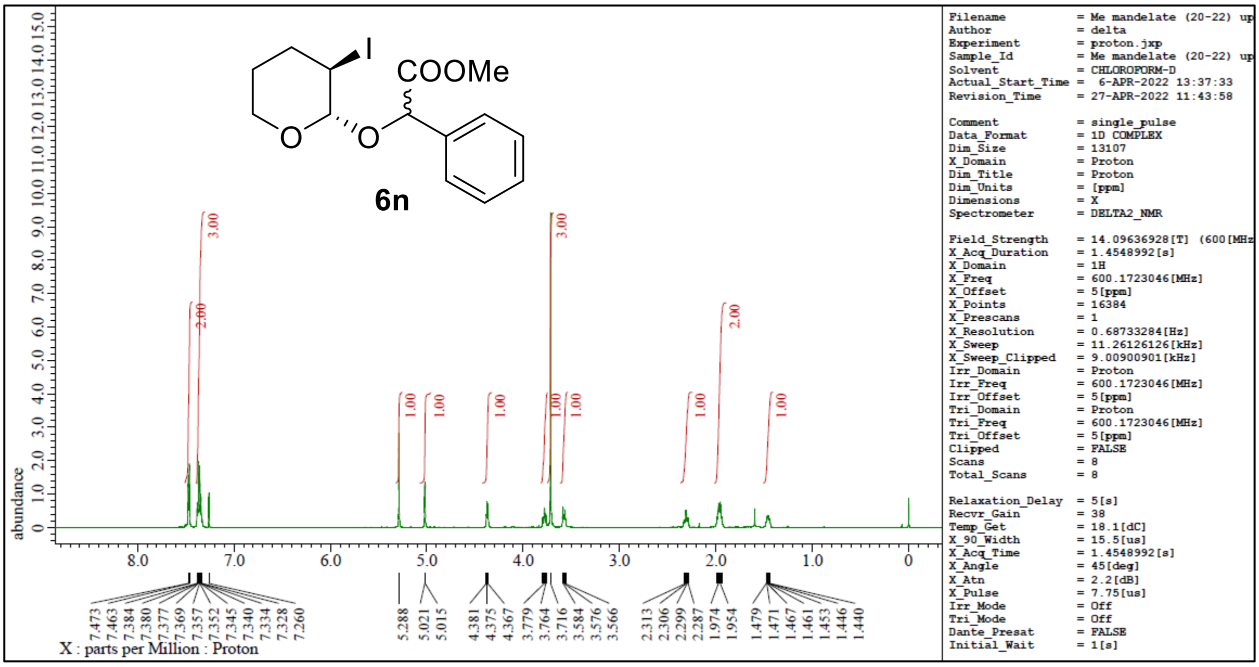

Compound **6n**-upper diastereomer (^{1}H NMR, 600 MHz, $CDCl_3$).

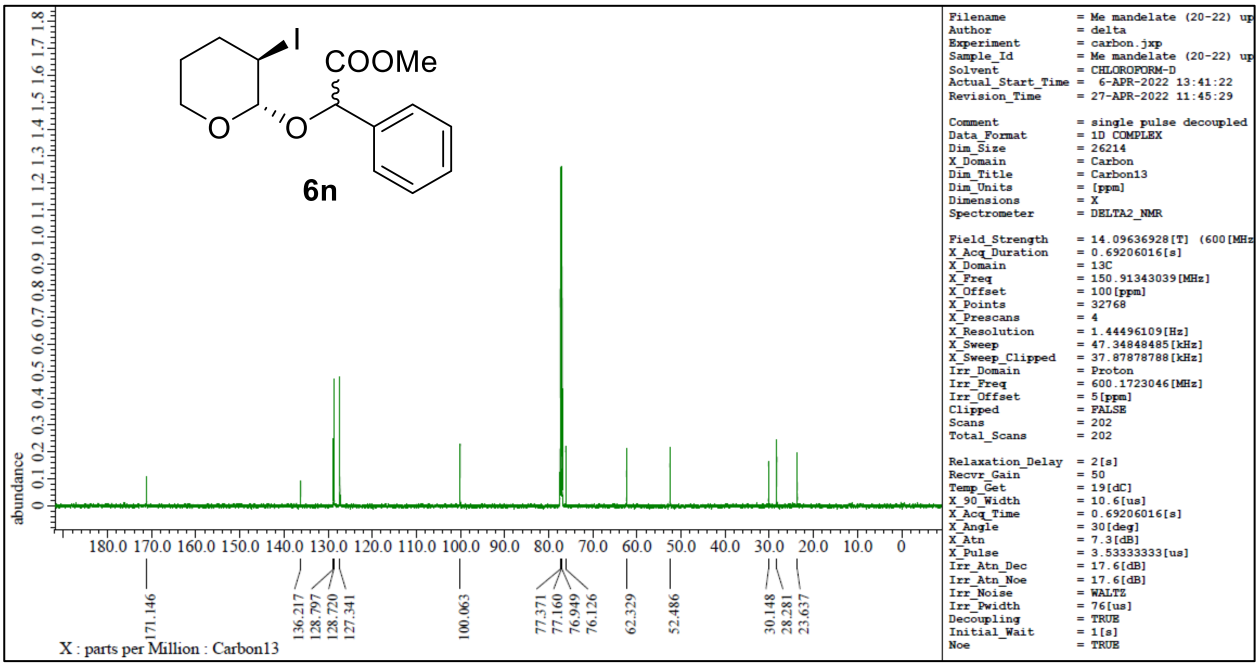

Compound **6n**-upper diastereomer (^{13}C NMR, 150 MHz, $CDCl_3$).

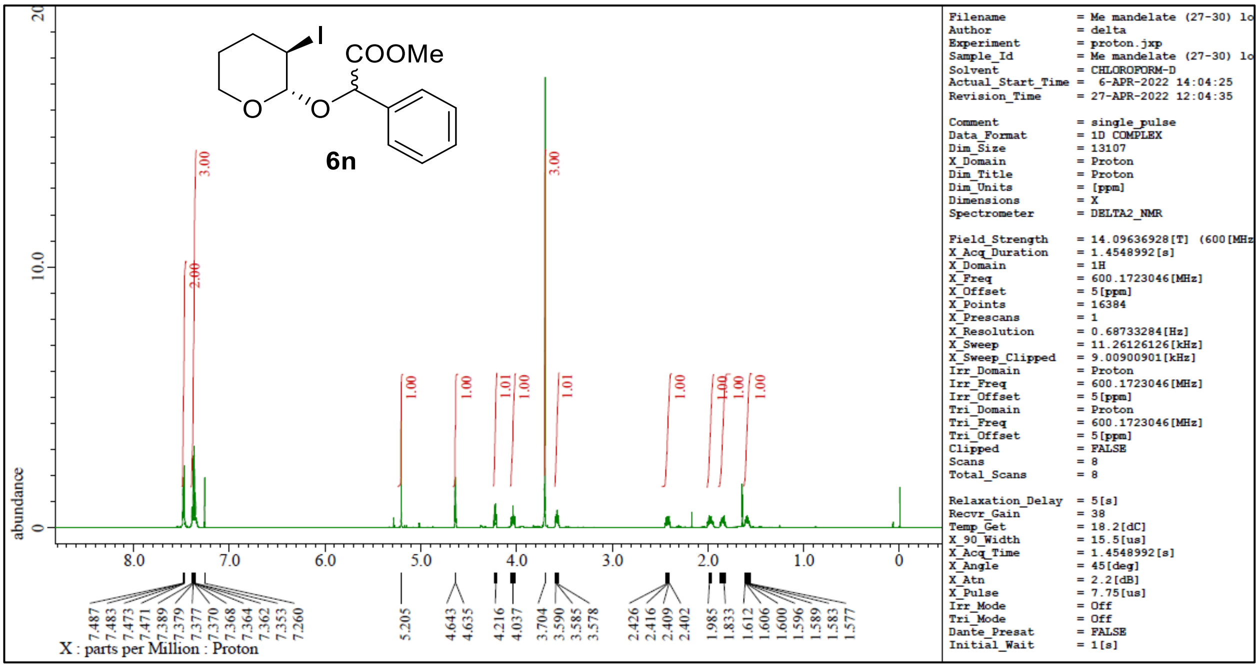

Compound **6n**-lower diastereomer (^{1}H NMR, 600 MHz, $CDCl_3$).

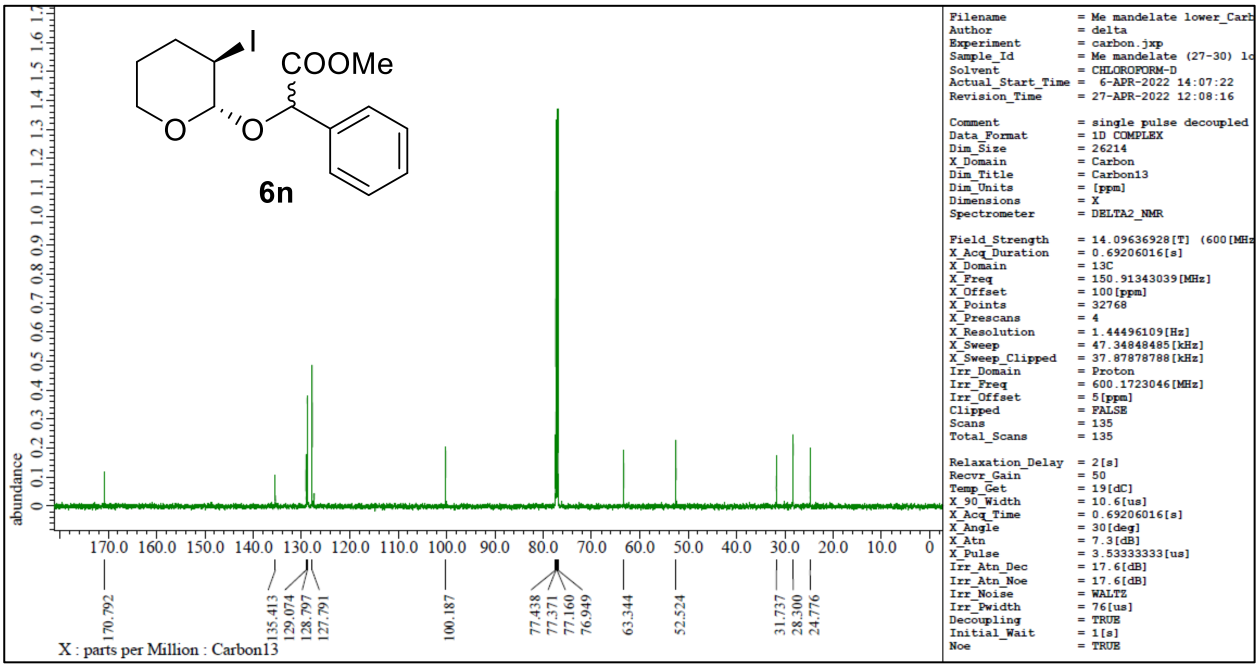

Compound **6n**-lower diastereomer (^{13}C NMR, 150 MHz, $CDCl_3$).

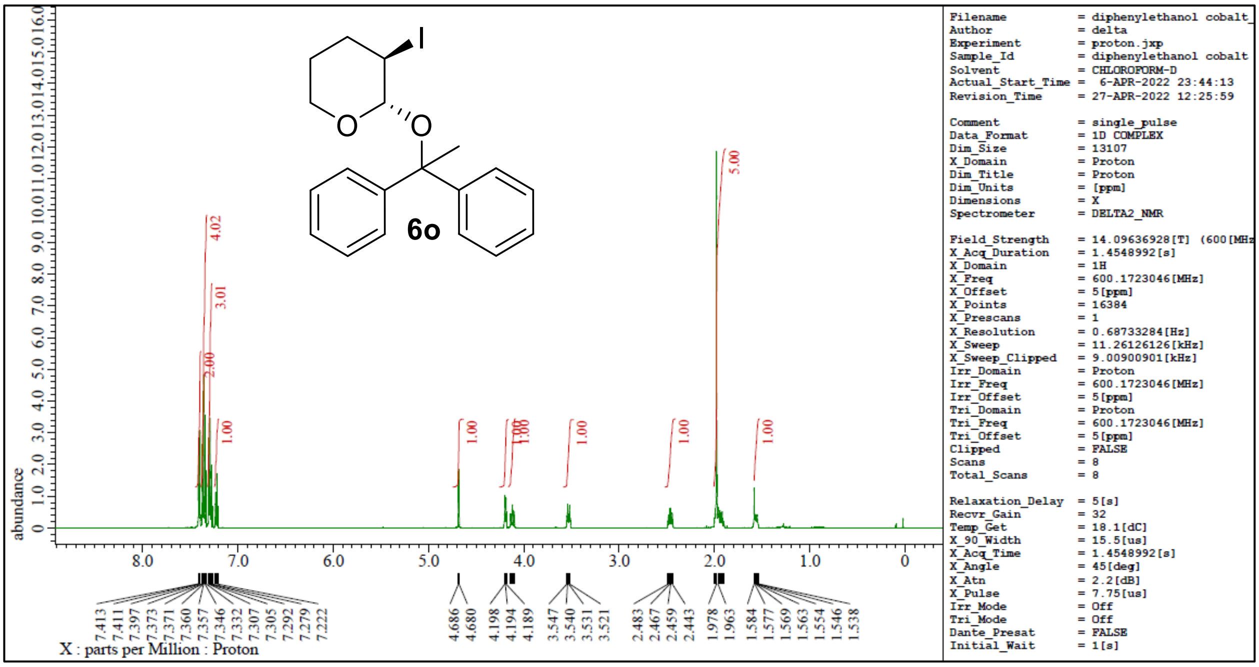

Compound **6o** (^1H NMR, 600 MHz, $CDCl_3$).

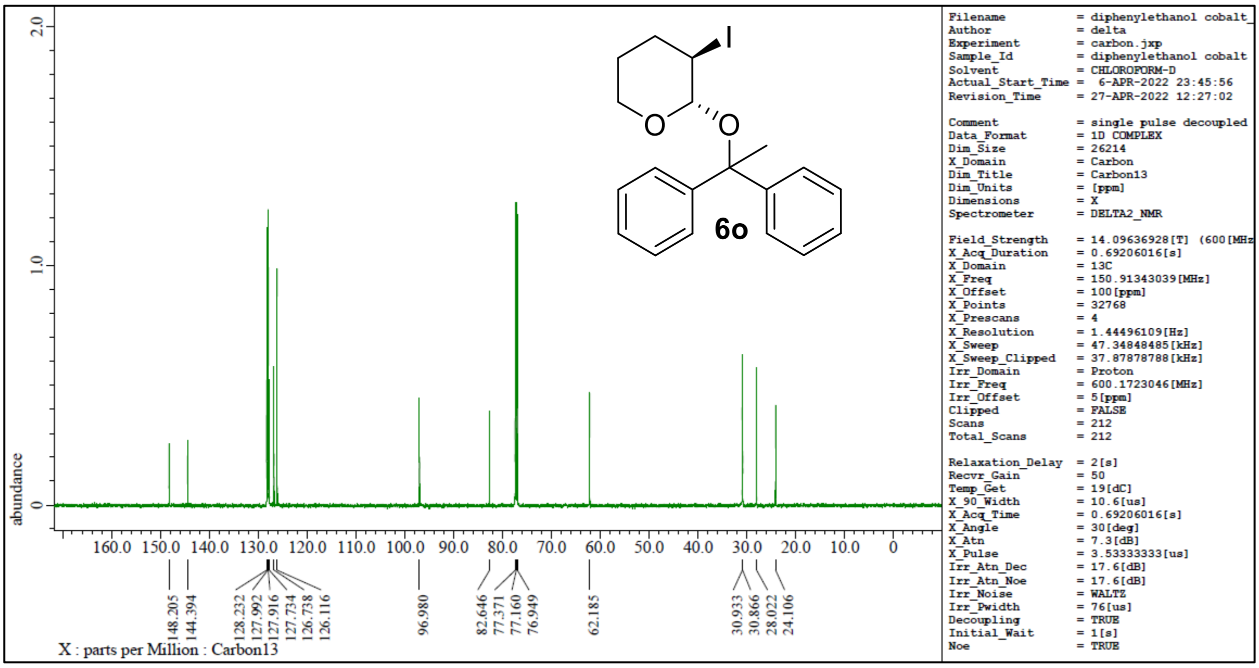

Compound **6o** (^{13}C NMR, 150 MHz, $CDCl_3$).

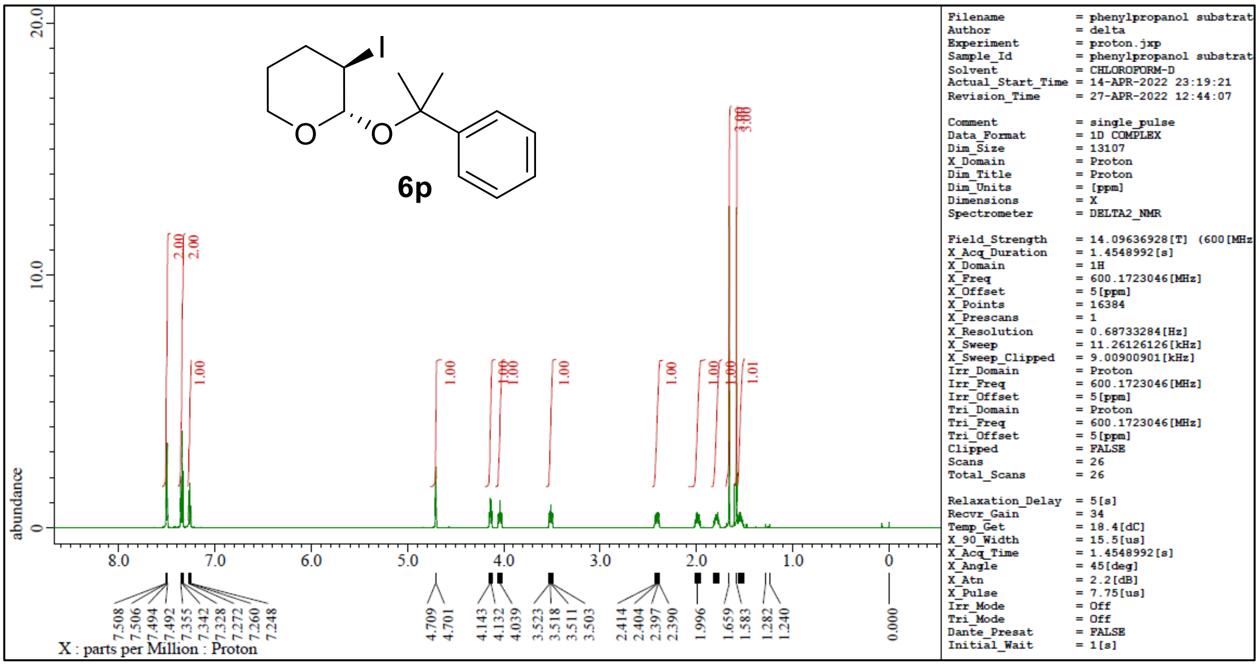

Compound **6p** (^{1}H NMR, 600 MHz, $CDCl_3$).

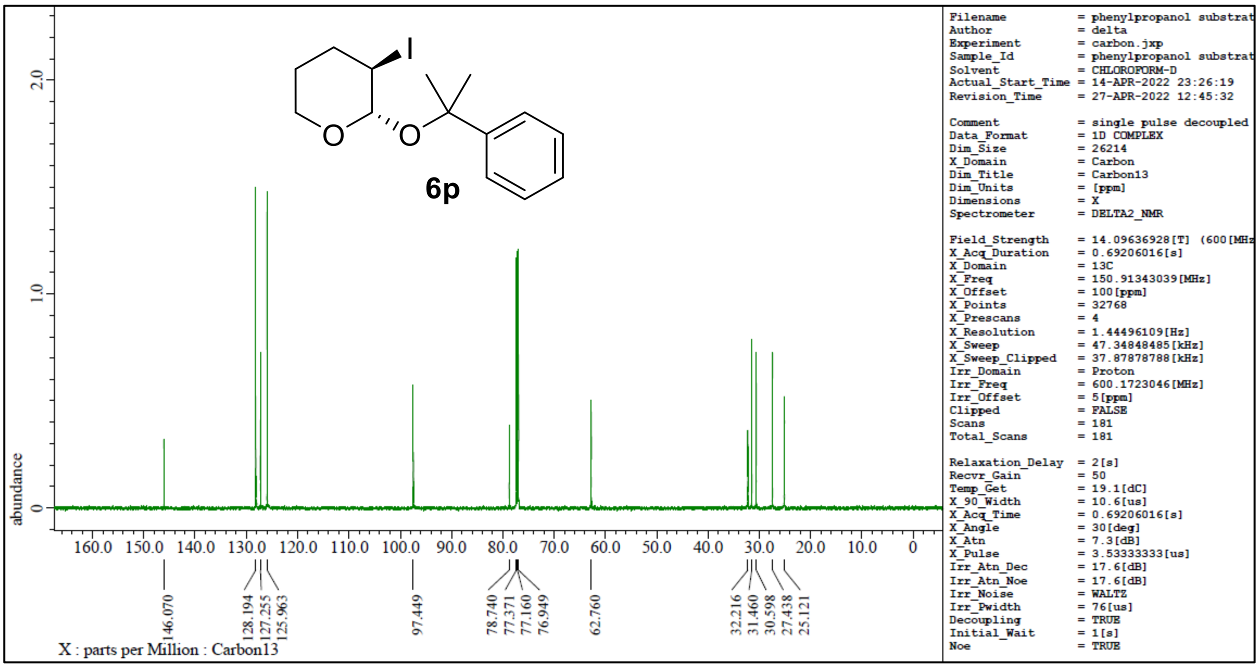

Compound **6p** (^{13}C NMR, 150 MHz, $CDCl_3$).

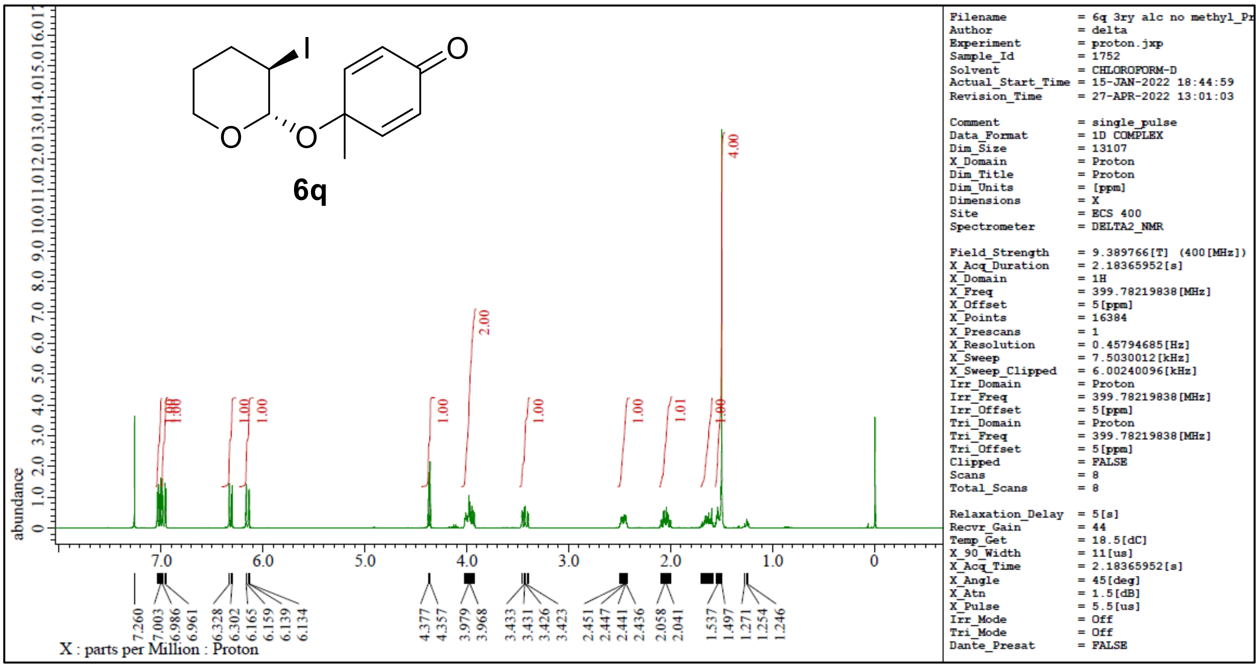

Compound **6q** (^{1}H NMR, 400 MHz, $CDCl_3$).

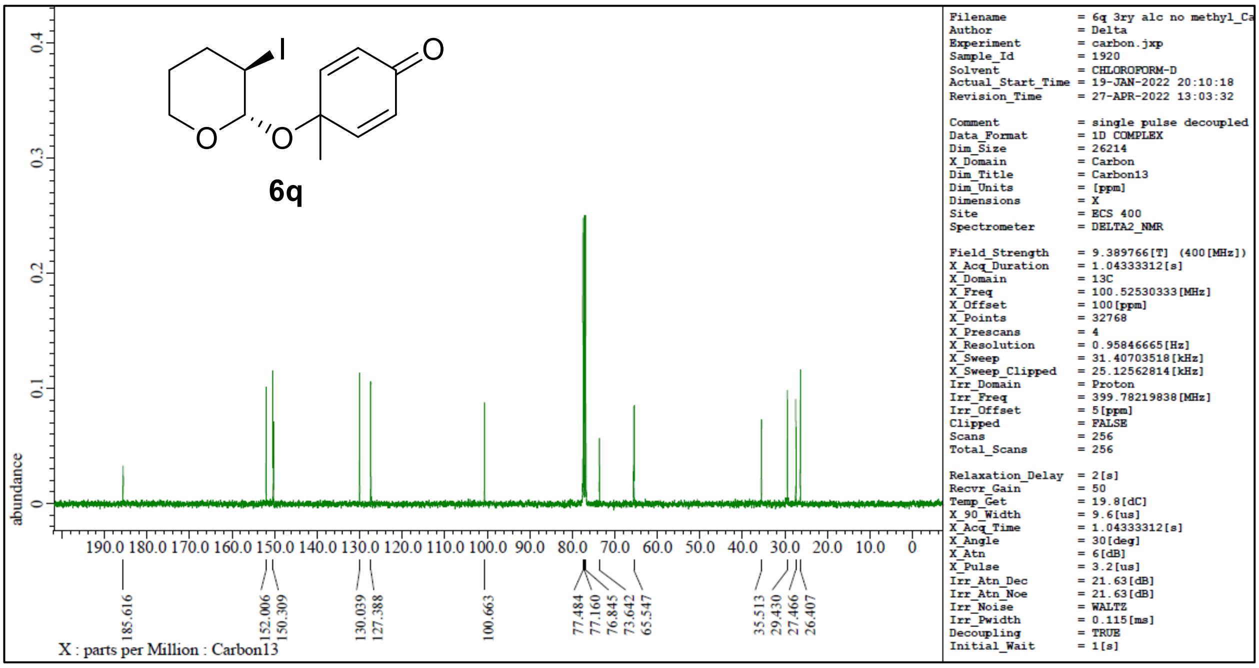

Compound **6q** (^{13}C NMR, 100 MHz, $CDCl_3$).

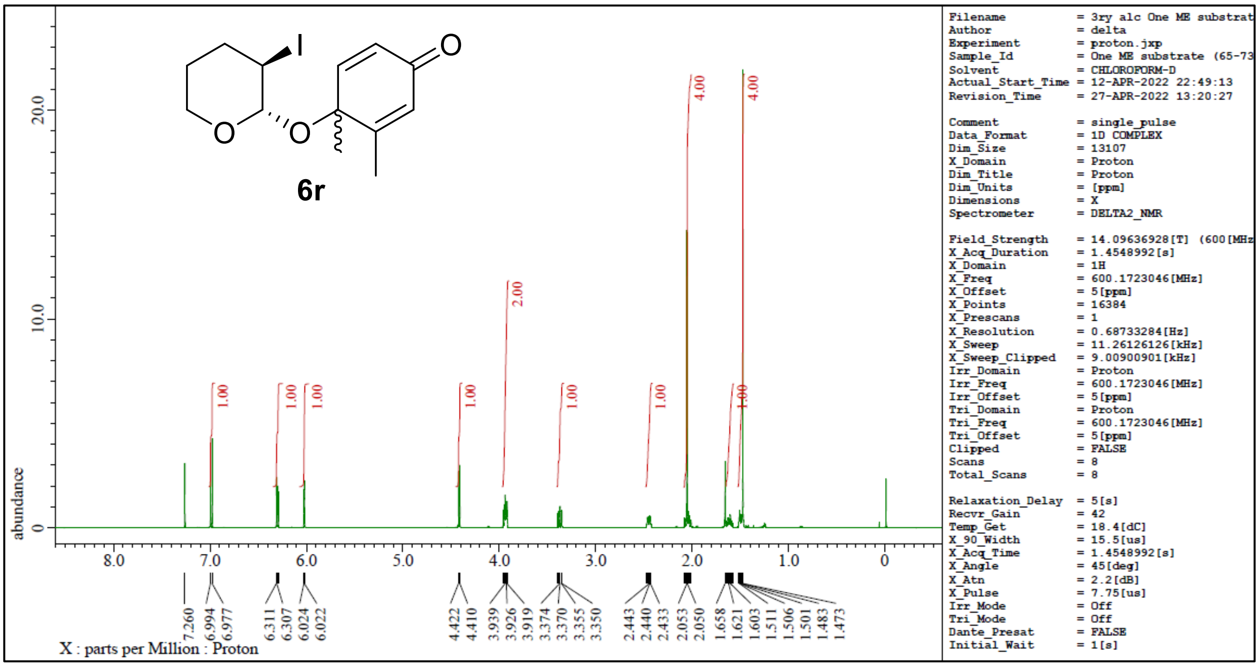

Compound **6r**-upper (^{1}H NMR, 600 MHz, $CDCl_3$).

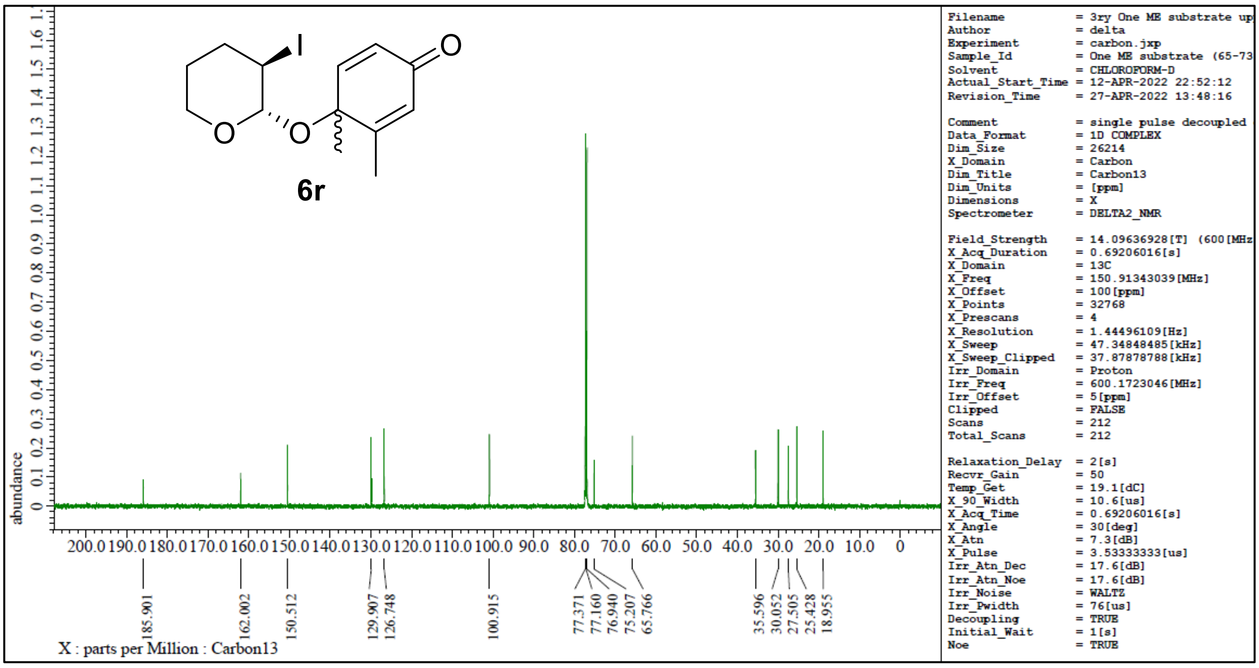

Compound **6r**-upper (^{13}C NMR, 150 MHz, $CDCl_3$).

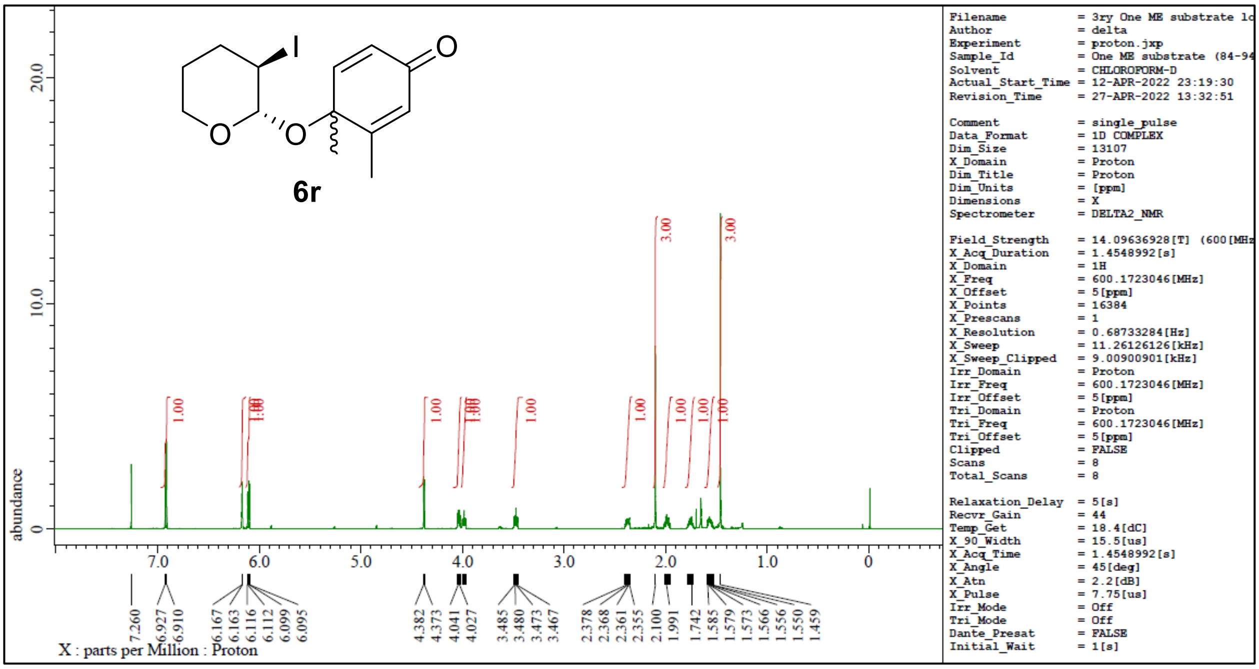

Compound **6r**-lower (^{1}H NMR, 600 MHz, $CDCl_3$).

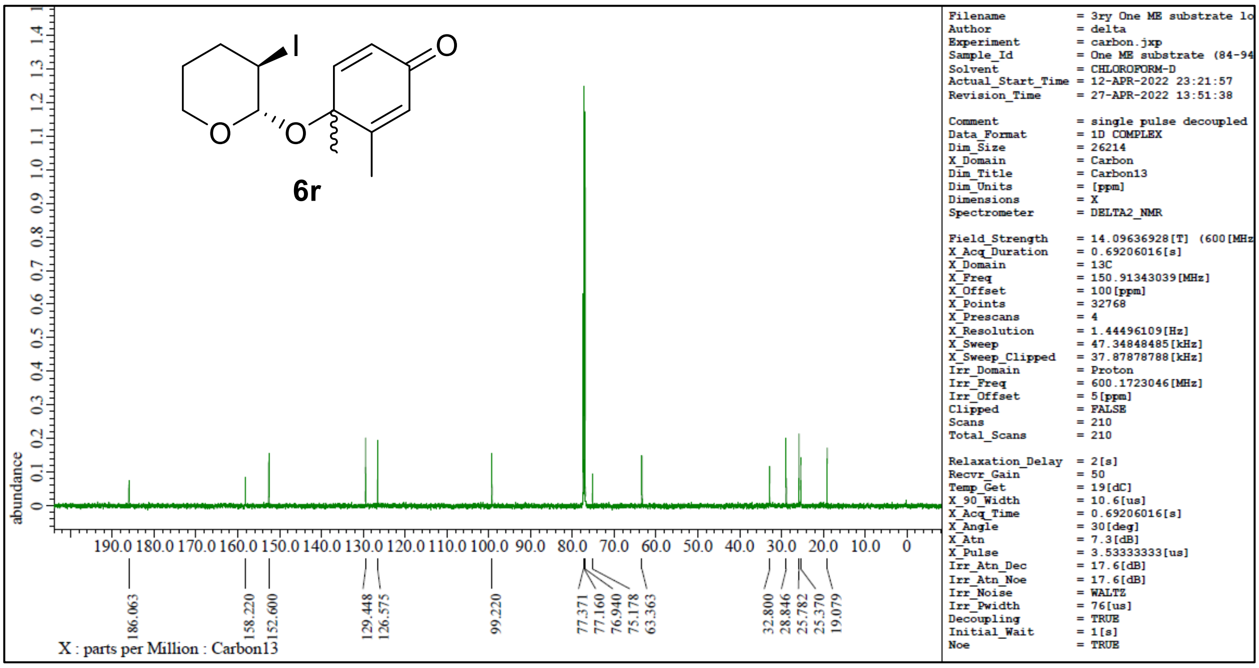

Compound **6r**-lower (^{13}C NMR, 150 MHz, $CDCl_3$).

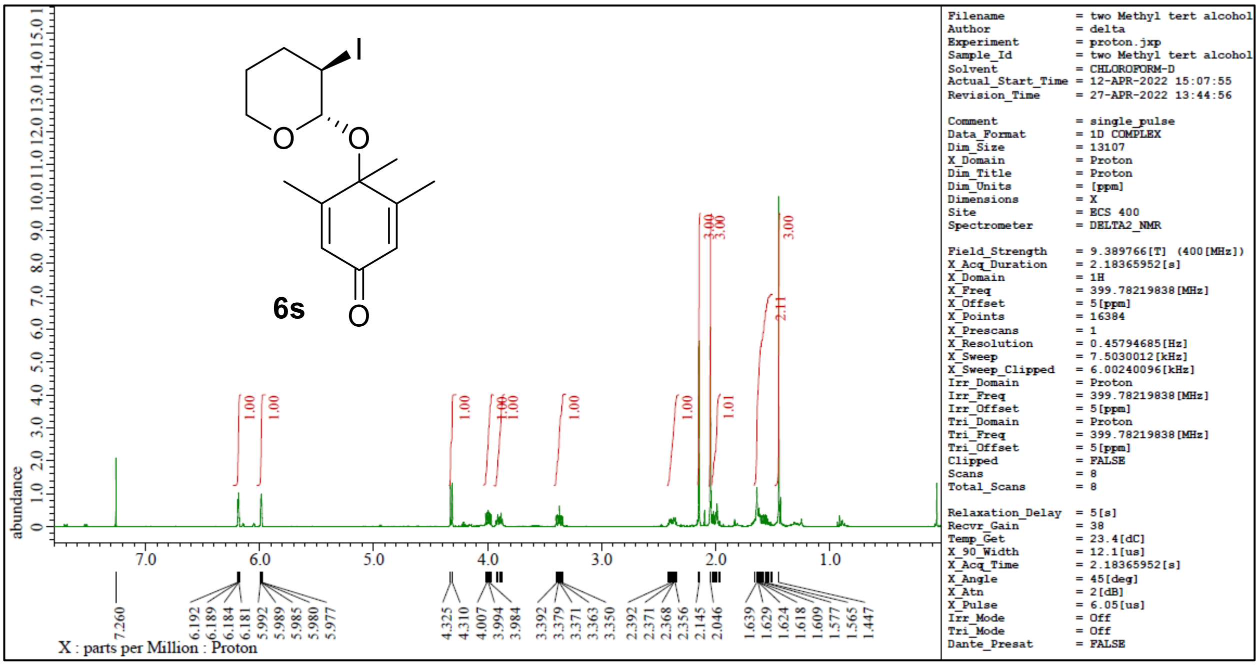

Compound **6S** (^{1}H NMR, 400 MHz, $CDCl_3$).

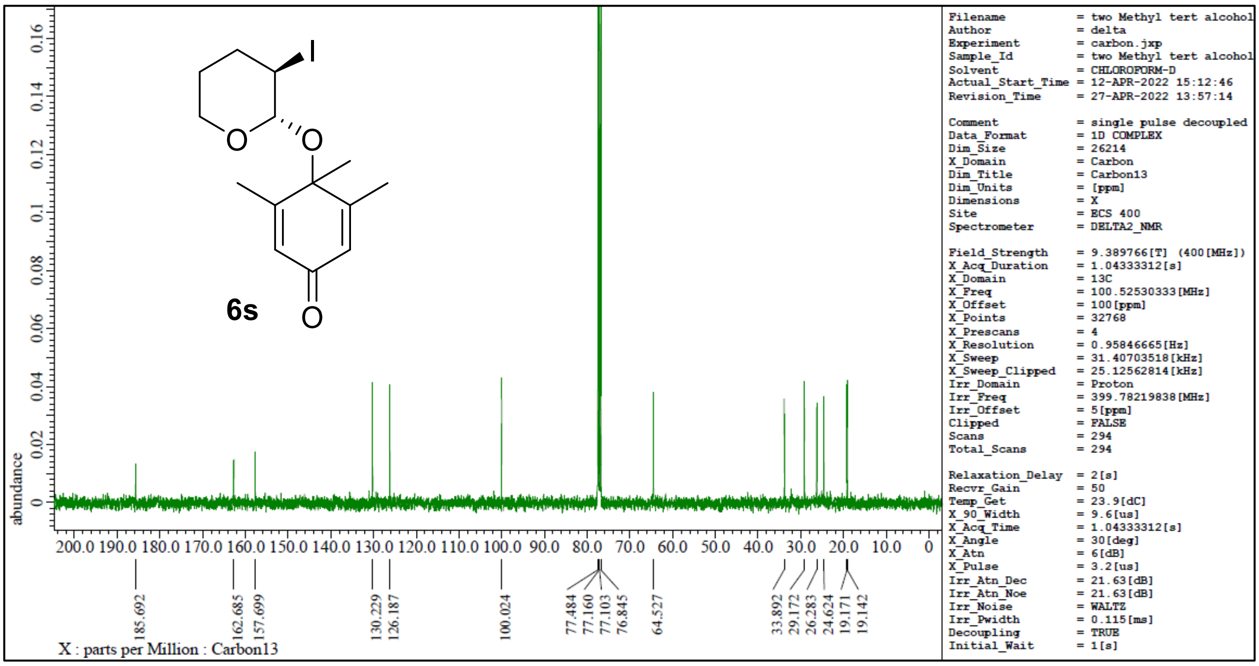

Compound **6S** (^{13}C NMR, 100 MHz, $CDCl_3$).

16. HPLC charts

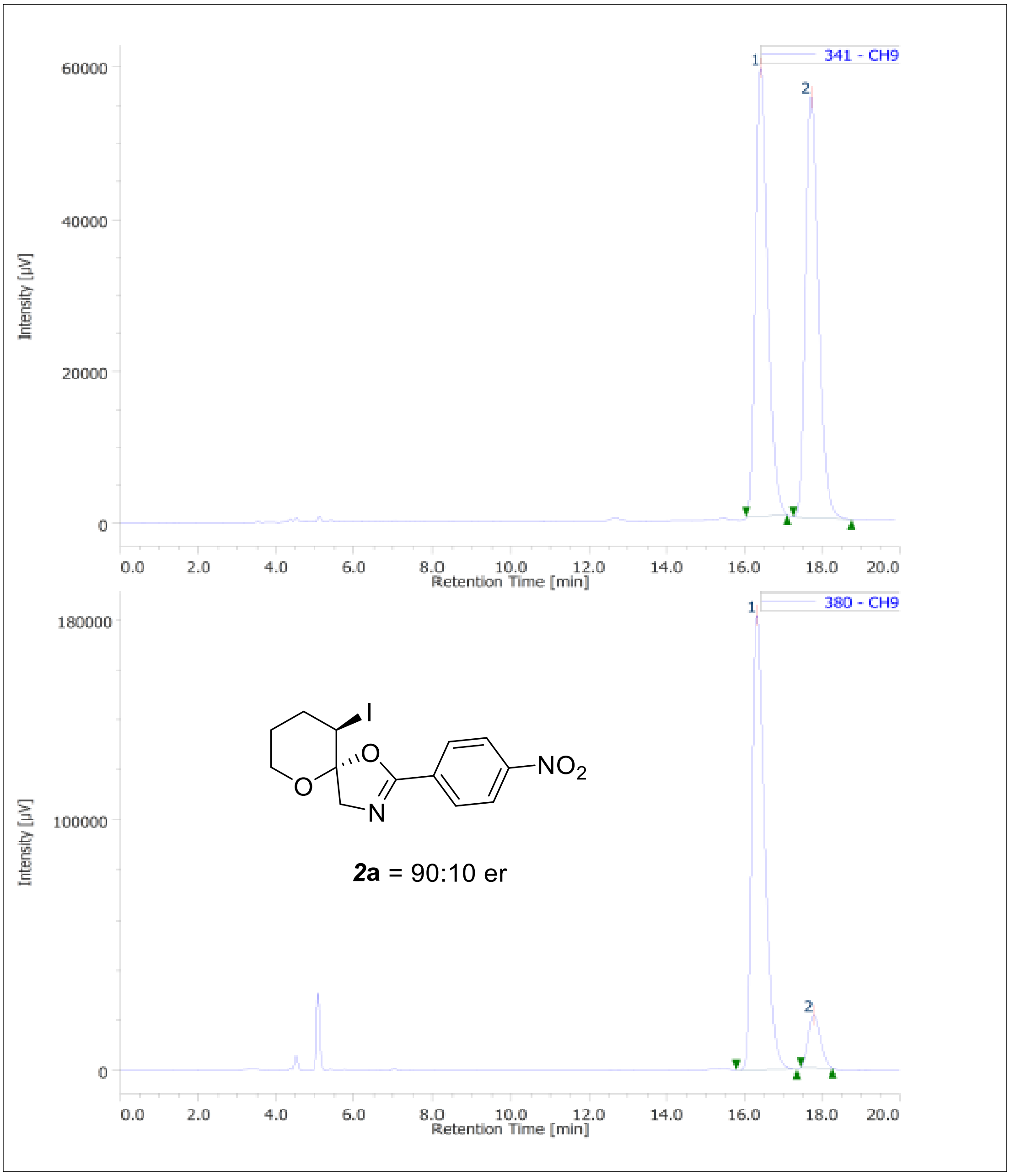

Channel & Peak Information Table

Chromatogram Name 341−CH9

Sample Name

Channel Name 270.0nm

#	Peak Name	CH	tR [min]	Area [µV·sec]	Height [µV]	Area%	Height%	Quantity	NTP	Resolution	Symmetry Factor	Warning
1	Unknown	9	16.420	1295583	58805	50.237	51.571	N/A	13035	2.212	1.336	
2	Unknown	9	17.720	1283363	55222	49.763	48.429	N/A	13817	N/A	1.308	

Chromatogram Name 380−CH9

Sample Name

Channel Name 270.0nm

#	Peak Name	CH	tR [min]	Area [µV·sec]	Height [µV]	Area%	Height%	Quantity	NTP	Resolution	Symmetry Factor	Warning
1	Unknown	9	16.323	4141823	181564	90.052	89.597	N/A	12214	2.501	1.503	
2	Unknown	9	17.790	457560	21082	9.948	10.403	N/A	14783	N/A	1.183	

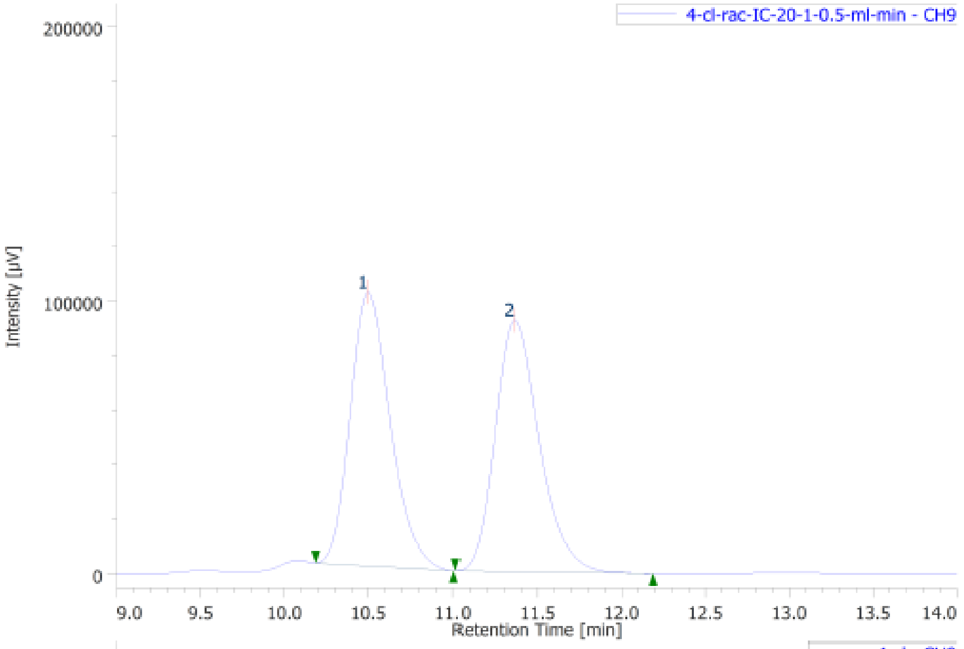

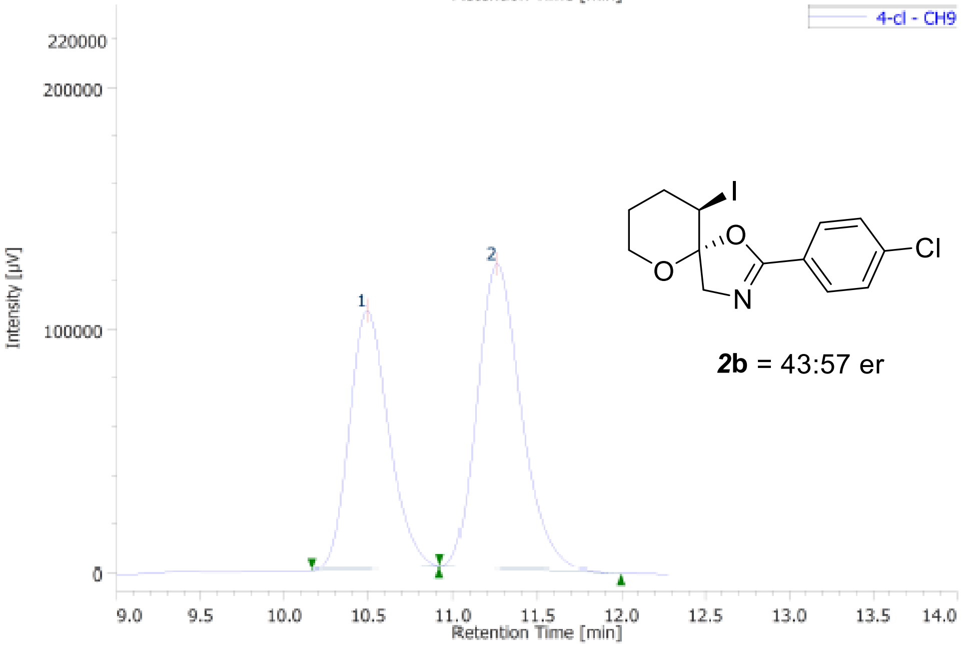

2b = 43:57 er

Channel & Peak Information Table

Chromatogram Name 4−cl−rac−IC−20−1−0.5−ml−min−CH9
Sample Name
Channel Name 270.0nm

#	Peak Name	CH	tR [min]	Area [µV·sec]	Height [µV]	Area%	Height%	Quantity	NTP	Resolution	Symmetry Factor	Warning
1	Unknown	9	10.500	1667347	100282	49.952	52.132	N/A	9392	1.918	1.222	
2	Unknown	9	11.363	1670527	92080	50.048	47.868	N/A	9384	N/A	1.255	

Chromatogram Name 4−cl−CH9
Sample Name
Channel Name 254.0nm

#	Peak Name	CH	tR [min]	Area [µV·sec]	Height [µV]	Area%	Height%	Quantity	NTP	Resolution	Symmetry Factor	Warning
1	Unknown	9	10.493	1735765	105928	43.325	45.830	N/A	9585	1.703	1.194	
2	Unknown	9	11.257	2270611	125207	56.675	54.170	N/A	9170	N/A	1.255	

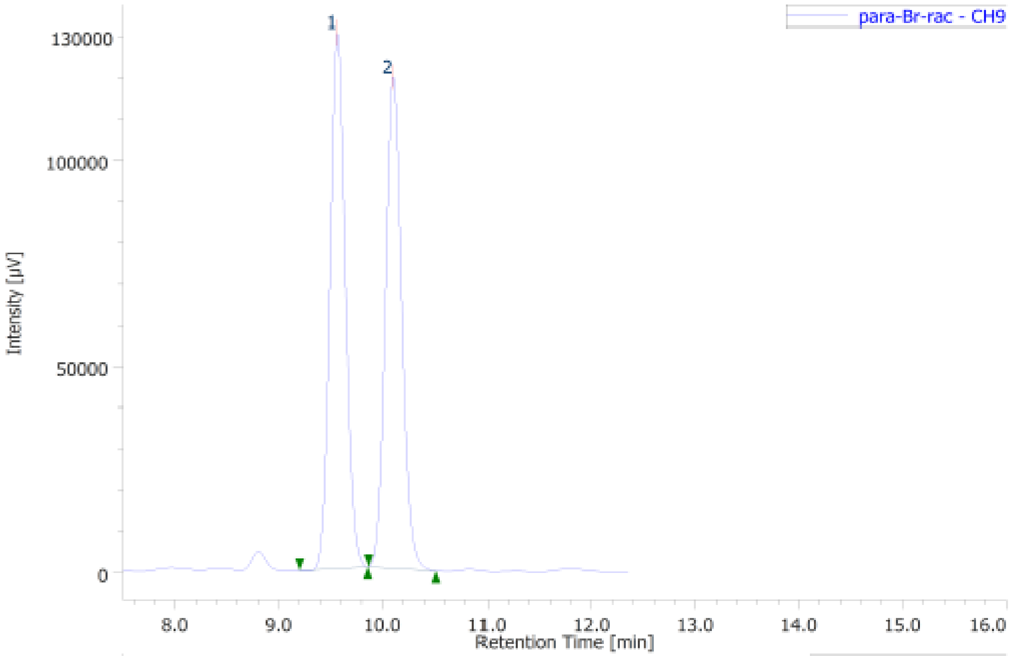

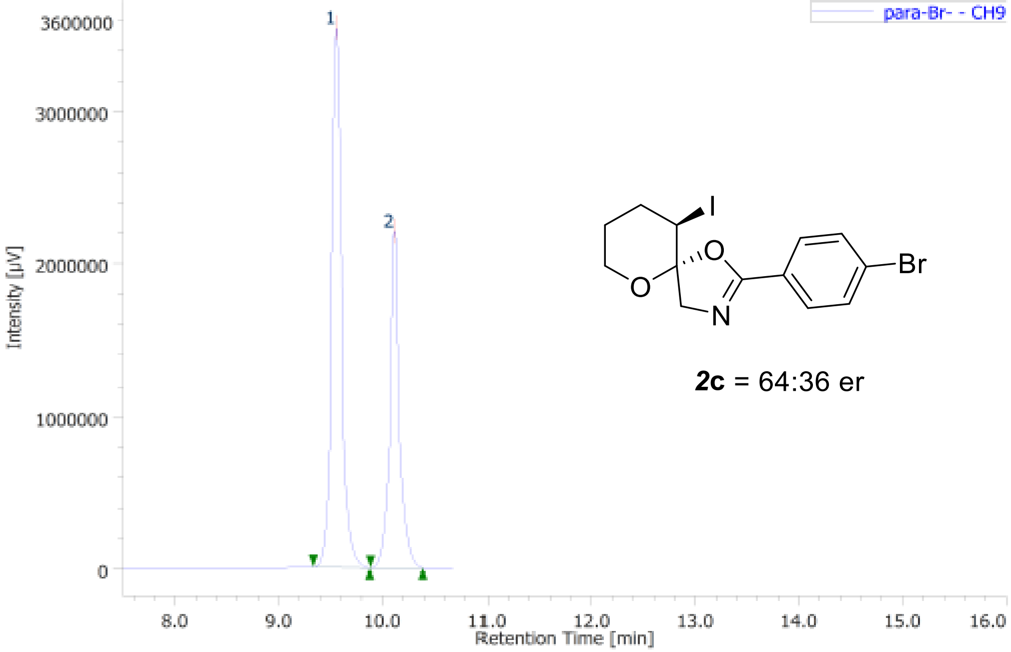

2c = 64:36 er

Channel & Peak Information Table

Chromatogram Name para-Br-rac-CH9
Sample Name
Channel Name 538.0nm

#	Peak Name	CH	tR [min]	Area [µV·sec]	Height [µV]	Area%	Height%	Quantity	NTP	Resolution	Symmetry Factor	Warning
1	Unknown	9	9.567	1334164	129689	50.688	52.155	N/A	20089	1.942	1.079	
2	Unknown	9	10.103	1297945	118970	49.312	47.845	N/A	20204	N/A	1.112	

Chromatogram Name para-Br--CH9
Sample Name
Channel Name 538.0nm

#	Peak Name	CH	tR [min]	Area [µV·sec]	Height [µV]	Area%	Height%	Quantity	NTP	Resolution	Symmetry Factor	Warning
1	Unknown	9	9.557	24451834	3539556	63.824	61.646	N/A	48111	3.881	1.117	
2	Unknown	9	10.117	13859515	2202200	36.176	38.354	N/A	123662	N/A	1.096	

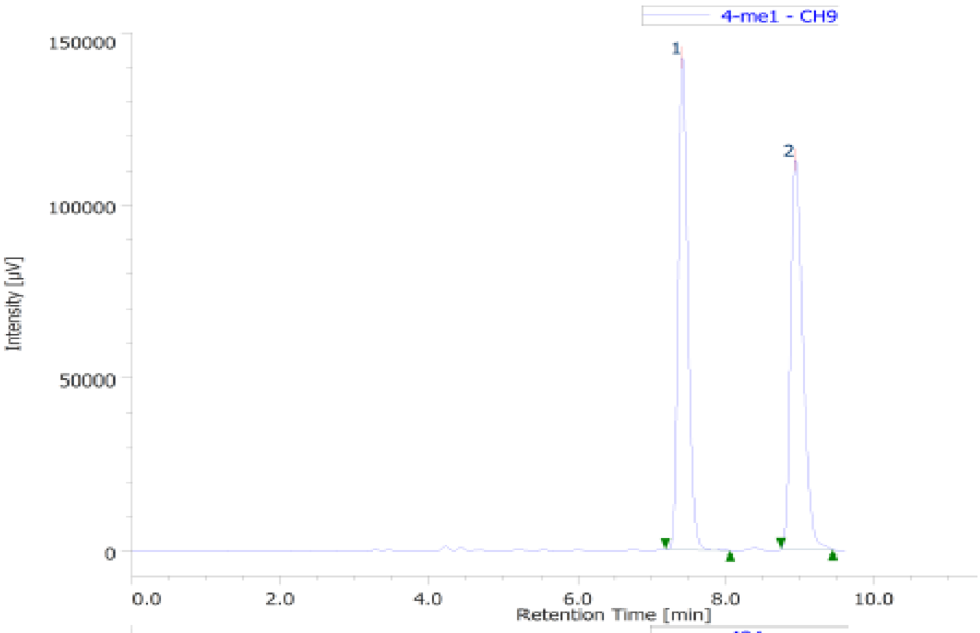

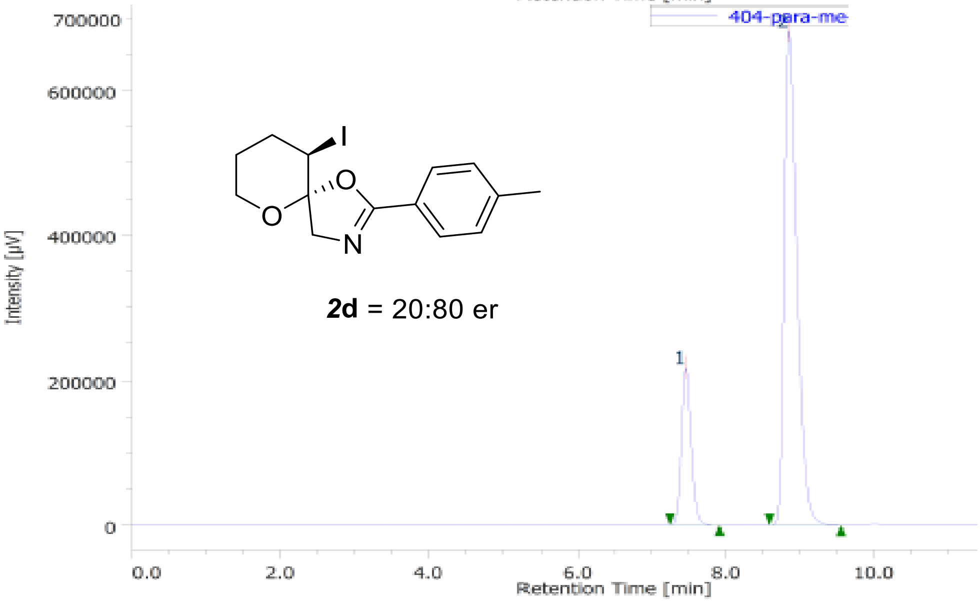

Channel & Peak Information Table

Chromatogram Name 4−me1−CH9
Sample Name
Channel Name 270.0nm

#	Peak Name	CH	tR [min]	Area [µV·sec]	Height [µV]	Area%	Height%	Quantity	NTP	Resolution	Symmetry Factor	Warning
1	Unknown	9	7.410	1293944	142453	49.818	55.868	N/A	15727	5.693	1.232	
2	Unknown	9	8.937	1303391	112527	50.182	44.132	N/A	14061	N/A	1.414	

Chromatogram Name 404−para−me
Sample Name
Channel Name 254.0nm

#	Peak Name	CH	tR [min]	Area [µV·sec]	Height [µV]	Area%	Height%	Quantity	NTP	Resolution	Symmetry Factor	Warning
1	Unknown	9	7.457	1982407	217413	19.646	24.167	N/A	15868	5.138	1.158	
2	Unknown	9	8.850	8108251	682205	80.354	75.833	N/A	13293	N/A	1.513	

Chromatogram

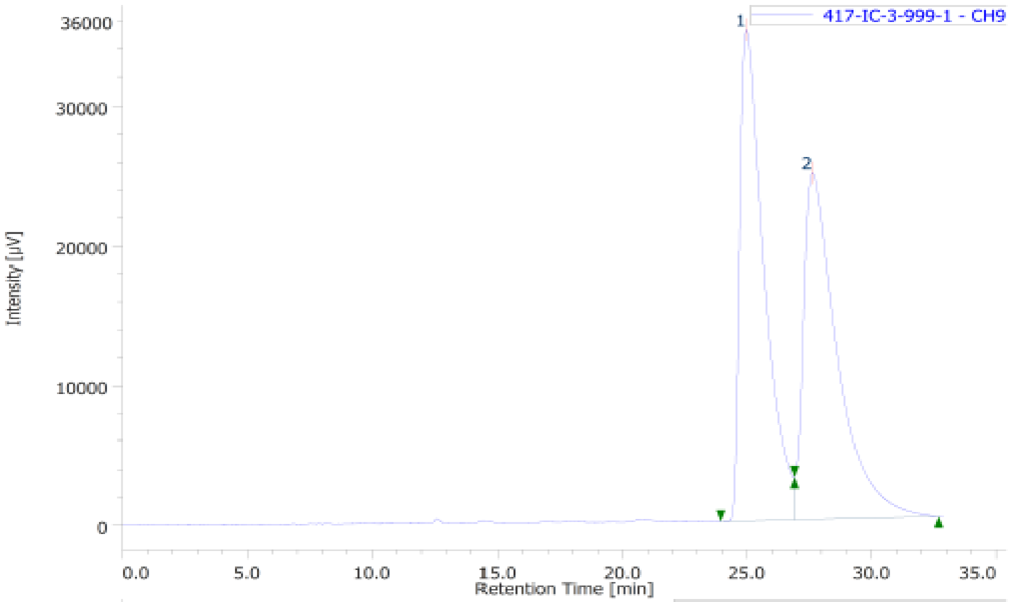

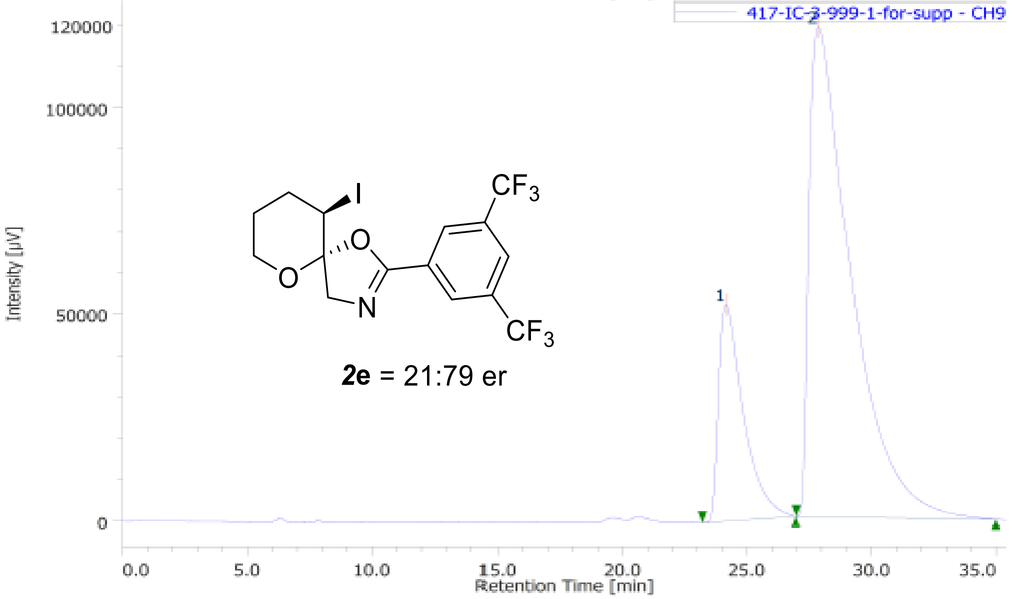

Channel & Peak Information Table

Chromatogram Name 417-IC-3-999-1-CH9
Sample Name
Channel Name 270.0nm

#	Peak Name	CH	tR [min]	Area [µV·sec]	Height [µV]	Area%	Height%	Quantity	NTP	Resolution	Symmetry Factor	Warning
1	Unknown	9	24.977	2398944	35021	50.603	58.550	N/A	3274	1.326	N/A	
2	Unknown	9	27.637	2341780	24793	49.397	41.450	N/A	2355	N/A	N/A	

Chromatogram Name 417-IC-3-999-1-for-supp-CH9
Sample Name
Channel Name 254.0nm

#	Peak Name	CH	tR [min]	Area [µV·sec]	Height [µV]	Area%	Height%	Quantity	NTP	Resolution	Symmetry Factor	Warning
1	Unknown	9	24.153	3633069	52049	20.765	30.534	N/A	3046	1.591	2.265	
2	Unknown	9	27.870	13863005	118415	79.235	69.466	N/A	1445	N/A	3.374	

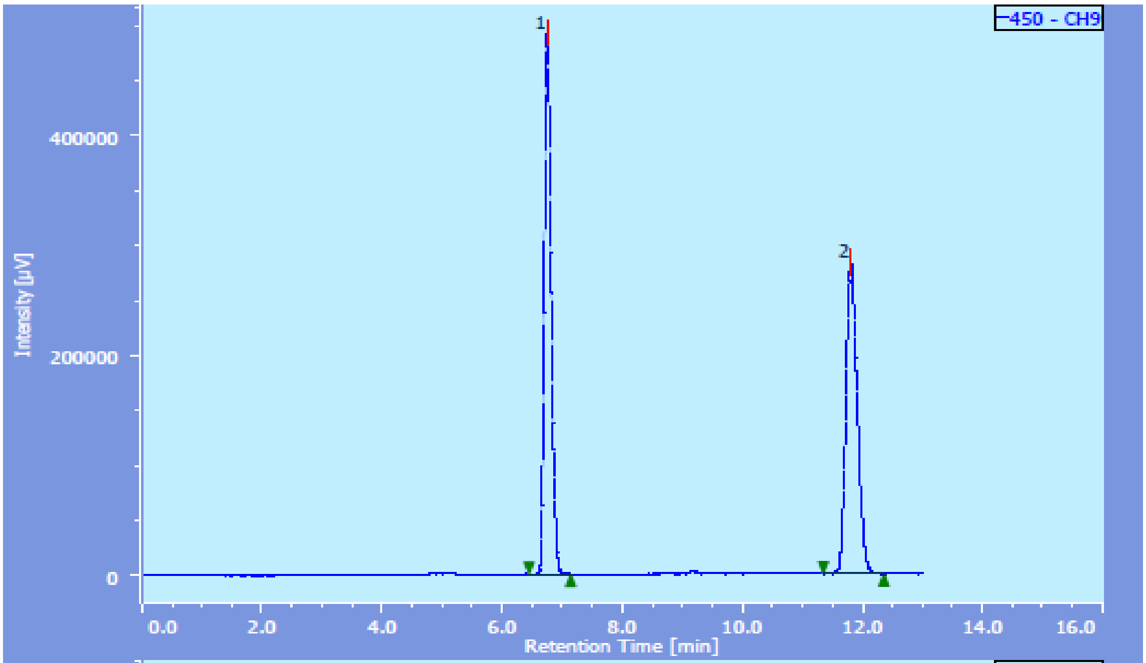

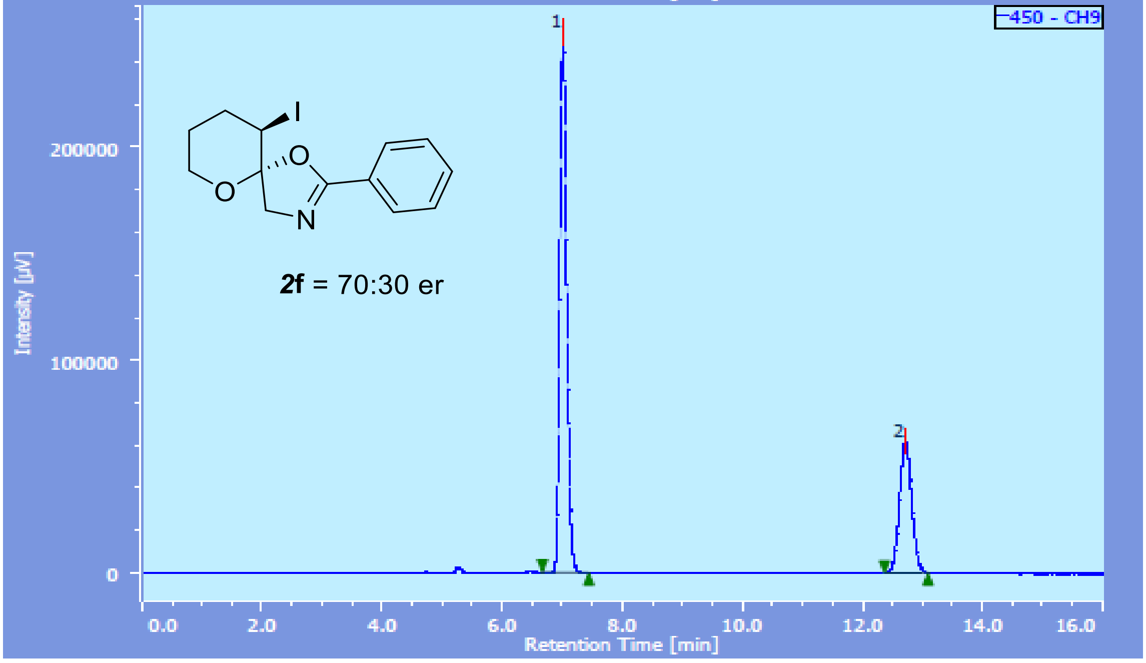

Channel & Peak Information Table

Chromatogram Name 450-CH9

Sample Name

Channel Name 270.0nm

#	Peak Name	CH	tR [min]	Area [µV·sec]	Height [µV]	Area%	Height%	Quantity	NTP	Resolution	Symmetry Factor	Warning
1	Unknown	9	6.747	3809451	491527	50.330	63.446	N/A	17616	18.339	1.359	
2	Unknown	9	11.803	3759564	283192	49.670	36.554	N/A	18236	N/A	1.211	

Chromatogram Name 450-CH9

Sample Name

Channel Name 254.0nm

#	Peak Name	CH	tR [min]	Area [µV·sec]	Height [µV]	Area%	Height%	Quantity	NTP	Resolution	Symmetry Factor	Warning
1	Unknown	9	7.013	2009703	253913	69.503	80.344	N/A	18282	19.639	1.178	
2	Unknown	9	12.713	881828	62118	30.497	19.656	N/A	18433	N/A	1.067	

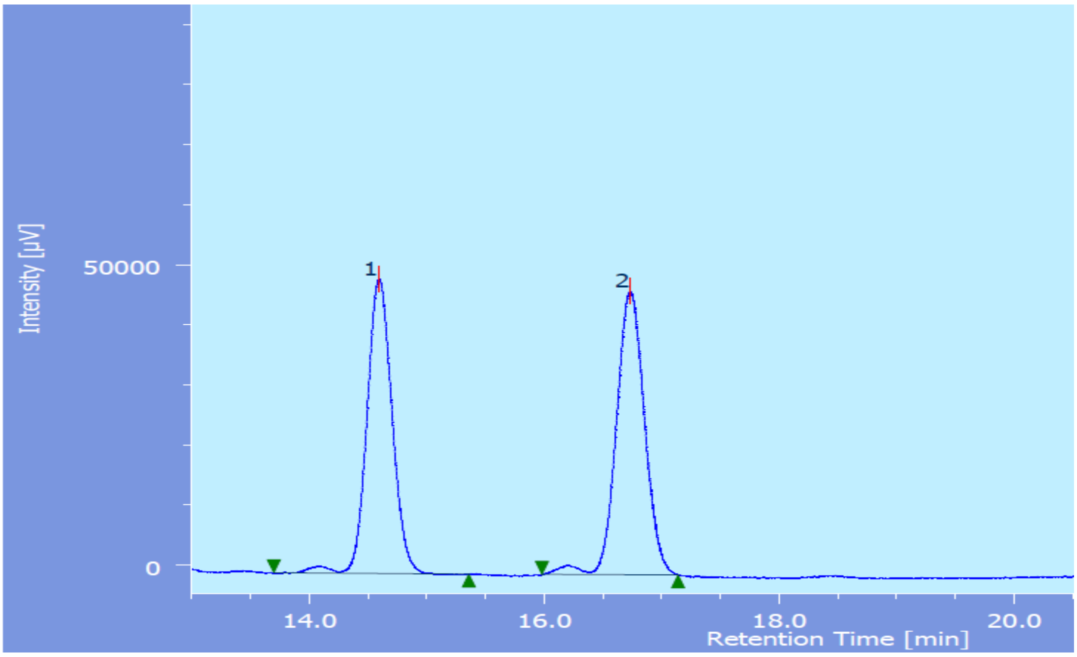

Channel Name 273.0nm

#	Peak Name	CH	tR [min]	Area [µV·sec]	Height [µV]	Area%	Height%	Quantity	NTP	Resolution	Symmetry Factor	Warning
1	Unknown	9	14.595	734550	49006	48.102	50.932	N/A	22874	5.234	1.085	
2	Unknown	9	16.732	792531	47212	51.898	49.068	N/A	23924	N/A	1.061	

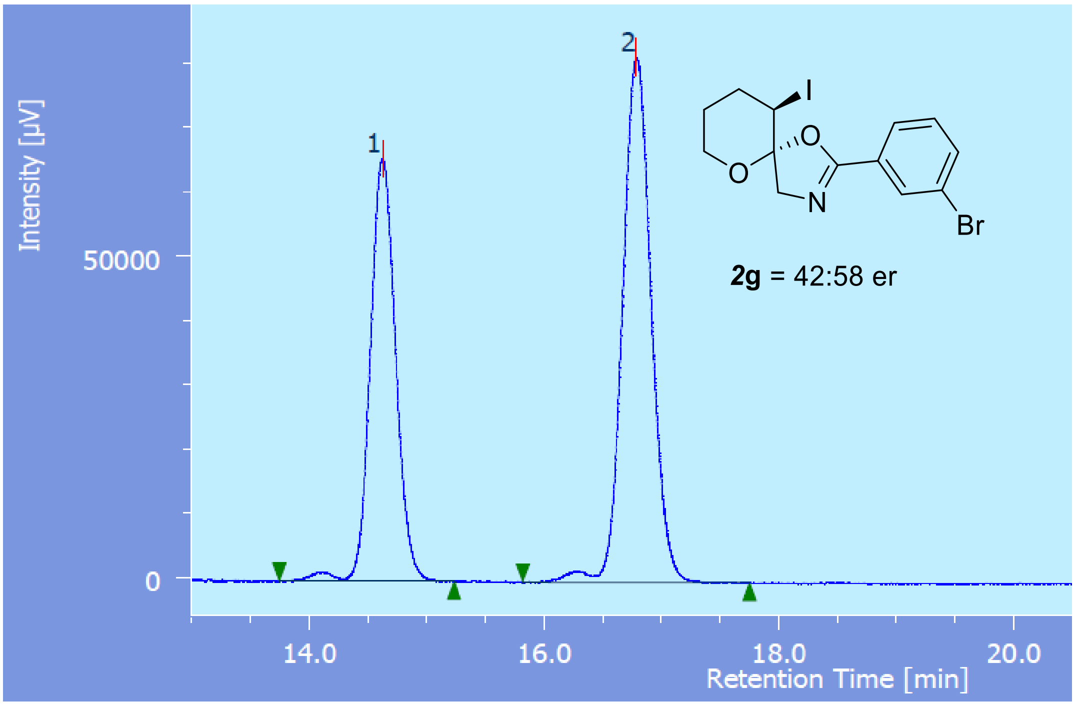

Channel Name 273.0nm

#	Peak Name	CH	tR [min]	Area [µV·sec]	Height [µV]	Area%	Height%	Quantity	NTP	Resolution	Symmetry Factor	Warning
1	Unknown	9	14.625	990725	65614	41.647	44.596	N/A	22520	5.240	1.091	
2	Unknown	9	16.787	1388149	81516	58.353	55.404	N/A	23565	N/A	1.086	

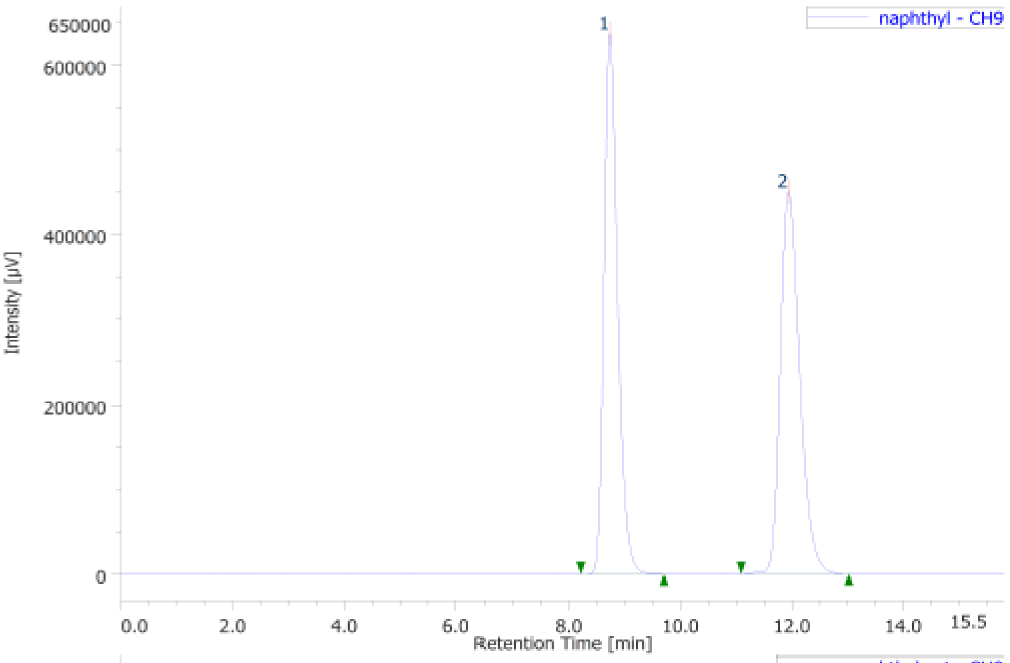

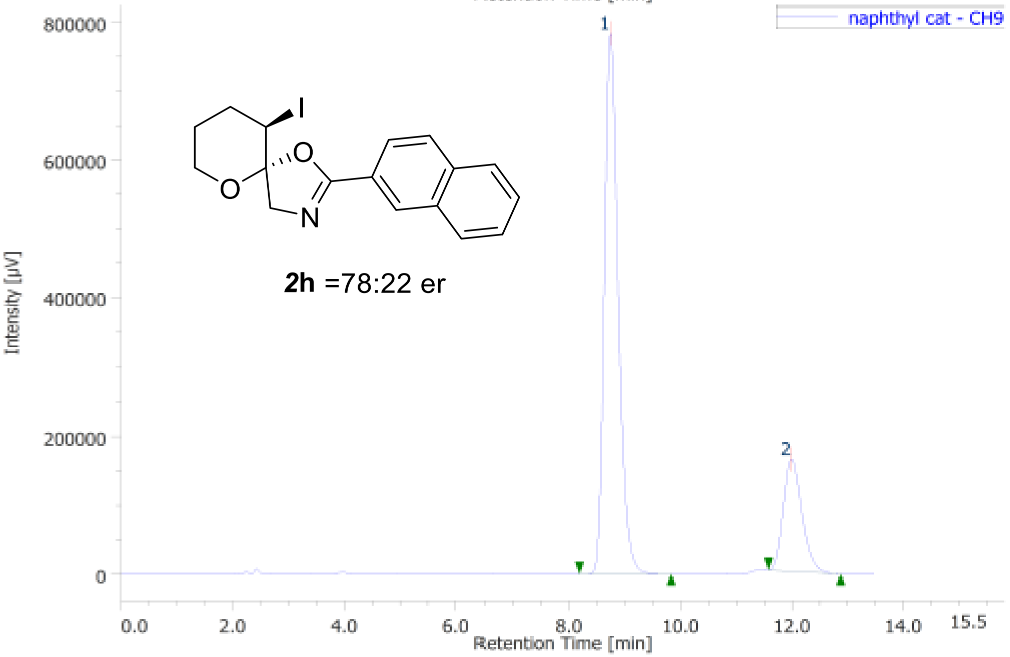

Channel & Peak Information Table

Chromatogram Name naphthyl−CH9
Sample Name
Channel Name 275.0nm

#	Peak Name	CH	tR [min]	Area [µV·sec]	Height [µV]	Area%	Height%	Quantity	NTP	Resolution	Symmetry Factor	Warning
1	Unknown	9	8.727	10854975	637230	49.902	58.597	N/A	6353	6.051	1.313	
2	Unknown	9	11.933	10897751	450249	50.098	41.403	N/A	5838	N/A	1.329	

Chromatogram Name naphthyl cat−CH9
Sample Name
Channel Name 255.0nm

#	Peak Name	CH	tR [min]	Area [µV·sec]	Height [µV]	Area%	Height%	Quantity	NTP	Resolution	Symmetry Factor	Warning
1	Unknown	9	8.737	13532939	783212	78.172	82.785	N/A	6182	6.208	1.290	
2	Unknown	9	11.990	3778702	162864	21.828	17.215	N/A	6252	N/A	1.244	

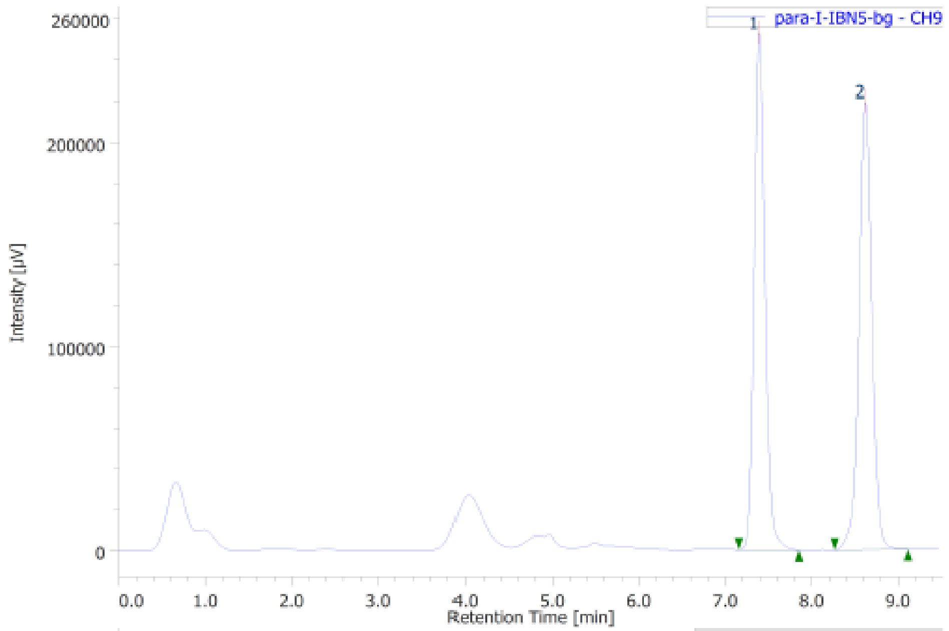

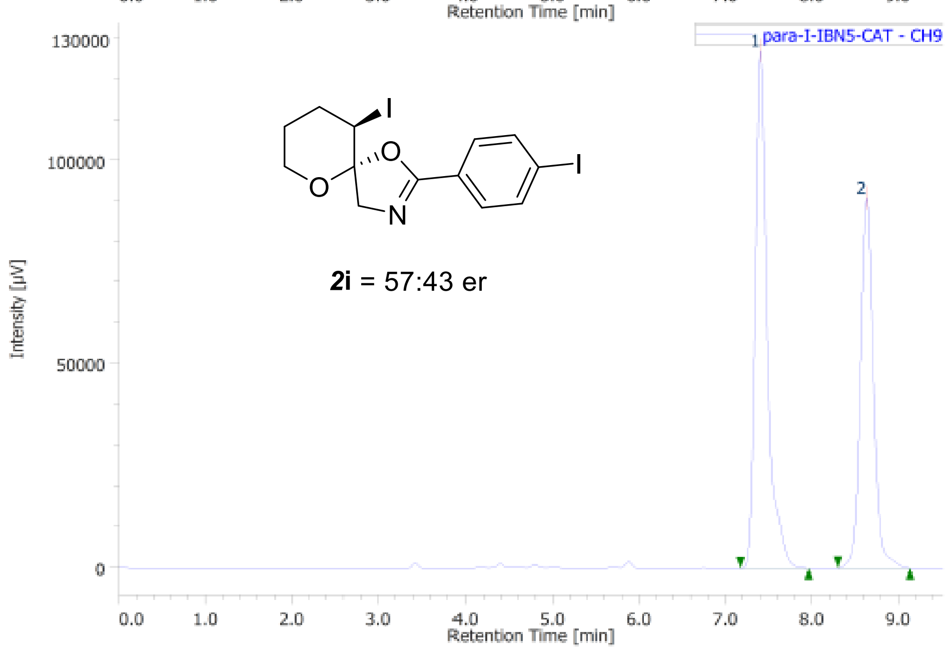

Channel & Peak Information Table

Chromatogram Name para-I-IBN5-bg-CH9
Sample Name
Channel Name 255.0nm

#	Peak Name	CH	tR [min]	Area [µV·sec]	Height [µV]	Area%	Height%	Quantity	NTP	Resolution	Symmetry Factor	Warning
1	Unknown	9	7.390	2225793	252965	49.512	53.571	N/A	17129	5.043	1.146	
2	Unknown	9	8.620	2269702	219241	50.488	46.429	N/A	17160	N/A	0.986	

Chromatogram Name para-I-IBN5-CAT-CH9
Sample Name
Channel Name 254.0nm

#	Peak Name	CH	tR [min]	Area [µV·sec]	Height [µV]	Area%	Height%	Quantity	NTP	Resolution	Symmetry Factor	Warning
1	Unknown	9	7.407	1242187	127431	56.515	58.372	N/A	16294	4.947	1.483	
2	Unknown	9	8.633	955806	90876	43.485	41.628	N/A	16964	N/A	1.093	

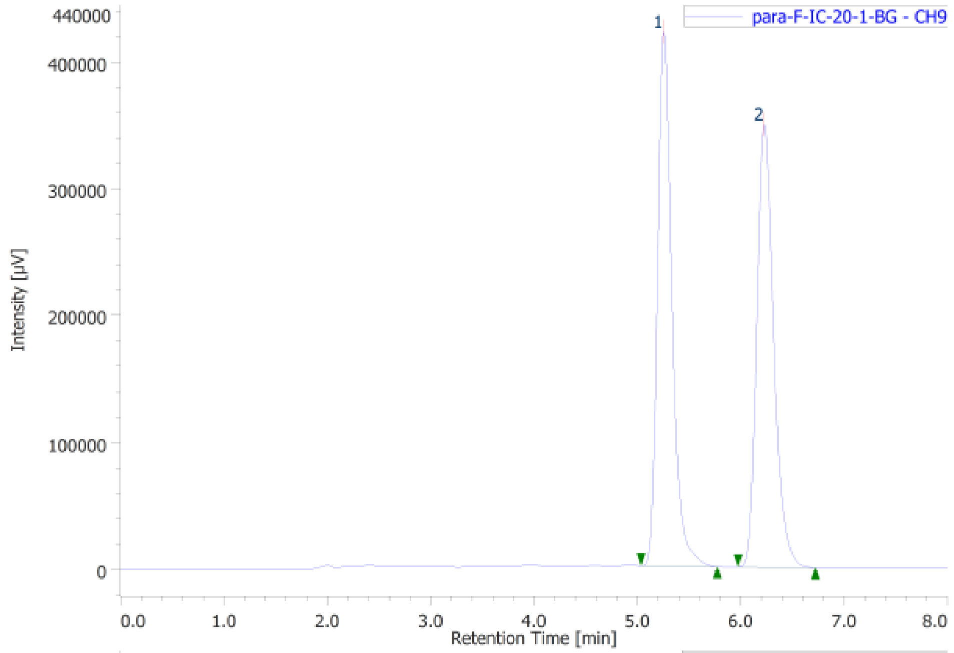

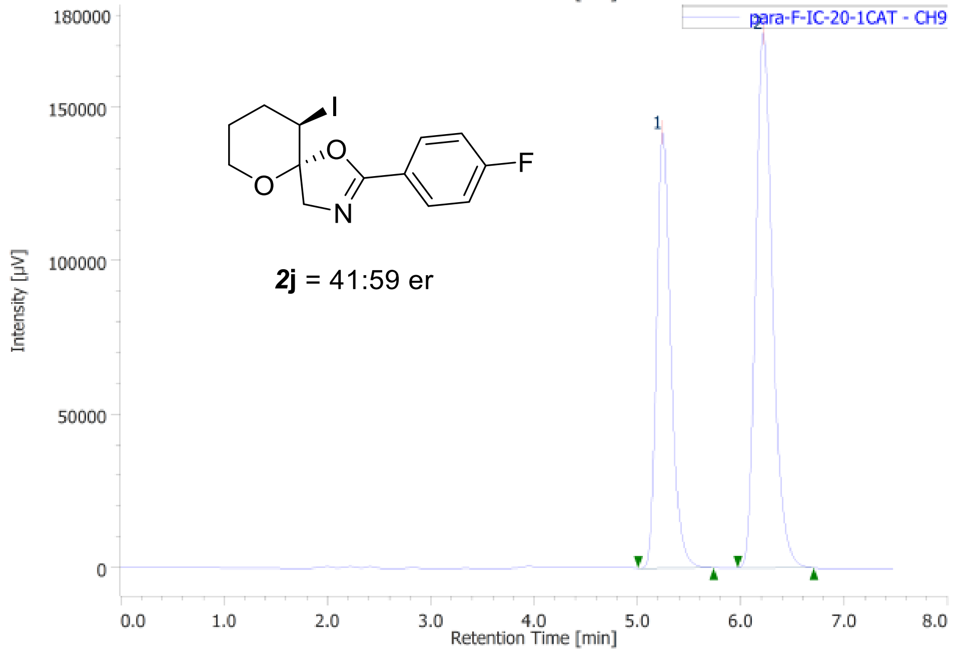

Channel & Peak Information Table

Chromatogram Name para-F-IC-20-1-BG-CH9
Sample Name
Channel Name 225.0nm

#	Peak Name	CH	tR [min]	Area [μV·sec]	Height [μV]	Area%	Height%	Quantity	NTP	Resolution	Symmetry Factor	Warning
1	Unknown	9	5.253	4063066	421097	50.653	54.701	N/A	7571	3.670	1.333	
2	Unknown	9	6.227	3958349	348723	49.347	45.299	N/A	7359	N/A	1.269	

Chromatogram Name para-F-IC-20-1CAT-CH9
Sample Name
Channel Name 225.0nm

#	Peak Name	CH	tR [min]	Area [μV·sec]	Height [μV]	Area%	Height%	Quantity	NTP	Resolution	Symmetry Factor	Warning
1	Unknown	9	5.247	1354156	141856	40.591	44.860	N/A	7506	3.650	1.282	
2	Unknown	9	6.217	1981912	174362	59.409	55.140	N/A	7319	N/A	1.255	

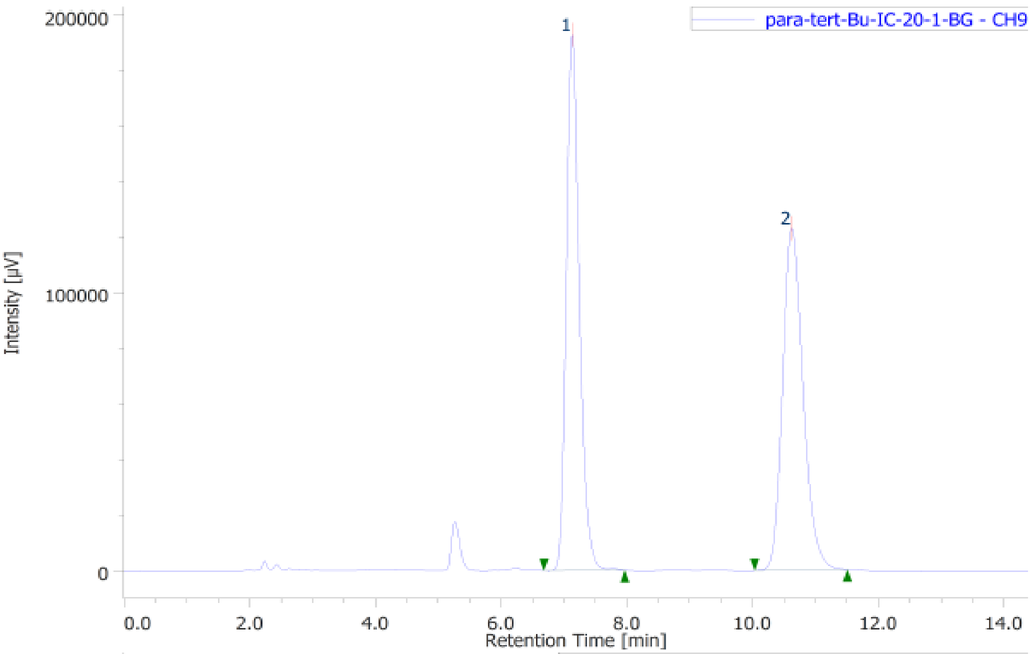

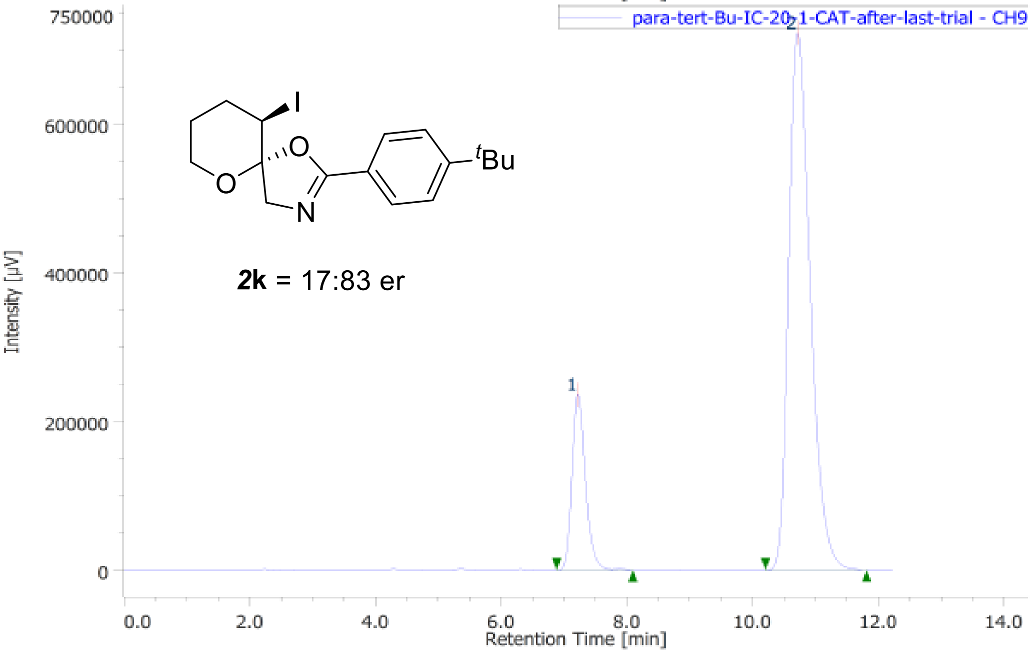

Channel & Peak Information Table

Chromatogram Name para−tert−Bu−IC−20−1−BG−CH9
Sample Name
Channel Name 260.0nm

#	Peak Name	CH	tR [min]	Area [µV·sec]	Height [µV]	Area%	Height%	Quantity	NTP	Resolution	Symmetry Factor	Warning
1	Unknown	9	7.130	2792994	192646	50.128	61.050	N/A	5899	7.330	1.248	
2	Unknown	9	10.623	2778776	122907	49.872	38.950	N/A	5288	N/A	1.229	

Chromatogram Name para−tert−Bu−IC−20−1−CAT−after−last−trial−CH9
Sample Name
Channel Name 260.0nm

#	Peak Name	CH	tR [min]	Area [µV·sec]	Height [µV]	Area%	Height%	Quantity	NTP	Resolution	Symmetry Factor	Warning
1	Unknown	9	7.223	3503987	235787	16.927	24.626	N/A	5820	7.009	1.254	
2	Unknown	9	10.717	17196475	721689	83.073	75.374	N/A	4770	N/A	1.359	

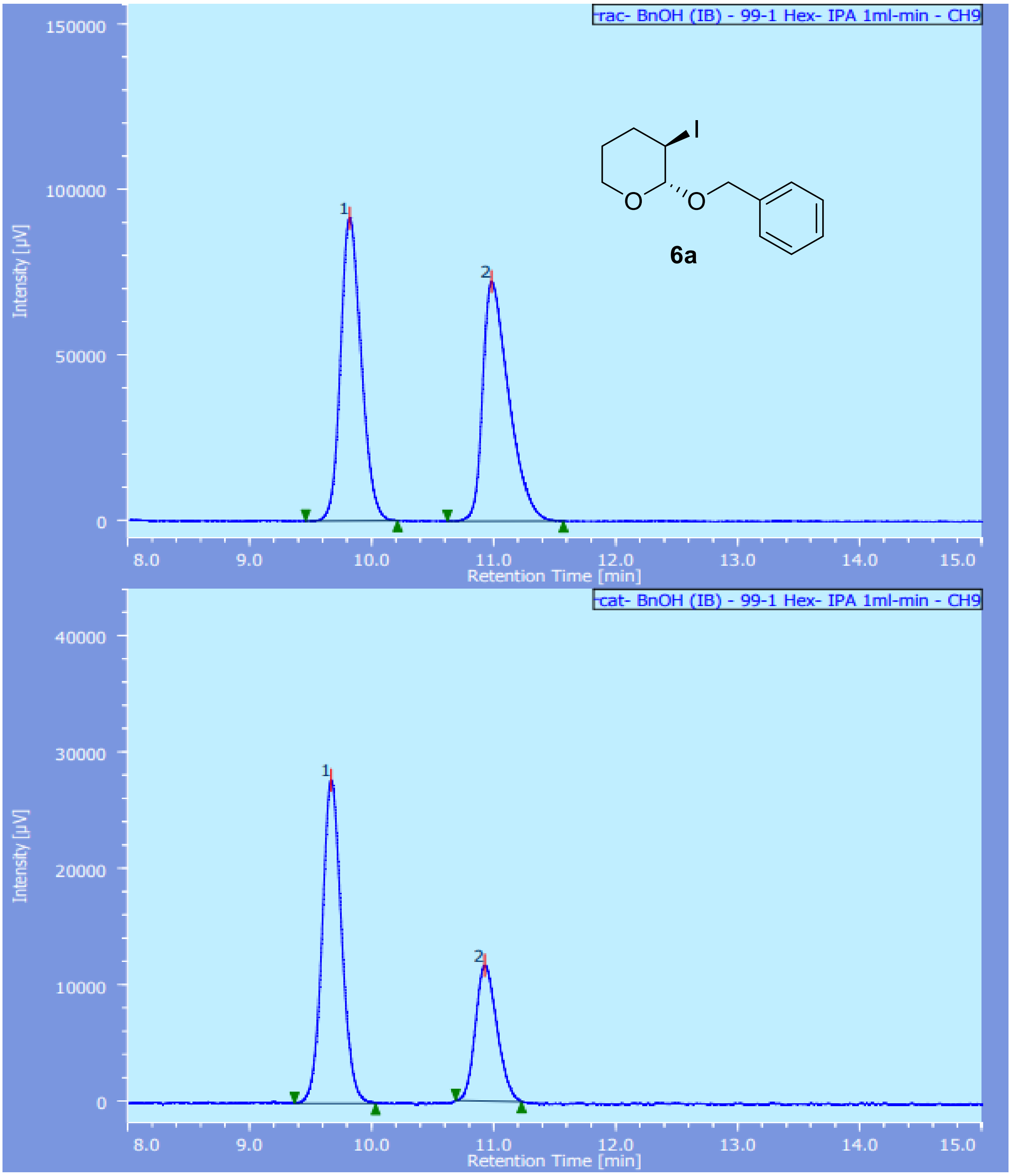

Channel & Peak Information Table

Chromatogram Name rac– BnOH (IB) – 99–1 Hex– IPA 1ml–min–CH9

Sample Name

Channel Name 275.0nm

#	Peak Name	CH	tR [min]	Area [µV·sec]	Height [µV]	Area%	Height%	Quantity	NTP	Resolution	Symmetry Factor	Warning
1	Unknown	9	9.820	1066935	91455	49.767	55.806	N/A	16815	3.394	1.185	
2	Unknown	9	10.982	1076944	72424	50.233	44.194	N/A	13121	N/A	1.628	

Chromatogram Name cat– BnOH (IB) – 99–1 Hex– IPA 1ml–min–CH9

Sample Name

Channel Name 275.0nm

#	Peak Name	CH	tR [min]	Area [µV·sec]	Height [µV]	Area%	Height%	Quantity	NTP	Resolution	Symmetry Factor	Warning
1	Unknown	9	9.670	310094	27772	68.509	70.466	N/A	18057	4.121	1.101	
2	Unknown	9	10.927	142538	11640	31.491	29.534	N/A	18235	N/A	1.197	

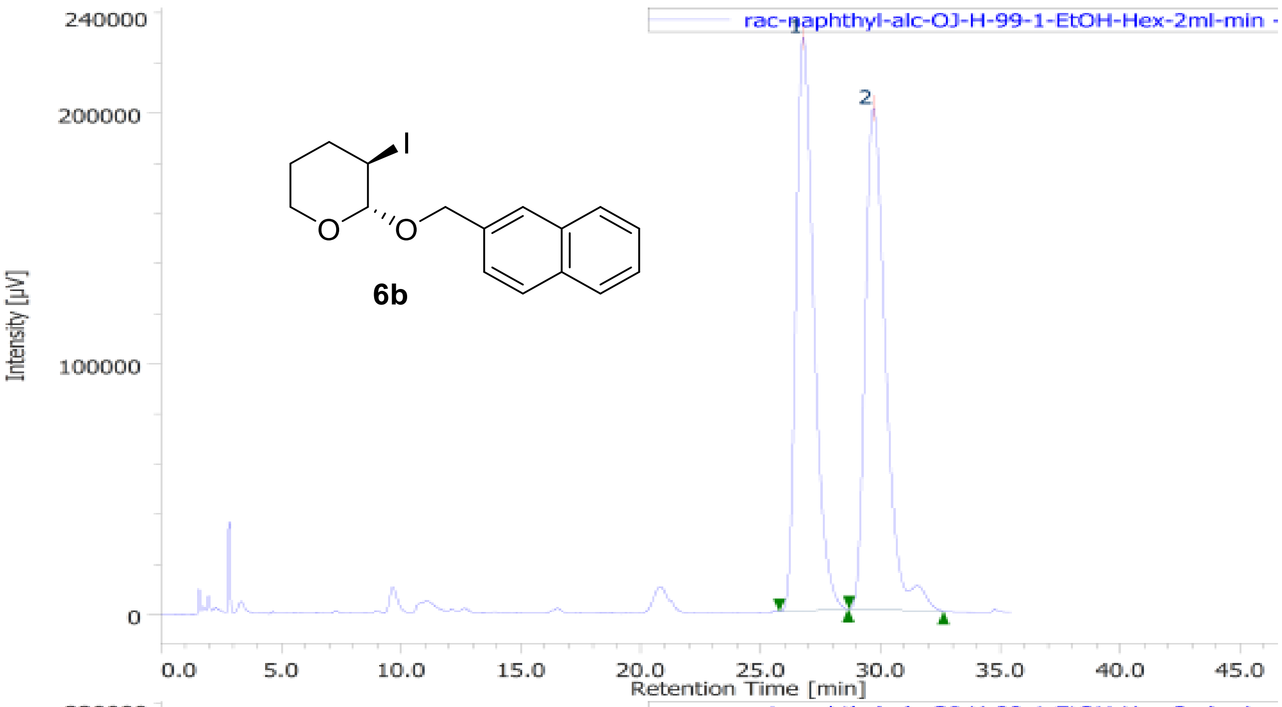

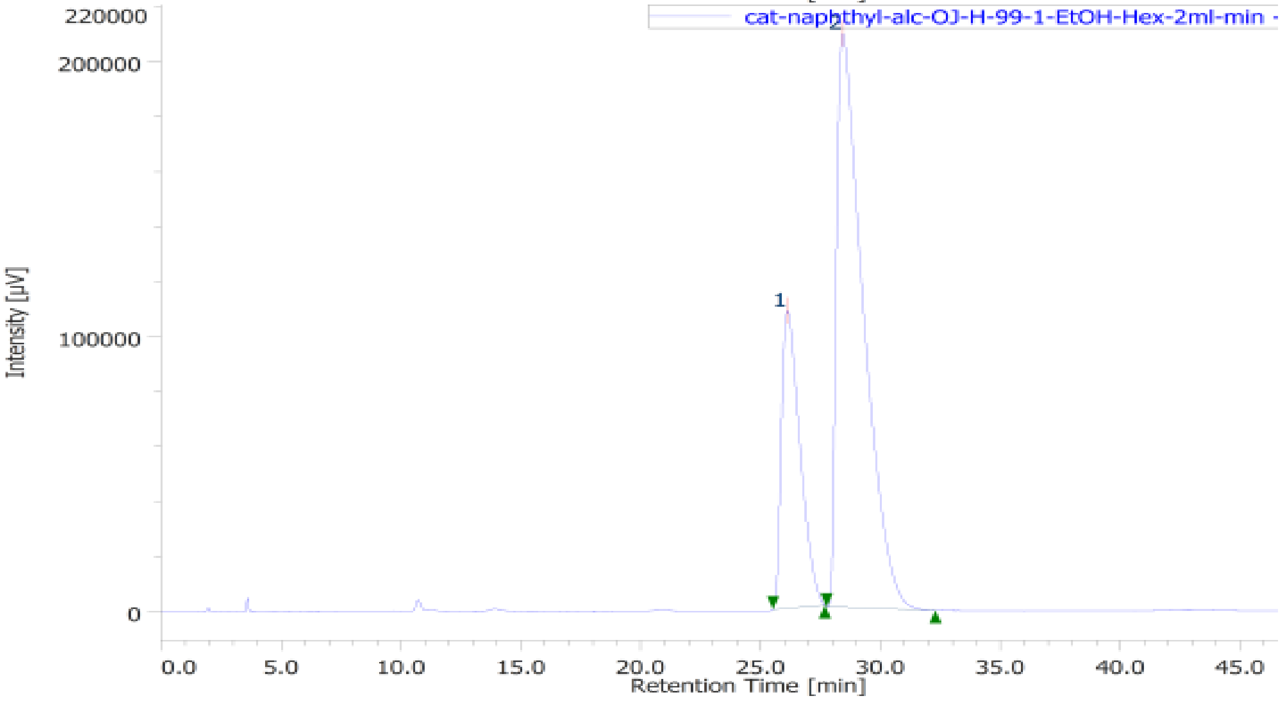

Channel & Peak Information Table

Chromatogram Name	rac−naphthyl−alc−OJ−H−99−1−EtOH−Hex−2ml−min−CH9
Sample Name	
Channel Name	220.0nm

#	Peak Name	CH	tR [min]	Area [µV·sec]	Height [µV]	Area%	Height%	Quantity	NTP	Resolution	Symmetry Factor	Warning
1	Unknown	9	26.773	11890807	228878	49.933	53.376	N/A	6367	2.058	1.402	
2	Unknown	9	29.697	11922523	199927	50.067	46.624	N/A	6216	N/A	1.385	

Chromatogram Name	cat−naphthyl−alc−OJ−H−99−1−EtOH−Hex−2ml−min−CH9
Sample Name	
Channel Name	275.0nm

#	Peak Name	CH	tR [min]	Area [µV·sec]	Height [µV]	Area%	Height%	Quantity	NTP	Resolution	Symmetry Factor	Warning
1	Unknown	9	26.113	5827902	108083	26.452	34.133	N/A	5209	1.332	1.821	
2	Unknown	9	28.430	16204183	208571	73.548	65.867	N/A	3106	N/A	2.696	

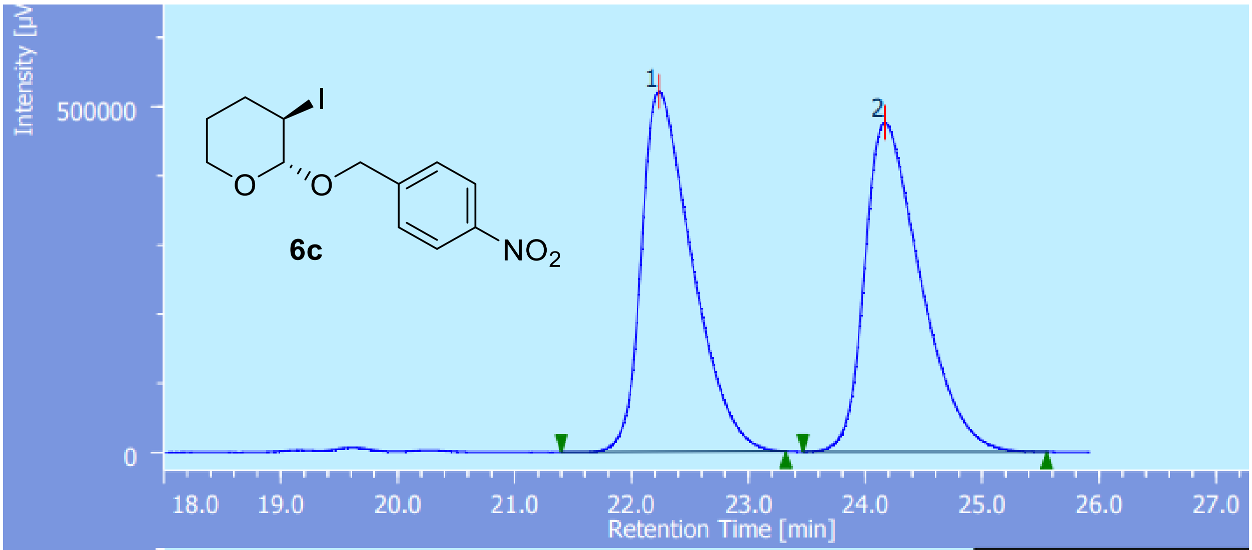

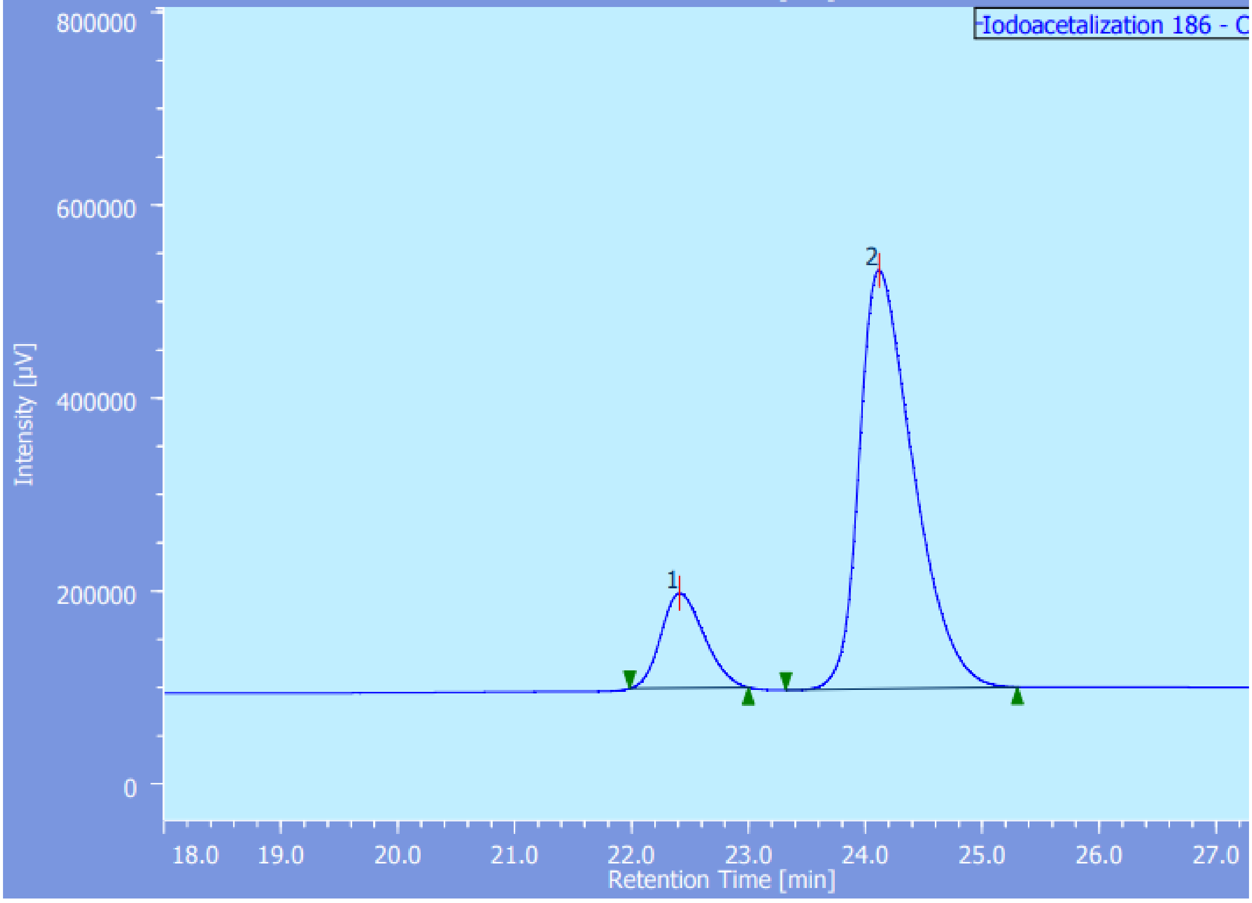

Channel & Peak Information Table

Chromatogram Name Iodoacetalization 186-CH1

Sample Name

Channel Name UV-2075

#	Peak Name	CH	tR [min]	Area [μV·sec]	Height [μV]	Area%	Height%	Quantity	NTP	Resolution	Symmetry Factor	Warning
1	Unknown	1	22.233	15807822	520819	49.912	52.256	N/A	12777	2.353	1.558	
2	Unknown	1	24.167	15863395	475854	50.088	47.744	N/A	12611	N/A	1.516	

Chromatogram Name Iodoacetalization 186-CH1

Sample Name

Channel Name UV-2075

#	Peak Name	CH	tR [min]	Area [μV·sec]	Height [μV]	Area%	Height%	Quantity	NTP	Resolution	Symmetry Factor	Warning
1	Unknown	1	22.408	2524003	98228	15.320	18.468	N/A	17091	2.257	1.209	
2	Unknown	1	24.117	13950864	433643	84.680	81.532	N/A	13429	N/A	1.486	

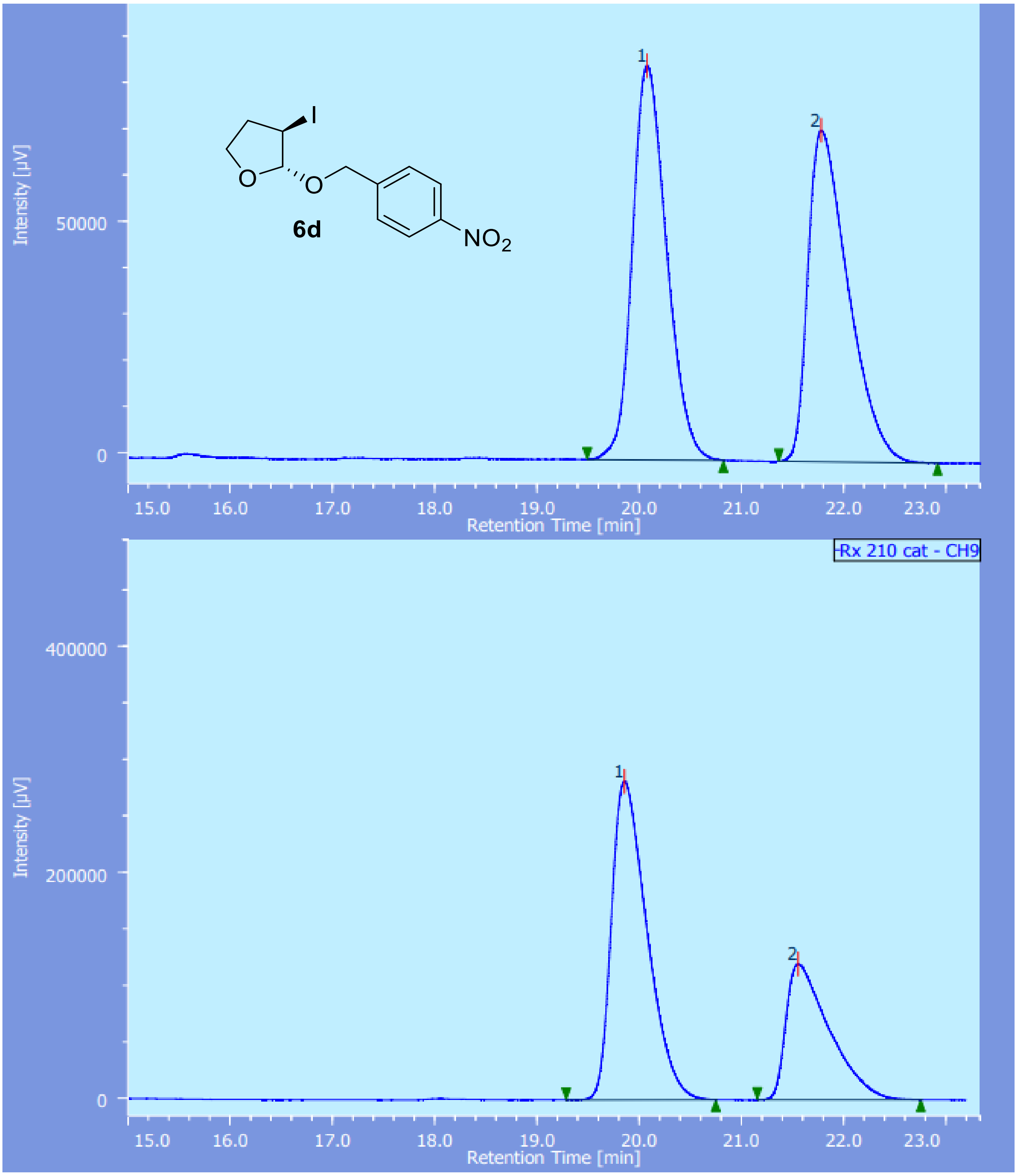

Channel & Peak Information Table

Chromatogram Name Rx 211 BG-CH9

Sample Name

Channel Name 230.0nm

#	Peak Name	CH	tR [min]	Area [μV·sec]	Height [μV]	Area%	Height%	Quantity	NTP	Resolution	Symmetry Factor	Warning
1	Unknown	9	20.075	1984765	85176	50.443	54.319	N/A	17469	2.584	1.217	
2	Unknown	9	21.777	1949919	71632	49.557	45.681	N/A	14914	N/A	1.746	

Chromatogram Name Rx 210 cat-CH9

Sample Name

Channel Name 230.0nm

#	Peak Name	CH	tR [min]	Area [μV·sec]	Height [μV]	Area%	Height%	Quantity	NTP	Resolution	Symmetry Factor	Warning
1	Unknown	9	19.857	6804790	281657	65.870	70.108	N/A	15610	2.438	1.522	
2	Unknown	9	21.553	3525774	120092	34.130	29.892	N/A	12871	N/A	2.098	

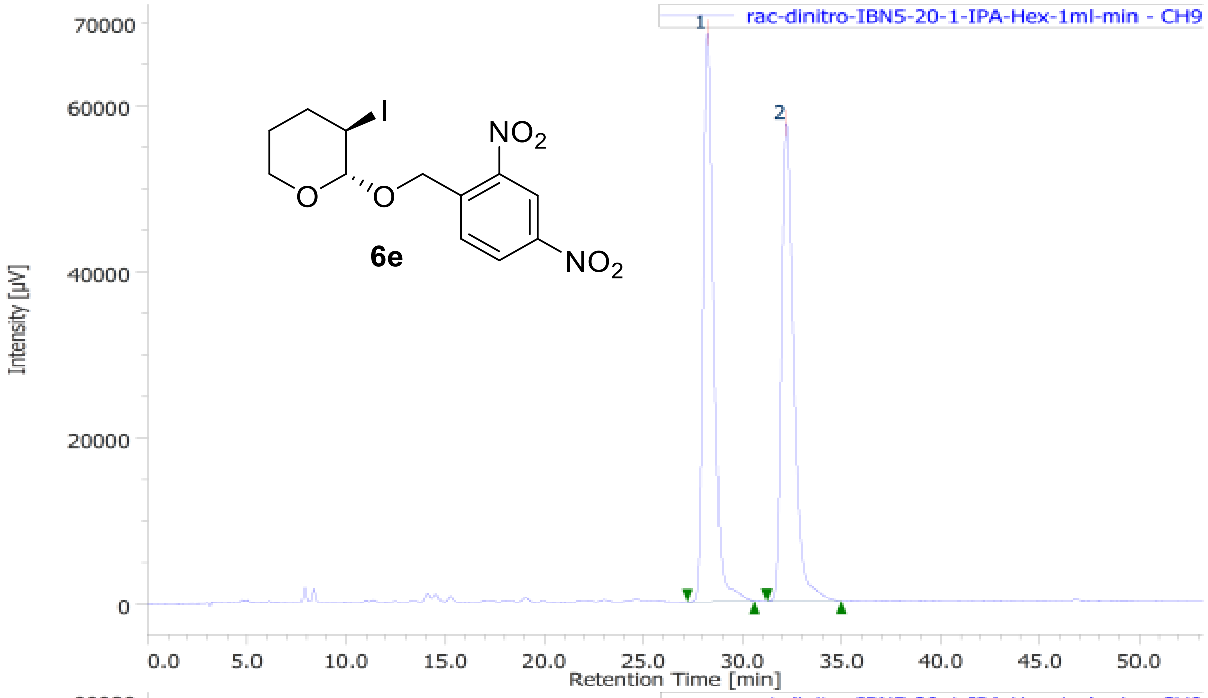

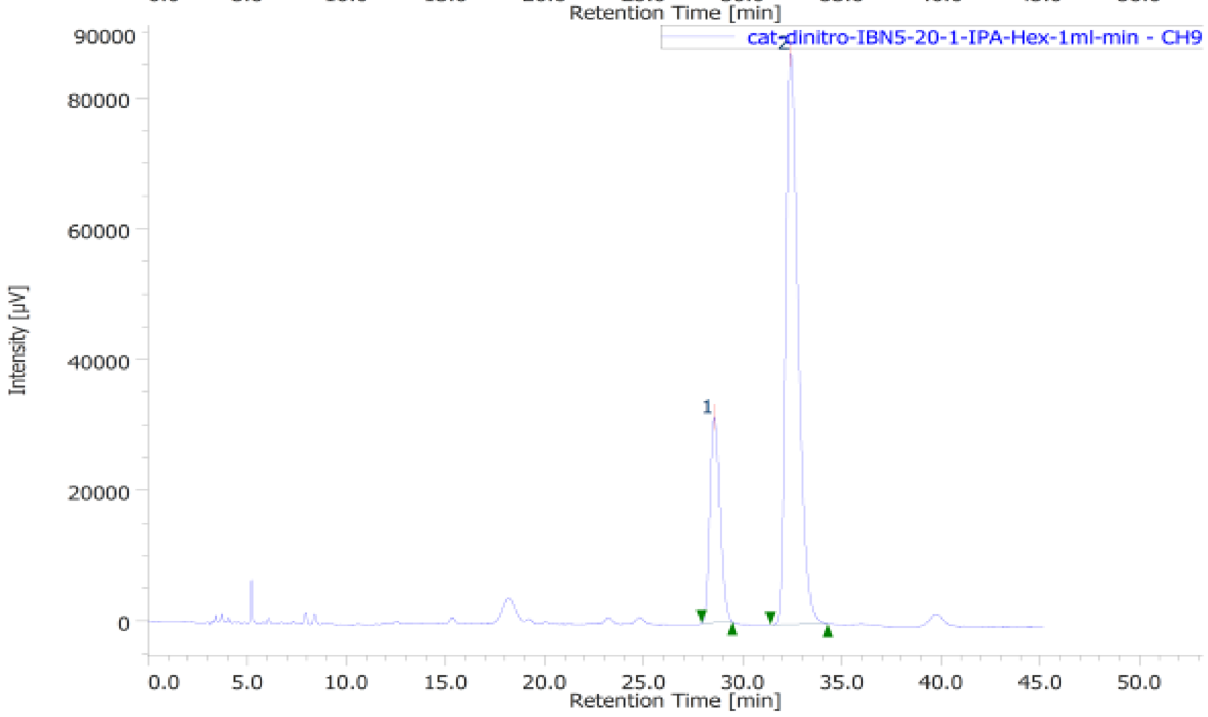

Channel & Peak Information Table

Chromatogram Name rac−dinitro−IBN5−20−1−IPA−Hex−1ml−min−CH9
Sample Name
Channel Name 254.0nm

#	Peak Name	CH	tR [min]	Area [µV·sec]	Height [µV]	Area%	Height%	Quantity	NTP	Resolution	Symmetry Factor	Warning
1	Unknown	9	28.227	2532784	68463	50.057	54.357	N/A	14626	3.907	1.348	
2	Unknown	9	32.197	2527040	57488	49.943	45.643	N/A	13602	N/A	1.453	

Chromatogram Name cat−dinitro−IBN5−20−1−IPA−Hex−1ml−min−CH9
Sample Name
Channel Name 254.0nm

#	Peak Name	CH	tR [min]	Area [µV·sec]	Height [µV]	Area%	Height%	Quantity	NTP	Resolution	Symmetry Factor	Warning
1	Unknown	9	28.550	1091288	31403	22.335	26.467	N/A	15404	3.776	1.205	
2	Unknown	9	32.407	3794766	87247	77.665	73.533	N/A	13207	N/A	1.445	

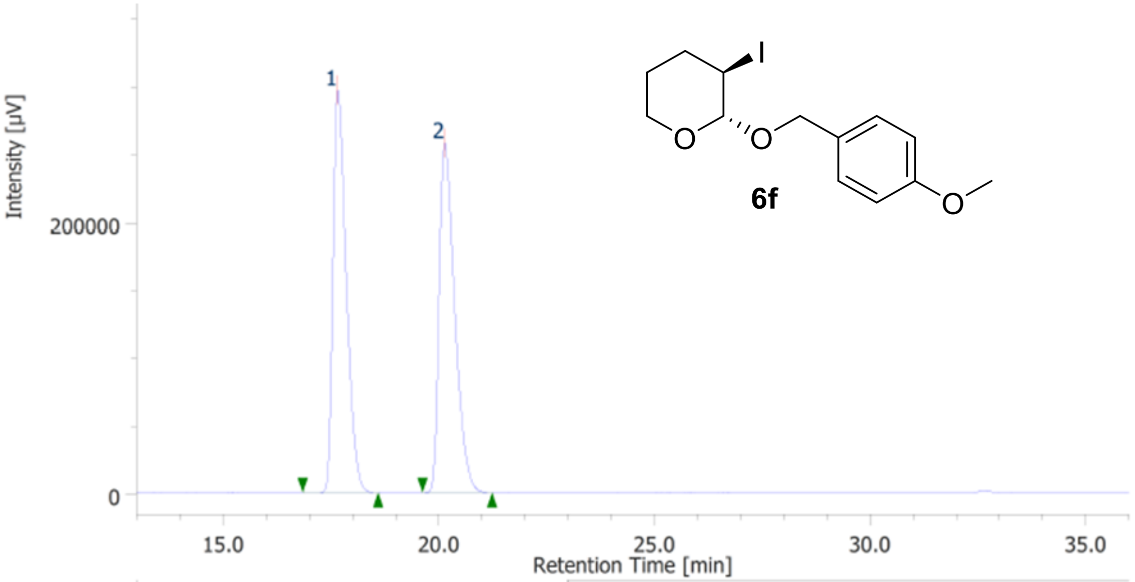

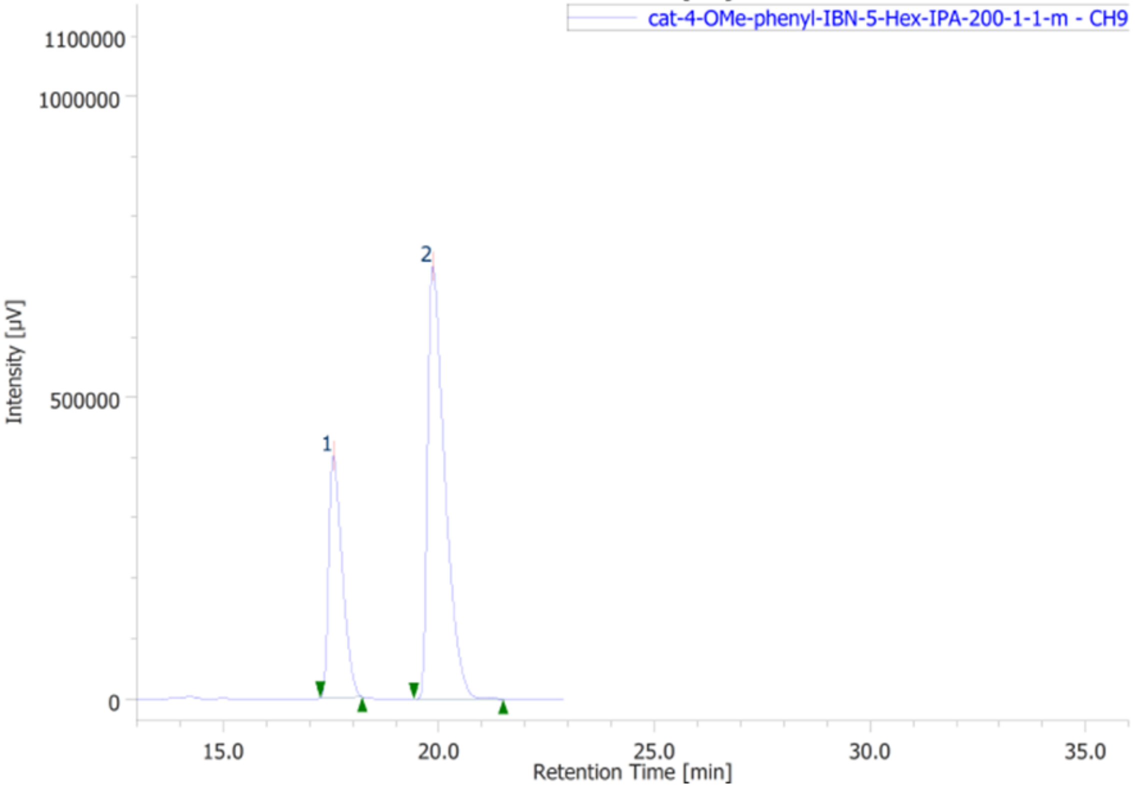

Channel & Peak Information Table

Chromatogram Name rac−4−OMe−phenyl−IBN−5−Hex−IPA−200−1−1−m−CH9

Sample Name

Channel Name 235.0nm

#	Peak Name	CH	tR [min]	Area [µV·sec]	Height [µV]	Area%	Height%	Quantity	NTP	Resolution	Symmetry Factor	Warning
1	Unknown	9	17.657	6344645	296795	49.515	53.507	N/A	15902	4.096	1.519	
2	Unknown	9	20.140	6468855	257889	50.485	46.493	N/A	15099	N/A	1.566	

Chromatogram Name cat−4−OMe−phenyl−IBN−5−Hex−IPA−200−1−1−m−CH9

Sample Name

Channel Name 235.0nm

#	Peak Name	CH	tR [min]	Area [µV·sec]	Height [µV]	Area%	Height%	Quantity	NTP	Resolution	Symmetry Factor	Warning
1	Unknown	9	17.553	8627755	400020	30.418	35.806	N/A	15155	3.607	1.630	
2	Unknown	9	19.860	19736683	717179	69.582	64.194	N/A	12443	N/A	2.019	

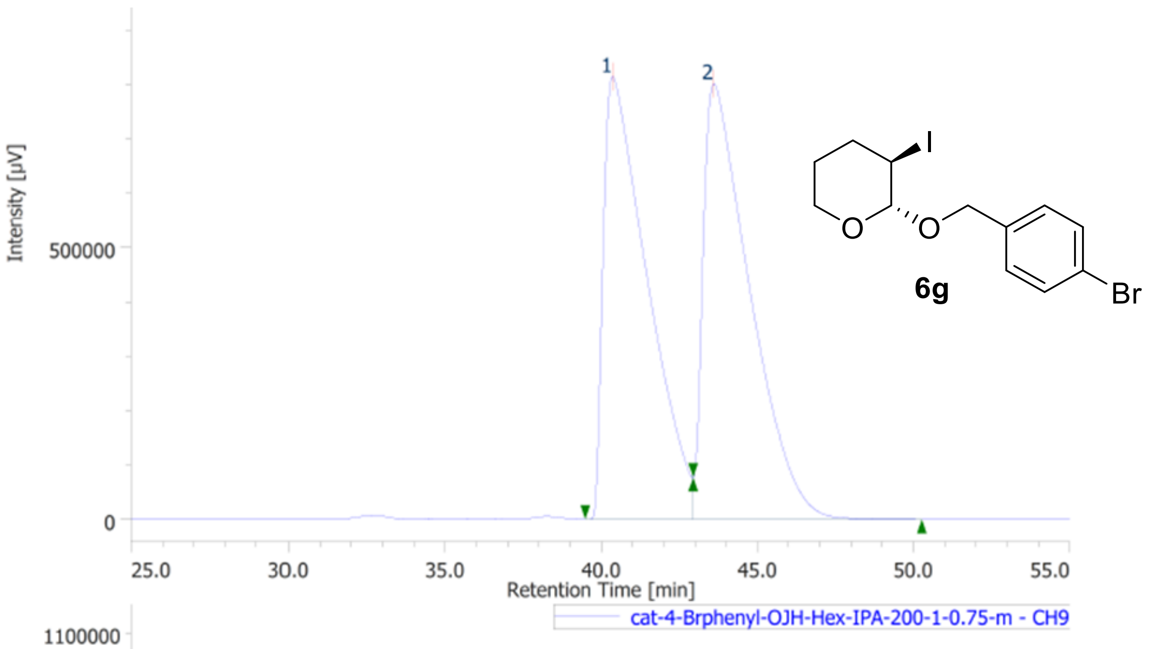

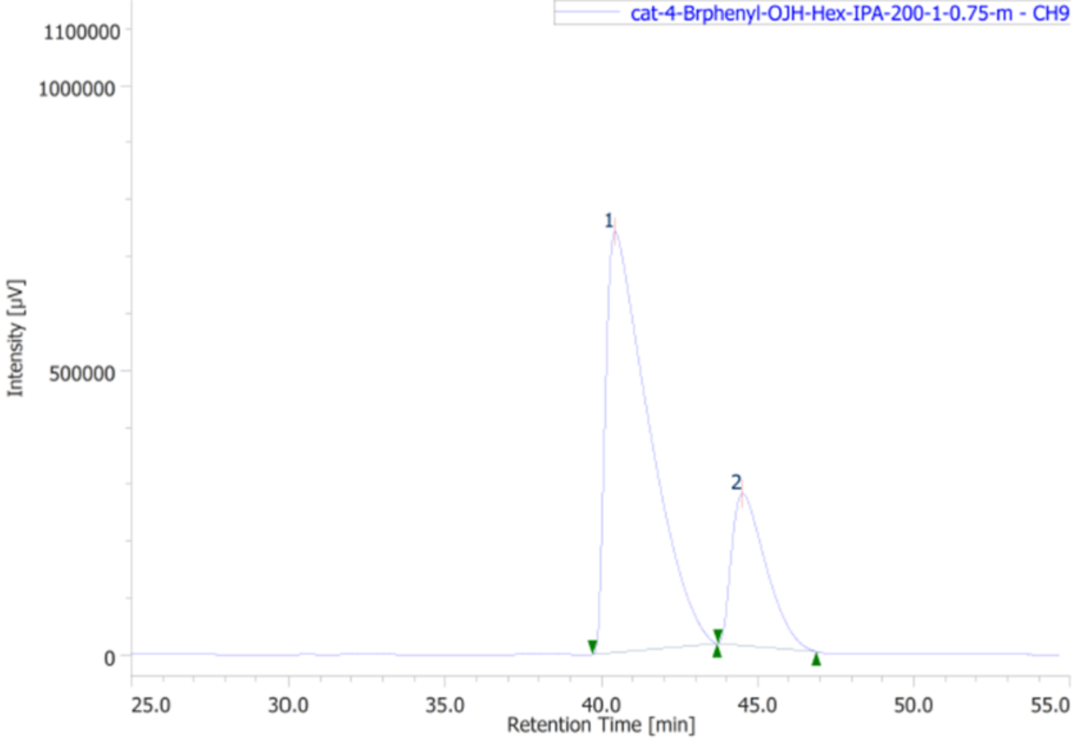

Channel & Peak Information Table

Chromatogram Name rac−4−Brphenyl−OJH−Hex−IPA−200−1−0.75−m−CH9
Sample Name
Channel Name 225.0nm

#	Peak Name	CH	tR [min]	Area [µV·sec]	Height [µV]	Area%	Height%	Quantity	NTP	Resolution	Symmetry Factor	Warning
1	Unknown	9	40.363	79630400	813480	48.892	50.395	N/A	3718	1.208	N/A	
2	Unknown	9	43.603	83240770	800733	51.108	49.605	N/A	4075	N/A	N/A	

Chromatogram Name cat−4−Brphenyl−OJH−Hex−IPA−200−1−0.75−m−CH9
Sample Name
Channel Name 225.0nm

#	Peak Name	CH	tR [min]	Area [µV·sec]	Height [µV]	Area%	Height%	Quantity	NTP	Resolution	Symmetry Factor	Warning
1	Unknown	9	40.430	71611228	739049	77.834	73.496	N/A	3932	1.770	2.956	
2	Unknown	9	44.513	20393532	266513	22.166	26.504	N/A	7556	N/A	1.893	

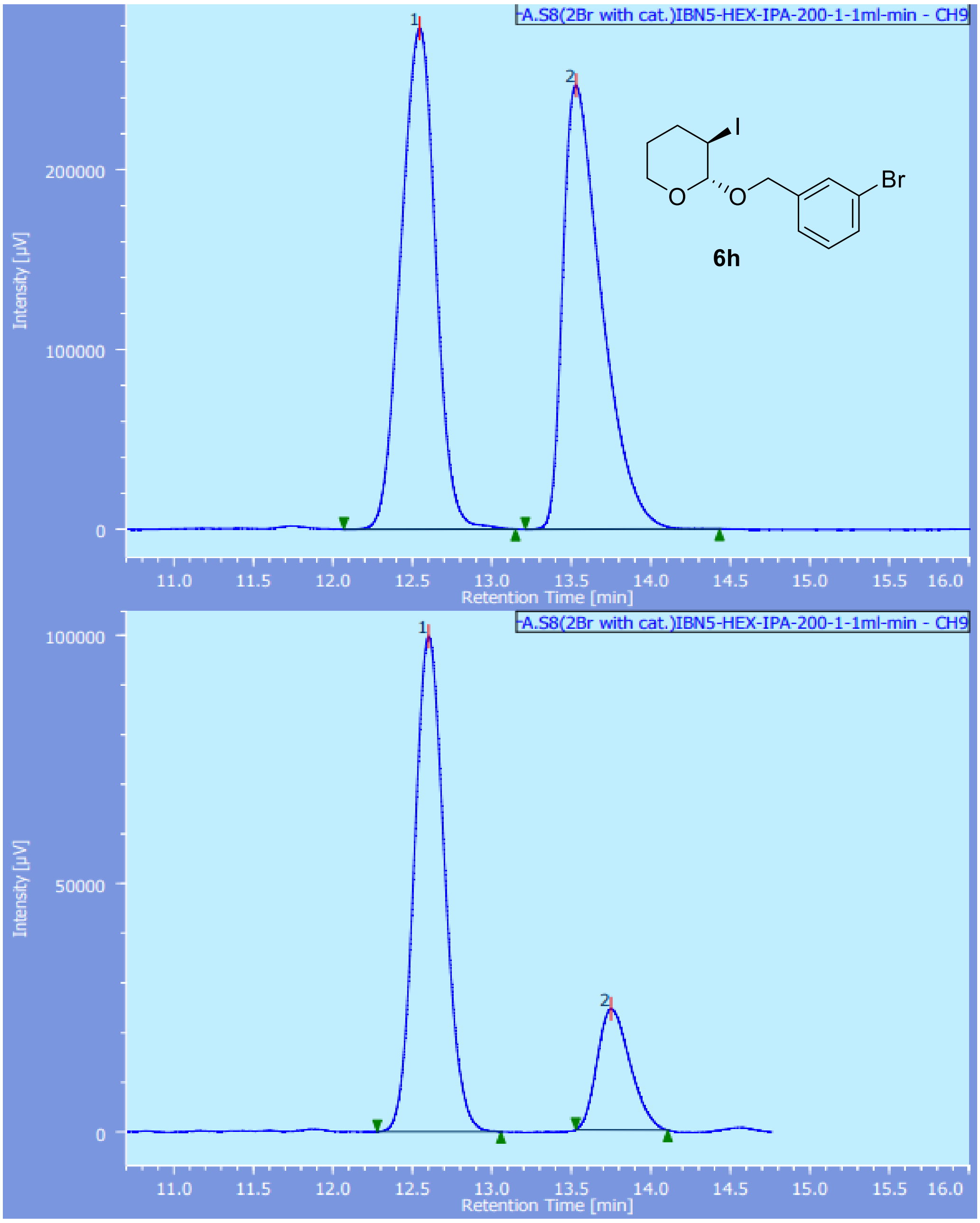

Channel & Peak Information Table

Chromatogram Name A.S8(2Br with cat.)IBN5−HEX−IPA−200−1−1ml−min−CH9

Sample Name

Channel Name 250.0nm

#	Peak Name	CH	tR [min]	Area [µV·sec]	Height [µV]	Area%	Height%	Quantity	NTP	Resolution	Symmetry Factor	Warning
1	Unknown	9	12.547	4217878	278495	50.302	53.020	N/A	15469	2.342	0.977	
2	Unknown	9	13.528	4167169	246769	49.698	46.980	N/A	15324	N/A	1.793	

Chromatogram Name A.S8(2Br with cat.)IBN5−HEX−IPA−200−1−1ml−min−CH9

Sample Name

Channel Name 250.0nm

#	Peak Name	CH	tR [min]	Area [µV·sec]	Height [µV]	Area%	Height%	Quantity	NTP	Resolution	Symmetry Factor	Warning
1	Unknown	9	12.602	1313145	99670	78.915	80.378	N/A	21091	3.144	1.113	
2	Unknown	9	13.748	350859	24332	21.085	19.622	N/A	20470	N/A	1.262	

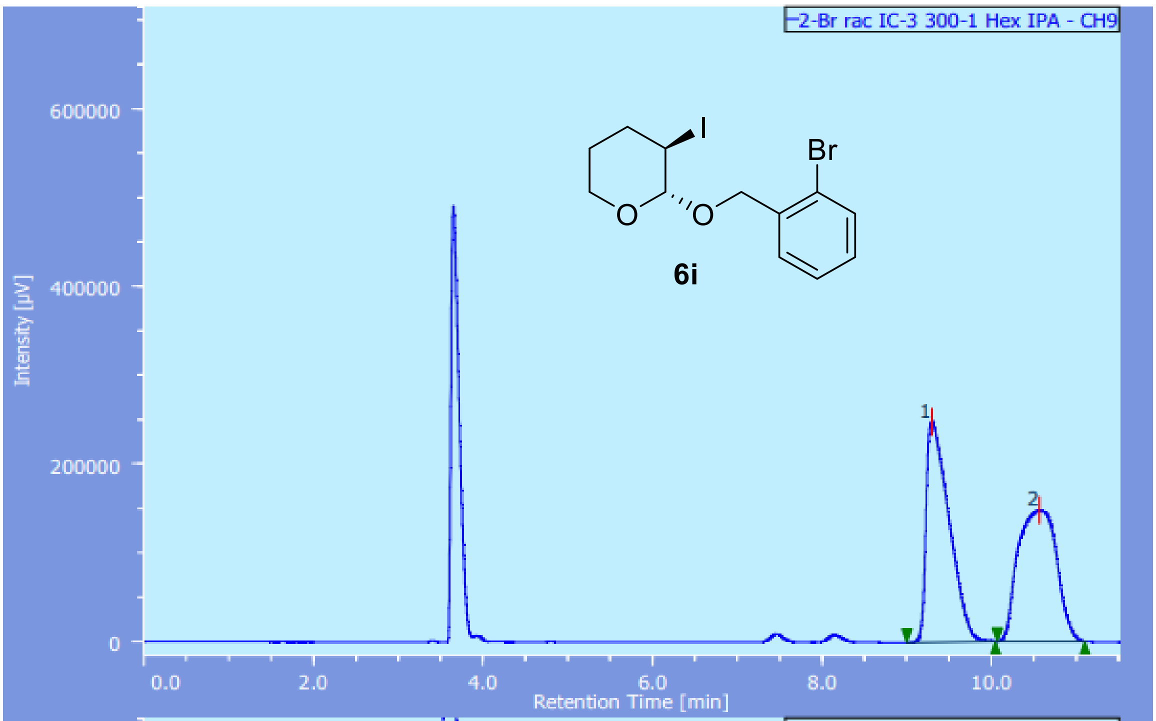

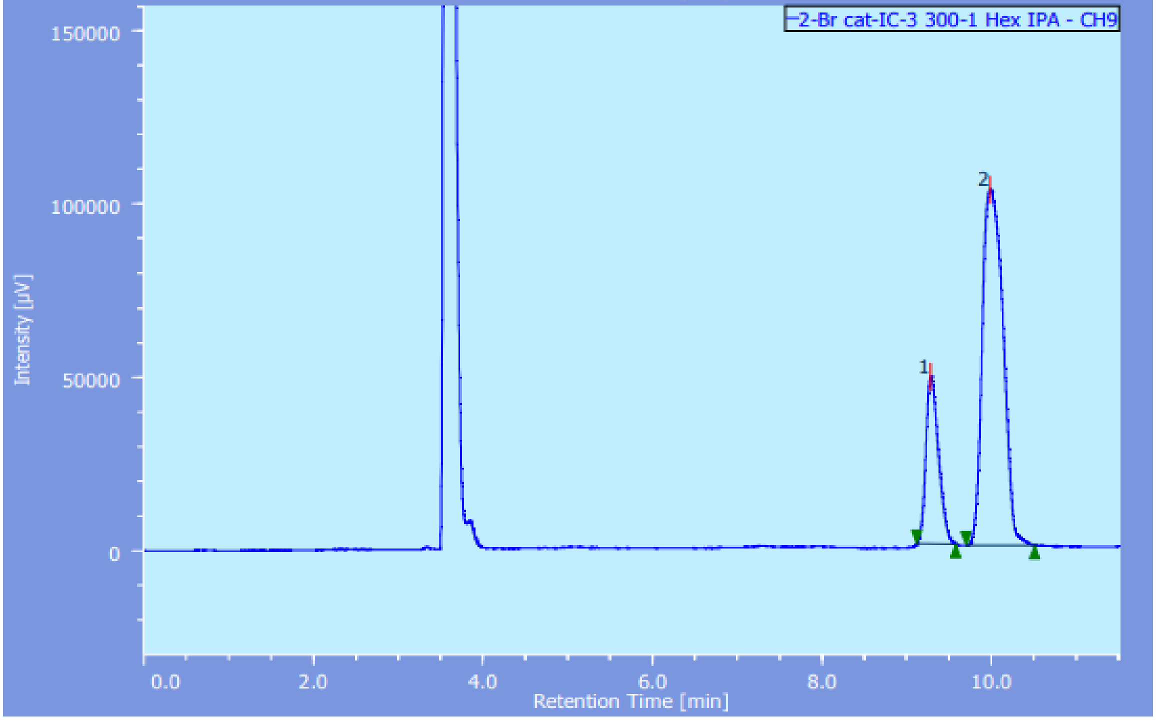

Channel & Peak Information Table

Chromatogram Name 2-Br rac IC-3 300-1 Hex IPA-CH9

Sample Name

Channel Name 230.0nm

#	Peak Name	CH	tR [min]	Area [µV·sec]	Height [µV]	Area%	Height%	Quantity	NTP	Resolution	Symmetry Factor	Warning
1	Unknown	9	9.292	4655584	247891	49.871	62.689	N/A	5289	1.793	2.207	
2	Unknown	9	10.565	4679625	147538	50.129	37.311	N/A	2142	N/A	0.989	

Chromatogram Name 2-Br cat-IC-3 300-1 Hex IPA-CH9

Sample Name

Channel Name 230.0nm

#	Peak Name	CH	tR [min]	Area [µV·sec]	Height [µV]	Area%	Height%	Quantity	NTP	Resolution	Symmetry Factor	Warning
1	Unknown	9	9.282	502698	48091	22.425	31.973	N/A	18039	1.869	1.318	
2	Unknown	9	9.982	1738999	102319	77.575	68.027	N/A	7080	N/A	1.255	

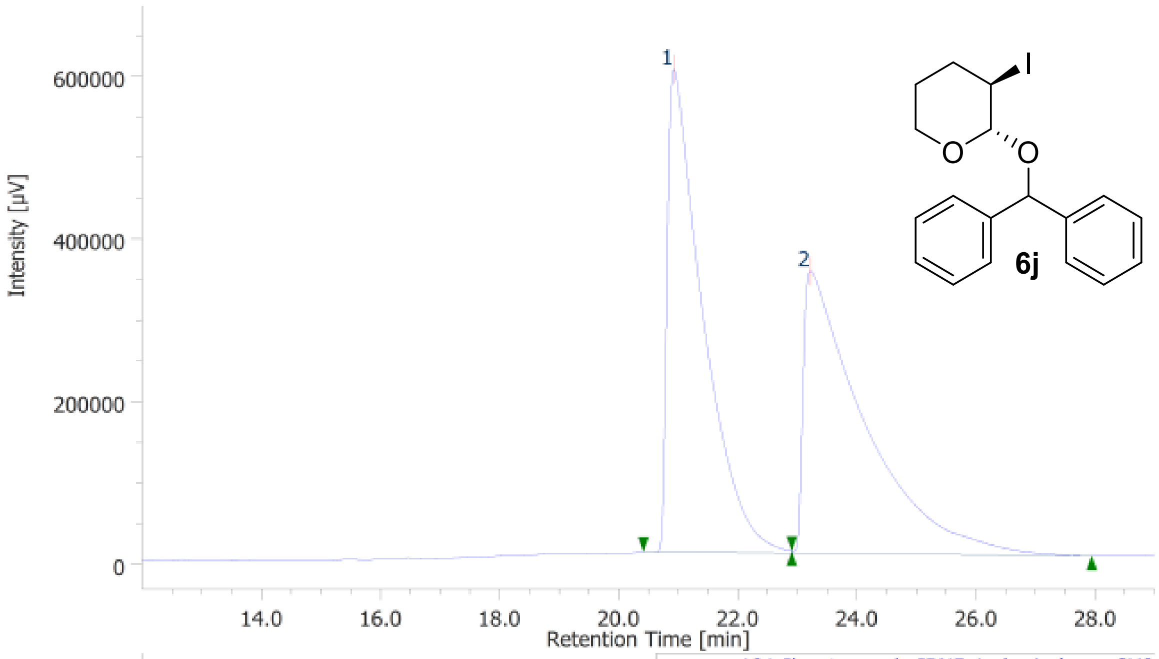

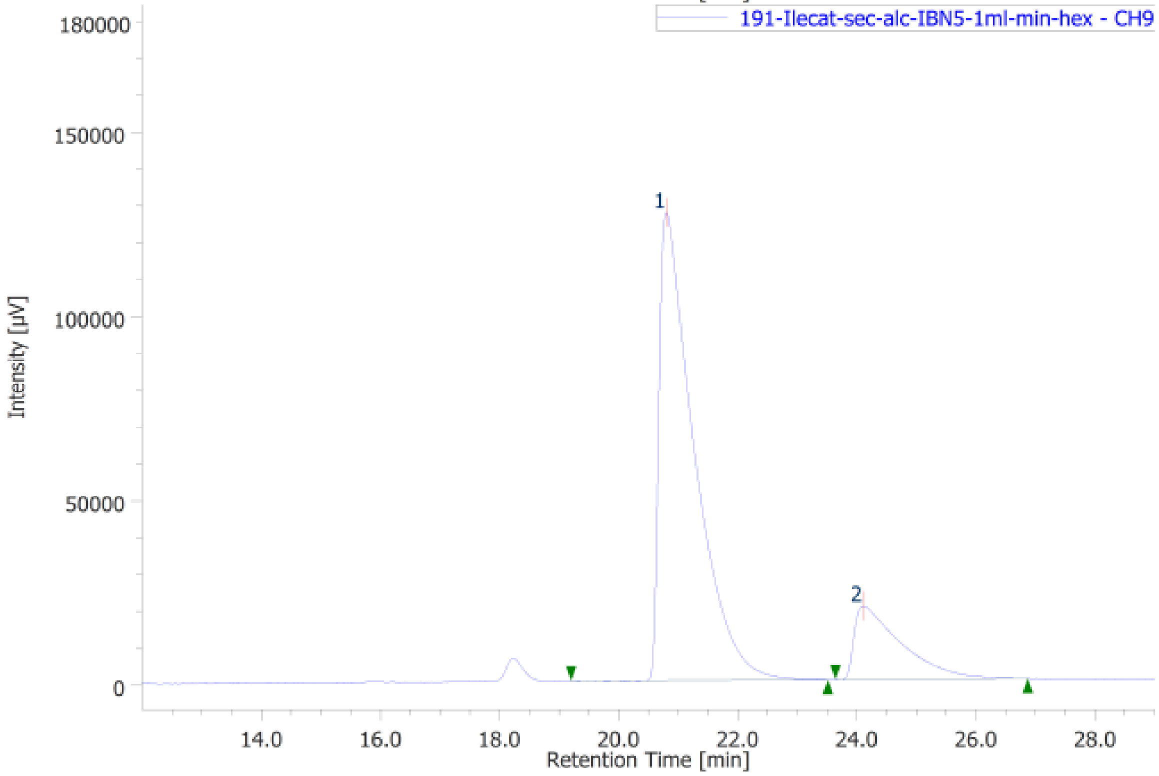

Channel & Peak Information Table

Chromatogram Name 170racemic-sec-alc-IBN5-1ml-min-hex-CH9
Sample Name
Channel Name 210.0nm

#	Peak Name	CH	tR [min]	Area [µV·sec]	Height [µV]	Area%	Height%	Quantity	NTP	Resolution	Symmetry Factor	Warning
1	Unknown	9	20.927	24582985	593413	49.957	63.120	N/A	6621	1.712	3.734	
2	Unknown	9	23.220	24625484	346721	50.043	36.880	N/A	3141	N/A	7.061	

Chromatogram Name 191-Ilecat-sec-alc-IBN5-1ml-min-hex-CH9
Sample Name
Channel Name 230.0nm

#	Peak Name	CH	tR [min]	Area [µV·sec]	Height [µV]	Area%	Height%	Quantity	NTP	Resolution	Symmetry Factor	Warning
1	Unknown	9	20.797	5193028	126930	82.784	86.471	N/A	6632	2.799	3.341	
2	Unknown	9	24.103	1079969	19858	17.216	13.529	N/A	5121	N/A	3.685	

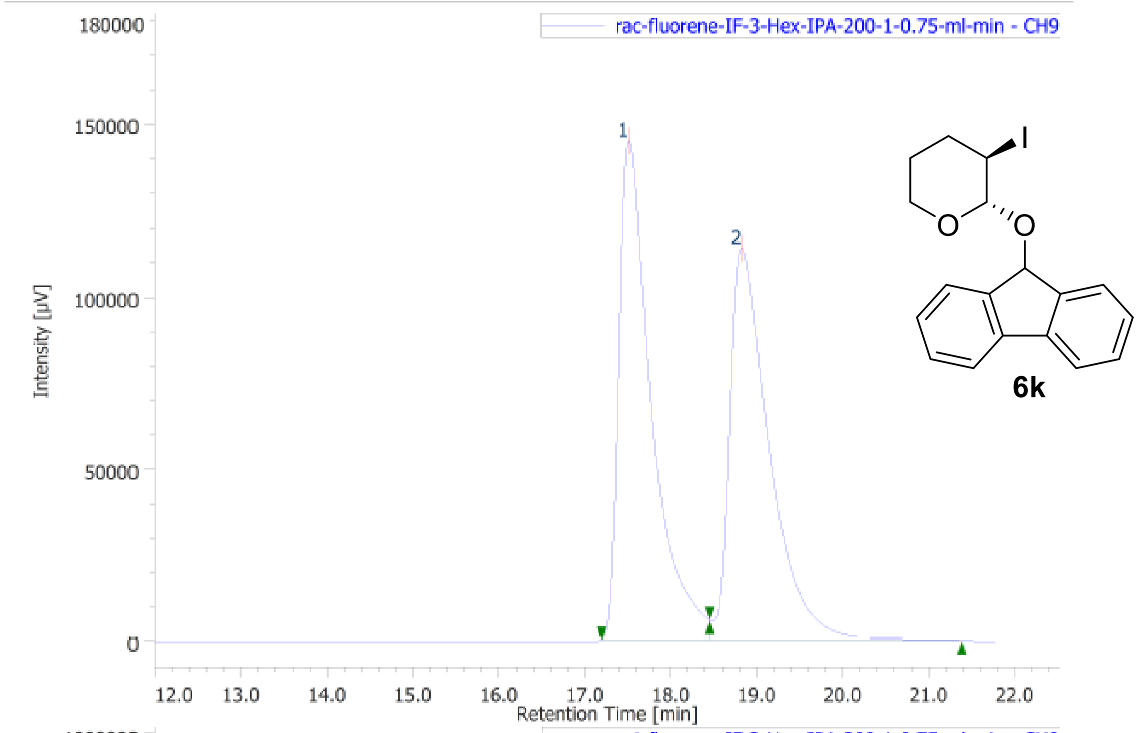

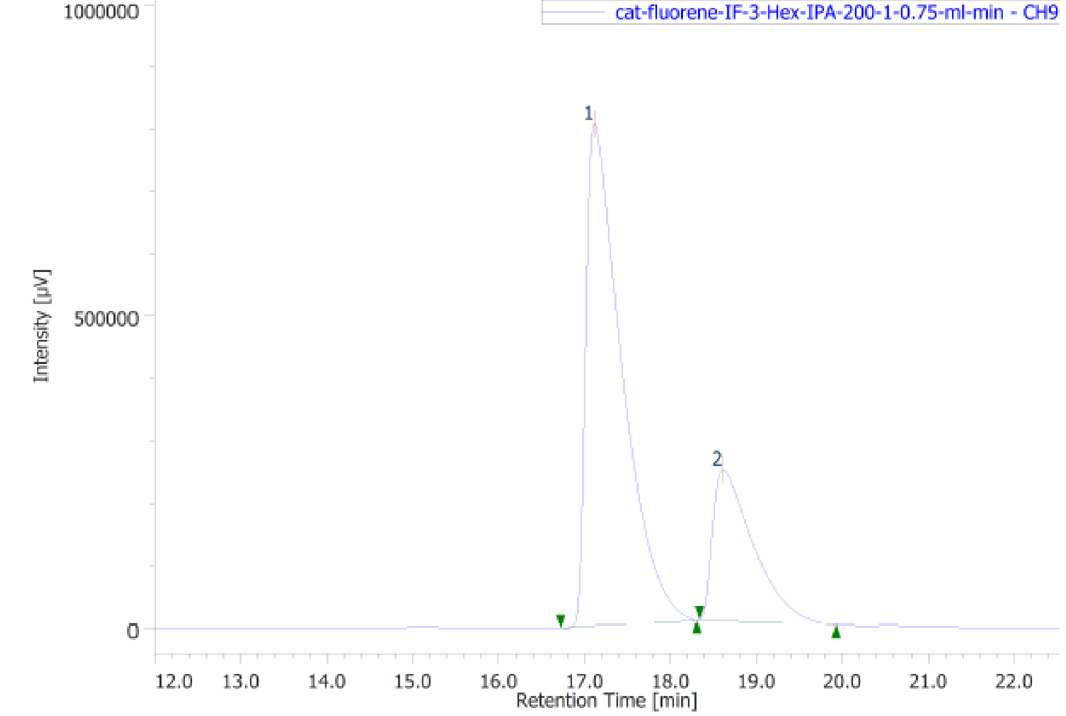

Channel & Peak Information Table

Chromatogram Name rac−fluorene−IF−3−Hex−IPA−200−1−0.75−ml−min−CH9
Sample Name
Channel Name 260.0nm

#	Peak Name	CH	tR [min]	Area [μV·sec]	Height [μV]	Area%	Height%	Quantity	NTP	Resolution	Symmetry Factor	Warning
1	Unknown	9	17.510	3827459	144696	50.945	55.929	N/A	12143	1.847	2.361	
2	Unknown	9	18.827	3685392	114016	49.055	44.071	N/A	9002	N/A	N/A	

Chromatogram Name cat−fluorene−IF−3−Hex−IPA−200−1−0.75−ml−min−CH9
Sample Name
Channel Name 260.0nm

#	Peak Name	CH	tR [min]	Area [μV·sec]	Height [μV]	Area%	Height%	Quantity	NTP	Resolution	Symmetry Factor	Warning
1	Unknown	9	17.117	23435895	804764	74.687	76.903	N/A	8471	1.889	2.717	
2	Unknown	9	18.613	7942943	241706	25.313	23.097	N/A	7758	N/A	2.517	

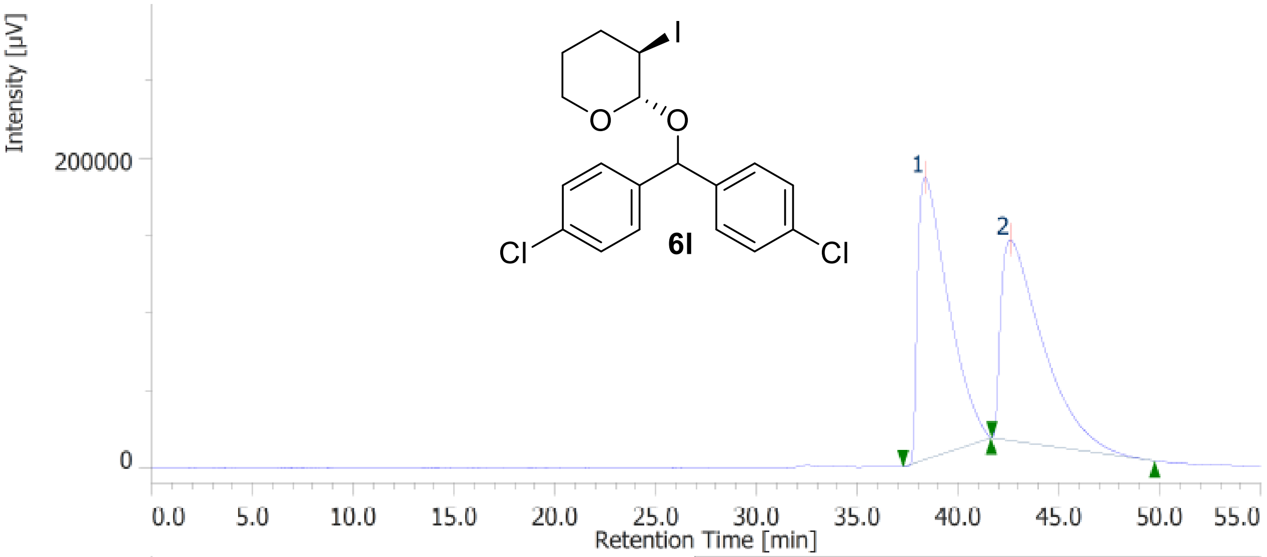

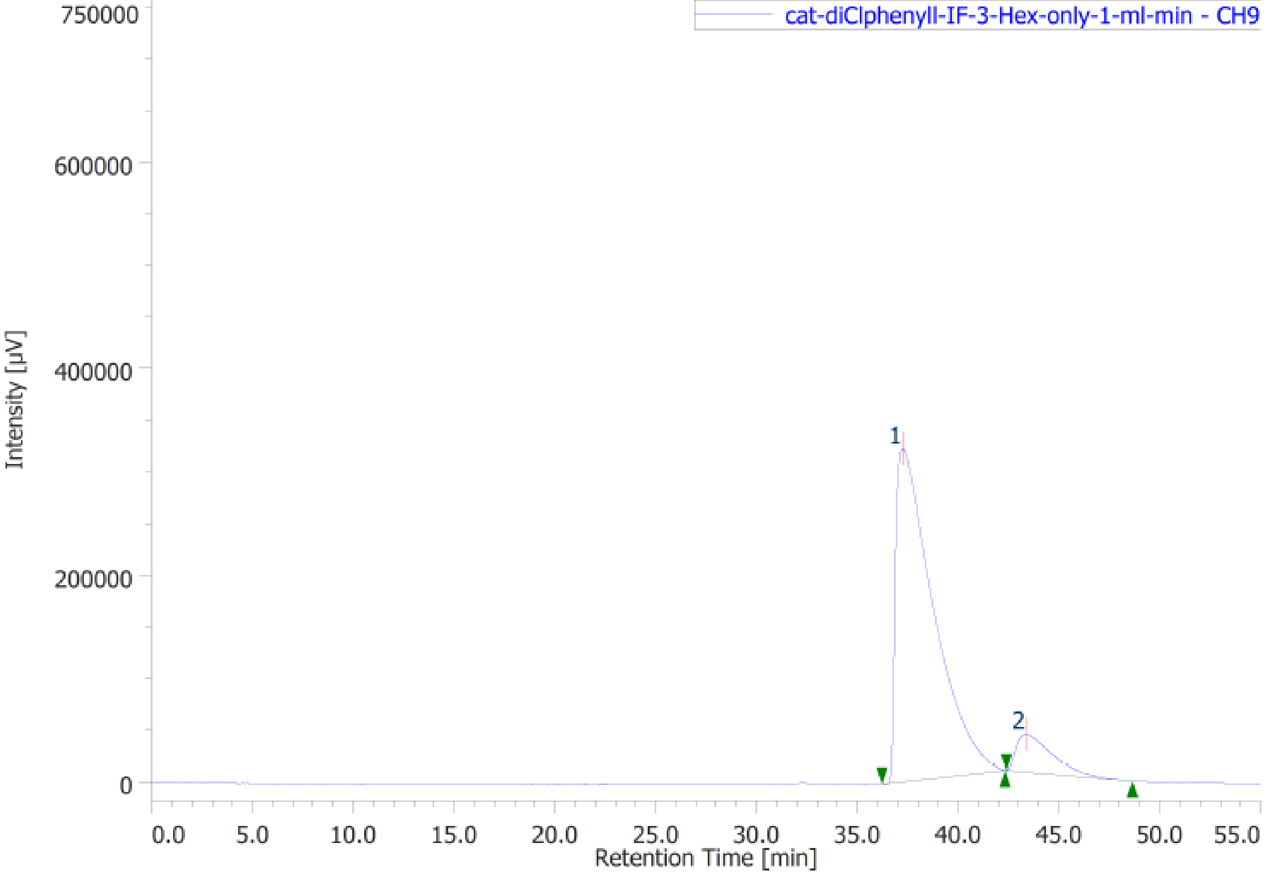

Channel & Peak Information Table

Chromatogram Name rac−diClphenylI−IF−3−Hex−only−1−ml−min−CH9
Sample Name
Channel Name 227.0nm

#	Peak Name	CH	tR [min]	Area [µV·sec]	Height [µV]	Area%	Height%	Quantity	NTP	Resolution	Symmetry Factor	Warning
1	Unknown	9	38.353	19279854	181930	50.650	58.422	N/A	2963	1.300	2.788	
2	Unknown	9	42.577	18784643	129477	49.350	41.578	N/A	2122	N/A	3.359	

Chromatogram Name cat−diClphenylI−IF−3−Hex−only−1−ml−min−CH9
Sample Name
Channel Name 235.0nm

#	Peak Name	CH	tR [min]	Area [µV·sec]	Height [µV]	Area%	Height%	Quantity	NTP	Resolution	Symmetry Factor	Warning
1	Unknown	9	37.243	41881139	321699	90.116	89.748	N/A	1980	1.854	4.045	
2	Unknown	9	43.357	4593635	36748	9.884	10.252	N/A	2825	N/A	2.468	

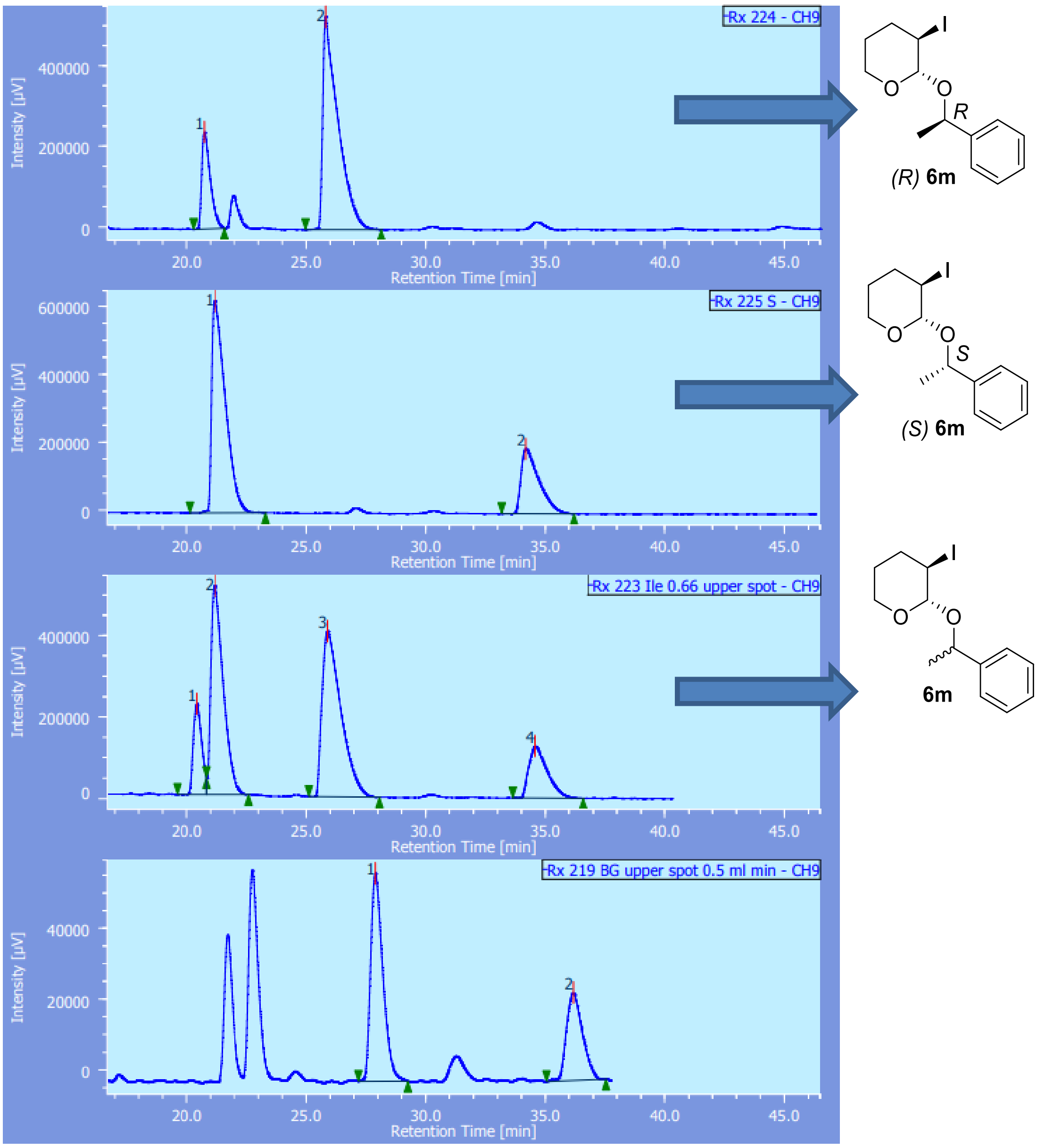

Chromatogram Name Rx 224-CH9
Sample Name
Channel Name 210.0nm

#	Peak Name	CH	tR [min]	Area [µV·sec]	Height [µV]	Area%	Height%	Quantity	NTP	Resolution	Symmetry Factor
1	Unknown	9	20.745	5870497	239794	20.092	31.224	N/A	16929	5.744	1.824
2	Unknown	9	25.810	23347761	528183	79.908	68.776	N/A	8341	N/A	3.266

Chromatogram Name Rx 225 S-CH9
Sample Name
Channel Name 210.0nm

#	Peak Name	CH	tR [min]	Area [µV·sec]	Height [µV]	Area%	Height%	Quantity	NTP	Resolution	Symmetry Factor
1	Unknown	9	21.170	24498796	622369	71.498	76.545	N/A	6706	11.056	2.770
2	Unknown	9	34.180	9766357	190710	28.502	23.455	N/A	10636	N/A	2.231

Chromatogram Name Rx 223 Ile 0.66 upper spot-CH9
Sample Name
Channel Name 210.0nm

#	Peak Name	CH	tR [min]	Area [µV·sec]	Height [µV]	Area%	Height%	Quantity	NTP	Resolution	Symmetry Factor
1	Unknown	9	20.427	5307454	221431	10.127	17.515	N/A	14955	0.920	N/A
2	Unknown	9	21.163	18174390	511058	34.679	40.423	N/A	8166	3.939	N/A
3	Unknown	9	25.880	22232280	405399	42.423	32.066	N/A	4997	6.093	2.181
4	Unknown	9	34.565	6692683	126372	12.771	9.996	N/A	9836	N/A	1.721

Chromatogram Name Rx 219 BG upper spot 0.5 ml min-CH9
Sample Name
Channel Name 210.0nm

#	Peak Name	CH	tR [min]	Area [µV·sec]	Height [µV]	Area%	Height%	Quantity	NTP	Resolution	Symmetry Factor
1	Unknown	9	27.892	2071394	58654	64.558	70.307	N/A	14694	7.748	1.482
2	Unknown	9	36.175	1137188	24772	35.442	29.693	N/A	13987	N/A	1.261

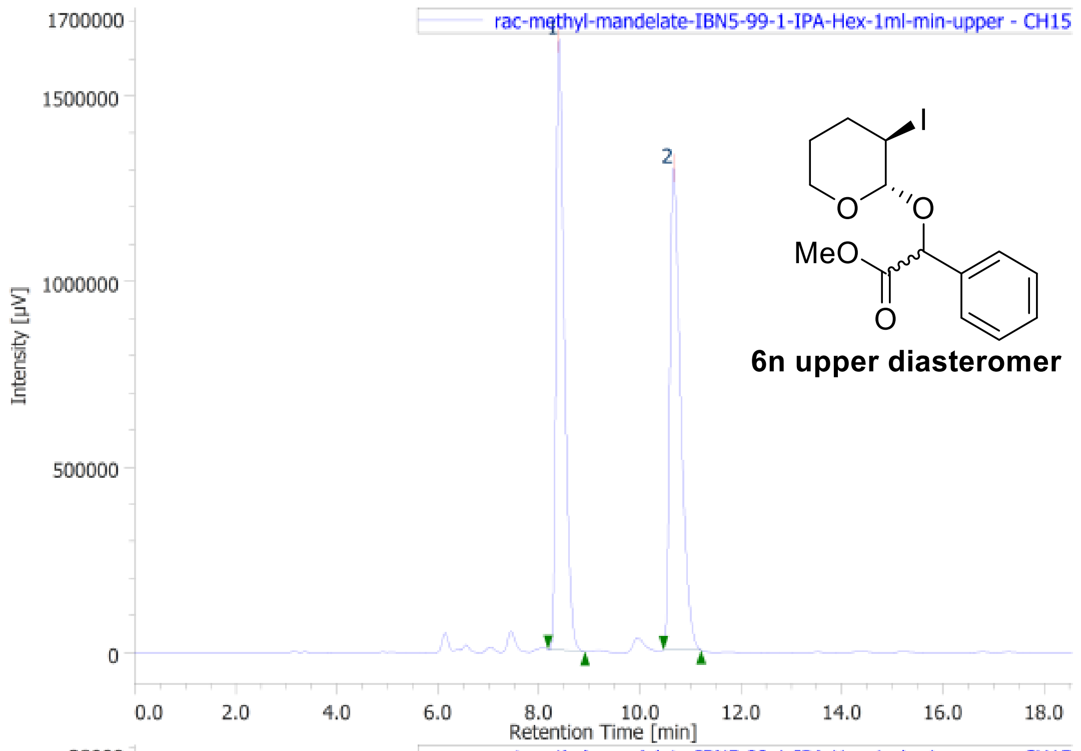

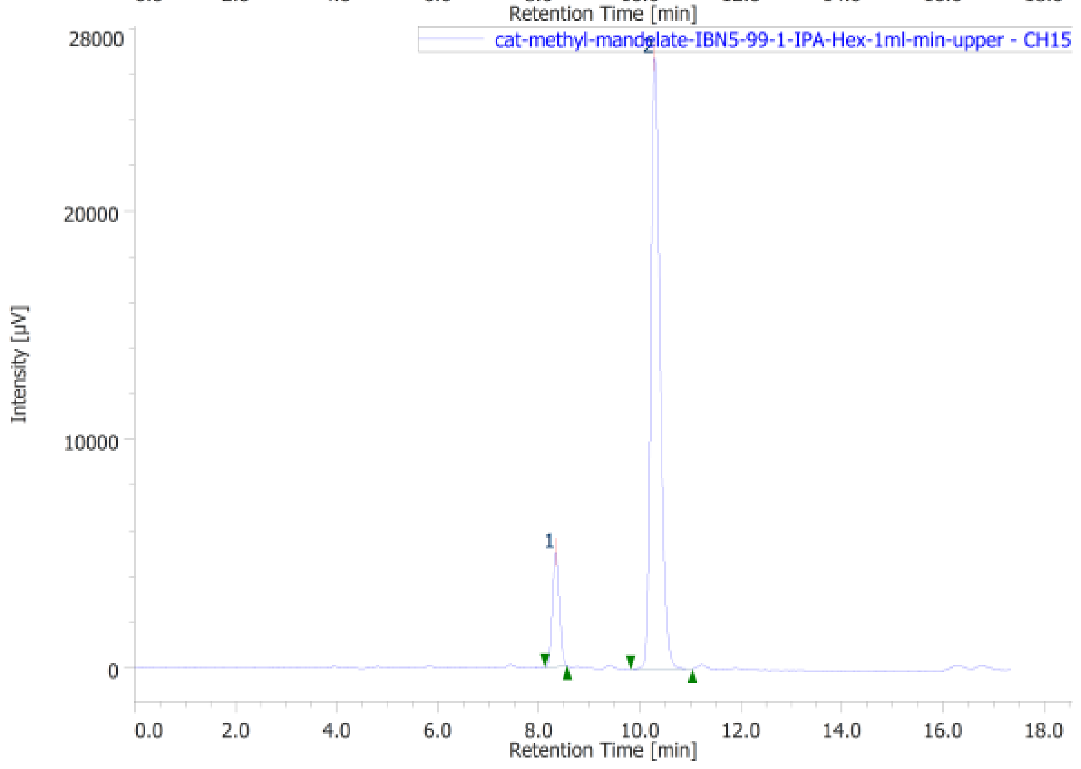

Channel & Peak Information Table

Chromatogram Name rac−methyl−mandelate−IBN5−99−1−IPA−Hex−1ml−min−upper−CH15
Sample Name
Channel Name 220.0nm

#	Peak Name	CH	tR [min]	Area [µV·sec]	Height [µV]	Area%	Height%	Quantity	NTP	Resolution	Symmetry Factor	Warning
1	Unknown	15	8.393	18880583	1646159	49.301	55.923	N/A	12812	6.640	1.642	
2	Unknown	15	10.660	19416253	1297470	50.699	44.077	N/A	12080	N/A	1.759	

Chromatogram Name cat−methyl−mandelate−IBN5−99−1−IPA−Hex−1ml−min−upper−CH15
Sample Name
Channel Name 270.0nm

#	Peak Name	CH	tR [min]	Area [µV·sec]	Height [µV]	Area%	Height%	Quantity	NTP	Resolution	Symmetry Factor	Warning
1	Unknown	15	8.330	48473	5016	12.333	15.768	N/A	16942	6.669	1.100	
2	Unknown	15	10.290	344566	26797	87.667	84.232	N/A	15245	N/A	1.304	

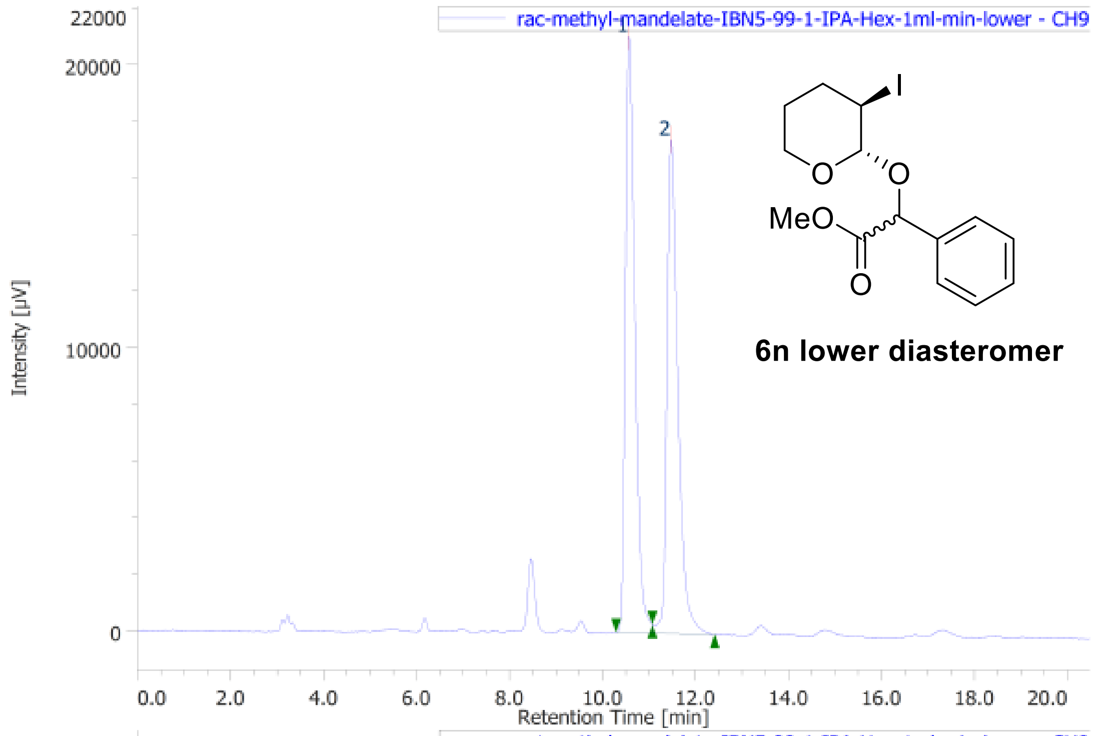

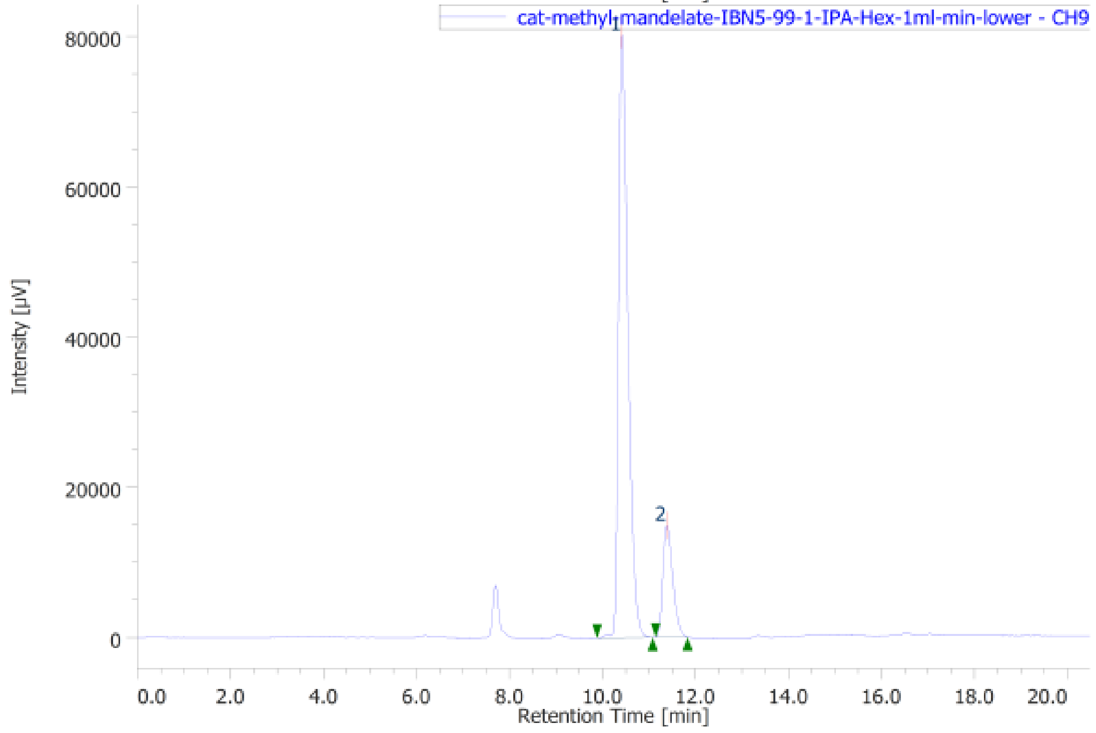

Channel & Peak Information Table

Chromatogram Name rac−methyl−mandelate−IBN5−99−1−IPA−Hex−1ml−min−lower−CH9
Sample Name
Channel Name 275.0nm

#	Peak Name	CH	tR [min]	Area [μV·sec]	Height [μV]	Area%	Height%	Quantity	NTP	Resolution	Symmetry Factor	Warning
1	Unknown	9	10.570	302673	21059	51.482	54.706	N/A	12781	2.314	1.467	
2	Unknown	9	11.470	285245	17436	48.518	45.294	N/A	12774	N/A	1.602	

Chromatogram Name cat−methyl−mandelate−IBN5−99−1−IPA−Hex−1ml−min−lower−CH9
Sample Name
Channel Name 270.0nm

#	Peak Name	CH	tR [min]	Area [μV·sec]	Height [μV]	Area%	Height%	Quantity	NTP	Resolution	Symmetry Factor	Warning
1	Unknown	9	10.417	1129850	80228	83.928	84.406	N/A	13028	2.591	1.592	
2	Unknown	9	11.383	216358	14822	16.072	15.594	N/A	14129	N/A	1.387	

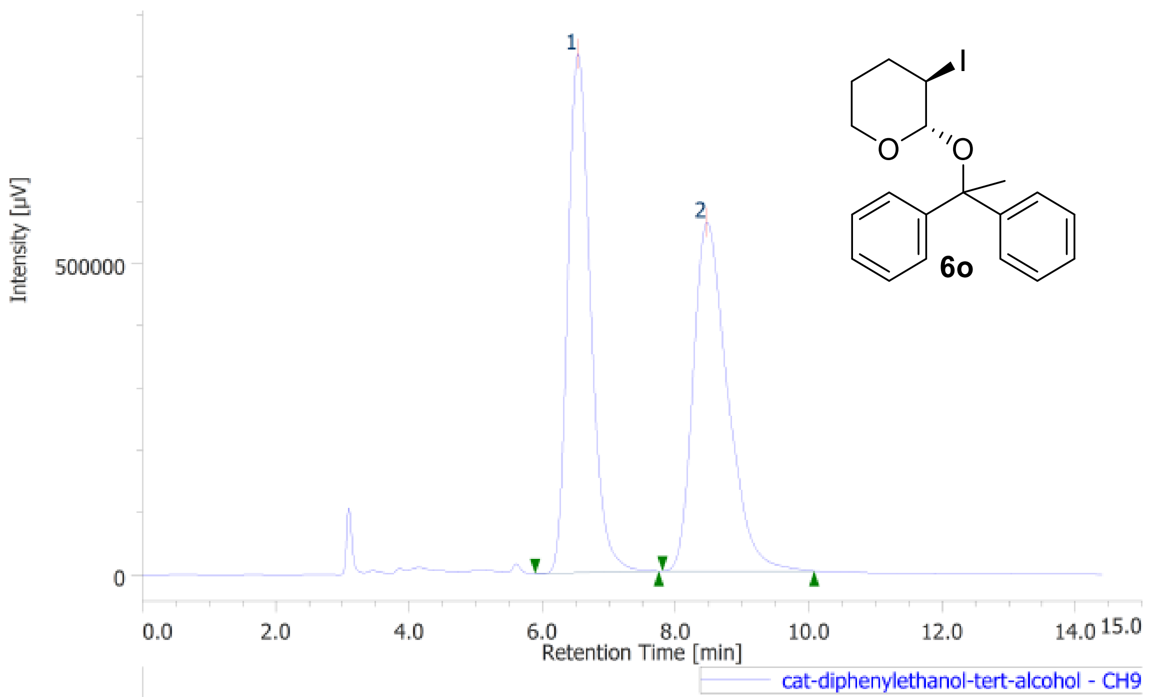

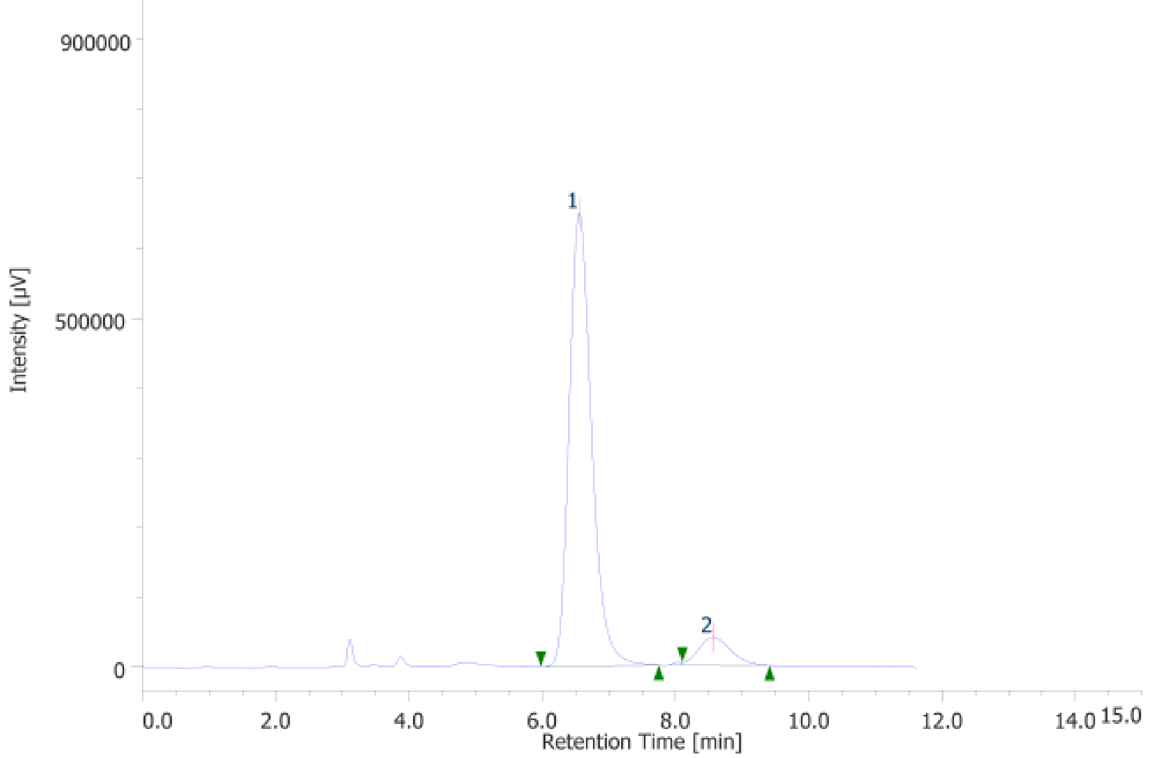

Channel & Peak Information Table

Chromatogram Name cat−diphenylethanol−tert−alcohol−CH9
Sample Name
Channel Name 210.0nm

#	Peak Name	CH	tR [min]	Area [µV·sec]	Height [µV]	Area%	Height%	Quantity	NTP	Resolution	Symmetry Factor	Warning
1	Unknown	9	6.533	19801492	833058	49.552	59.796	N/A	1808	2.528	1.261	
2	Unknown	9	8.470	20159909	560120	50.448	40.204	N/A	1352	N/A	1.396	

Chromatogram Name cat−diphenylethanol−tert−alcohol−CH9
Sample Name
Channel Name 210.0nm

#	Peak Name	CH	tR [min]	Area [µV·sec]	Height [µV]	Area%	Height%	Quantity	NTP	Resolution	Symmetry Factor	Warning
1	Unknown	9	6.550	15090339	649445	91.777	94.206	N/A	1922	2.710	1.276	
2	Unknown	9	8.563	1352044	39943	8.223	5.794	N/A	1474	N/A	N/A	

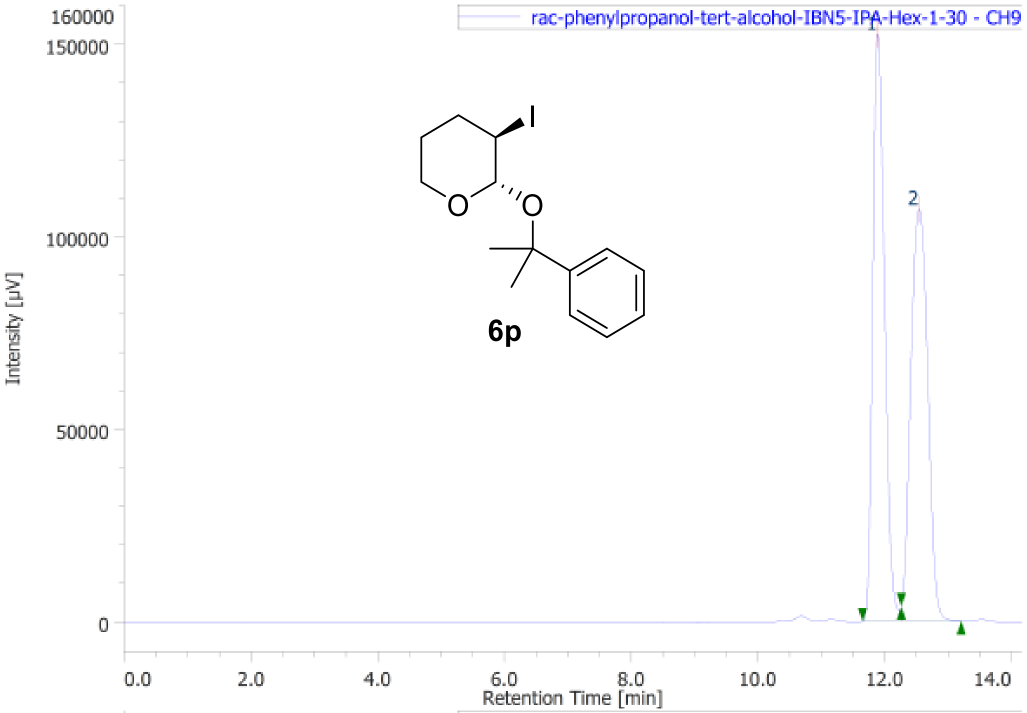

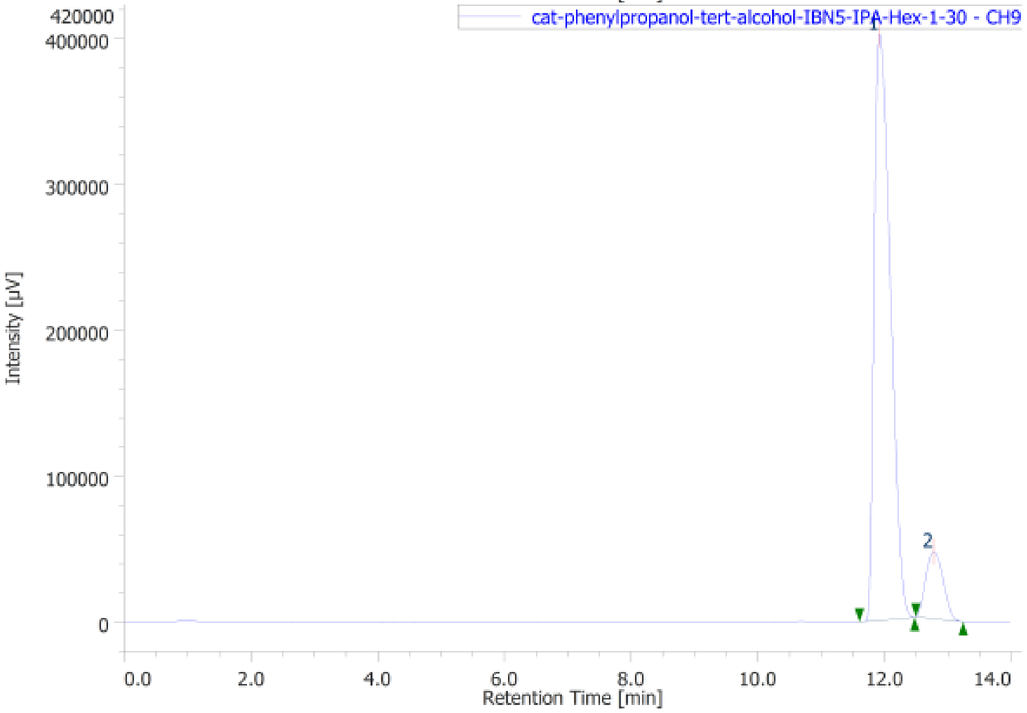

Channel & Peak Information Table

Chromatogram Name rac−phenylpropanol−tert−alcohol−IBN5−IPA−Hex−1−30−CH9
Sample Name
Channel Name 262.0nm

#	Peak Name	CH	tR [min]	Area [µV·sec]	Height [µV]	Area%	Height%	Quantity	NTP	Resolution	Symmetry Factor	Warning
1	Unknown	9	11.887	1980943	152437	49.657	58.765	N/A	18743	1.509	1.292	
2	Unknown	9	12.540	2008289	106963	50.343	41.235	N/A	9264	N/A	1.057	

Chromatogram Name cat−phenylpropanol−tert−alcohol−IBN5−IPA−Hex−1−30−CH9
Sample Name
Channel Name 262.0nm

#	Peak Name	CH	tR [min]	Area [µV·sec]	Height [µV]	Area%	Height%	Quantity	NTP	Resolution	Symmetry Factor	Warning
1	Unknown	9	11.917	7193643	401657	89.182	89.745	N/A	9235	1.674	1.657	
2	Unknown	9	12.773	872630	45896	10.818	10.255	N/A	9281	N/A	1.123	

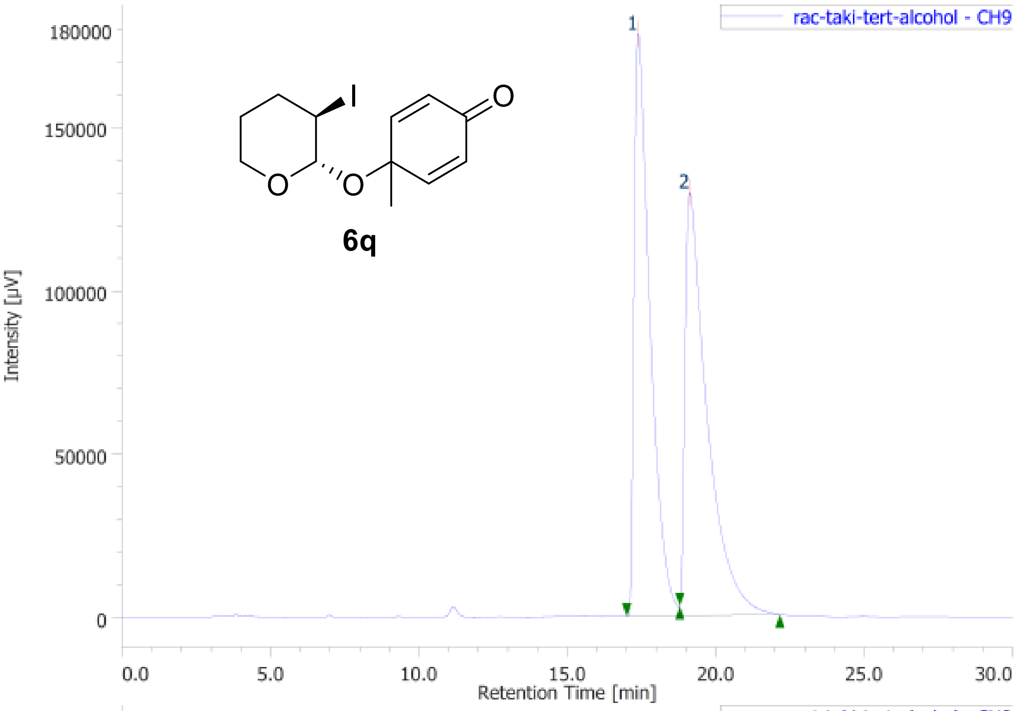

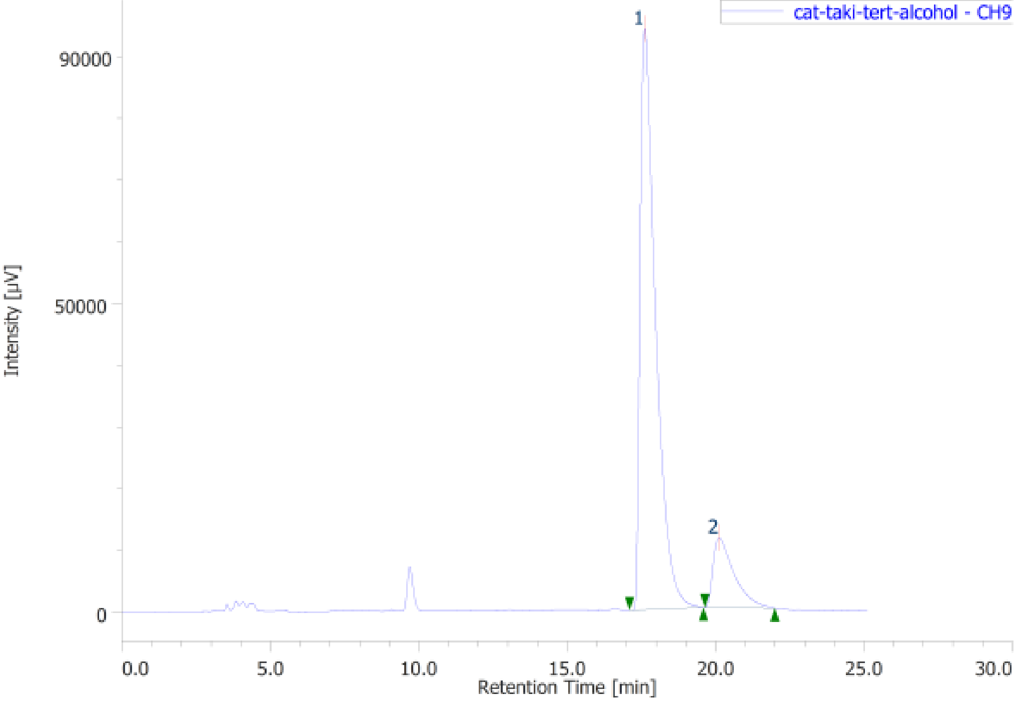

Channel & Peak Information Table

Chromatogram Name rac−taki−tert−alcohol−CH9
Sample Name
Channel Name 265.0nm

#	Peak Name	CH	tR [min]	Area [µV·sec]	Height [µV]	Area%	Height%	Quantity	NTP	Resolution	Symmetry Factor	Warning
1	Unknown	9	17.390	6693661	178160	49.964	57.929	N/A	5230	1.579	2.525	
2	Unknown	9	19.127	6703192	129387	50.036	42.071	N/A	3787	N/A	3.383	

Chromatogram Name cat−taki−tert−alcohol−CH9
Sample Name
Channel Name 265.0nm

#	Peak Name	CH	tR [min]	Area [µV·sec]	Height [µV]	Area%	Height%	Quantity	NTP	Resolution	Symmetry Factor	Warning
1	Unknown	9	17.603	3418665	94042	86.099	89.298	N/A	5819	2.335	2.285	
2	Unknown	9	20.107	551964	11271	13.901	10.702	N/A	4298	N/A	2.356	

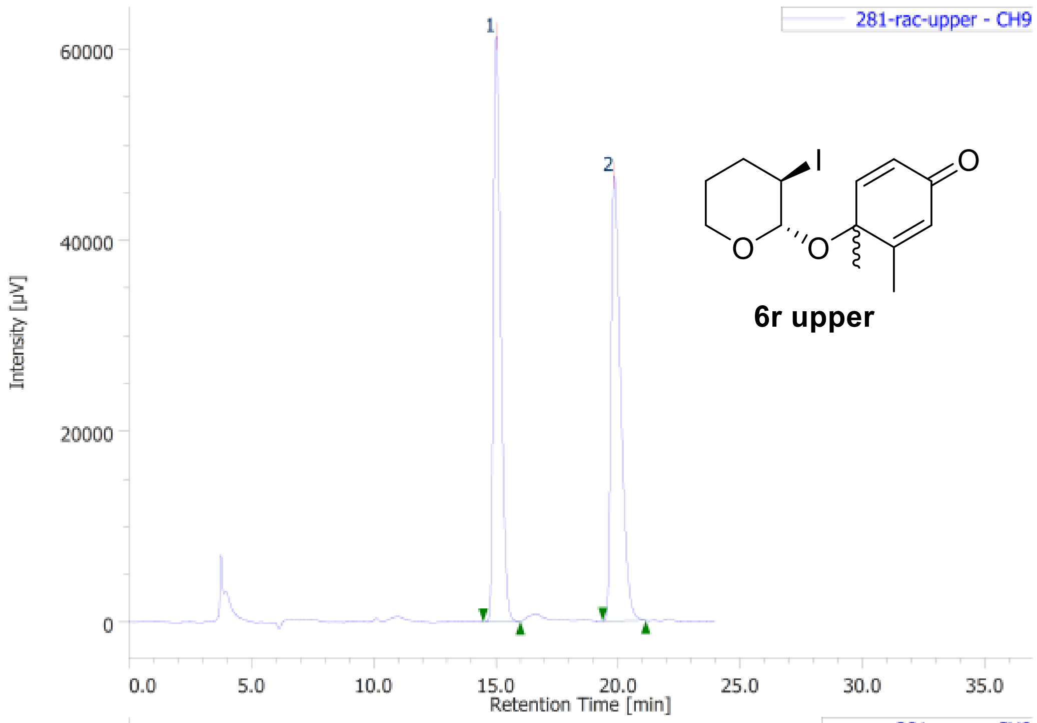

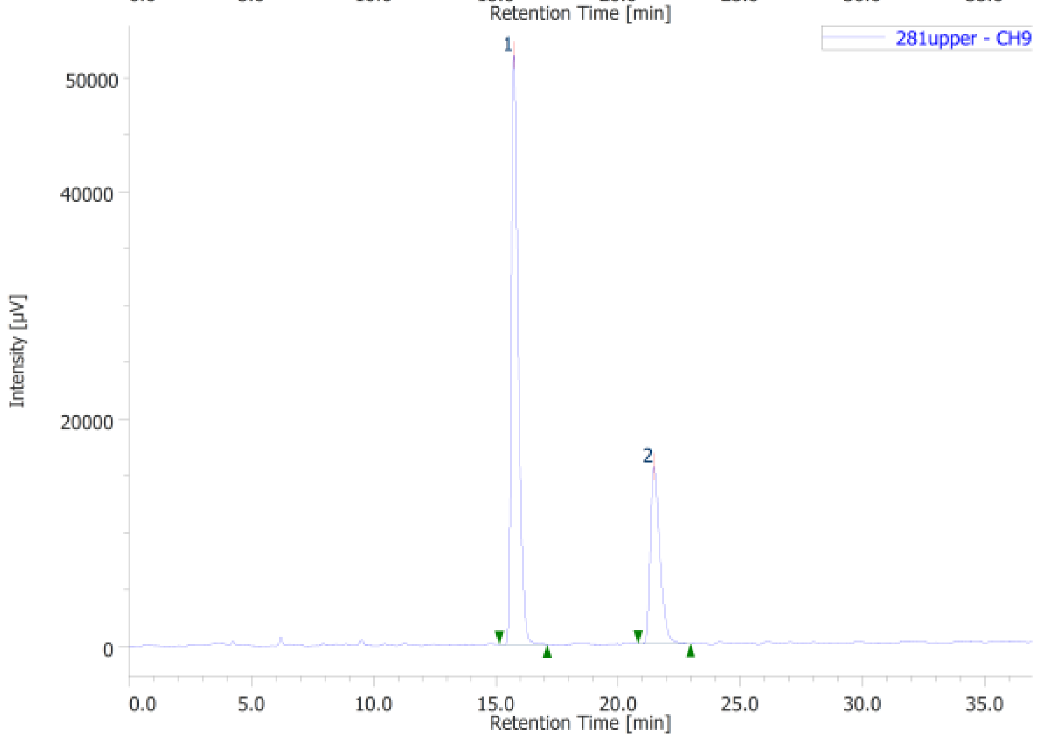

Channel & Peak Information Table

Chromatogram Name 281−rac−upper−CH9
Sample Name
Channel Name 254.0nm

#	Peak Name	CH	tR [min]	Area [µV·sec]	Height [µV]	Area%	Height%	Quantity	NTP	Resolution	Symmetry Factor	Warning
1	Unknown	9	15.007	1274693	61313	49.805	56.779	N/A	12119	7.696	1.532	
2	Unknown	9	19.837	1284669	46672	50.195	43.221	N/A	12373	N/A	1.722	

Chromatogram Name 281upper−CH9
Sample Name
Channel Name 255.0nm

#	Peak Name	CH	tR [min]	Area [µV·sec]	Height [µV]	Area%	Height%	Quantity	NTP	Resolution	Symmetry Factor	Warning
1	Unknown	9	15.730	1027046	51775	70.917	76.872	N/A	15185	9.544	1.606	
2	Unknown	9	21.460	421191	15577	29.083	23.128	N/A	15329	N/A	1.492	

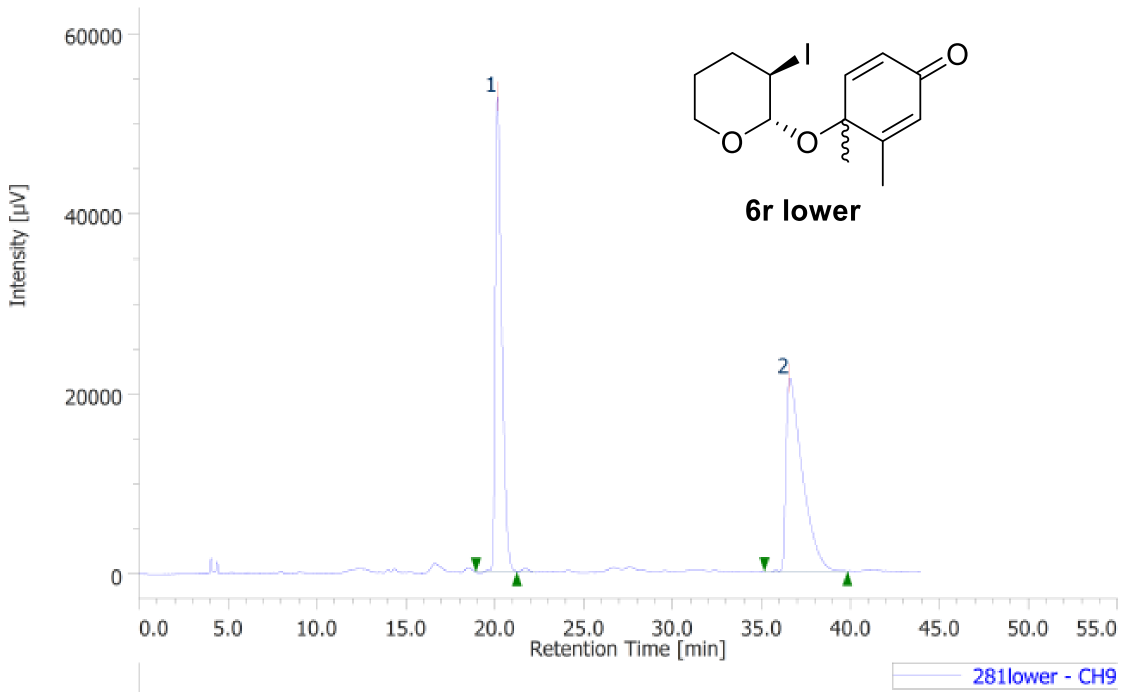

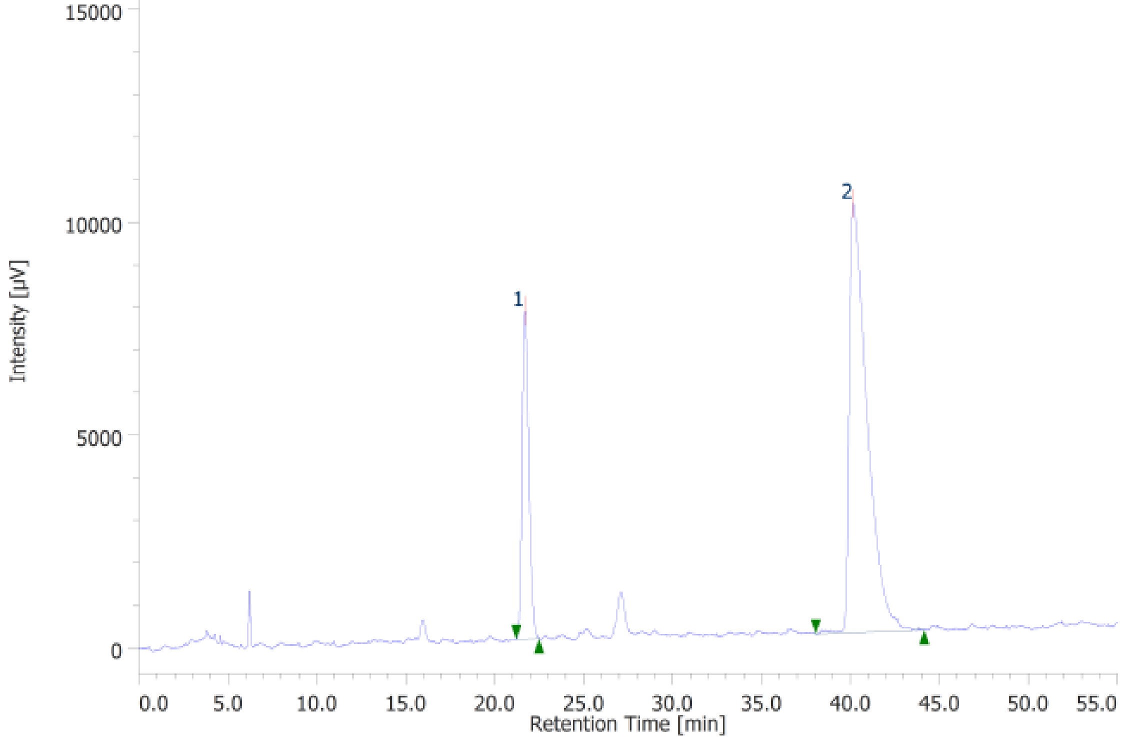

Channel & Peak Information Table

Chromatogram Name 281−rac−lower−CH9
Sample Name
Channel Name 254.0nm

#	Peak Name	CH	tR [min]	Area [μV·sec]	Height [μV]	Area%	Height%	Quantity	NTP	Resolution	Symmetry Factor	Warning
1	Unknown	9	20.147	1383649	52811	50.223	71.083	N/A	14161	14.313	1.692	
2	Unknown	9	36.580	1371360	21484	49.777	28.917	N/A	8106	N/A	2.922	

Chromatogram Name 281lower−CH9
Sample Name
Channel Name 260.0nm

#	Peak Name	CH	tR [min]	Area [μV·sec]	Height [μV]	Area%	Height%	Quantity	NTP	Resolution	Symmetry Factor	Warning
1	Unknown	9	21.683	199979	7707	22.100	43.343	N/A	16254	15.139	1.347	
2	Unknown	9	40.163	704904	10075	77.900	56.657	N/A	8261	N/A	2.690	

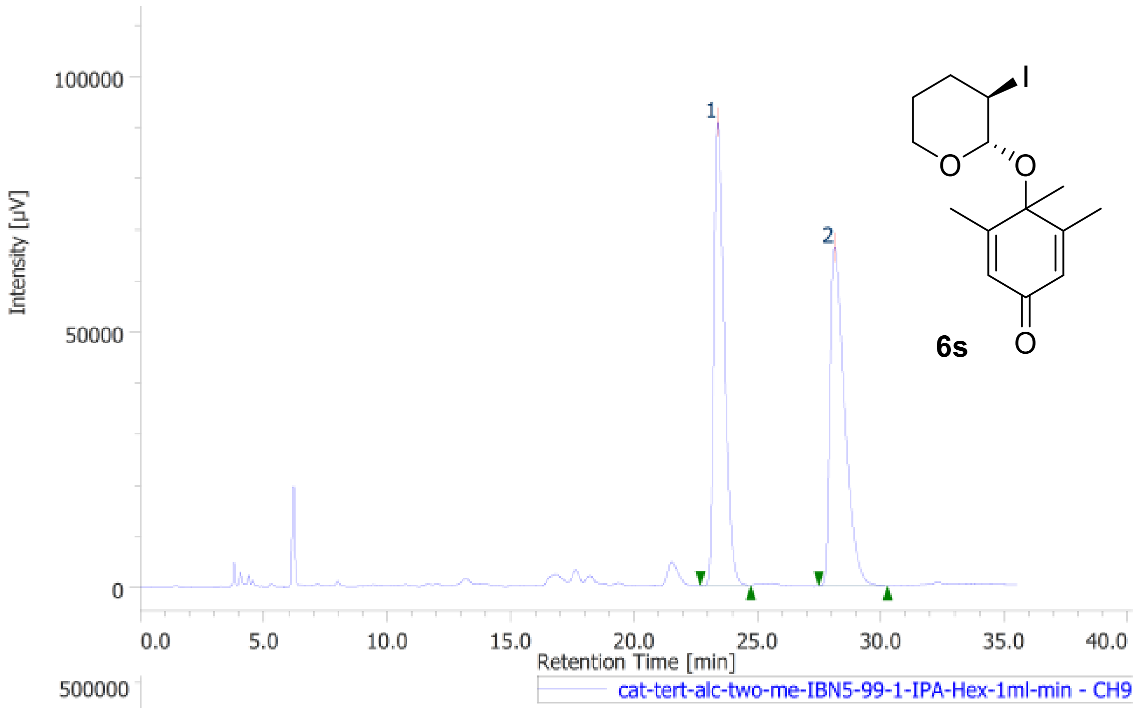

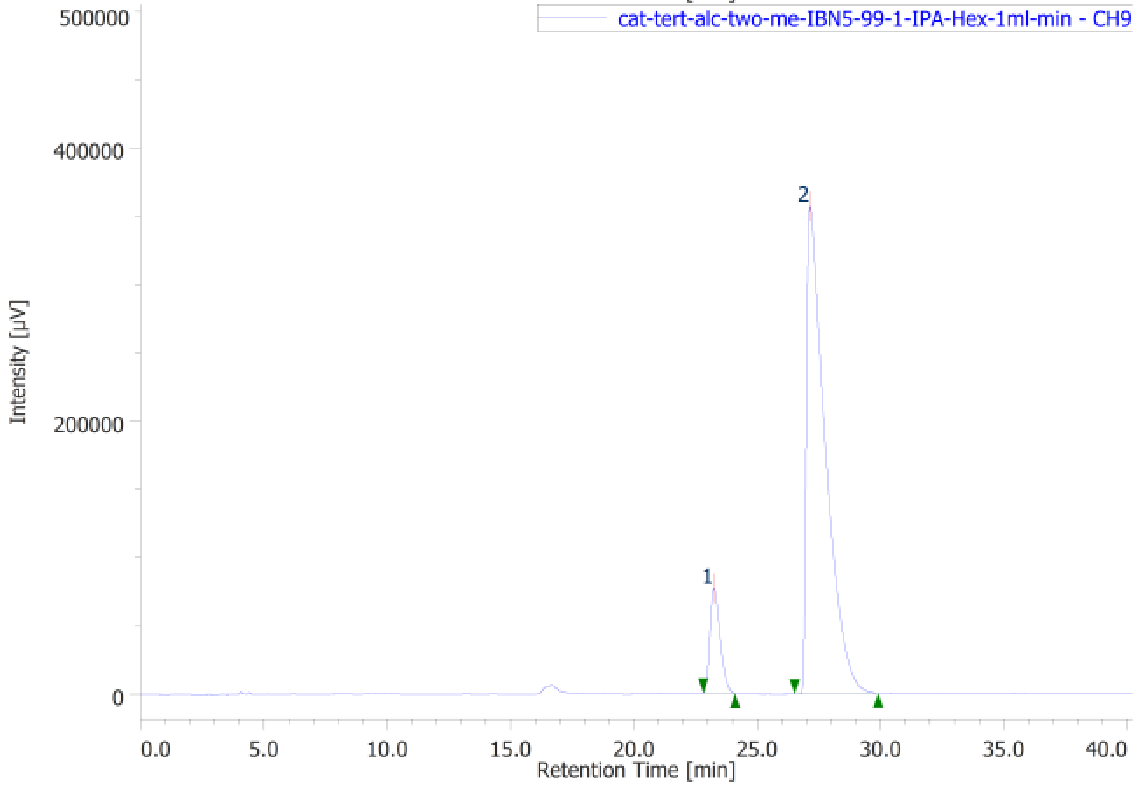

Channel & Peak Information Table

Chromatogram Name rac−tert−alc−two−me−IBN5−99−1−IPA−Hex−1ml−min−CH9

Sample Name

Channel Name 245.0nm

#	Peak Name	CH	tR [min]	Area [µV·sec]	Height [µV]	Area%	Height%	Quantity	NTP	Resolution	Symmetry Factor	Warning
1	Unknown	9	23.387	2661365	90709	49.328	57.801	N/A	14985	5.197	1.528	
2	Unknown	9	28.127	2733906	66224	50.672	42.199	N/A	11162	N/A	1.922	

Chromatogram Name cat−tert−alc−two−me−IBN5−99−1−IPA−Hex−1ml−min−CH9

Sample Name

Channel Name 245.0nm

#	Peak Name	CH	tR [min]	Area [µV·sec]	Height [µV]	Area%	Height%	Quantity	NTP	Resolution	Symmetry Factor	Warning
1	Unknown	9	23.233	2201537	76912	10.375	17.741	N/A	15055	3.705	1.411	
2	Unknown	9	27.133	19018352	356613	89.625	82.259	N/A	6428	N/A	3.510	

17. X-ray Crystallographic Analysis

2a (CCDC 2057246)

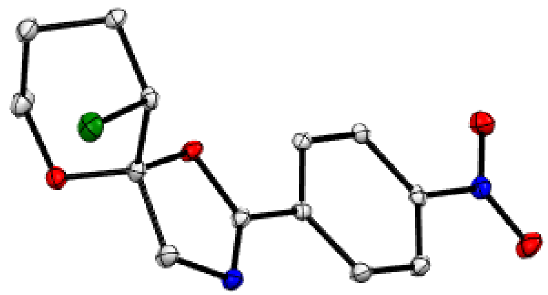

Empirical formula	$C_{13}H_{13}IN_2O_4$
Formula weight	388.164
Temperature/K	100
Crystal system	orthorhombic
Space group	$P2_12_12_1$
a/Å	6.40713(17)
b/Å	6.77975(19)
c/Å	32.2251(8)
α/°	90
β/°	90
γ/°	90
Volume/Å^3	1399.82(6)
Z	4
ρ_{calc}g/cm^3	1.842
μ/mm^{-1}	2.302
F(000)	758.8
Crystal size/mm^3	0.147 × 0.113 × 0.065
Radiation	Mo Kα (λ = 0.71073)
2Θ range for data collection/°	5.06 to 59.02
Index ranges	$-8 \le h \le 8$, $-8 \le k \le 8$, $-41 \le l \le 32$
Reflections collected	11416
Independent reflections	3386 [R_{int} = 0.0159, R_{sigma} = 0.0163]
Data/restraints/parameters	3386/0/181
Goodness-of-fit on F^2	1.032
Final R indexes [I>=2σ (I)]	R_1 = 0.0162, wR_2 = 0.0364
Final R indexes [all data]	R_1 = 0.0171, wR_2 = 0.0367
Largest diff. peak/hole / e Å^{-3}	0.30/-0.31
Flack parameter	-0.012(17)

Datablock: No2020-3-C1-1

Bond precision:	C-C = 0.0025 A		Wavelength=0.71073
Cell:	a=6.40713(17)	b=6.77975(19)	c=32.2251(8)
	alpha=90	beta=90	gamma=90
Temperature:	100 K		

	Calculated	Reported
Volume	1399.82(6)	1399.82(6)
Space group	P 21 21 21	P 21 21 21
Hall group	P 2ac 2ab	P 2ac 2ab
Moiety formula	C13 H13 I N2 O4	C13 H13 I N2 O4
Sum formula	C13 H13 I N2 O4	C13 H13 I N2 O4
Mr	388.15	388.16
Dx,g cm-3	1.842	1.842
Z	4	4
Mu (mm-1)	2.302	2.302
F000	760.0	758.8
F000'	758.44	
h,k,lmax	8,9,44	8,8,41
Nref	3894[2283]	3386
Tmin,Tmax	0.739,0.861	0.807,1.000
Tmin'	0.706	

Correction method= # Reported T Limits: Tmin=0.807 Tmax=1.000
AbsCorr = GAUSSIAN

Data completeness= 1.48/0.87 Theta(max)= 29.510

R(reflections)= 0.0162(3299) wR2(reflections)= 0.0367(3386)

S = 1.032 Npar= 181

The following ALERTS were generated. Each ALERT has the format
test-name_ALERT_alert-type_alert-level.
Click on the hyperlinks for more details of the test.

Alert level C

```
PLAT790_ALERT_4_C Centre of Gravity not Within Unit Cell: Resd.  #          1 Note
                   C13 H13 I N2 O4
```

Alert level G

```
PLAT068_ALERT_1_G Reported F000 Differs from Calcd (or Missing)...     Please Check
PLAT073_ALERT_1_G H-atoms ref, but _hydrogen_treatment Reported as     constr Check
PLAT398_ALERT_2_G Deviating  C-O-C    Angle From 120 for O1        .    106.2 Degree
PLAT720_ALERT_4_G Number of Unusual/Non-Standard Labels ..........        2 Note
PLAT769 ALERT 4 G CIF Embedded explicitly supplied scattering data     Please Note
PLAT791_ALERT_4_G Model has Chirality at C4            (Sohnke SpGr)        R Verify
PLAT791_ALERT_4_G Model has Chirality at C11           (Sohnke SpGr)        S Verify
PLAT910_ALERT_3_G Missing # of FCF Reflection(s) Below Theta(Min).        1 Note
PLAT912_ALERT_4_G Missing # of FCF Reflections Above STh/L=  0.600      170 Note
PLAT958_ALERT_1_G Calculated (ThMax) and Actual (FCF) Lmax Differ.        3 Units
PLAT978_ALERT_2_G Number C-C Bonds with Positive Residual Density.        8 Info
PLAT982_ALERT_1_G The  I-f'=   -0.4119 Deviates from  IT-value =    -0.4742 Check
PLAT983_ALERT_1_G The  I-f"=    1.8326 Deviates from  IT-Value =     1.8119 Check
```

0 **ALERT level A** = Most likely a serious problem - resolve or explain
0 **ALERT level B** = A potentially serious problem, consider carefully
1 **ALERT level C** = Check. Ensure it is not caused by an omission or oversight
13 **ALERT level G** = General information/check it is not something unexpected

5 ALERT type 1 CIF construction/syntax error, inconsistent or missing data
2 ALERT type 2 Indicator that the structure model may be wrong or deficient
1 ALERT type 3 Indicator that the structure quality may be low
6 ALERT type 4 Improvement, methodology, query or suggestion
0 ALERT type 5 Informative message, check

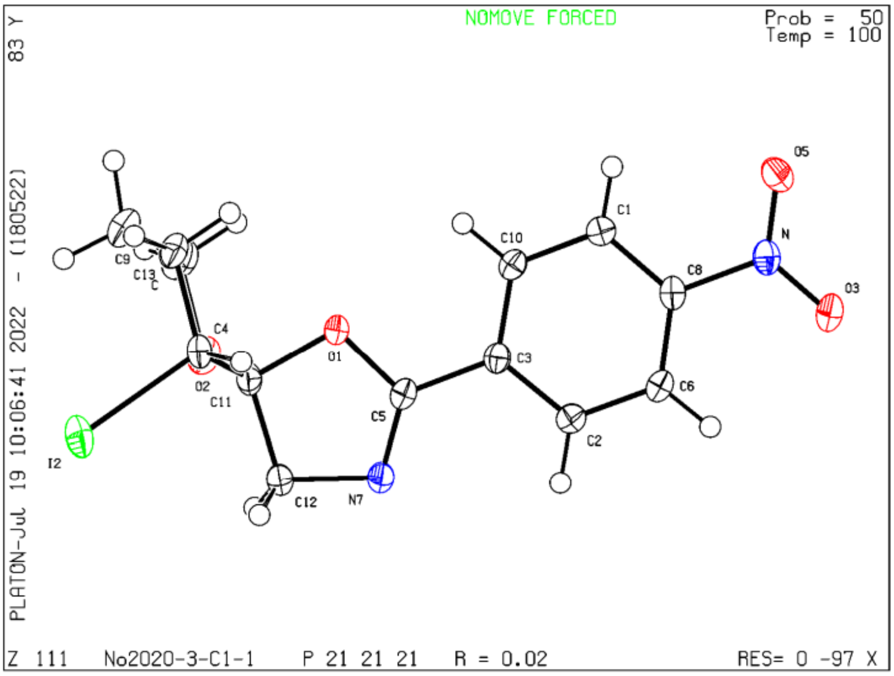

Supplement: Supplementary file 1 [file DataSheet1.zip › 1-Supporting information.PDF]
