# Supplementary material for: New anionic cobalt(III) complexes enable enantioselective synthesis of spiro-fused oxazoline and iodoacetal derivatives
Source: Front Chem. 2022 Oct 13;10:1034291. doi: 10.3389/fchem.2022.1034291 (PMC9606352; doi:10.3389/fchem.2022.1034291)

checkCIF/PLATON report

Structure factors have been supplied for datablock(s) No2020-3-C1-1

THIS REPORT IS FOR GUIDANCE ONLY. IF USED AS PART OF A REVIEW PROCEDURE FOR PUBLICATION, IT SHOULD NOT REPLACE THE EXPERTISE OF AN EXPERIENCED CRYSTALLOGRAPHIC REFEREE.

No syntax errors found. CIF dictionary Interpreting this report

Datablock: No2020-3-C1-1

Bond precision: C-C = 0.0025 A Wavelength=0.71073

Cell:	a=6.40713(17)	b=6.77975(19)	c=32.2251(8)
	alpha=90	beta=90	gamma=90
Temperature:	100 K		

	Calculated	Reported
Volume	1399.82(6)	1399.82(6)
Space group	P 21 21 21	P 21 21 21
Hall group	P 2ac 2ab	P 2ac 2ab
Moiety formula	C13 H13 I N2 O4	C13 H13 I N2 O4
Sum formula	C13 H13 I N2 O4	C13 H13 I N2 O4
Mr	388.15	388.16
Dx,g cm-3	1.842	1.842
Z	4	4
Mu (mm-1)	2.302	2.302
F000	760.0	758.8
F000’	758.44	
h,k,lmax	8,9,44	8,8,41
Nref	3894[2283]	3386
Tmin,Tmax	0.739,0.861	0.807,1.000
Tmin’	0.706	

Correction method= # Reported T Limits: Tmin=0.807 Tmax=1.000
AbsCorr = GAUSSIAN

Data completeness= 1.48/0.87 Theta(max)= 29.510

R(reflections)= 0.0162(3299) wR2(reflections)= 0.0367(3386)

S = 1.032 Npar= 181

The following ALERTS were generated. Each ALERT has the format
test-name_ALERT_alert-type_alert-level.
Click on the hyperlinks for more details of the test.

Alert level C

```
PLAT790_ALERT_4_C Centre of Gravity not Within Unit Cell: Resd.  #          1 Note
              C13 H13 I N2 O4
```

Alert level G

```
PLAT068_ALERT_1_G Reported F000 Differs from Calcd (or Missing)...     Please Check
PLAT073_ALERT_1_G H-atoms ref, but _hydrogen_treatment Reported as     constr Check
PLAT398_ALERT_2_G Deviating  C-O-C   Angle From 120 for O1       .      106.2 Degree
PLAT720_ALERT_4_G Number of Unusual/Non-Standard Labels ..........          2 Note
PLAT769_ALERT_4_G CIF Embedded explicitly supplied scattering data     Please Note
PLAT791_ALERT_4_G Model has Chirality at C4          (Sohnke SpGr)          R Verify
PLAT791_ALERT_4_G Model has Chirality at C11         (Sohnke SpGr)          S Verify
PLAT910_ALERT_3_G Missing # of FCF Reflection(s) Below Theta(Min).          1 Note
PLAT912_ALERT_4_G Missing # of FCF Reflections Above STh/L=  0.600        170 Note
PLAT958_ALERT_1_G Calculated (ThMax) and Actual (FCF) Lmax Differ.          3 Units
PLAT978_ALERT_2_G Number C-C Bonds with Positive Residual Density.          8 Info
PLAT982_ALERT_1_G The  I-f'=   -0.4119 Deviates from  IT-value =      -0.4742 Check
PLAT983_ALERT_1_G The  I-f"=    1.8326 Deviates from  IT-Value =       1.8119 Check
```

0 **ALERT level A** = Most likely a serious problem - resolve or explain
0 **ALERT level B** = A potentially serious problem, consider carefully
1 **ALERT level C** = Check. Ensure it is not caused by an omission or oversight
13 **ALERT level G** = General information/check it is not something unexpected

5 ALERT type 1 CIF construction/syntax error, inconsistent or missing data
2 ALERT type 2 Indicator that the structure model may be wrong or deficient
1 ALERT type 3 Indicator that the structure quality may be low
6 ALERT type 4 Improvement, methodology, query or suggestion
0 ALERT type 5 Informative message, check

It is advisable to attempt to resolve as many as possible of the alerts in all categories. Often the minor alerts point to easily fixed oversights, errors and omissions in your CIF or refinement strategy, so attention to these fine details can be worthwhile. In order to resolve some of the more serious problems it may be necessary to carry out additional measurements or structure refinements. However, the purpose of your study may justify the reported deviations and the more serious of these should normally be commented upon in the discussion or experimental section of a paper or in the "special_details" fields of the CIF. checkCIF was carefully designed to identify outliers and unusual parameters, but every test has its limitations and alerts that are not important in a particular case may appear. Conversely, the absence of alerts does not guarantee there are no aspects of the results needing attention. It is up to the individual to critically assess their own results and, if necessary, seek expert advice.

Publication of your CIF in IUCr journals

A basic structural check has been run on your CIF. These basic checks will be run on all CIFs submitted for publication in IUCr journals (*Acta Crystallographica*, *Journal of Applied Crystallography*, *Journal of Synchrotron Radiation*); however, if you intend to submit to *Acta Crystallographica Section C* or *E* or *IUCrData*, you should make sure that full publication checks are run on the final version of your CIF prior to submission.

Publication of your CIF in other journals

Please refer to the *Notes for Authors* of the relevant journal for any special instructions relating to CIF submission.

PLATON version of 18/05/2022; check.def file version of 17/05/2022

Datablock No2020-3-C1-1 - ellipsoid plot

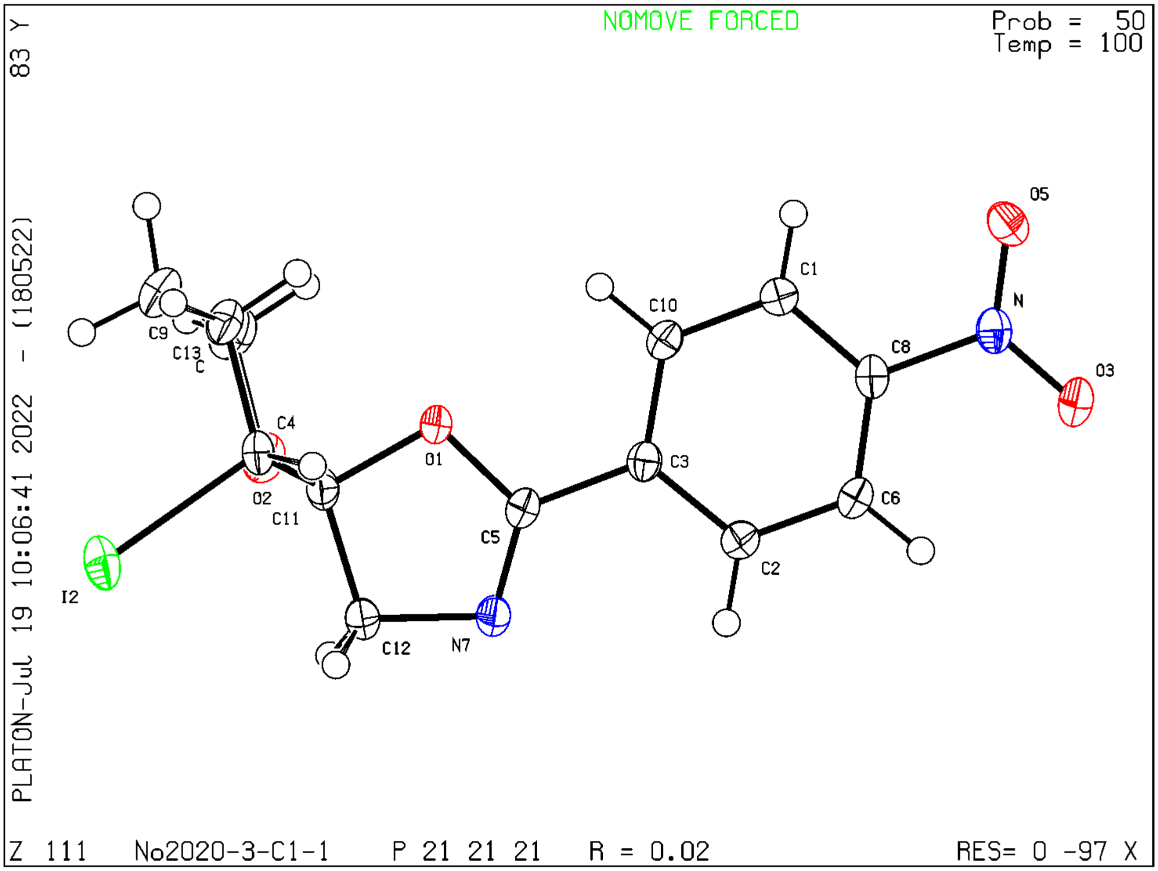

Supplement: Supplementary file 1 [file DataSheet1.zip › 2-Cif check file for 2a.PDF]
